# Characterizing PFAS hazards and risks: a human population-based in vitro cardiotoxicity assessment strategy

**DOI:** 10.1186/s40246-024-00665-x

**Published:** 2024-09-02

**Authors:** Lucie C. Ford, Hsing-Chieh Lin, Yi-Hui Zhou, Fred A. Wright, Vijay K. Gombar, Alexander Sedykh, Ruchir R. Shah, Weihsueh A. Chiu, Ivan Rusyn

**Affiliations:** 1 Department of Veterinary Physiology and Pharmacology, TAMU 4466, Texas A&M University, College Station, TX 77843-4466 USA; 2 Department of Biological Sciences and Statistics, North Carolina State University, Raleigh, NC 27695 USA; 3 Bioinformatics Research Center, North Carolina State University, Raleigh, NC 27695 USA; 4 Sciome LLC, Durham, NC 27713 USA

## Abstract

Per- and poly-fluoroalkyl substances (PFAS) are emerging contaminants of concern because of their wide use, persistence, and potential to be hazardous to both humans and the environment. Several PFAS have been designated as substances of concern; however, most PFAS in commerce lack toxicology and exposure data to evaluate their potential hazards and risks. Cardiotoxicity has been identified as a likely human health concern, and cell-based assays are the most sensible approach for screening and prioritization of PFAS. Human-induced pluripotent stem cell (iPSC)-derived cardiomyocytes are a widely used method to test for cardiotoxicity, and recent studies showed that many PFAS affect these cells. Because iPSC-derived cardiomyocytes are available from different donors, they also can be used to quantify human variability in responses to PFAS. The primary objective of this study was to characterize potential human cardiotoxic hazard, risk, and inter-individual variability in responses to PFAS. A total of 56 PFAS from different subclasses were tested in concentration-response using human iPSC-derived cardiomyocytes from 16 donors without known heart disease. Kinetic calcium flux and high-content imaging were used to evaluate biologically-relevant phenotypes such as beat frequency, repolarization, and cytotoxicity. Of the tested PFAS, 46 showed concentration-response effects in at least one phenotype and donor; however, a wide range of sensitivities were observed across donors. Inter-individual variability in the effects could be quantified for 19 PFAS, and risk characterization could be performed for 20 PFAS based on available exposure information. For most tested PFAS, toxicodynamic variability was within a factor of 10 and the margins of exposure were above 100. This study identified PFAS that may pose cardiotoxicity risk and have high inter-individual variability. It also demonstrated the feasibility of using a population-based human in vitro method to quantify population variability and identify cardiotoxicity risks of emerging contaminants.

**Supplementary Information:**

The online version contains supplementary material available at 10.1186/s40246-024-00665-x.

## Introduction

Per- and poly-fluoroalkyl substances (PFAS) have utility in many industrial and consumer-use products because of their repellant and lubricating properties, as well as resistance to high temperatures. High-volume production, wide-spread use, and resistance to degradation has resulted in ubiquitous presence of PFAS in the environment; humans can be exposed through water, soil, food and by inhalation [1–6]. Indeed, exposure and biomonitoring studies detected PFAS in nearly all tested human and environmental samples [7, 8]. Even though there are thousands of PFAS on various chemical inventories, there is little to no data on the potential human health hazards for the vast majority of these substances [9, 10]. Some PFAS have been shown to be harmful to humans [11–13]; however, only a handful of these have been studied in detail. Even fewer PFAS have sufficient data to establish causal associations with human disease because approaches relying on epidemiological data and studies in animals are not possible to scale up to dozens or hundreds of PFAS that are produced in large volumes.

Cell-based assays have been proposed as a sensible approach to test various PFAS in a time- and resource-efficient manner [10, 14, 15]. Many PFAS have already been tested in cells from the liver, immune, nervous, and other organ systems [16–21]. Still, other tissues/cell types have been suggested to be potential targets for PFAS toxicity in humans [8] and additional testing is needed. Recent studies using diverse human cell types from potential target organs (human induced pluripotent stem cell (iPSC)-derived hepatocytes, neurons, and cardiomyocytes, primary human hepatocytes, endothelial and HepG2 cells) showed that PFAS demonstrated cell-specific activity highlighting the potential complexity of their effects [22, 23]. Interestingly, by evaluating PFAS effects on different cell types, these studies corroborated previous mechanistic and laboratory animal research suggesting that PFAS could contribute to cardiovascular disease [24]. Because cardiovascular disease is a leading public health burden worldwide and environmental risk factors are known to contribute to the global burden of cardiovascular disease [25], additional studies of potential effects of PFAS on human cardiomyocytes are warranted.

iPSC-derived cardiomyocytes are not only a powerful tool for determining what drugs and chemicals might exert adverse effects on the human heart [26–28], but they can also be used as a population-wide human in vitro model to better understand susceptibility [29, 30]. Many previous studies showed that these cells are an effective model for characterization of cardiotoxicity hazard, risk, and population variability of environmental chemicals and can be used in a medium- to high-throughput format to test large numbers of substances [31–36]. Characterization of inter-individual variability in hazardous effects of chemicals is often an unaddressed need in risk assessment [37], and in vitro methods have been proposed to replace default assumptions on the extent of inter-individual variability with chemical-specific data [38].

Therefore, in this study, we used a human population-based in vitro model of iPSC-derived cardiomyocytes from multiple donors to evaluate the potential cardiotoxicity and quantify the inter-individual variability in responses to a structurally diverse set of 56 PFAS. These data were then interpreted in the context of risk assessment by comparing the observed bioactivity to measured or predicted exposures to establish margins of exposure. The bioactivity data also was used to identify structure-bioactivity relationships that can be used to group these chemicals and infer potential hazards and risks of other PFAS that are yet to be tested [39, 40].

## Experimental methods

### Chemical and biological reagents

Plating and maintenance media for the iPSC-derived cardiomyocytes were obtained from Fujifilm Cellular Dynamics International (Madison, WI). Penicillin-streptomycin (Cat#10378016), Hoechst 33,342 (Cat# H3570), and MitoTracker Orange (Cat# M7510) were obtained from LifeTechnologies (Grand Island, NY). The EarlyTox Cardiotoxicity Assay Kit (Cat# R8211) was obtained from Molecular Devices (San Jose, CA). Isoproterenol (CAS# 768-59-2), propranolol (CAS# 525-66-6), and sotalol (CAS# 959-24-0), compounds used as positive controls for cardiomyocyte assays, were obtained from Molecular Devices. Tissue-culture treated 384-well black/clear bottom plates were obtained from Corning (Cat# 3764, Kennebunk, ME). Trypan Blue 0.4% solution (Cat# T8154-100ML), and gelatin from porcine skin (CAS# 9000-70-8) were obtained from MilliporeSigma (Cat# T8154-100ML, Burlington, MA). Tetra-octyl ammonium bromide (TAB, CAS# 14866-33-2, cat#D2438) was obtained from SigmaAldrich (St. Louis, MO). Tissue-culture grade dimethyl sulfoxide (DMSO, CAS# 67-68-5, cat# sc-358801) was obtained from Santa Cruz Biotechnology (Dallas, TX).

A panel of human iPSC-derived cardiomyocytes (*n* = 16, Tables 1and S1) were obtained from Fujifilm Cellular Dynamics International (Madison, WI).

**Table 1:** Donor identification and characteristics of iPSC-derived cardiomyocytes used in this study. Additional details can be found in Table S1

Cell Line ID	Source	Sex	Ancestral Background	Batch #	Catalog Number	Lot #
1434	CDI	Female	Mixed African American/ European	1,5,6	CMC-100-010-001	105,008
1565	FDA	Male		5	DDP-CMC-1 × 01565.104	101,514
30,171	CIRM	Female	European	3	CW30171HH1	101,998
1309	CDI			2	DDP-CMC-1 × 103,046	103,046
1531	FDA			2	DDP-CMC-1 × 101,317	101,317
1368	NHLBI			3	DDP-CMC-0.5 × 01368.716	1368.716
30,145	CIRM			4	CW30145AA1	102,118
1392	NHLBI	Male		1	DDP-CMC-0.5 × 01392.734	1392.734
1518	FDA			6	DDP-CMC-1 × 101,421	101,421
1516	FDA	Female	African American	6	DDP-CMC-1 × 01516.102	102,177
1083	NHLBI			1	DDP-CMC-0.5 × 01083.758	1083.758
1535	FDA	Male		3	DDP-CMC-1 × 01535.102	102,176
11,235	CDI			5	DDP-CMC-1 × 11235.106	102,328
1118	NHLBI			4	DDP-CMC-0.5 × 01118.704	1118.704
20,084	CDI	Male	Hispanic/ Latino	2	DDP-CMC-1 × 102,668	102,668
20,032	CIRM	Male	Asian	4	CW20032AA1	102,500

**Fig. 1:**
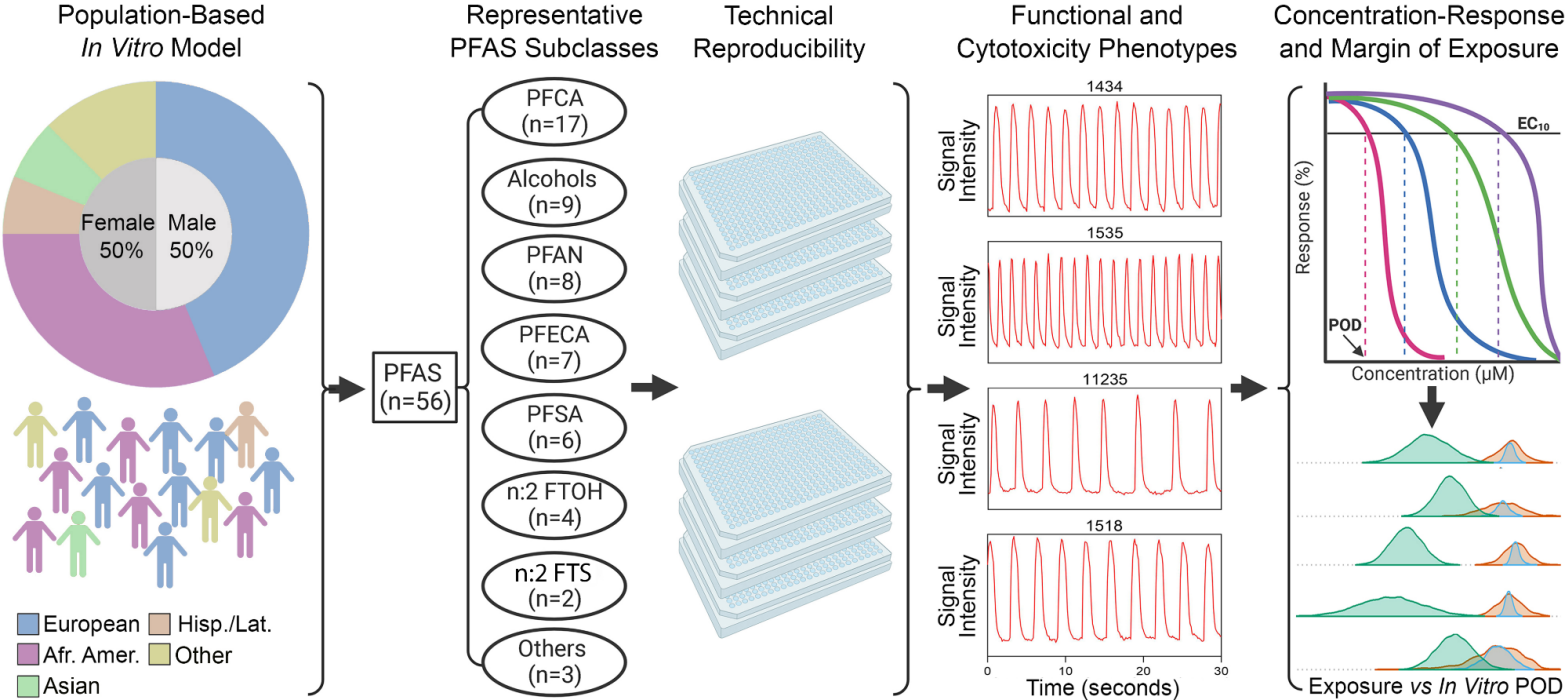
Overall study design to evaluate the potential toxicity of 56 diverse per- and poly-fluoroalkyl substances (PFAS) using human induced pluripotent stem cell (iPSC)-derived cardiomyocytes from multiple donors. Human iPSC-derived cardiomyocytes were from 16 donors and used to test 56 structurally-diverse PFAS in concentration response. Intra- and inter-plate replicates, as well as positive and negative controls, were included to ensure experimental reproducibility. Following exposure to PFAS, functional and cytotoxic phenotypes were evaluated. The concentration-response bioactivity data were used to derive donor-specific phenotypic points of departure (PODs), which were then used to estimate the extent of inter-individual variability and compared to exposure estimates to calculate margins of exposure

These cells were derived from donors with no known or family history of cardiovascular disease (as verified by Fujifilm Cellular Dynamics) and were meant to represent “healthy” individuals. The selection of 16 donors used herein was based on cell availability from the manufacturer and previous analyses that showed that for estimating inter-individual variability, cohorts of around 20 donors are needed [31]. The cell lines used herein were from five race/ethnicity subpopulations that represented various ancestral backgrounds and had equal representation of males and females (Fig. 1). The populations included European, Asian, African American, Hispanic/Latino, and individuals of mixed ancestry (e.g., mixed African American and European).

### Test chemicals

PFAS used herein (see Table 2 and S2 for chemical information, abbreviations, and supplier) were gifted by the US Environmental Protection Agency (Research Triangle Park, NC). These substances were procured from various commercial sources by MRI Global (Kansas City, MO).

**Table 2:** PFAS used in this study. Additional chemical details can be found in Table S2

Subclass Assignment		Abbreviated Name	Chemical Name
ALCOHOL		PFUd2OH	1-(Perfluorofluorooctyl)propane-2,3-diol
		PFPOH	1 H,1 H,5 H-Perfluoropentanol
		PFBOH	1-Pentafluoroethylethanol
		AmFPrOH	2-Aminohexafluoropropan-2-ol
		PFHp2OH	3-(Perfluoro-2-butyl)propane-1,2-diol
		7 H 6:1 FTOH	Dodecafluoroheptanol
		C7F3ETOH	Fluorinated triethylene glycol monomethyl ether
		HpFBOH	Heptafluorobutanol
		CFHx2OH	Hexafluoroamylene glycol
n:2 FTOH		4:2 FTOH	4:2 Fluorotelomer alcohol
		6:1 FTOH	6:1 Fluorotelomer alcohol
		6:2 FTOH	6:2 Fluorotelomer alcohol
		8:2 FTOH	8:2 Fluorotelomer alcohol
n:2 FTS		6:2 FTS	6:2 Fluorotelomer sulfonic acid
		8:2 FTS	8:2 Fluorotelomer sulfonic acid
PFAN	PFAM	PFHpAM	Heptafluorobutyramide
		PFNAM	Nonafluoropentanamide
		PFOAM	Perfluorooctanamide
		PFO2AM	Octafluoroadipamide
		PFPAM	Perfluoropentanamide
	FASA	PFHxSA	Perfluorohexanesulfonamide
		MeFOSE	N-Methyl-N-(2-hydroxyethyl)perfluorooctanesulfonamide
	PFAA	PFOAMD	Perfluorooctanamidine
PFCA		4 H-PFBA	2,2,3,3,4,4-Hexafluorobutanoic acid
		5:3 FTCA	2 H,2 H,3 H,3 H-Perfluorooctanoic acid
		PFIpOA	3-(Perfluoroisopropyl)-2-propenoic acid
		TFPrOA	3,3-Bis(trifluoromethyl)-2-propenoic acid
		7:3 FTCA	3-Perfluoroheptylpropanoic acid
		Cl-PFNA	9-Chloro-perfluorononanoic acid
PFCA		NH4PFOA	Ammonium perfluorooctanoate
		PFHx2OA	Octafluoroadipic acid
		PFBA	Perfluorobutanoic acid
		PFDA	Perfluorodecanoic acid
		PFHpA	Perfluoroheptanoic acid
		PFHxA	Perfluorohexanoic acid
		PFNA	Perfluorononanoic acid
		PFOA	Perfluorooctanoic acid
		PFPeA	Perfluoropentanoic acid
		PFPrA	Perfluoropropanoic acid
		PFUnDA	Perfluoroundecanoic acid
PFECA		MePF2ETOA	Methylperfluoro(3-(1-ethenyloxypropan-2-yloxy)propanoate)
		PFMBA	Perfluoro(4-methoxybutanoic) acid
		PFPE-6	Perfluoro-3,6,9-trioxatridecanoic acid
		NFDHA	Perfluoro-3,6-dioxaheptanoic acid
		PFHx2Et2OA	Perfluoro-3,6-dioxaoctane-1,8-dioic acid
		PFMPA	Perfluoro-3-methoxypropanoic acid
		PFPE-1	Perfluoro-4-isopropoxybutanoic acid
PFSA		ET-PFBS	2,2,2-Trifluoroethyl perfluorobutanesulfonate
		4:2 FTS	4:2 Fluorotelomer sulfonic acid
		PFBS	Perfluorobutanesulfonic acid
		PFHxS	Perfluorohexanesulfonic acid
		PFOS	Perfluorooctanesulfonic acid
		PFBS-K	Potassium perfluorobutanesulfonate
Other	FTCA	3:3 FTCA	3:3 Fluorotelomer carboxylic acid
	PFPA	8:2 FTPA	((Perfluorooctyl)ethyl)phosphonic acid
	non-PFAA	PFHx2ON	3 H,3 H-Perfluoro-2,4-hexanedione

Chemicals were supplied frozen at concentrations of ∼20 mM in 100% tissue culture grade DMSO and stored at − 80 °C until use. A total of 56 PFAS were tested in this study and were selected to represent various chemical classes, chain length, and to include both legacy and more contemporary substances (see PCA of PFAS tested herein versus OECD list of PFAS, Fig. S1). Selected PFAS represent 8 subclasses which include: (i) PFCA (*n* = 17), (ii) alcohols (*n* = 9), (iii) PFAN (*n* = 8), (iv) PFECA (*n* = 7), (v) PFSA (*n* = 6), (vi) n:2 FTOH (*n* = 4), (vii) n:2 FTS (*n* = 2), (viii) others (*n* = 3) (Fig. 1). From stock solutions, a master plate (200×) was prepared where each chemical was serially (10×) diluted with 100% DMSO three times from a top concentration of 20 mM. In addition, positive controls were included on each master plate (see plate design Fig. S2). These plates were stored sealed at − 80 °C until experiments.

### In vitro experiments

Human iPSC-derived cardiomyocyte cell lines were divided into 6 testing batches, sex and ancestral backgrounds were balanced among batches. The cell culture conditions were performed as described in previous publications [41, 42]. Briefly, the tissue-culture treated 384-well plates were coated with 25 µL/well of 0.1% (w/v) gelatin solution (gelatin from porcine skin diluted in cell culture grade water), the plates were then incubated for 2 h at 37 °C and 5% CO_2_. Cells were removed from liquid nitrogen storage and thawed in a 37 °C water bath for 3 min. Cell concentration calculations accounted for manufacturer-provided viability and plating efficiency estimates; in addition, the cells were counted using the Cellometer™ Auto T4 Plus (Nexcelom Bioscience, Lawrence, MA) to confirm live cell count and viability prior to plating. The cell suspension was then added dropwise into room temperature plating medium containing 1:500 penicillin/streptomycin solution and the volume of cell culture medium was adjusted to a concentration of 2 × 10^5^ cells/mL. Immediately before plating, the gelatin solution was aspirated from all wells in the plates, and 25 µL of the cell suspension was added into each well in the 384-well plate (excluding the outer wells), resulting in a seeding density of 5,000 cells/well in 308 wells/plate. The outer wells of each plate were filled with 65 µL of sterile phosphate-buffered saline solution. The plates were kept at room temperature for 30 min to avoid “edge-effect”, they were then incubated at 37 °C and 5% CO_2_. After 48-h post-plating, 17.5 µL of plating medium was removed, and exchanged with 32.5 µL of maintenance medium containing 1:500 penicillin/streptomycin solution (complete maintenance medium), for a total volume of 40 µL/well. The plates were incubated for a duration of 13 days; every 48–72 h 25 µL of maintenance medium was exchanged with 25 µL/well of fresh pre-warmed complete maintenance medium. Prior to media changes, the cells were inspected under the microscope to verify that the cells in all wells began to exhibit spontaneous and synchronous beating (typically around day 7 post-plating). In the evening of day 13 post-plating, the entire volume in each well was aspirated (carefully to ensure that the monolayer was not disturbed) and replaced with 25 µL/well of fresh pre-warmed complete maintenance medium. The chemical assays were performed on day 14.

### Functional phenotyping of iPSC-derived cardiomyocytes

The EarlyTox Cardiotoxicity Ca^2+^ flux assay kit was used to evaluate the functional effects of the PFAS on the iPSC-derived cardiomyocytes as demonstrated previously [41, 42]. Intracellular Ca^2+^ flux is measured using a series of time-resolved images (8 frames per s) using FLIPR Tetra Cellular Screening System (Molecular Devices) as a quantitative functional readout based on a fluorescent Ca^2+^ probe. Ca^2+^ flux reads are recorded at baseline prior to chemical treatment and after chemical exposures. As indicated by the manufacturer’s (Molecular Devices) protocol, the assay was performed by first preparing the Ca^2+^ dye by equilibrating the reagents in a 37 °C water bath. An equal volume (25 µL) of the Ca^2+^ dye reagent was added manually to each well in the plate with cells, resulting in a total volume of 50 µL/well. The plates were then incubated at 37 °C and 5% CO_2_ for 2 h, and imaged for the baseline Ca^2+^ flux reading in the entire plate simultaneously using the FLIPR Tetra Cellular Screening System (Molecular Devices) instrument that was kept at 37 °C. Ca^2+^ flux was recorded at the rate of 8 frames per s for 100 s (*n* = 800 total images) with stage temperature = 37 °C, λ_exc_ = 470–495 nm, λ_em_ = 515–575 nm, gain = 2000, and exposure time = 0.05 s.

Following the baseline read recording, chemicals were added to each well as follows. On the day of the experiment (day 14 of iPSC-derived cardiomyocyte culture), the chemical master plate (200×) was diluted 40-fold in cardiomyocyte maintenance medium to yield a 5× working solution in 2.5% DMSO for each test compound. Then, 12.5 µL of the 5× working solution was added simultaneously to each well with cells already containing 50 µL (25 µL maintenance media and 25 µL of calcium flux dye) using the automated liquid handler in the FLIPR Tetra (Molecular Devices) to yield the final concentrations of 0.1, 1, 10, 100 µM (each in 0.5% DMSO) for each test substance. The concentration of 0.5% DMSO in assay wells was consistent with previous studies and it itself has no effect on the viability of cardiomyocytes [42, 43]. FLIPR Tetra settings were set to mix and then transfer test chemicals from the 5× plate to the plate with cells at height = 40 µL, speed = 1 µL/s, and removal speed = 6 mm/s. After the chemical addition, the cells were then incubated at 37 °C and 5% CO_2_ for 90 min. The exposure duration was based on optimized protocols from previous studies that evaluated functional readouts after various exposure durations [35, 43]. After the 90 min incubation with the chemicals, the Ca^2+^ flux again was simultaneously recorded on all wells of the plate.

### Cytotoxicity phenotyping of iPSC-derived cardiomyocytes

After the 90 min Ca^2+^ flux measurements were completed as detailed above, cytotoxicity phenotypes were evaluated using high-content imaging in the ImageXpress Micro Confocal Imaging System (Molecular Devices) as detailed previously [35]. The high-content imaging assay was performed by first aspirating the total volume of maintenance medium containing the Ca^2+^ dye reagent and replacing it with 25 µL/well of pre-warmed staining solution. The staining solution comprised of fluorescent probes for nuclei (2.2 µg/mL Hoechst 33342) and mitochondria (0.2 M MitoTracker Orange) in complete maintenance medium. Upon adding the staining solution to the plates, they were placed in the incubator for 15 min, the stain was then fully aspirated and replaced with 25 µL/well of pre-warmed complete maintenance medium before proceeding to image acquisition. Images were captured at 10× magnification using the following fluorescent filters: DAPI (Hoechst 33342 for nuclear staining), TRITC (MitoTracker Orange for mitochondrial staining), and FITC (Ca^2+^ dye).

### Image analysis and data processing

The data collected from both the Ca^2+^ functional assay and the cytotoxicity high-content imaging assay were processed using algorithms detailed elsewhere and used for concentration-response analysis [35]. A total of 5 phenotypes (4 functional and 1 cytotoxic) have been selected for in vitro cardiotoxicity assessments based on the previous analyses of human relevance of iPSC-derived cardiomyocyte in vitro readouts [26, 44]. The Ca^2+^ flux assay data was processed using a custom script [26]. The Ca^2+^ flux data yields four functional cardiotoxic phenotypes that were evaluated: “[+]/positive chronotrope”, representing an increase in beating frequency compared to baseline unpaced rate; “[-]/negative chronotrope”, a decrease in beating frequency; “decay-to-rise ratio”, the ratio of the time from peak maximum to baseline to the time from baseline to peak maximum which is representing QT interval length; and “asystole”, indicative of cell quiescence where the peak frequency reaches zero but there is no evidence of cell death. The cytotoxicity phenotype was assessed using the image processing and quantification methods on the multi-wavelength cell scoring module on the MetaXpress software (Molecular Devices). The total cell number parameter was used as the measure of “cytotoxicity”, which was quantified as the total number of nuclei in a representative imaging field.

### Assessment of assay reproducibility

Reproducibility of these multi-plate/-well experiments was evaluated both within and among plates using negative and positive controls, intra-plate replicates, and inter-plate replicates. Within each plate, in addition to the negative (vehicle (0.5% DMSO) [*n* = 15] and media [*n* = 5] wells) and 3 positive controls (isoproterenol, sotalol, and propranolol), 6 chemicals were tested as intra-plate replicates in concentration-response: perfluorooctanoic acid (PFOA) 3,3-bis(trifluoromethyl)-2-propenoic acid (TFPrOA), perfluoro-3,6,9-trioxatridecanoic acid (PFPE-6), 1 H,1 H,5 H-perfluoropentanol (PFPOH), dodecafluoroheptanol (7 H 6:1FTOH), and perfluoro-hexanesulfonamide (PFHxSA). Inter-plate reproducibility was evaluated with plate replicates.

The raw phenotypic values for intra-plate replicate chemicals and positive controls were used to assess the Pearson correlation coefficients and associated p-values. Inter-plate reproducibility was assessed from the Pearson correlation coefficient and corresponding p-values of the identical wells using the raw data from each cell line that was screened on multiple plates. Further, in addition to examining the certificates of analysis from the manufacturer, we ensured that each well was functional and exhibited expected cardiomyocyte phenotypes after plating. For this, both negative and positive control data were evaluated. The positive compounds included: isoproterenol, propranolol, and sotalol; all of these were tested in concentration response (0.1, 1, 10, and 100 µM) and have been well characterized as positive controls using the iPSC-derived cardiomyocyte model [42, 43].

### Bayesian population-based concentration-response modeling

First, raw phenotypic data in each experimental well were normalized to the average of the wells containing vehicle (0.5% DMSO). After normalization, quality control was assessed by verifying the positive and negative control wells, the data was then fit to a concentration-response model for each chemical across the various phenotypes of interest using a hierarchal Bayesian random effects Hill model as described elsewhere [26, 44]. The concentration-response profiles were used to derive chemical- and phenotype-specific points of departure (PODs). PODs for positive/negative chronotropes were defined as the concentration at which the response increased/decreased peak frequency by 5% from vehicle controls (media with 0.5% DMSO, EC_05_). Similarly, the POD for QT prolongation was defined as the concentration at which there was a 5% increase in the decay-to-rise ratio from the vehicle controls (EC_05_). The POD for asystole was defined as the concentration at which there was a 95% decrease from the vehicle controls, represented by the EC_95_. The cytotoxic POD was defined by a 10% decrease in the total number of cells from the vehicle controls (EC_10_), consistent with previous studies [45, 46].

For Bayesian modeling, all parameters in the Hill model were fitted under natural-log transformation to ensure the parameters were strictly positive. The prior distribution settings for parameters were the same as those previously described elsewhere [44]. Sampling of the posterior distribution for each parameter was conducted using Markov chain Monte Carlo simulation with the *Rstan* package (version 2.21.5). Each chemical-phenotype combination simulation consisted of four independent Markov chains with 8,000–32,000 iterations. All the iterations were processed with the first half discarded and the last half applied to evaluate convergence. The estimated potential scale reduction factor (R̂) ≤ 1.2 [47] was used to diagnose the convergence of each simulation and to determine the final number of iterations needed for each chemical-phenotype combination. When converged, a total of 1,000 posterior samples extracted, consisting of 250 randomly sampled iterations from each of four chains, and utilized to derive POD and other further analysis. PODs for the population 5th (sensitive) and 50th (median) %iles and for each individual donor were derived (Tables S3–S5).

### Data integration using toxicological priority index (ToxPi) approach

The ToxPi Graphical User Interface (ToxPi GUI) [48], was utilized for data integration and visualization. Following the standard ToxPi data analysis protocol, we used the donor-specific PODs across all phenotypes as the quantitative input for the bioactivity profiling, the input POD data can be found in the supplementary material (Table S5). After inputting the data into the ToxPi GUI, a ToxPi score is calculated and assigned to each PFAS. ToxPi scores range on a 0 to 1 scale, with 0 representing the highest PODs (i.e., the lowest observed bioactivity) and 1 representing the lowest PODs (i.e., the highest observed bioactivity). The ToxPi scores are then used to rank the chemicals and identify which phenotype is the most/least bioactive (Table S6).

### Phenotype- and chemical-specific activity calls

Results for each chemical-phenotype combination was determined to be active based on criteria similar to those previously detailed [44]: (i) the convergence needed to be adequate with R̂ ≤ 1.2; (ii) the coefficient of variability for model fit was required to be less than 20%; (iii) the POD estimate for the population median individual need to be lower than 3× the maximum tested concentration (100 µM); and (4) 5th %ile of maximum response (E_max_) was larger than a 10% change. Results for specific chemical-phenotype combinations that meet all the above-mentioned criteria were considered “active” for the cardiotoxicity hazard at the population median level for the given endpoint.

Several *additional* criteria were applied to determine if the results were sufficiently robust for estimating population variability: [5] the 90% confidence interval for the population median POD spanned less than 100-fold, [6] the 90% confidence interval for the sensitive (population 5th %ile) POD spanned < 100 fold, and [7] at least half of the individuals had (non-zero) data at three concentrations in addition to controls. Toxicodynamic variability factors (below) were derived for chemicals-phenotype combinations that fulfill these additional criteria.

### Derivation of toxicodynamic variability factors (TDVF_05_)

Once the simulation results of a PFAS for a given phenotype were found to fulfill all the criteria for population variability analysis, population variability in the POD can be derived. To analyze inter-individual variability in responses to the PFAS, the toxicodynamic variability factor at population 5th %ile (TDVF_05_) is defined as the ratio of the POD for the median individual to the POD for the most sensitive 5th %ile individual. Using asystole as an example, its POD was defined as the EC_95_ corresponding to a 95% decreasing peak frequency. Here, the symbol $\:{EC}_{95}^{50}$ is the estimated EC_95_ for the median individual and $\:{EC}_{95}^{05}$ is the estimated EC_95_ for the sensitive individual (5th %ile); then, the equation for the TDVF is expressed as $\:{{TDVF}_{05}=\:{EC}_{95}^{50}/EC}_{95}^{05}$. The default uncertainty factor for toxicodynamic variability was considered to be 10^1/2^ = 3.16 [49] and was used as a benchmark to compare against our derived TDVF_05_. Derived TDVF_05_ values can be found in Table S7.

### Derivation of margins of exposure using a probabilistic in vitro-to-in vivo extrapolation

Margins of exposure (MOE) for the PFAS were calculated to further characterize the potential cardiotoxic risk in responses to these chemicals. MOE assessments are an equivalent measurement to margin of safety (MOS) evaluations that are used for pharmaceutical compounds [50]. The human exposure data of the tested PFAS were sourced from the U.S EPA Computational Toxicology Chemistry Dashboard (CompTox Dashboard) [51]. [51], and consisted of the median estimate and 95th %ile confidence bound of the predicted population median exposure in mg/kg body weight/day. These were fit to an equivalent lognormal distribution, and values were sampled via Monte Carlo simulation. To compare to the POD values, the oral exposure estimates were subsequently converted to a steady-state plasma concentration (C_ss_) using the following equation:

$$\begin{aligned}{C}_{ss,\:\text{o}\text{r}\text{a}\text{l}\:\text{e}\text{x}\text{p}\text{o}\text{s}\text{u}\text{r}\text{e}\:\text{e}\text{s}\text{t}\text{i}\text{m}\text{a}\text{t}\text{e}\text{s}}\left(\mu\:M\right)&=\text{o}\text{r}\text{a}\text{l}\:\text{e}\text{x}\text{p}\text{o}\text{s}\text{u}\text{r}\text{e}\:\text{e}\text{s}\text{t}\text{i}\text{m}\text{a}\text{t}\text{e}\text{s}\:(\text{m}\text{g}/\text{k}\text{g}\:\text{B}\text{W}/\text{d}\text{a}\text{y})\\ &\times\:\frac{{C}_{ss,\:1\:\text{m}\text{g}/\text{k}\text{g}\:\text{B}\text{W}/\text{d}\text{a}\text{y}}\left(\mu\:M\right)}{1\:\text{m}\text{g}/\text{k}\text{g}\:\text{B}\text{W}/\text{d}\text{a}\text{y}\:}\end{aligned}$$

Where $\:{C}_{ss,1\:\text{m}\text{g}/\text{k}\text{g}\:\text{B}\text{W}/\text{d}\text{a}\text{y}}$ means the C_ss_ under daily oral dosing with 1 mg/kg-day of the given chemical. The values of $\:{C}_{ss,1\:\text{m}\text{g}/\text{k}\text{g}\:\text{B}\text{W}/\text{d}\text{a}\text{y}}$ were obtained by two alternative methods: (i) if the chemical is available from httk database, the *parameterize_steadystate* function was executed to extract parameters used in the three-compartment steady state (3compartmentss) equation in R httk package [52] (version 2.2.1), then these parameters were used as input into 3compartmentss equation; and (ii) the fraction unbound in plasma as detailed elsewhere [53] and [54] was used in the same C_ss_ equation with the assumption of no hepatic clearance. In addition to the predicted C_ss_ values, biomonitoring blood concentrations were also collected from the literature and used as exposure estimates (details are shown in Table S8).

Two types of POD distributions for each PFAS and phenotype were constructed by Monte Carlo sampling from the population median POD and a “random” individual POD. The “random” individual POD was estimated by randomly sampling the population medians, population variances and Z-scores for each parameter. Note that only for the chemicals fulfilling the criteria for an active call of population variability did we derive the “random” individual POD. MOE estimates were calculated for the chemicals with available exposure estimates and were bioactive in at least one phenotype. Based on the types of PODs, two MOEs were calculated as follows: (i) the MOE for population median, was estimated by dividing the 5th %ile confidence bound POD from the population median distribution by the 95th %ile confidence bound internal concentration (C_ss_ or biomonitoring value) from exposure to PFAS; and (ii) the MOE for random individual, estimated by dividing the 5th %ile POD from the random individual distribution by the 95th %ile confidence bound internal concentration (C_ss_ or biomonitoring value) from exposure to PFAS. If a given chemical has more than one “active” phenotype, the MOE was calculated by using the most sensitive (lowest) POD across all phenotypes. The minimum MOEs were calculated by using two types of C_ss_ and biomonitoring blood concentration were chosen for overall cardiotoxicity risk characterization. Traditionally, a margin < 1 is considered likely to be of concern; a margin between 1 and 100 is considered of potential concern; and a margin $\:\ge\:\:$100 is considered “protective” in risk assessments of environmental chemicals. The MOE summary data can be found in Table S9 and Fig. S3.

### Correlation analyses and cross-validated predictions for bioactivity using chemical descriptors and physicochemical properties of PFAS

Correlation analyses were conducted to thoroughly evaluate the relationships between PFAS structure (as described by a diverse array of chemical molecular descriptors) and effects on cardiomyocytes from each donor. For these analyses, we used Saagar descriptors [55], a collection of diverse chemical sub-structures (i.e., atoms, atom pairs, and local “motifs” that are searched and counted in each query molecule). Open (Quantitative) Structure-activity/property Relationship App (OPERA) physicochemical descriptors were pulled from the National Institute of Health (NIH) Integrated Chemical Environment (ICE) database and Saagar descriptors were derived from basic chemical functionalities, including metrics such as alkyl halogen counts, di-halogen atom pairs, etc. The overall chemical matrix for both OPERA (9 OPERA descriptors and molecular weight, formula, and carbon chain length) and Saagar (834) descriptors for tested PFAS is provided in Table S10 and the bioactivity data matrix (arranged by phenotype and donor) is provided in Table S11. The 5 bioactivity phenotypes, were evaluated by donor and we also computed minimum PODs for each phenotype across all donors, resulting in a final set of 85 bioactivity phenotypes for these analyses. Additional data reduction was performed for Saagar descriptors because the descriptors failing the variation criterion have almost no power to detect associations. The filtering method required at least two samples with a feature to differ from the remaining samples. After applying this filter, 123 Saagar descriptors were retained.

For pairwise correlation analyses (using Saagar and OPERA descriptors separately), we calculated Spearman rank correlations for each set of Saggar descriptors vs. the 5 bioactivity phenotypes, with two-sided p-values adjusted for the multiple testing [56] and considered significant at *q* < 0.1.

For cross-validated prediction analyses, we used a previously reported approach [57] to determine if chemical descriptors (either Saagar or OPERA) could be used predict bioactivity (either a minimum POD across all donors, or responses of the individual donors). This approach is based on multivariate ridge regression with a common penalty parameter across features and *n*-fold cross-validation. To further guard against overfitting, 10,000 permutations of the procedure were performed for each set of descriptors, resulting in an empirical p-value for the correlation between the cross-validated predictions of each phenotype and the actual observed phenotype values. Multiple testing corrections used Holm’s method (denoted as p_adj_) [58] and the Benjamini-Hochberg false discovery rate (BH-FDR, denoted as q-values) corrected for the number of bioactivity phenotypes [56]. The results matrix for the cross-validated predictions can be found in Tables S12-S14 and Figs. S4-S6.

## Results

An overall study design is shown in Fig. 1. We used iPSC-derived cardiomyocytes from 16 donors that were representative of multiple race/ethnicity backgrounds and balanced for sex ratio. Cells were treated in concentration-response (0.1 to 100 µM) with 56 structurally diverse PFAS in 384-well plate format and a number of functional and cytotoxicity phenotypes were collected. After assessing both quality control and reproducibility, the data were then used for concentration-response modeling to derive points of departure (PODs) for each substance/donor and to determine hazard, risk, and inter-individual variability. Specifically, the PODs were used to (i) rank tested chemicals using ToxPi, (ii) quantify the variability in responses to PFAS, and (iii) derive chemical-specific estimate for the margin of exposure (MOE).

**Fig. 2:**
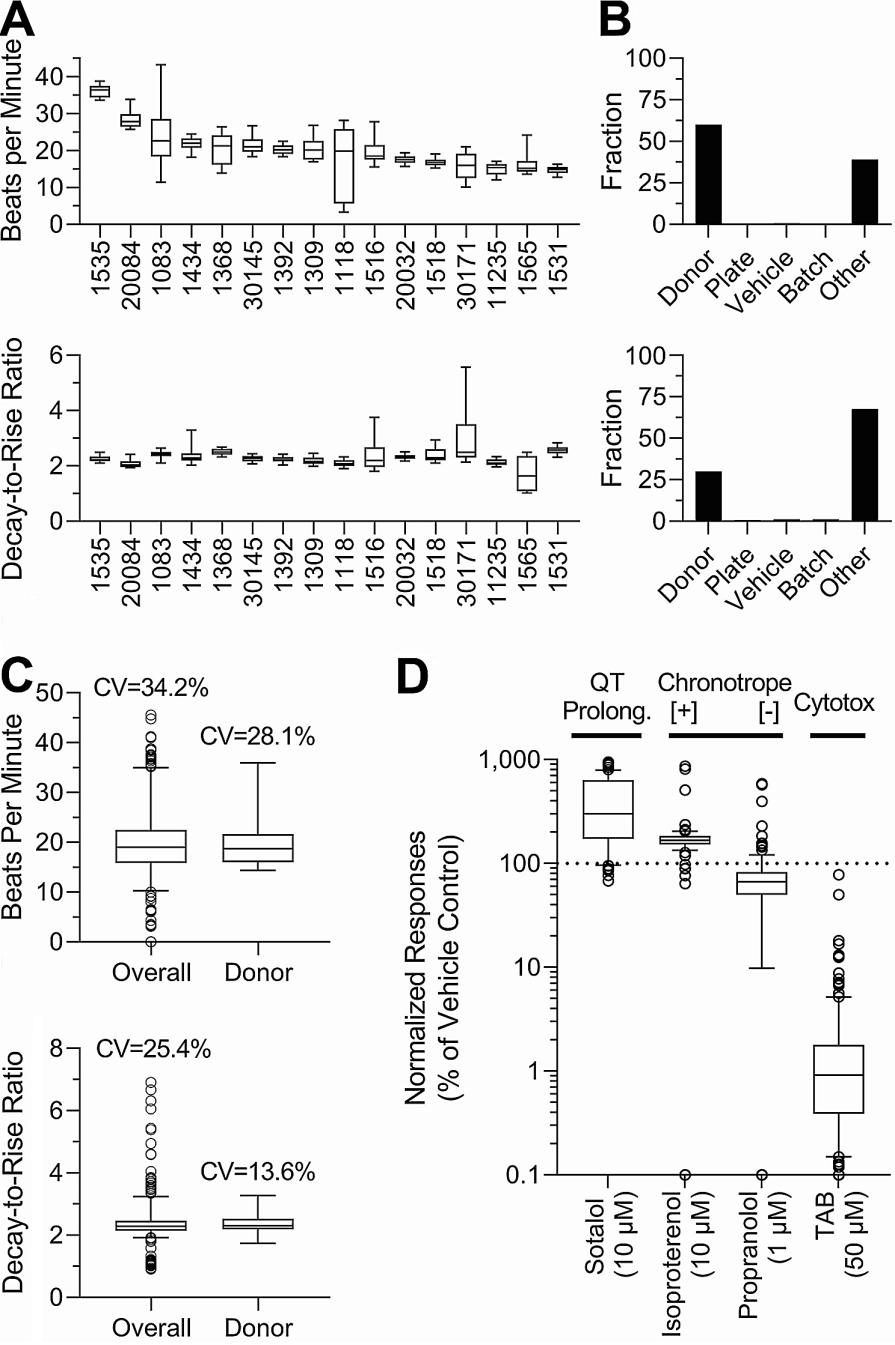
Inter-individual variability in baseline beating parameters and quality control assessment. (**A**) Baseline unstimulated beat rate (beats per min, top) and decay-to-rise ratio (bottom) for vehicle (DMSO, 0.5%) and media-treated wells across all donors were arranged by donor with the highest median baseline BPM rate. Boxes represent the interquartile range, with a line at the median and the whiskers at 10th to 90th %ile. (**B**) Histograms of technical and biological factor contributions for the total observed variability for both beat rate (top) and decay-to-rise ratio (bottom). The contributions to total variability include: Donor = diversity between donors, Plate = inter-plate variability, Vehicle = difference between effects of 0.5% DMSO (vehicle) and cell culture media, and Other = intra-plate variability. (**C**) Coefficients of variability (CV) for total (including both technical and biological) and biological (donor only) replicates. Box plots represent the interquartile range, and the whiskers show the 5th to 95th %ile across all donors. (**D**) Quality control assessment across various phenotype-specific (as indicated above each plot) positive control compounds (names and concentrations are shown). Box plots represent the interquartile range, and the whiskers show the 10th to 90th %ile across all donors

Even though human iPSC-derived cardiomyocytes from multiple healthy donors have been previously shown to exhibit reproducible donor-specific differences in baseline function and drug-induced effects [59], we evaluated functionality and reproducibility of the data in this large-scale experiment (Fig. 2).

Several baseline and drug (positive control)-elicited parameters were collected to ensure data quality for subsequent interpretation of PFAS effects. As expected, [31, 32, 44, 59, 60], iPSC-derived cardiomyocytes from multiple donors demonstrated inter-individual variability in their baseline spontaneous beating parameters. Figure 2A shows examples of inter- and intra-individual variability in spontaneous cell beat rate and decay-to-rise ratio (an indicator of the QT interval). Mean donor-specific beat rate varied between 36 (Donor ID: 1535) and 14 (Donor ID: 1531) beats per min, the range that is similar to that reported previously for the cells from the same donors [59]. Decay-to-rise ratio varied less than the beat frequency, again similar to previously reported values. Figure 2B shows histograms of relative technical and biological contributions to total observable variability for these two representative phenotypes; for the beat rate, inter-individual variability was the dominant contributor to overall variability, it was also s most dominant factor for the decay-to-rise ratio phenotype. Figure 2C shows that inter-individual variability was the dominant contributor to overall variability for beat frequency (28.1% coefficient of variability (CV) for the donor vs. 34.2% CV for total variability) and decay-to-rise ratio (13.6% vs. 25.4%), with very little contribution from technical variability (plate, vehicle, and batch). Finally, for each donor, cardio-specific positive controls, including sotalol (inducing long QT), isoproterenol (increasing the beat rate), and propranolol (decreasing the beat rate) were used, and compared to the negative controls – vehicle (DMSO 0.5%) and media-only wells (Fig. 2D). Even though expected donor-specific responses to the positive control drugs were observed, the population median values were affected in accord with the known pharmacological (isoproterenol and propranolol) and pathological (sotalol) effects of these drugs. Overall, these experiments confirmed the functionality of the cells and the reproducibility of the overall experiment. Inter-plate reproducibility was assessed for donors with plate replicates, see Table S15 for inter-plate replicate comparisons.

**Fig. 3:**
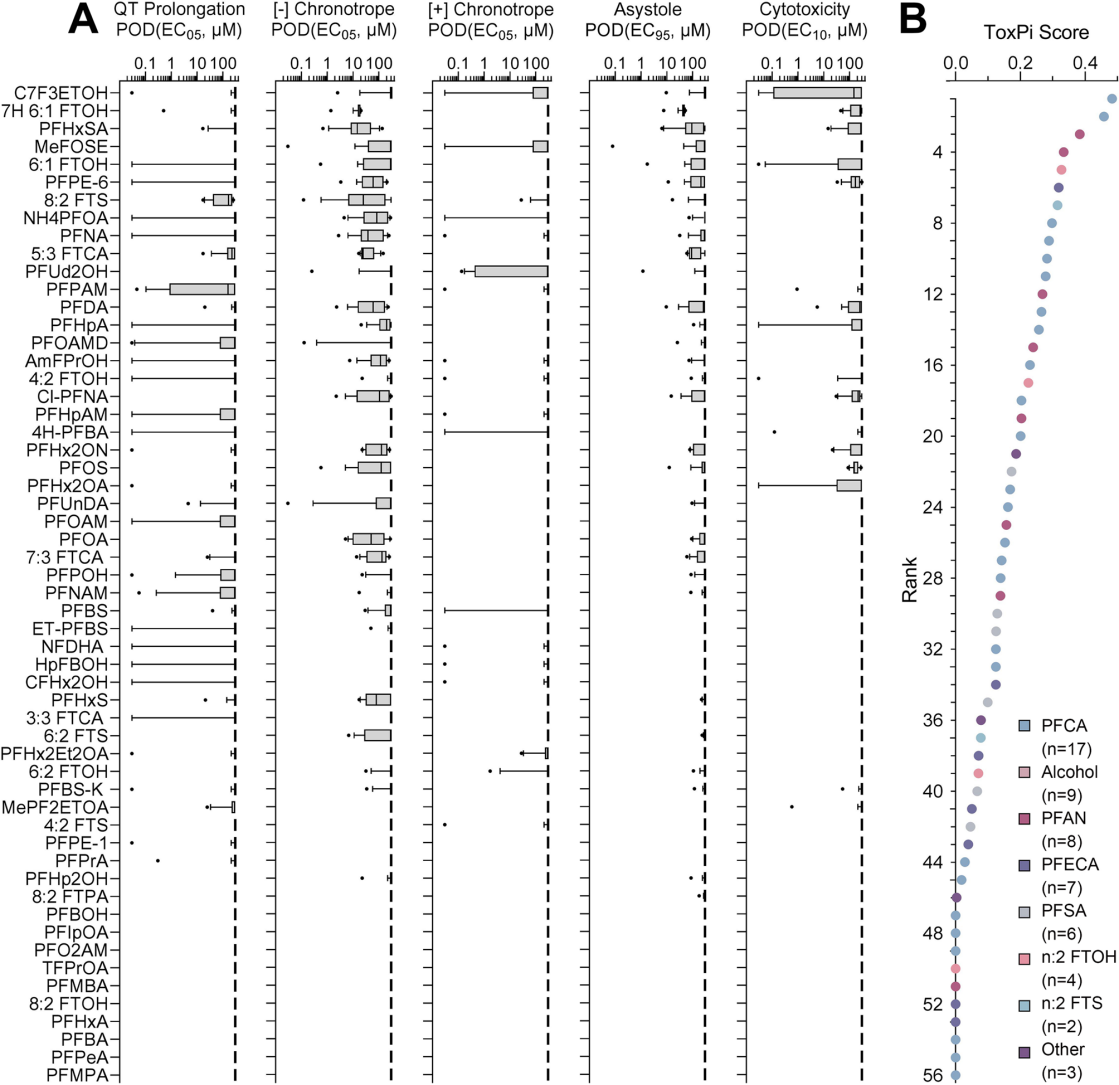
PFAS-specific effects on iPSC-derived cardiomyocytes and variability among donors. (**A**) PODs for five phenotypes are shown and chemicals are sorted by the overall ToxPi score (**B**). For each phenotype and PFAS, box-and-whisker plots include data from all tested donors. Boxes show the interquartile range, with a line at the median and the whiskers illustrate the 10th to 90th %ile. (**B**) The PODs for all five phenotypes were integrated (equal weight) into a ToxPi score for each tested PFAS as described in the Methods. A higher score (and rank) indicates higher potency (i.e., lower POD) for cardiotoxicity as evaluated by all five phenotypes combined. Colors of the dots represent the corresponding PFAS subclass as indicated by the legend insert

Next, the effects of PFAS were assessed across donors (Fig. 3). Concentration-response analyses were performed for each of the 5 phenotypes across all 16 iPSC-derived cardiomyocyte lines. The box plots in Fig. 3A show the range of chemical-specific PODs across all donors. A decrease in cell beat rate was the phenotype affected by the largest number of PFAS, albeit the effects were mostly observed at concentrations above 10 µM. Interestingly, QT prolongation was the most sensitive endpoint as for many chemicals there was at least one donor that showed an effect at the lowest concentration tested. The PFAS were ordered in this figure based on the overall effect across all 5 phenotypes, as indicated by the ToxPi scores (Fig. 3B). No tested compound was active in all 5 phenotypes – among 56 tested PFAS, the most bioactive substance (fluorinated triethylene glycol monomethyl ether, C7F3ETOH) had a ToxPi score of 0.48, on a scale ranging from 0 to 1. For each compound, the corresponding PFAS subclass is indicated by the colored circles, the interspersed colors across the ToxPi rankings indicate that there were no clear trends based on the traditional PFAS structure-based subclasses.

**Fig. 4:**
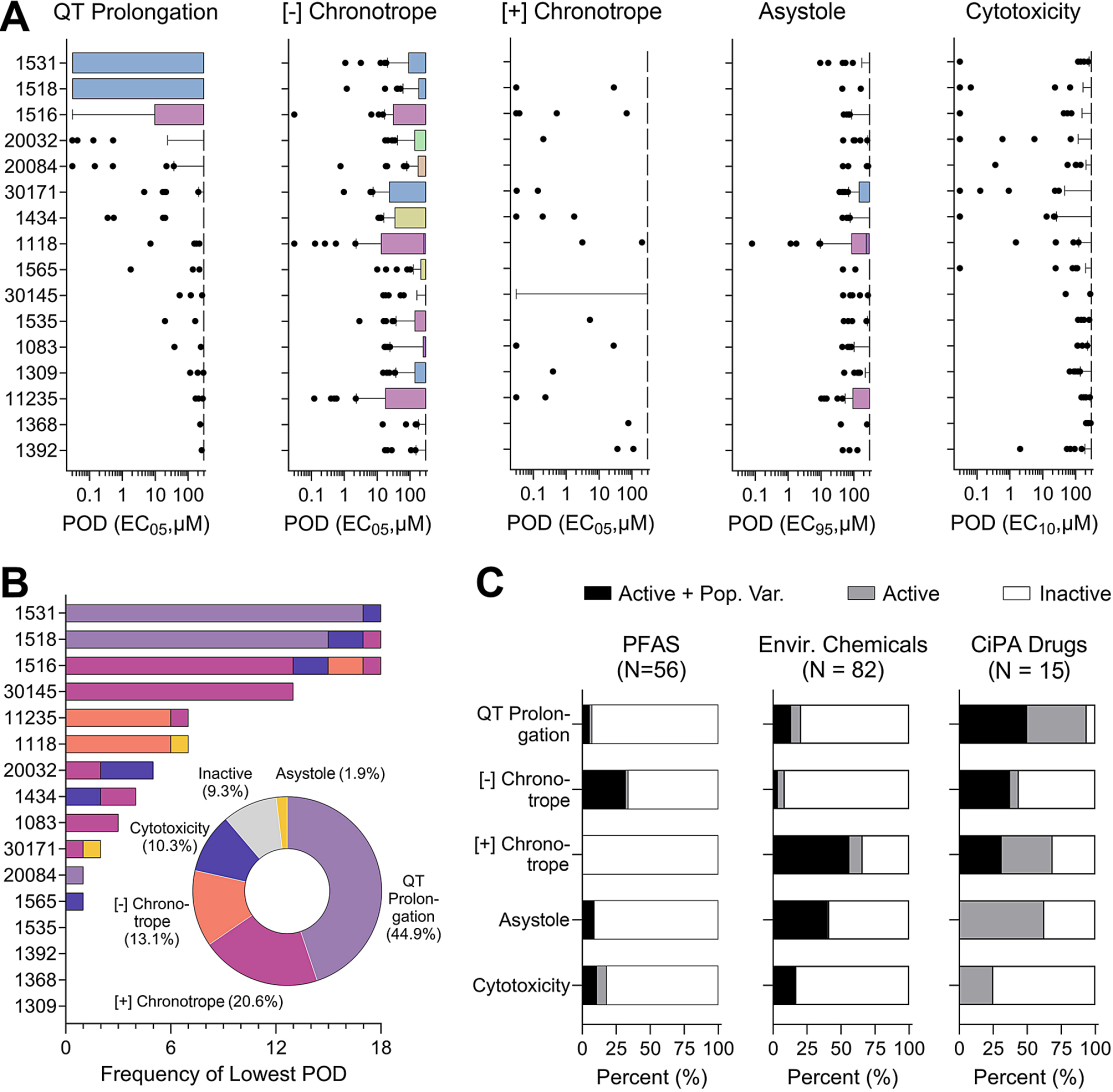
Donor-specific effects of PFAS. (**A**) For each phenotype and donor, box-and-whisker plots include data from all tested PFAS. Boxes are the interquartile range; the whiskers are the 10th to 90th %ile and the dots are chemical PODs outside of the 10th -90th range. Boxes are colored based on the subpopulation for the corresponding donor (blue – European, pink – African-American, yellow – mixed African-American/European (other), beige – Hispanic/Latino, and green – Asian). Donors are sorted by the median PODs. (**B**) Stacked bar graphs show the number of times each donor had the lowest POD for a given PFAS and the colors of the stacked bars illustrate from which phenotype the lowest POD was derived. The pie chart insert shows the frequency at which the lowest POD corresponded to each of the 5 phenotypes. Colors in the stacked bars and pie graph represent the phenotypes (light purple – QT prolongation, pink – positive chronotrope, orange – negative chronotrope, dark purple – cytotoxicity, yellow – asystole, and grey for inactive chemicals). (**C**) Cardiotoxicity hazard characterization. The stacked bars represent the percentage of compounds that were active and passed criteria for population variability analysis (Active + Pop. Var. – black), active but failed criteria for population variability (Active – grey), and inactive (Inactive – white). Data are shown for PFAS tested in this study (*n* = 56) as well as data for other environmental chemicals (*n* = 82), and CiPA drugs (*n* = 15)

As evident from the range of PODs across all donors and phenotypes, inter-individual variability in responses to PFAS was substantial; therefore, we examined chemical effects for each donor separately (Fig. 4A). Donors from different sub-populations were interspersed in these box plots as shown by colors. The intra-donor variability depended on the chemical and phenotype of interest and some PFAS showed effects. The widest range of responses among 56 tested PFAS was observed for donors 1531 (female) and 1518 (male), both subjects of European descent, which had PODs for QT prolongation spanning the entire testing range. As shown in Fig. 4B, we used donor-specific PODs to determine which donor and phenotype was the most susceptible using the lowest PODs for each of the 56 PFAS. Overall, three donors (Donor IDs: 1531, 1518, and 1516) were more sensitive in comparison to the other donors tested (Fig. 4B). Similarly, there were multiple donors (Donor IDs: 1535, 1392, 1368, and 1309) that were more resistant in responses to PFAS, such that the lowest POD was never derived from those donors. Upon examining the “sensitive” and “resistant” donor data (Fig. 4C), it is also evident that the lowest PODs were most often derived from the QT prolongation endpoint (44.9%). Furthermore, there were chemicals (9.3% of total) that were deemed as inactive across all donors (Fig. 4C).

**Fig. 5:**
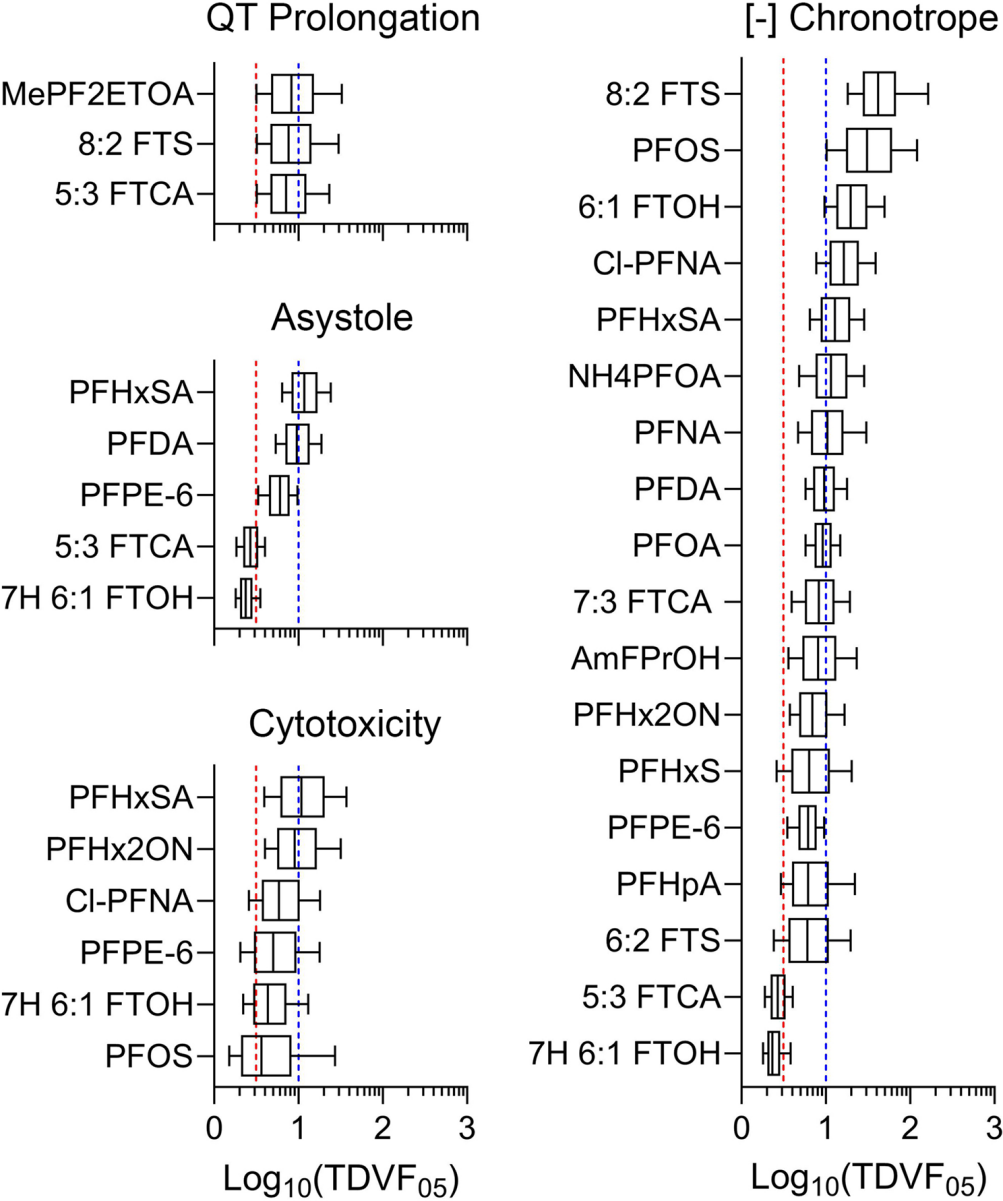
Inter-individual variability in PFAS-associated cardiotoxicity phenotypes. TDVF_05_ values were derived for PFAS that passed variability and activity criteria for each phenotype shown. Box-and-whisker plots show distributions of the TDVF_05_ values with boxes depicting the interquartile range and whiskers illustrating the 10th to 90th %iles. The vertical red dashed lines represent the default inter-individual toxicodynamic variability factor of 10^1/2^. The vertical blue dashed lines represent the default total inter-individual variability factor of 10. Chemical-specific TDVF_05_ data can be found in Table S7

Population-level cardiotoxicity hazard was evaluated by designating PFAS as active or inactive based on the population median POD (Fig. 4D) or more conservatively, using the 5th %ile population POD (Fig. 4E). For chemicals determined to be active for population variability (a total of 19 out 56 tested PFAS), the TDVF_05_ was calculated using the ratio of the POD (i.e., 5% change) for the median individual to the POD for the most sensitive 5th %ile individual. The distributions shown in Fig. 5 show PFAS and phenotypes for which TDVF_05_ could be calculated. Across all phenotypes, 2 chemicals had TDVF_05_ values that fell below the default value of 10^1/2^ and 10 chemicals had a TDVF_05_ between 10^1/2^ and 10. Close to 50% (7 of 19) of the chemicals had a TDVF_05_ above the total default uncertainty factor of 10. Previously, inter-individual variability in responses to chemicals has been evaluated in different in vitro, in vivo, and clinical studies, and in many cases both toxicodynamic and total intra-species variability exceeded 10-fold [45, 61–63]. Similar results have been observed in human iPSC-derived cardiomyocytes exposed to a wide range of environmental chemicals and drugs [59, 60].

**Fig. 6:**
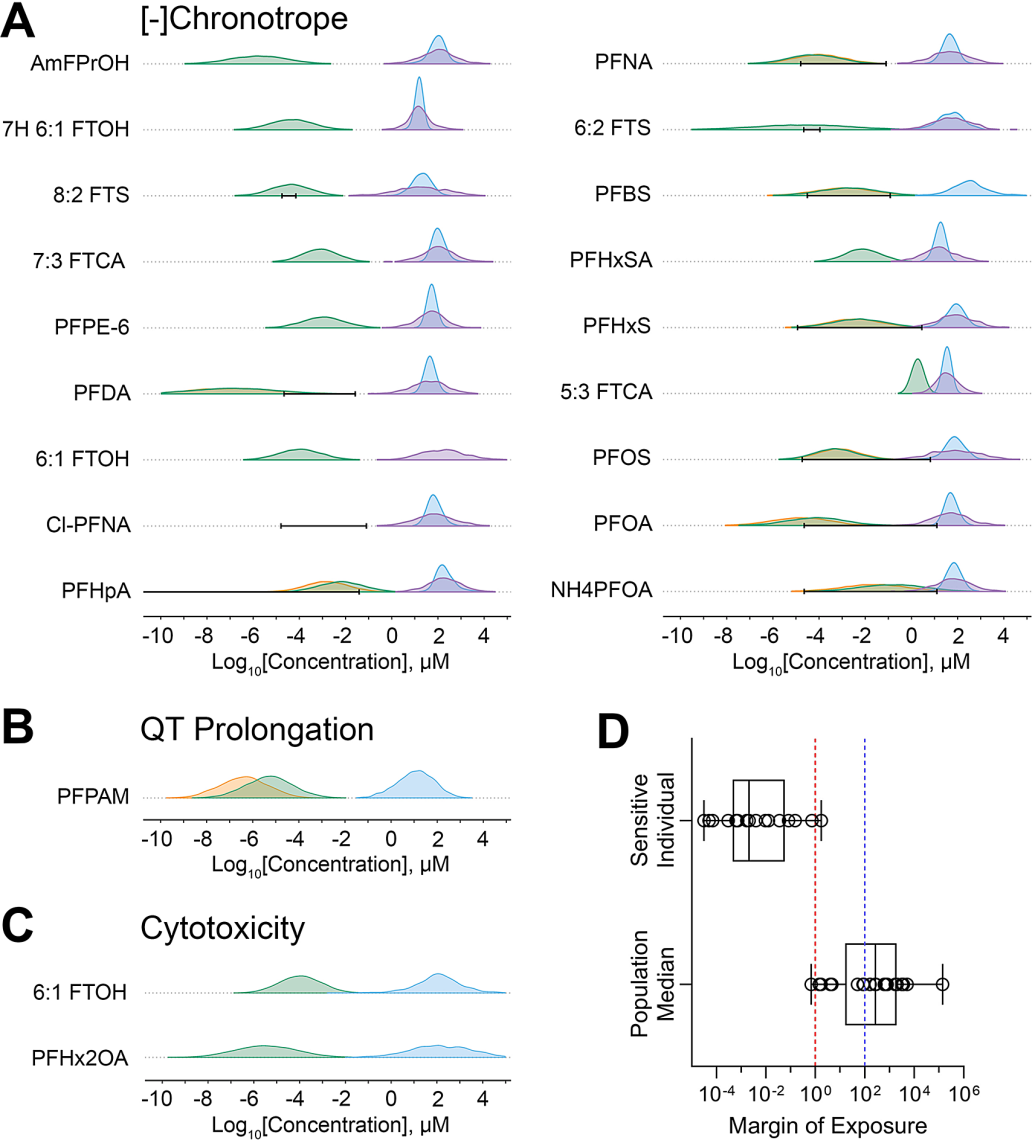
Margin of exposure (MOE) estimates for tested PFAS. Chemical-specific MOEs were derived from the most sensitive iPSC-derived cardiomyocyte POD (from all donors and phenotypes) and chemical-specific exposure data (using levels measured in humans and supplementing it with predicted exposure data when needed). (**A-C**) Density plots illustrating distributions for three phenotypes with at least one PFAS for which an MOE could be derived. Distributions for exposure (green histograms – predicted exposures using plasma protein binding assumptions, orange histograms – predicted exposures using the HTTK assumptions, black bars – the range (5th to 95th %ile) of reported blood levels) and bioactivity (blue histograms –population median PODs, purple – random individual PODs) are shown. (**D**) The distribution of the MOEs for both the sensitive individual and the population median. The ratio between exposure and bioactivity was calculated as the MOE (on a log scale). Box plots represent the interquartile range and whiskers showing the range from minimum to maximum, and individual dots show values for specific chemicals. The vertical dashed lines are drawn at 1 (no margin of safety) and 100 (the value considered to be “protective” in many human health risk assessments). Chemical-specific MOE estimates can be found in Table S9 and Fig. S3

To put the bioactivity data in the context of risk characterization, we compared the in vitro PODs to measured and/or predicted PFAS exposure levels to derive chemical-specific margins of exposure (MOE), as shown in Fig. 6. MOEs could be derived for 20 PFAS, these substances had at least one active phenotype and information on human exposure. Bioactivity for most of these PFAS showed little overlap with exposure data and/or estimates. Most PFAS (~ 60%) had MOEs above 100, 7 had MOEs between 1 and 100, and 1 (ammonium perfluorooctanoate (NH4PFOA)) had an MOE below 1 indicating potential human health concern at current population median exposure levels.

**Fig. 7:**
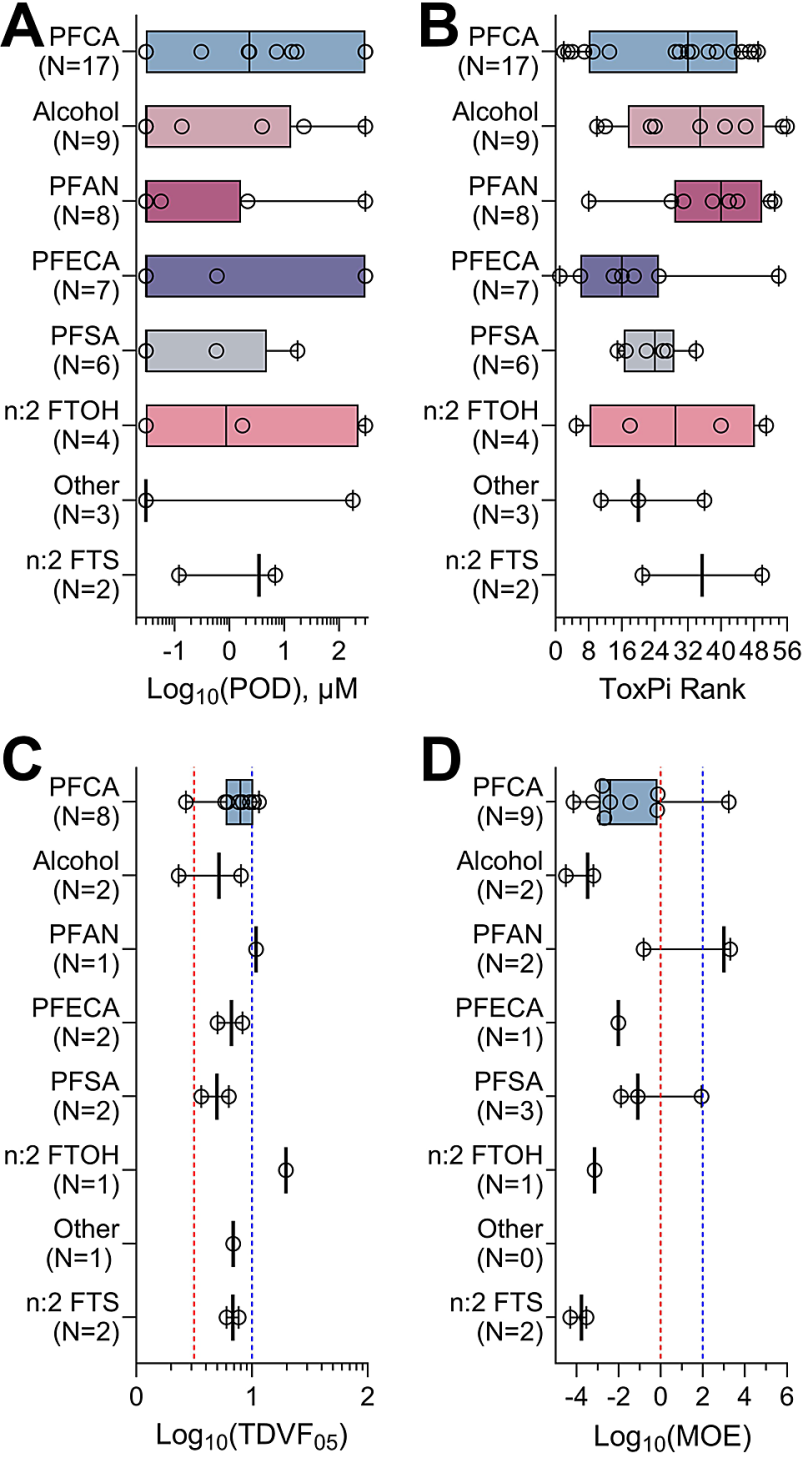
PFAS subclass effects on iPSC-derived cardiomyocytes. Data are presented separately for each PFAS subclass (see Table 1). Box plots represent the interquartile range and whiskers showing the range from minimum to maximum, and individual dots show values for specific chemicals. Colors represent the various subclasses and are arranged by subclass with most chemicals to least chemicals. (**A**) The lowest PODs across all phenotypes for each chemical in a subclass. (**B**) Overall ToxPi rankings aggregated by subclass. All tested PFAS were included in panels A and B. (**C**) Toxicodynamic Variability Factors 5th %ile organized by subclass. Only chemicals that were active and passed the criteria for population variability were included. The vertical red dashed line represents the default inter-individual toxicodynamic variability factor of 10^1/2^. The vertical blue dashed line represents the default total inter-individual variability factor of 10. (**D**) Chemical-specific MOE estimates arranged by subclass. The vertical dashed lines are drawn at 1 (red, no margin of safety) and 100 (blue, the value considered to be “protective” in many human health risk assessments)

To examine structure-activity relationships among tested PFAS, we utilized previously established structure-based subclasses (Table 2) to determine if there are subclass-specific similarities in PODs (Fig. 7A), ToxPi rankings (Fig. 7B), TDVF_05_ values (Fig. 7C), and MOEs (Fig. 7D). In each subclass, there were few discernable patterns in potency, activity, population variability, or risk. These results are consistent with previous studies that demonstrated that structure-based subclasses are not a feasible way to group PFAS [22, 23]. Specifically, for each of the four indicators, there is not a subclass that is significantly different in comparison to the others with the exception of alcohols and PFCA in the MOE comparison.

In addition, we evaluated specific chemical structural descriptors as to their potential relationship to bioactivity (Table 3). Upon calculating all pair-wise correlations (Spearman rank) among all in vitro phenotypes and Saagar descriptors and adjusting for multiple comparisons, a large number of significant negative correlations (presence of the feature in a molecule indicated greater effect, i.e. lower POD) were observed. Descriptors were grouped by type such as atoms/atom pairs present, bioavailability, functional groups, and topology and the phenotype, number of donors, and number of PFAS for which the associations were significant are included. The specific chemicals and the highlighted features are shown in Fig. S7. Many of the molecular features that were significant for negative chronotrope were found to be significant for most donors and all (or the majority of) tested PFAS and were indicators of the overall size of the molecule and the carbon chain length. Interestingly, the positive chronotrope, asystole and QT prolongation associations were donor- and chemical-specific, indicating the potential molecular features that can be indicative of substructure-specific effects and inter-individual variability.

**Table 3:** Chemical structure descriptors that were significantly negatively correlated with iPSC-derived cardiomyocyte bioactivity phenotypes after exposure to PFAS

Descriptor ID^a^	Phenotype(s)	*N* of Donors^b^	*N* of Subst.^c^	Descriptor Meaning
Atoms				
SGR10004	[-] Chronotrope	11	56	Fluorine (≥ 5 fluorine atoms present)
	Asystole	4		
SGR10013	[-] Chronotrope	7	56	Any carbon
	Asystole	5		
SGR10022	[-] Chronotrope	8	56	sp3 Carbon
	Asystole	5		
SGR10029	[-] Chronotrope	10	56	Any non-carbon
	Asystole	4		
	Cytotoxicity	2		
SGR10043	[-] Chronotrope	12	56	Tetrasubstituted sp3 carbon
	Asystole	4		
	Cytotoxicity	1		
SGR10068^d^	[+] Chronotrope	1	9	Oxygen with two heavy atom substitutions
SGR10032^d^	[+] Chronotrope	2	3	Methyl-group
Atom Pairs				
SGR10521	[-] Chronotrope	11	54	Two halogens, 3 bonds away (≥ 4 occurrences)
	Asystole	3		
SGR10553	[-] Chronotrope	12	53	Two halogens, 4 bonds away (≥ 4 occurrences)
	Asystole	3		
SGR10583	[-] Chronotrope	10	44	Two halogens, 5 bonds away (≥ 4 occurrences)
	Asystole	4		
	Cytotoxicity	2		
SGR10786^d^	[+] Chronotrope	5	8	OH-mediated intramolecular hydrogen-bonds
SGR10112^d^	[+] Chronotrope	4	7	Two oxygens, 3 bonds away
SGR10199^d^	[+] Chronotrope	2	3	Two oxygens, 5 bonds away
Bioavailability				
SGR10261, SGR10780	[-] Chronotrope	13	56	F-C-F group (≥ 3 occurrences)
	Asystole	4		
SGR10805	[-] Chronotrope	6	51	X-C-X moiety (X = O, N, or F)
	Asystole	3		
SGR10633, SGR10708^d^	[+] Chronotrope	1	17	Primary alkyl bonded to O, N,S, or P atoms
SGR10275, SGR10295^d^	[+] Chronotrope	1	12	Primary alcohol
SGR10169^d^	[+] Chronotrope	1	8	Dialkyl ether
SGR10795^d^	Cytotoxicity	1	4	Primary carbon bonded to 2 fluorines
	Asystole	1		
SGR10290^d^	[+] Chronotrope	5	3	Ethylene glycols and their mono-ethers
SGR10418, SGR10493, SGR10684, SGR10736 ^d^	[+] Chronotrope	2	3	Secondary alcohol
SGR10354^d^	[+] Chronotrope	5	2	Isopropanol moiety
SGR10668^d^	[+] Chronotrope	3	2	Methoxy or methylamino group
Functional Groups				
SGR10009 SGR10205	[-] Chronotrope	11	56	Fluorine bonded to aliphatic carbon (≥ 5 occurrences)
	Asystole	4		
SGR10308	[-] Chronotrope	10	56	Potential hydrogen-bond acceptors (≥ 6 occurrences)
	Asystole	4		
	Cytotoxicity	2		
SGR10829	[+] Chronotrope	1	55	Potential hydrogen-bond donors
SGR10428	[-] Chronotrope	11	54	1,2-Dihalogenated ethyl (≥ 4 occurrences)
	Asystole	3		
SGR10092	[-] Chronotrope	10	53	1,1,2,2-Tetrafluorinated ethyl
	Asystole	3		
SGR10797	[-] Chronotrope	6	50	-CF_2_- or -O-CF- moiety
	Asystole	3		
SGR10057	[-] Chronotrope	9	47	1,2,3-Trifluorinated propyl (≥ 6 occurrences)
	Asystole	3		
SGR10072, SGR10761^d^	[+] Chronotrope	1	14	Hydroxyl attached to sp3 carbon
SGR10203^d^	[+] Chronotrope	1	8	Ether
SGR10153^d^	[+] Chronotrope	1	7	Sulfur bonded to 3 heavy atoms
SGR10109^d^	[+] Chronotrope	2	3	CH-OH moiety
SGR10343^d^	[-] Chronotrope	1	3	Any primary amine
SGR10289^d^	[+] Chronotrope	5	2	Ethylene glycol diol
SGR10587, SGR10099, SGR10703^d^	[+] Chronotrope	3	2	Sulfonamide
	Asystole	2		
Topology				
SGR10704^d^	Cytotoxicity	11	2	Polyether motifs as in PEGs
	[+] Chronotrope	2		
SGR10749^d^	[+] Chronotrope	5	2	1,2-disubstituted ethanol

^a^ Saagar descriptors that were significantly correlated with iPSC-derived cardiomyocyte phenotypes can be found in Table S18. Correlation coefficients and corresponding q-values for the donors and phenotypes with significant (q < 0.1) associations can be found in Table S19

^b^ Number of donors with significant (q < 0.1) associations from a total of 16 tested

^c^ Number of PFAS with significant (q < 0.1) associations from a total of 56 tested

^d^ Saagar descriptors with corresponding structural features for each PFAS, highlighting the region that describes the particular Saagar descriptor can be found in Fig. S7

Previous studies have revealed trends between descriptors such as carbon chain length and molecular weight, and in vitro bioactivity [18–21]. Therefore, in addition to exploring the associations between bioactivity and Saagar descriptors, we also tested the correlation between bioactivity and physicochemical descriptors extracted from the OPERA database (Tables S16-S17). Upon accounting for multiple testing, we found that both donor-specific and all donor PODs were significantly negatively correlated (Spearman rank) with molecular weight, carbon chain length, Henry’s Law Constant, melting point, and octanol-water partition coefficient. Additional descriptors (boiling point, octanol-water distribution coefficient, vapor pressure, and water solubility) were significantly negatively correlated with responses in the individual donors and some phenotypes.

Because Saagar descriptors and physicochemical properties showed associations with bioactivity, we next tested whether Saagar descriptors can be used to infer in vitro data. This question is relevant because in vitro testing of additional PFAS will be time consuming and if a predictive model can be developed, considerable time and resource savings can be achieved by prioritizing future analyses. Using a regression model with rigorous cross-validation, we found that some bioactivity data could be predicted (Tables S12-S14). For example, the minimum POD across all donors and for some individual donors and phenotypes could be predicted from the Saagar features alone (*r* = 0.53–0.66 and p_*adj*_=0.001–0.047 for [-] chronotrope; *r* = 0.56–0.63 and p_*adj*_ = 0.002–0.021 for asystole). For both negative chronotrope and asystole phenotypes, the highest cross-validated prediction was achieved for the minimum POD across all donors. When the same analyses were performed using the OPERA-derived physicochemical properties, the only significant prediction was achieved for the minimum POD across all donors for negative chronotrope and asystole phenotypes (*r* = 0.54 and p_*adj*_=0.028 for [-] chronotrope; *r* = 0.56 and p_*adj*_=0.019 for asystole).

## Discussion

This study is first to evaluate the extent of inter-individual variability in responses of human cardiomyocytes to many PFAS. Previous reports suggested that PFAS have effects on human iPSC-derived cardiomyocytes [22, 23]; this study independently corroborated those findings made in cells from one donor. In addition, our results demonstrate that such effects are occurring in cells from different individuals, and that inter-individual variability in the effects can be quantified and used in the context of risk characterization. Our observation of negative chronotrope as a main effect of PFAS corroborates our two previous PFAS studies [22, 23], experiments that performed using commercially-available human iPSC-derived cardiomyocytes from the “standard” donor (Donor ID: 1434) instead of a diverse population of donors. Our findings offer insight into several areas of importance, contributing not only to the overall body of knowledge in cardiovascular toxicology, but also for decision-making regarding a class of compounds that is of great concern to a number of regulators worldwide [64].

Cardiovascular disease is an important public health burden and several environmental risk factors, such as air pollution, smoking, and exposure to heavy metals are well-established contributors [25]. Several mechanistic and laboratory animal studies suggest that PFAS could also contribute to the global burden of cardiovascular disease [24]. However, there is a limited number of studies that examine cardiotoxicity of PFAS and the mechanisms by which these effects may be induced as compared to evidence on other chemical classes. In fact, a recent systematic mapping review demonstrated that most environmental exposure and cardiotoxicity studies are focused on air pollution, heavy metals and pesticides [65]. Several epidemiological studies have evaluated cardiovascular outcomes (e.g., ischemic heart disease, hypertension, stroke, cardiovascular disease, myocardial infarction, and pregnancy-induced hypertension) in relation to the body burden of, or exposure to, PFAS [66], however, only a few found significant associations. Studies that reported strong associations showed positive relationships between PFAS exposure and risk of stroke, hypertension, and atherosclerosis [66, 67]. *In vivo* PFAS studies, in both rodents and non-human primates, have shown no histological alterations in the heart [66]. Recent systematic evidence maps of PFAS evaluated both scientific publications and regulatory submission documents have identified several additional potential adverse effects of some PFAS [12, 68]. These effects consisted of incidental findings of decreases in absolute and/or relative heart weights in studies of rodents, most were from 28- or 90-day inhalation or oral exposures. Only one study in beagle dogs showed that inhalation exposures to trifluoroiodomethane or 1,1,2,2,3,3,3-heptafluoro-1-iodopropane (compounds not tested in our study) resulted in cardiac sensitization to adrenaline [69]. Cardiac sensitization effects have been associated with exposures to a number of other halogenated molecules, including halo- and fluoro-carbons [70]; however, no such *in vivo* studies have been performed with higher fluorinated PFAS.

Recent epidemiological studies that focused on cardiovascular disease and PFAS have measured vascular thickness as a sign of atherosclerosis [71], or used echocardiography to study morphology and function of the myocardium [72]. Still, the objective measures of the heart rhythm (e.g., pulse and/or electrocardiography) are not frequently included in studies of PFAS in humans, and almost never in laboratory animal studies of non-pharmaceuticals. Therefore, in vitro studies in human iPSC-derived cardiomyocytes provide important information about potential cardiotoxic hazards for both pharmaceuticals and environmental chemicals. The translational value of this in vitro model has been demonstrated in terms of its ability to replicate genetic disorders affecting heart rhythm [28], and clinical effects of various cardio-active and -toxic drugs [26, 43, 73]. More importantly, iPSC-derived cardiomyocytes are one of the very few human in vitro models available to study inter-individual variability in responses to drugs and chemicals [29, 37]. The iPSC-derived cardiomyocytes can be utilized as a model for personalized chemotherapy [30], or an experimental tool to quantify the extent of variability in drug effects in a population [26, 59] which also has been demonstrated. Our observation that negative chronotrope was the most frequent functional effect on iPSC-derived cardiomyocytes across all tested PFAS stands out in comparison to our previous findings for drugs, and different non-PFAS environmental and industrial chemicals. Specifically, QT prolongation was the most pronounced effect of comprehensive in vitro proarrhythmia assay (CiPA) drugs, while positive chronotrope was the most frequently impacted functional phenotype across ~ 1000 diverse environmental chemicals [32, 44]. This observation raises two questions – why there is such a difference in effects, and how our findings of a decreased beating frequency may relate to the epidemiological evidence of PFAS and cardiovascular disease, and overall human hazard and risk?

Mechanistically, the unique physicochemical properties of PFAS, specifically their surfactant-related tendency to be sorbed or concentrated on non-aqueous phase liquid-water interfaces [74], provide one hypothesis for explaining the differences observed. Specifically, the plasma membrane of all cells contains cholesterol-enriched lipid rafts which are critical to deliver proteins to the membrane and for sequestering proteins in close physical proximity to control their functional interactions [75]. Indeed, cardiac ion channels are known to be localized into lipid rafts, which are critical for their function and trafficking at the plasma membrane [76]. In addition, calcium and other ion channels in cardiomyocytes have been shown to be impacted by changes in membrane fluidity by various surface-active compounds [77]. PFAS have been shown to concentrate in cell membranes; for example, accumulation of PFOA in platelet membranes was shown to result in a more fluid state which can alter cell permeability and ion channel structure and function [78]. The membrane-disrupting effects of these substances have been observed in several cell types [79, 80]. Thus, exposure to PFAS and cardiovascular risk *in vivo* may involve endothelial dysfunction and activation of circulating platelets [67]. Taken together, these considerations outline the potential reasons for the divergence in the effects of PFAS versus other chemicals and drugs on human iPSC-derived cardiomyocytes; however, additional studies are needed to provide mechanistic support for this hypothesis.

With respect to the relationship between our findings in human cardiomyocytes and clinical outcomes, the most frequent phenotypic effect we observed after exposure to PFAS was a decrease in the beating rate, a phenotype analogous to the clinical syndrome of bradycardia. Heart rate is a well-established predictor of major cardiovascular disease types, such as atherosclerosis, in both the general population and patients with various cardiovascular diseases [81]. However, an increase in heart rate is typically of greater clinical concern, because it is thought to lead to endothelial dysfunction and is associated with increased progression of coronary atherosclerosis in animal models and patients. To the contrary, heart rate reduction has been shown to slow progression of atherosclerosis in animal models. Several epidemiological studies examined the relationship between bradycardia and cardiovascular disease risk and yielded conflicting results. A study of 6,733 older adults from a “multi-ethnic” cohort in the United States showed that bradycardia was not associated with an increased incidence of cardiovascular disease, but it was associated with mortality among participants who were on drugs that may slow heart rate [82]. A similarly-sized study in Japan found that both bradycardia and tachycardia are independent risk factors for future cardiovascular events in healthy men [83]. Given these previous reports, we cannot conclude that our findings of a slower beat rate after exposure to PFAS in vitro are without potential clinical relevance. The likely human health hazard concern from our data as a whole also comes from the finding that QT prolongation and positive chronotrope effects were two functional phenotypes that were most variable between individuals. The clinical importance of both prolongation of the QT interval that may provoke Torsades de Pointes, and other arrhythmias that involve tachycardia is well-established [84]. However, while the mechanisms of some drug-associated arrhythmias are well known, in the case of environmental chemicals the mechanisms remain poorly understood and require further studies.

In terms of the observed range in the functional effects of PFAS on human cardiomyocytes in vitro, it is noteworthy that our analysis of the relationships between chemical structural features of PFAS and their effects across different individuals also offers additional potential insights. We found that PFAS size (e.g., fluorine and carbon content) was significantly associated with greater bioactivity on negative chronotrope. This finding corroborated our previous analysis in a single cardiomyocyte donor [22], as well as studies in other cell types [18, 19, 85]. Interestingly, more specific structural features that were significantly negatively correlated with positive chronotrope effects were observed in only a subset of the donors. This indicates that individual susceptibility to this type of arrhythmia may be related to specific head groups in certain PFAS, such as a sulfonamide, primary amine, or polyether functional groups or other molecular topology features. Collectively, these observations provide further information for the current debate, as to whether or not structural features can be used to group PFAS in order to offer a pragmatic approach to address the daunting task of evaluating PFAS, for which an overwhelming majority have no data to inform traditional hazard and risk evaluations [86]. One commonly proposed testing strategy for PFAS involves prioritization based on chemical structure [87, 88]; however, little experimental evidence exists from cell-based assays suggests that specific structural features, beyond molecular weight and carbon chain length, can be used as predictors. On the contrary, recent studies show that many of the tested PFAS elicit cell-based effects and gene expression signatures [22, 23] and that high throughput testing of the individual chemicals and their mixtures may be the most protective approach. In this respect, deriving information on both potential hazard and inter-individual variability, the approach that was taken in our study, may be a sensible strategy for prioritization and risk characterization.

This study has several limitations that need to be acknowledged. First, while iPSC-derived cardiomyocytes have become a robust, reproducible and widely-used model to test for potential structural (e.g., cell viability) and functional (e.g. arrythmia-related) liabilities in drug development [28, 89, 90], the phenotypes that can be assessed using this model cover only some of the modalities of cardiovascular disease. In addition, all phenotypes evaluated in this study were weighted equally to maintain an unbiased approach; however, it should be noted that the phenotypes evaluated herein may be of different clinical importance and that alternative analyses with increased emphasis on certain endpoints may be needed. Studies in other in vitro models that probe effects on cardiomyocyte contractility [91, 92] or (micro)vasculature [93, 94] are needed but may require triaging of some PFAS for such testing because of the low-throughput. Second, the library of PFAS tested and the number of iPSC-derived cardiomyocyte donors available were both limited. The former is a challenge as the availability of high-purity PFAS is limited and the throughput of the experiments in cardiomyocytes is also an important practical barrier. Further, real-life exposures are to complex mixtures; therefore, our data on the individual PFAS may need to be interpreted with caution and additional studies of mixtures are needed to account for potential additive or multiplicative effects. Secondly, the number of cardiomyocyte donors that are robust and reproducible is also an area where future solutions are needed. Commercial offerings of iPSC-derived cardiomyocytes from different donors are limited and greater appreciation of the value of population-based human in vitro models is still evolving [37]. Third, confident risk characterization depends on robust empirical human exposure information and on the confidence in extrapolating data from cell-based experiments to human exposures. While we have derived MOE estimates in this study, we could do so only on some of the PFAS tested because of the lack of human biomonitoring data and/or in vitro bioavailability data. Moreover, exposure predictions in the absence of biomonitoring data were only predicted for the population median, with many orders of magnitude of uncertainty. Indeed, the overall uncertainty in exposure is likely to be even greater, since these predictions do not include highly exposed subpopulations. Considerable efforts are underway to improve human data on blood levels of PFAS [95, 96], to better characterize exposure patterns [97, 98], and to provide toxicokinetic data to enable in vitro-to-in vivo extrapolations [53, 54].

In summary, this study demonstrates a feasible approach to characterize and quantify cardiotoxicity and inter-individual variability in responses to PFAS. Furthermore, the data can be used to rank PFAS based on hazard potential and potency or based on risk through derivation of MOE estimates. Although, we did not find PFAS groupable by subclass structure, other molecular descriptors were correlated with the observed bioactivity, suggesting the potential for descriptor-based prioritization. These data and methodologies provide invaluable information for performing cardiotoxicity risk characterization of the thousands of PFAS to which people are exposed, and ultimately for informing decision-making for this critical public health concern.

### Electronic supplementary material

Below is the link to the electronic supplementary material.

Supplementary Material 1

Supplementary Material 2

Supplementary Material 3

Supplementary Material 4

Supplementary Material 5

Supplementary Material 6

Supplementary Material 7

Supplementary Material 8

Supplementary Material 9

Supplementary Material 10

Supplementary Material 11

Supplementary Material 12

Supplementary Material 13

Supplementary Material 14

Supplementary Material 15

Supplementary Material 16

Supplementary Material 17

Supplementary Material 18

Supplementary Material 19

Supplementary Material 20

Supplementary Material 21

Supplementary Material 22

Supplementary Material 23

Supplementary Material 24

Supplementary Material 25

Supplementary Material 26

Supplementary Material 27

Supplementary Material 28

Supplementary Material 29

Supplementary Material 30

Supplementary Material 31

Supplementary Material 32

Supplementary Material 33

Supplementary Material 34

Supplementary Material 35

Supplementary Material 36

Supplementary Material 37

Supplementary Material 38

Supplementary Material 39

Supplementary Material 40

Supplementary Material 41

Supplementary Material 42

Supplementary Material 43

Supplementary Material 44

Supplementary Material 45

Supplementary Material 46

Supplementary Material 47

Supplementary Material 48

Supplementary Material 49

## Data Availability

Data is provided within the manuscript or supplementary information files.

## Abbreviations

PFAS: Per–and polyfluoroalkyl substances
PFOA: Perfluorooctanoic acid
PFOS: Perfluorooctane sulfonic acid
PCA: Principal components analysis
OECD: Organisation for Economic Co–operation and Development
US EPA: United States Environmental Protection Agency
iPSC: Induced pluripotent stem cells
TAB: Tetra–octyl ammonium bromide
DMSO: Dimethyl sulfoxide
TFPrOA: 3,3–bis(trifluoromethyl)–2–propenoic acid
PFPE: 6–perfluoro–3,6,9–trioxatridecanoic acid
PFPOH: 1 H,1 H,5 H–perfluoropentanol
7H 6:1FTOH: Dodecafluoroheptanol
PFHxSA: Perfluorohexanesulfonamide
POD: Point(s) of departure
EC_05,10,95_: Effect concentration at which there is a 5, 10, or 95% effect observed compared to controls
ToxPi: Toxicological Priority Index
TDVF_05_: Toxicodynamic variability at 5%
MOE: Margin(s) of exposure
MOS: Margin(s) of safety
IVIVE: In vitro to in vivo extrapolation
CompTox Dashboard: Computational Toxicology Chemistry Dashboard
C_ss_: Steady–state plasma concentration
httk: High–throughput toxicokinetics
OPERA: Open (Quantitative) Structure–activity/property Relationship App
NIH: National Institute of Health
ICE: Integrated chemical environment
CV: Coefficient of variability
C7F3ETOH: Fluorinated triethylene glycol monomethyl ether
CiPA: Comprehensive in vitro proarrhythmia assays
