# Supplementary figures and images for: Characterizing PFAS hazards and risks: a human population-based in vitro cardiotoxicity assessment strategy
Source: Hum Genomics. 2024 Sep 2;18:92. doi: 10.1186/s40246-024-00665-x (PMC11368000; doi:10.1186/s40246-024-00665-x)

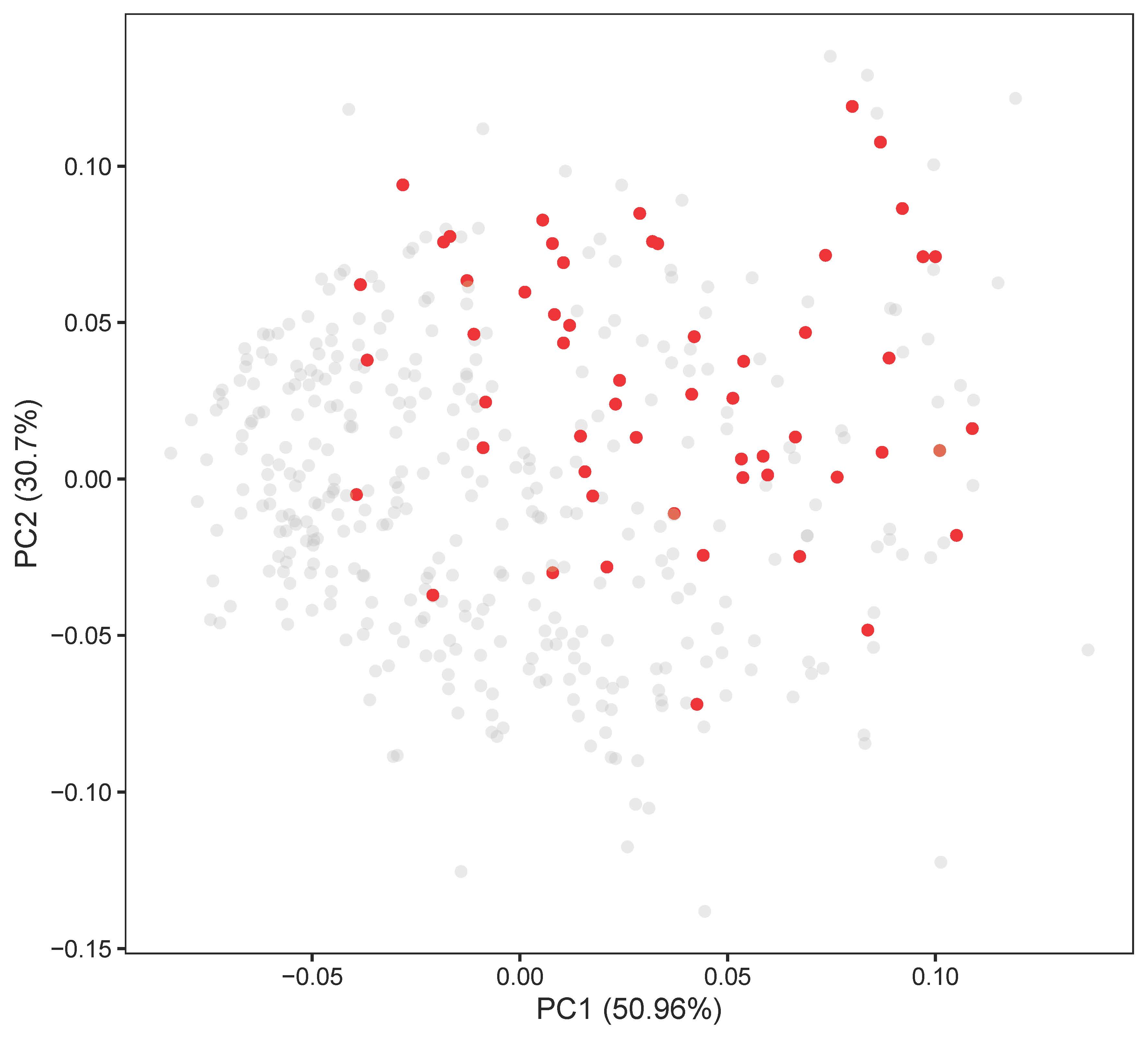

Supplement: Supplementary file 1 — Supplementary Material 1 [file 40246_2024_665_MOESM1_ESM.jpg]

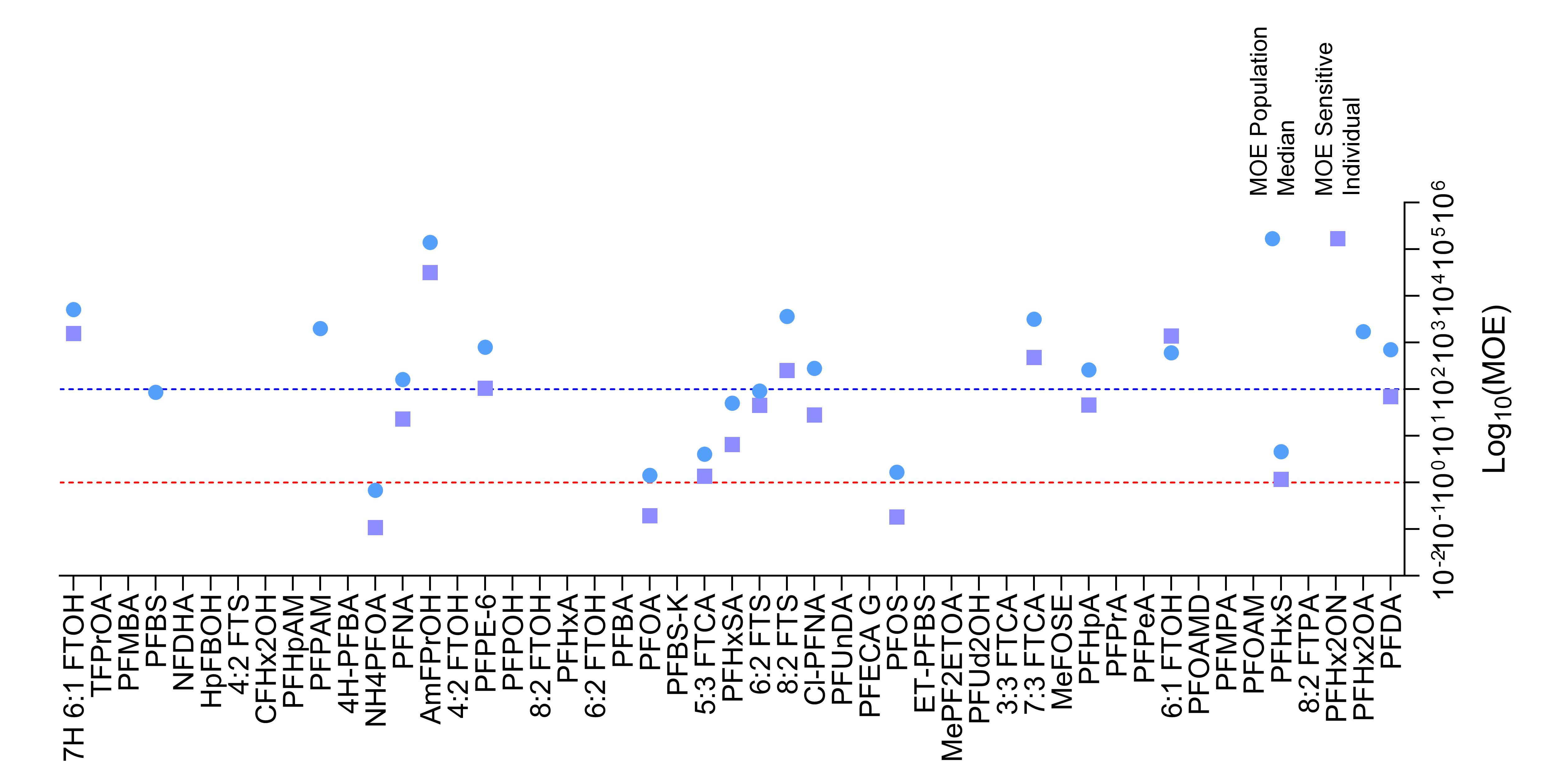

Supplement: Supplementary file 9 — Supplementary Material 9 [file 40246_2024_665_MOESM9_ESM.jpg]

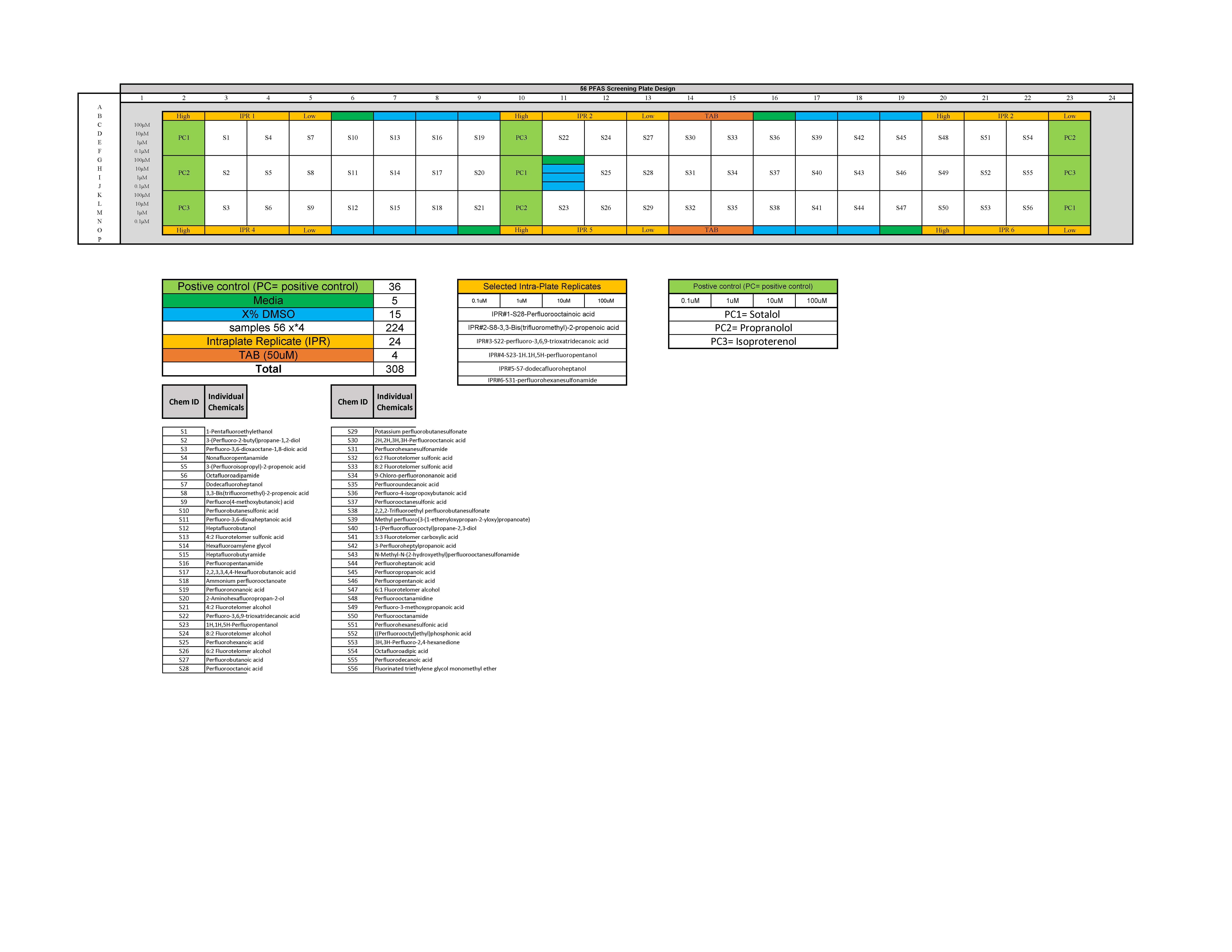

Supplement: Supplementary file 11 — Supplementary Material 11 [file 40246_2024_665_MOESM11_ESM.jpg]
