# Supplementary material for: Characterizing PFAS hazards and risks: a human population-based in vitro cardiotoxicity assessment strategy
Source: Hum Genomics. 2024 Sep 2;18:92. doi: 10.1186/s40246-024-00665-x (PMC11368000; doi:10.1186/s40246-024-00665-x)
Supplement: Supplementary file 3 — Supplementary Material 3 [file 40246_2024_665_MOESM3_ESM.pdf]

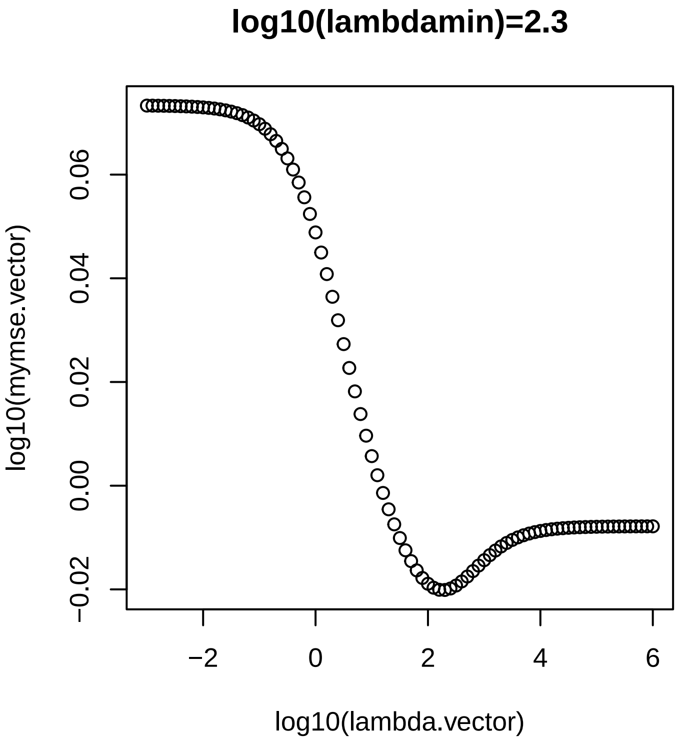
log10(lambdamin)=2.3
log10(mymse.vector)
0.06
0.04
0.02
0.00
−0.02
−2
0
2
4
6
log10(lambda.vector)

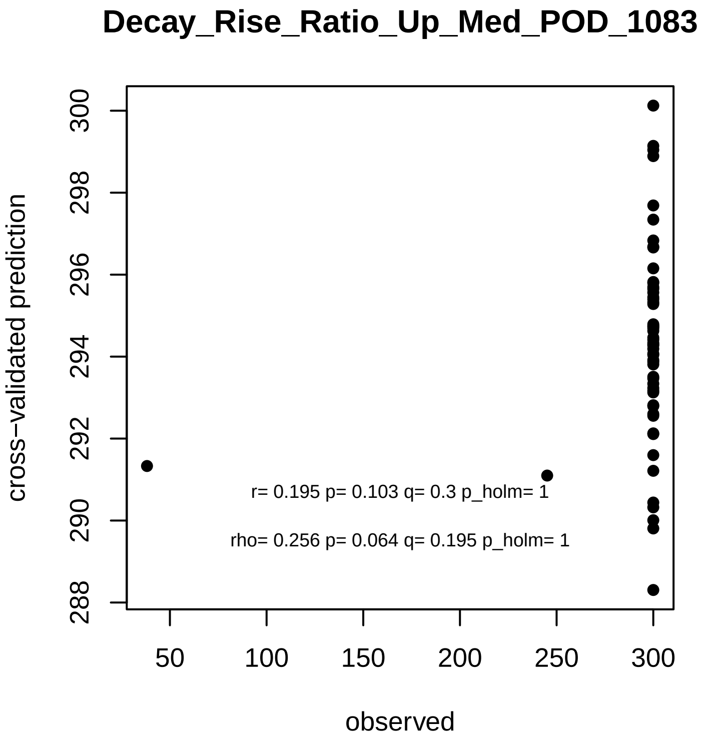
Decay_Rise_Ratio_Up_Med_POD_1083
r= 0.195 p= 0.103 q= 0.3 p_holm= 1
rho= 0.256 p= 0.064 q= 0.195 p_holm= 1
cross−validated prediction
observed

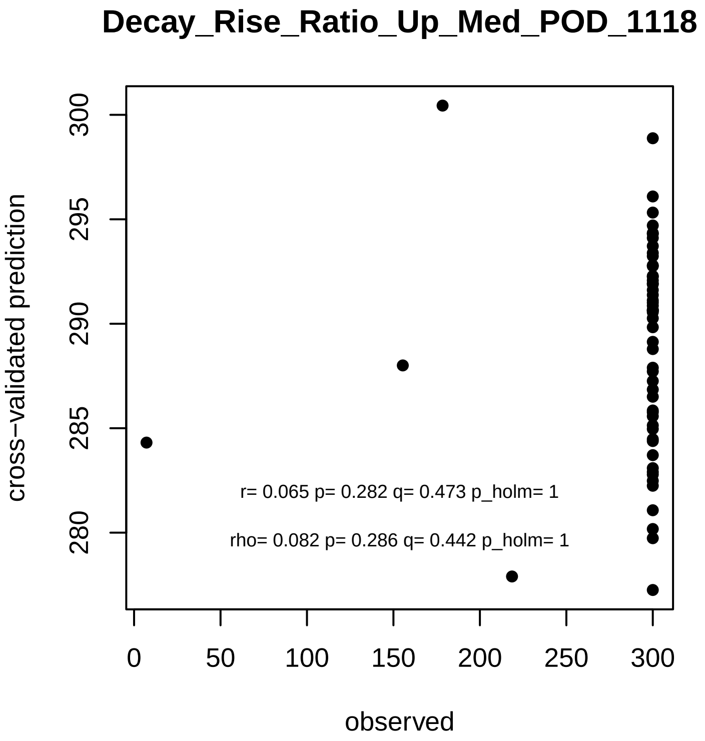
Decay_Rise_Ratio_Up_Med_POD_1118
r= 0.065 p= 0.282 q= 0.473 p_holm= 1
rho= 0.082 p= 0.286 q= 0.442 p_holm= 1
cross−validated prediction
observed

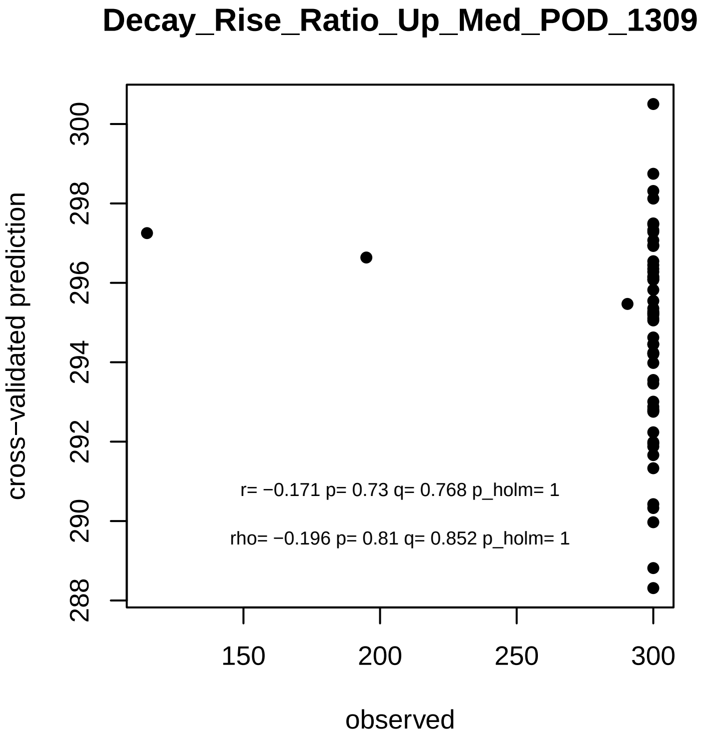
Decay_Rise_Ratio_Up_Med_POD_1309
r= −0.171 p= 0.73 q= 0.768 p_holm= 1
rho= −0.196 p= 0.81 q= 0.852 p_holm= 1
cross−validated prediction
observed

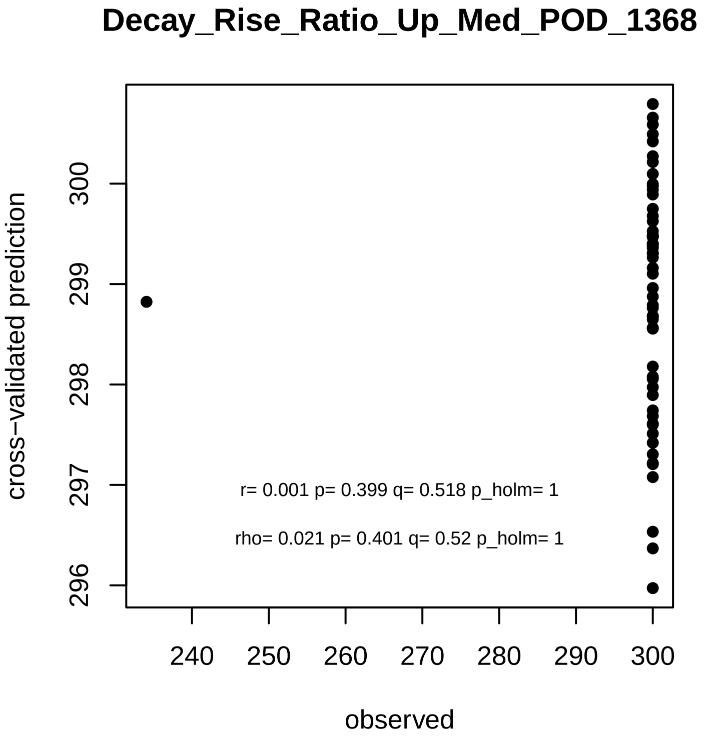
Decay_Rise_Ratio_Up_Med_POD_1368
r= 0.001 p= 0.399 q= 0.518 p_holm= 1
rho= 0.021 p= 0.401 q= 0.52 p_holm= 1
cross−validated prediction
observed

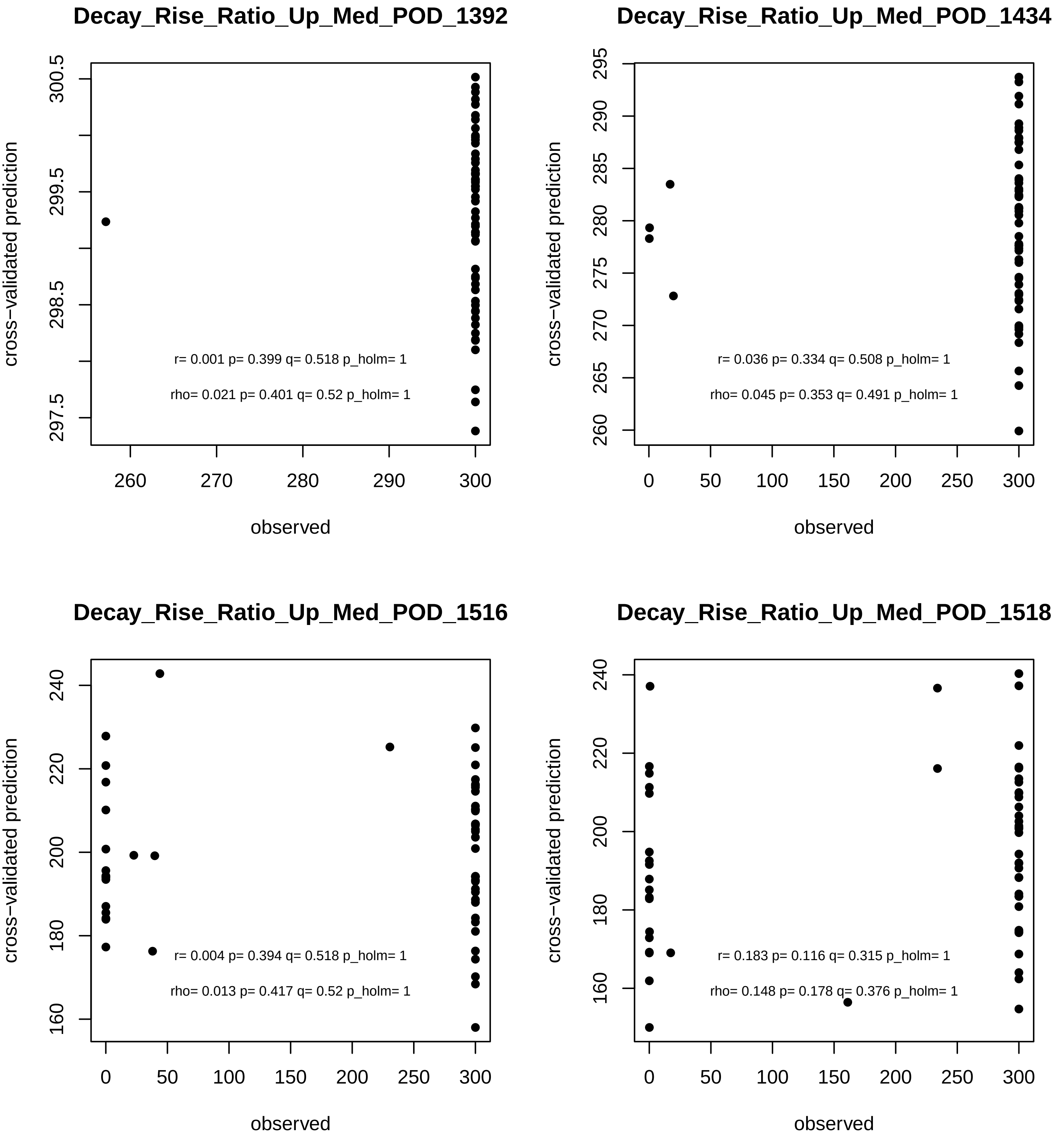
Decay_Rise_Ratio_Up_Med_POD_1392
cross−validated prediction
observed
r= 0.001 p= 0.399 q= 0.518 p_holm= 1
rho= 0.021 p= 0.401 q= 0.52 p_holm= 1
Decay_Rise_Ratio_Up_Med_POD_1434
cross−validated prediction
observed
r= 0.036 p= 0.334 q= 0.508 p_holm= 1
rho= 0.045 p= 0.353 q= 0.491 p_holm= 1
Decay_Rise_Ratio_Up_Med_POD_1516
cross−validated prediction
observed
r= 0.004 p= 0.394 q= 0.518 p_holm= 1
rho= 0.013 p= 0.417 q= 0.52 p_holm= 1
Decay_Rise_Ratio_Up_Med_POD_1518
cross−validated prediction
observed
r= 0.183 p= 0.116 q= 0.315 p_holm= 1
rho= 0.148 p= 0.178 q= 0.376 p_holm= 1

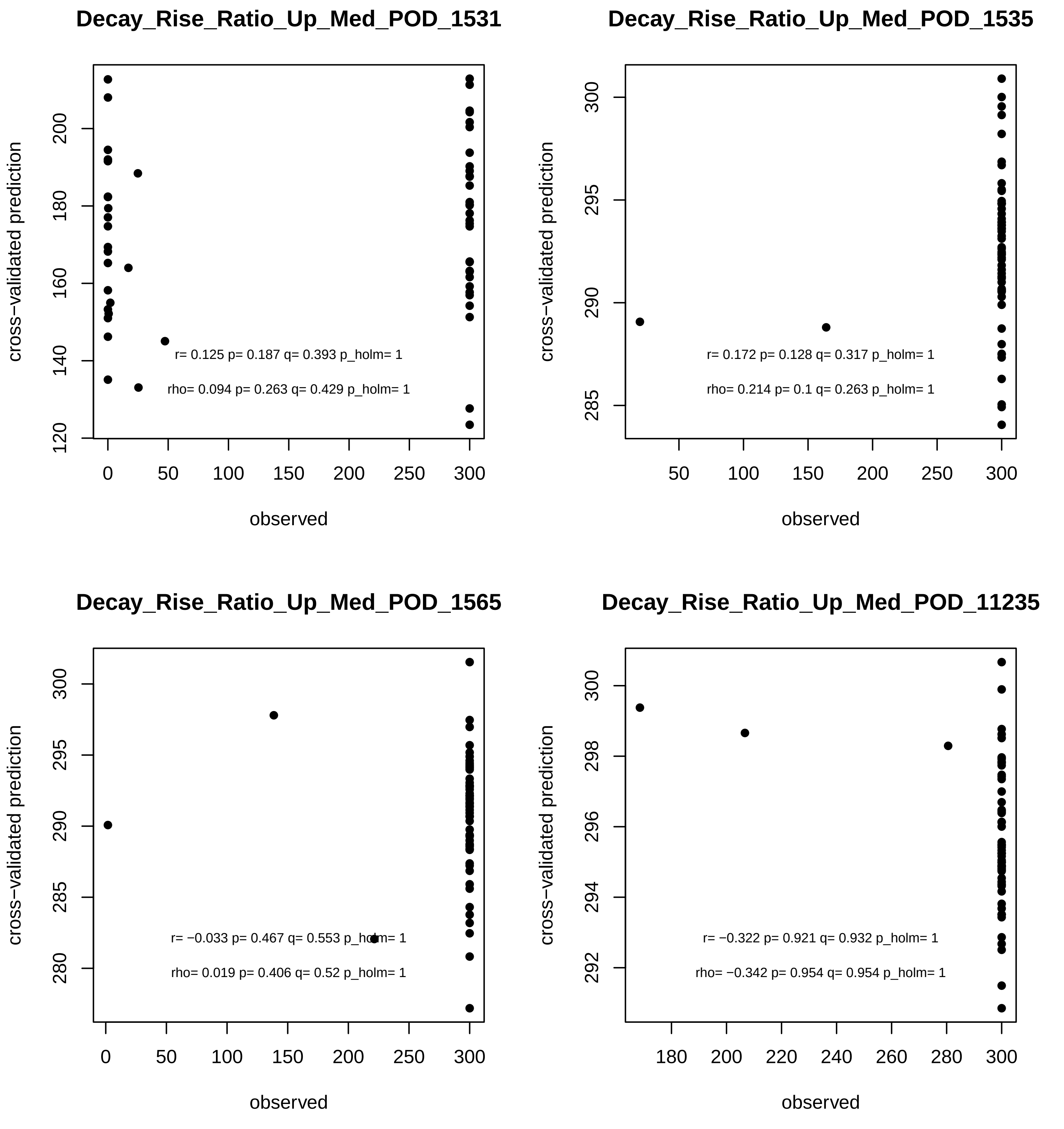
Decay_Rise_Ratio_Up_Med_POD_1531
cross−validated prediction
observed
120
140
160
180
200
0
50
100
150
200
250
300
r= 0.125 p= 0.187 q= 0.393 p_holm= 1
rho= 0.094 p= 0.263 q= 0.429 p_holm= 1
Decay_Rise_Ratio_Up_Med_POD_1535
cross−validated prediction
observed
285
290
295
300
50
100
150
200
250
300
r= 0.172 p= 0.128 q= 0.317 p_holm= 1
rho= 0.214 p= 0.1 q= 0.263 p_holm= 1
Decay_Rise_Ratio_Up_Med_POD_1565
cross−validated prediction
observed
280
285
290
295
300
0
50
100
150
200
250
300
r= −0.033 p= 0.467 q= 0.553 p_holm= 1
rho= 0.019 p= 0.406 q= 0.52 p_holm= 1
Decay_Rise_Ratio_Up_Med_POD_11235
cross−validated prediction
observed
292
294
296
298
300
180
200
220
240
260
280
300
r= −0.322 p= 0.921 q= 0.932 p_holm= 1
rho= −0.342 p= 0.954 q= 0.954 p_holm= 1

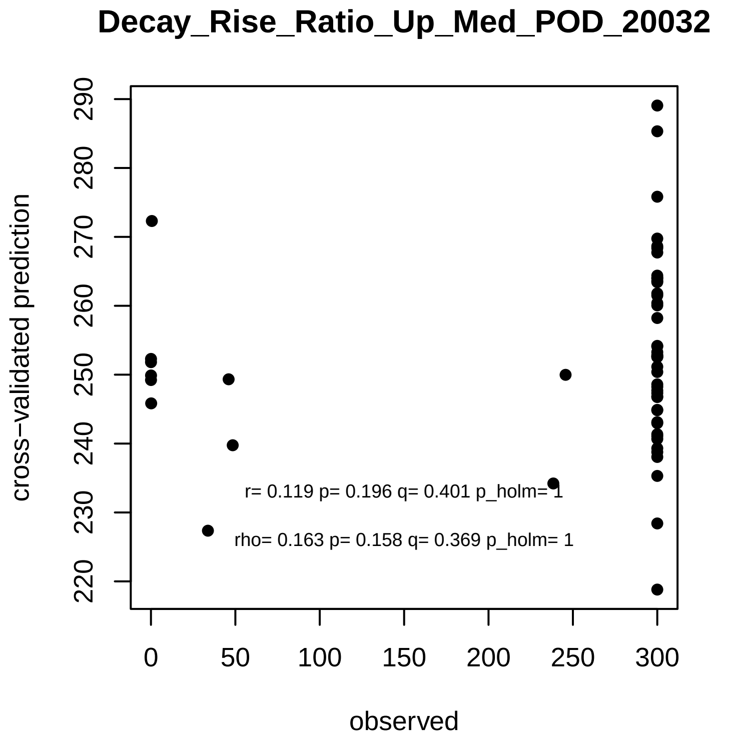
Decay_Rise_Ratio_Up_Med_POD_20032
cross−validated prediction
observed
r= 0.119 p= 0.196 q= 0.401 p_holm= 1
rho= 0.163 p= 0.158 q= 0.369 p_holm= 1

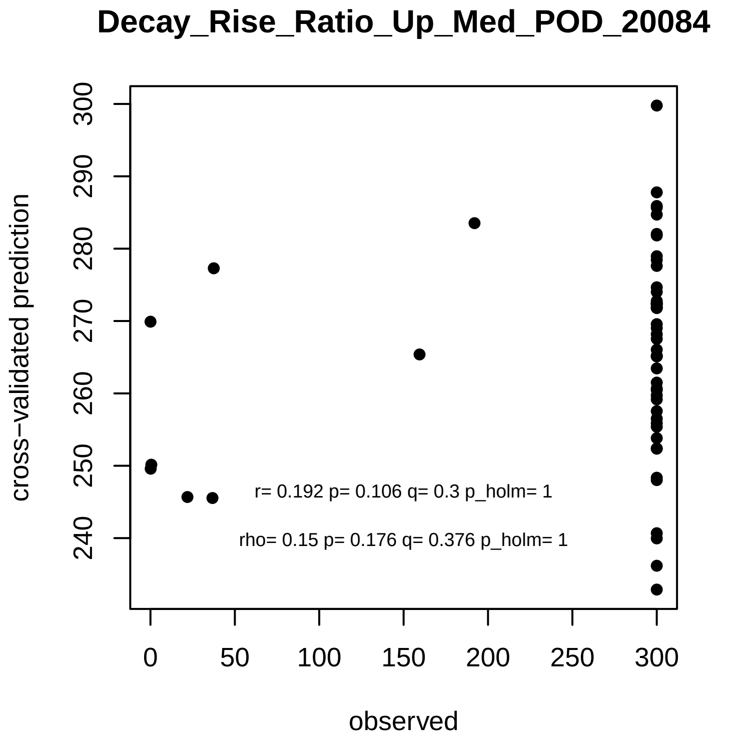
Decay_Rise_Ratio_Up_Med_POD_20084
cross−validated prediction
observed
r= 0.192 p= 0.106 q= 0.3 p_holm= 1
rho= 0.15 p= 0.176 q= 0.376 p_holm= 1

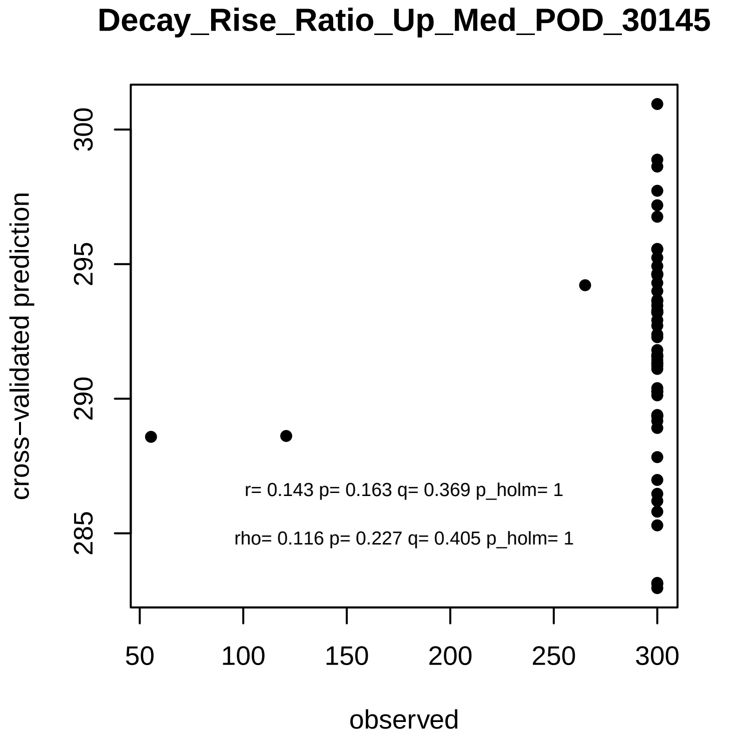
Decay_Rise_Ratio_Up_Med_POD_30145
cross−validated prediction
observed
r= 0.143 p= 0.163 q= 0.369 p_holm= 1
rho= 0.116 p= 0.227 q= 0.405 p_holm= 1

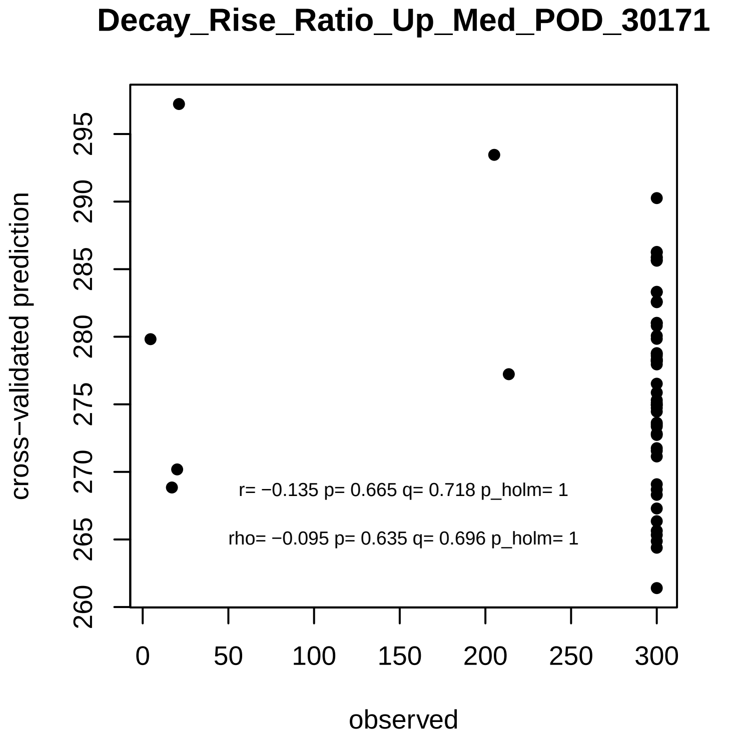
Decay_Rise_Ratio_Up_Med_POD_30171
cross−validated prediction
observed
r= −0.135 p= 0.665 q= 0.718 p_holm= 1
rho= −0.095 p= 0.635 q= 0.696 p_holm= 1

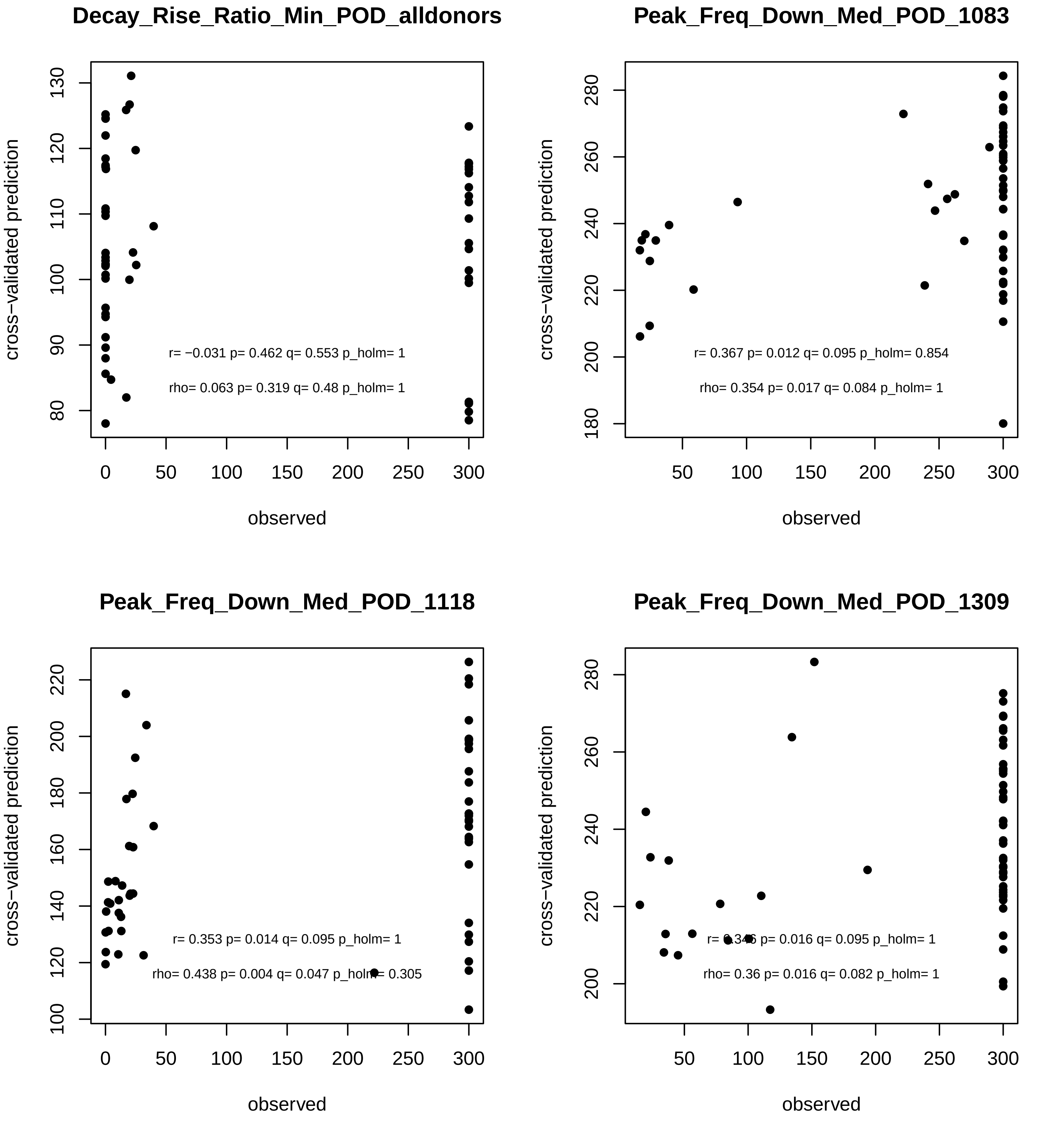

Decay_Rise_Ratio_Min_POD_alldonors
r= −0.031 p= 0.462 q= 0.553 p_holm= 1
rho= 0.063 p= 0.319 q= 0.48 p_holm= 1
cross−validated prediction
observed
Peak_Freq_Down_Med_POD_1083
r= 0.367 p= 0.012 q= 0.095 p_holm= 0.854
rho= 0.354 p= 0.017 q= 0.084 p_holm= 1
cross−validated prediction
observed
Peak_Freq_Down_Med_POD_1118
r= 0.353 p= 0.014 q= 0.095 p_holm= 1
rho= 0.438 p= 0.004 q= 0.047 p_holm= 0.305
cross−validated prediction
observed
Peak_Freq_Down_Med_POD_1309
r= 0.346 p= 0.016 q= 0.095 p_holm= 1
rho= 0.36 p= 0.016 q= 0.082 p_holm= 1
cross−validated prediction
observed

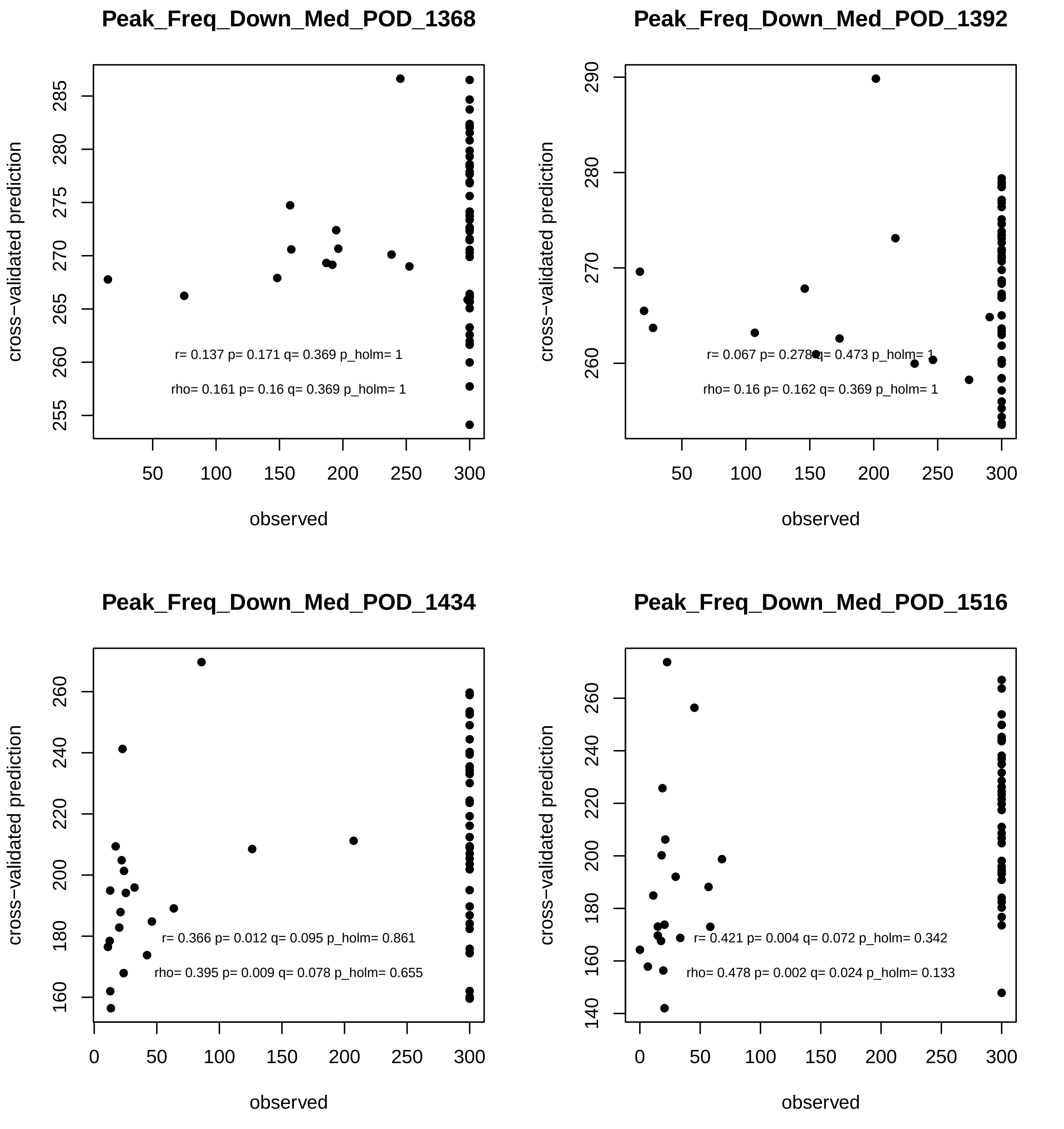
Peak_Freq_Down_Med_POD_1368
r= 0.137 p= 0.171 q= 0.369 p_holm= 1
rho= 0.161 p= 0.16 q= 0.369 p_holm= 1
cross−validated prediction
observed
Peak_Freq_Down_Med_POD_1392
r= 0.067 p= 0.278 q= 0.473 p_holm= 1
rho= 0.16 p= 0.162 q= 0.369 p_holm= 1
cross−validated prediction
observed
Peak_Freq_Down_Med_POD_1434
r= 0.366 p= 0.012 q= 0.095 p_holm= 0.861
rho= 0.395 p= 0.009 q= 0.078 p_holm= 0.655
cross−validated prediction
observed
Peak_Freq_Down_Med_POD_1516
r= 0.421 p= 0.004 q= 0.072 p_holm= 0.342
rho= 0.478 p= 0.002 q= 0.024 p_holm= 0.133
cross−validated prediction
observed

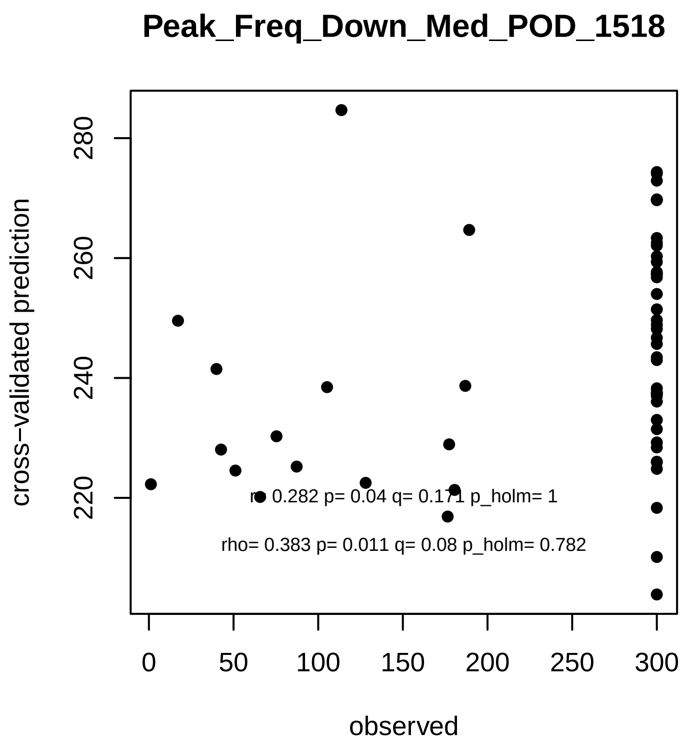
Peak_Freq_Down_Med_POD_1518
r= 0.282 p= 0.04 q= 0.171 p_holm= 1
rho= 0.383 p= 0.011 q= 0.08 p_holm= 0.782
cross−validated prediction
observed

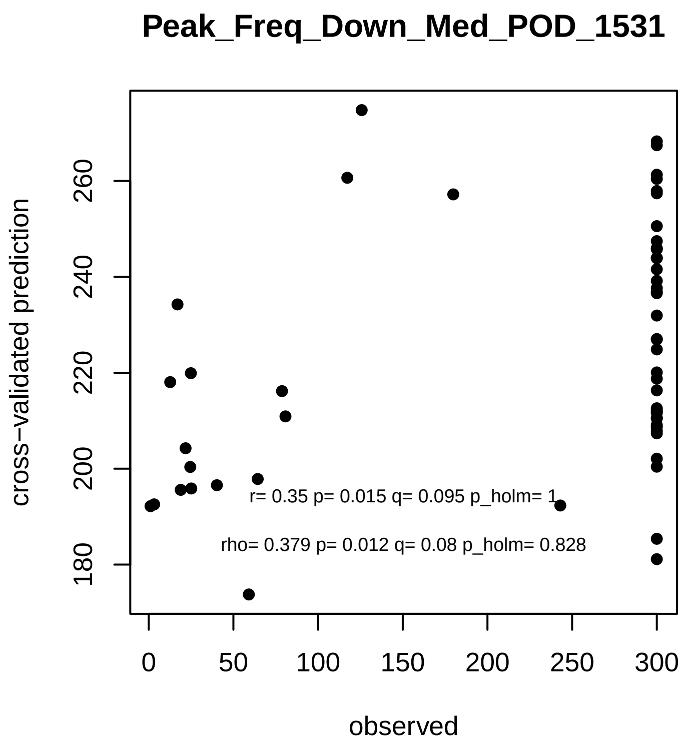
Peak_Freq_Down_Med_POD_1531
r= 0.35 p= 0.015 q= 0.095 p_holm= 1
rho= 0.379 p= 0.012 q= 0.08 p_holm= 0.828
cross−validated prediction
observed

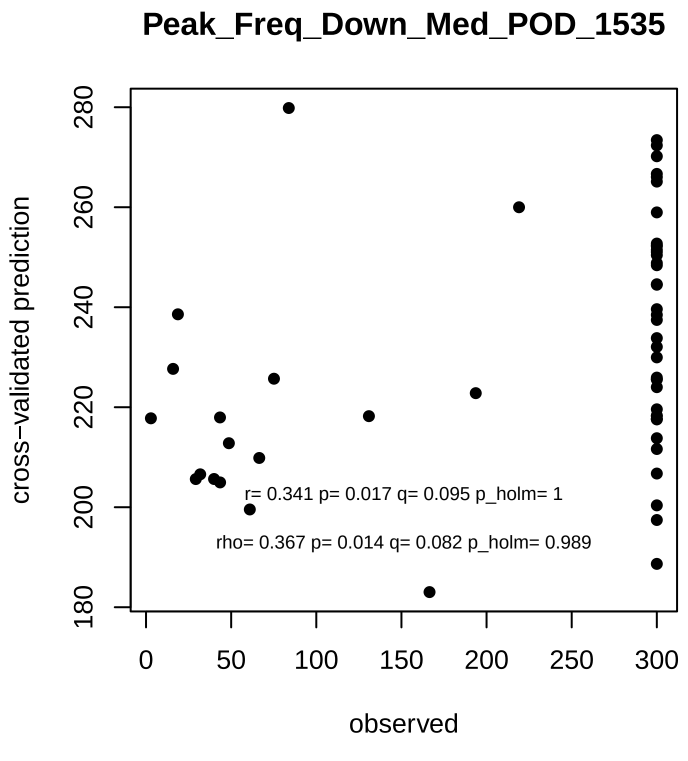
Peak_Freq_Down_Med_POD_1535
r= 0.341 p= 0.017 q= 0.095 p_holm= 1
rho= 0.367 p= 0.014 q= 0.082 p_holm= 0.989
cross−validated prediction
observed

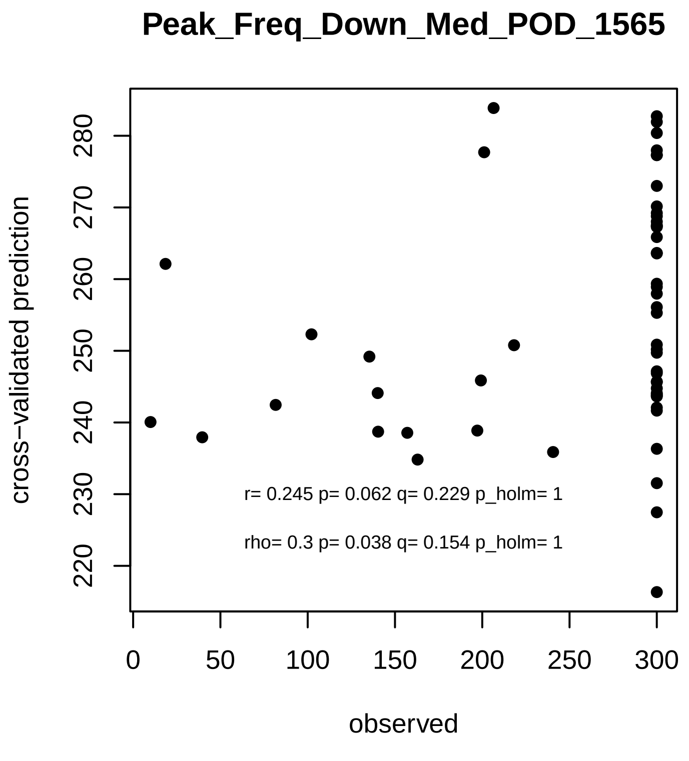
Peak_Freq_Down_Med_POD_1565
r= 0.245 p= 0.062 q= 0.229 p_holm= 1
rho= 0.3 p= 0.038 q= 0.154 p_holm= 1
cross−validated prediction
observed

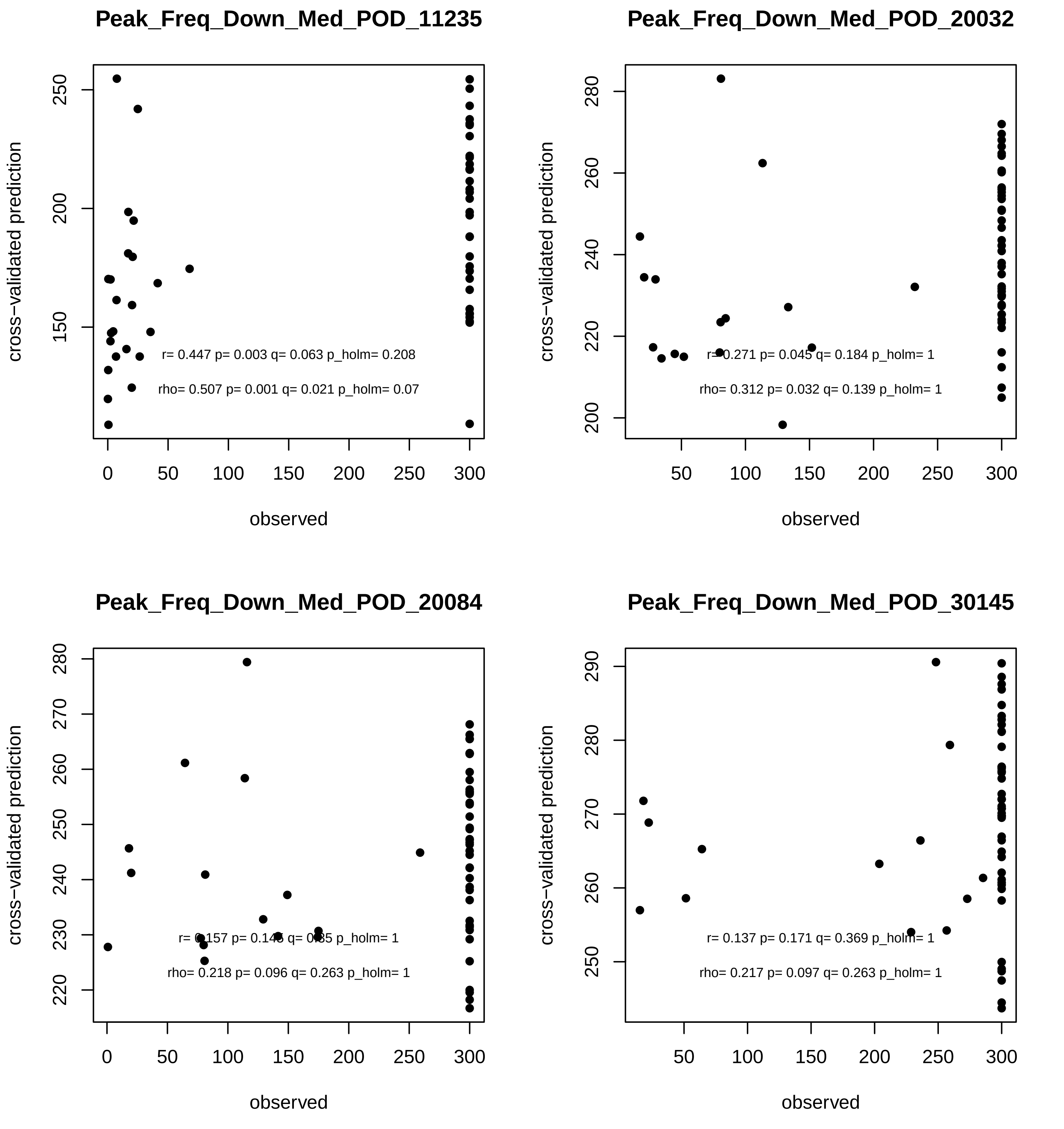
Peak_Freq_Down_Med_POD_11235
r= 0.447 p= 0.003 q= 0.063 p_holm= 0.208
rho= 0.507 p= 0.001 q= 0.021 p_holm= 0.07
cross−validated prediction
observed
Peak_Freq_Down_Med_POD_20032
r= 0.271 p= 0.045 q= 0.184 p_holm= 1
rho= 0.312 p= 0.032 q= 0.139 p_holm= 1
cross−validated prediction
observed
Peak_Freq_Down_Med_POD_20084
rho= 0.218 p= 0.096 q= 0.263 p_holm= 1
cross−validated prediction
observed
Peak_Freq_Down_Med_POD_30145
r= 0.137 p= 0.171 q= 0.369 p_holm= 1
rho= 0.217 p= 0.097 q= 0.263 p_holm= 1
cross−validated prediction
observed

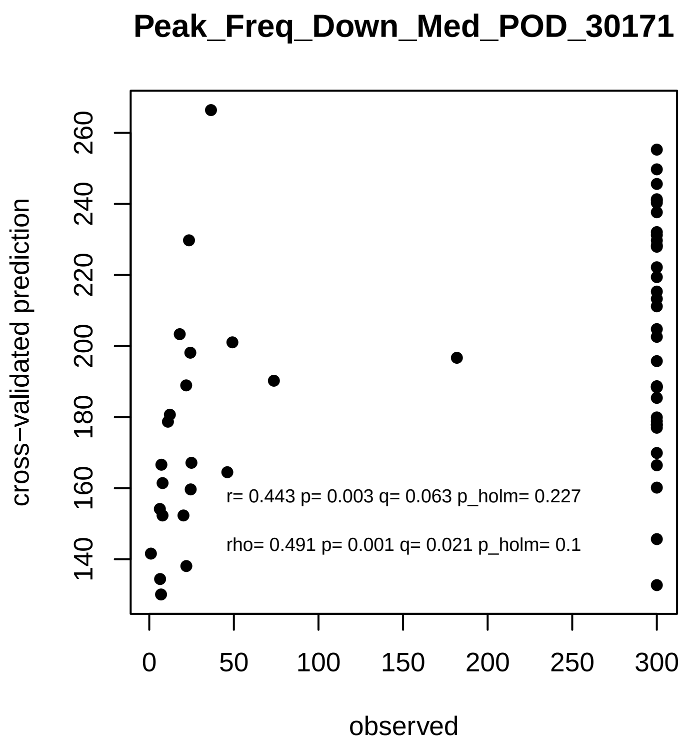
Peak_Freq_Down_Med_POD_30171
cross−validated prediction
140
160
180
200
220
240
260
r= 0.443 p= 0.003 q= 0.063 p_holm= 0.227
rho= 0.491 p= 0.001 q= 0.021 p_holm= 0.1
0
50
100
150
200
250
300
observed

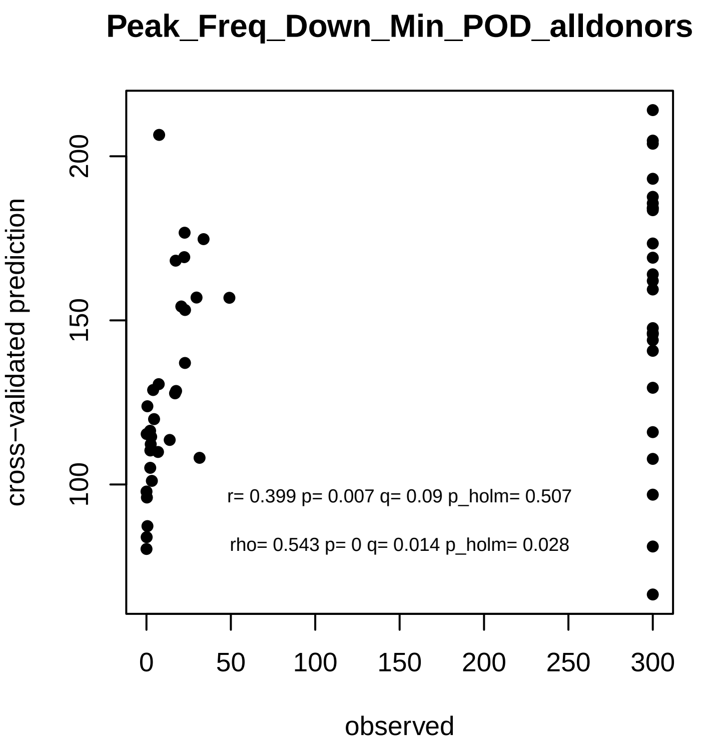
Peak_Freq_Down_Min_POD_alldonors
cross−validated prediction
100
150
200
r= 0.399 p= 0.007 q= 0.09 p_holm= 0.507
rho= 0.543 p= 0 q= 0.014 p_holm= 0.028
0
50
100
150
200
250
300
observed

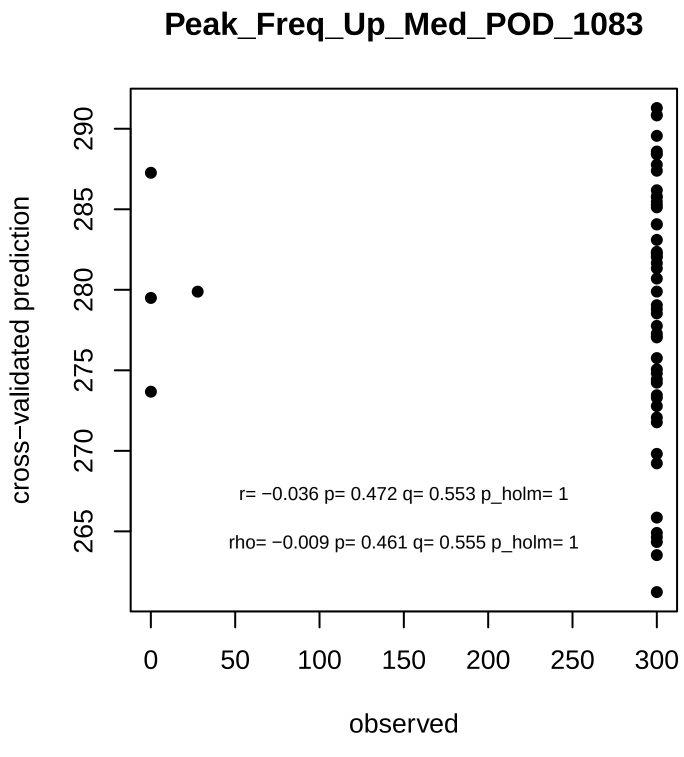
Peak_Freq_Up_Med_POD_1083
cross−validated prediction
265
270
275
280
285
290
r= −0.036 p= 0.472 q= 0.553 p_holm= 1
rho= −0.009 p= 0.461 q= 0.555 p_holm= 1
0
50
100
150
200
250
300
observed

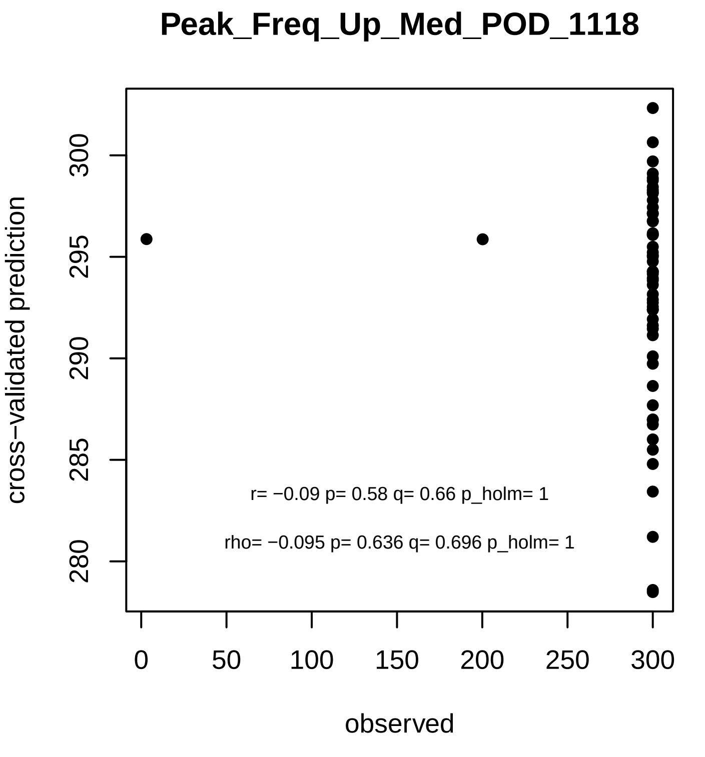
Peak_Freq_Up_Med_POD_1118
cross−validated prediction
280
285
290
295
300
r= −0.09 p= 0.58 q= 0.66 p_holm= 1
rho= −0.095 p= 0.636 q= 0.696 p_holm= 1
0
50
100
150
200
250
300
observed

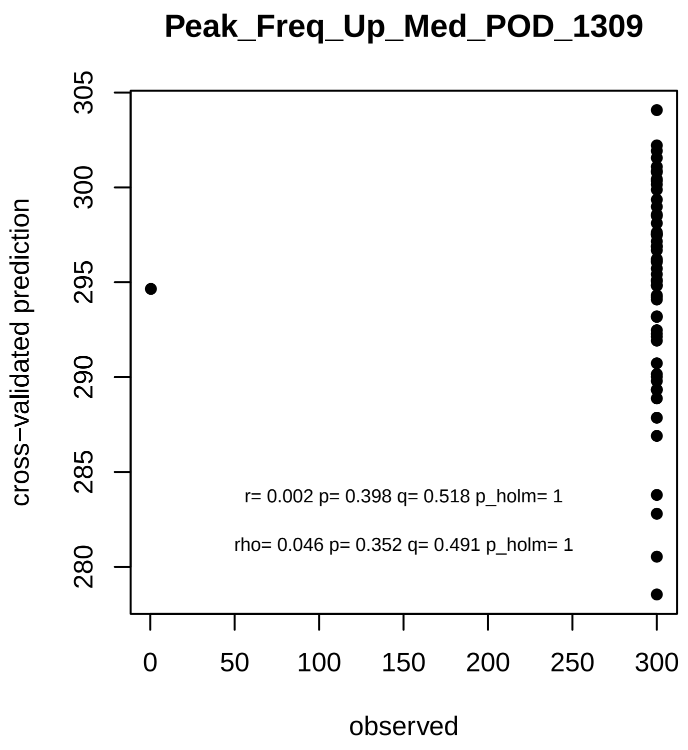
Peak_Freq_Up_Med_POD_1309
r= 0.002 p= 0.398 q= 0.518 p_holm= 1
rho= 0.046 p= 0.352 q= 0.491 p_holm= 1
cross−validated prediction
observed

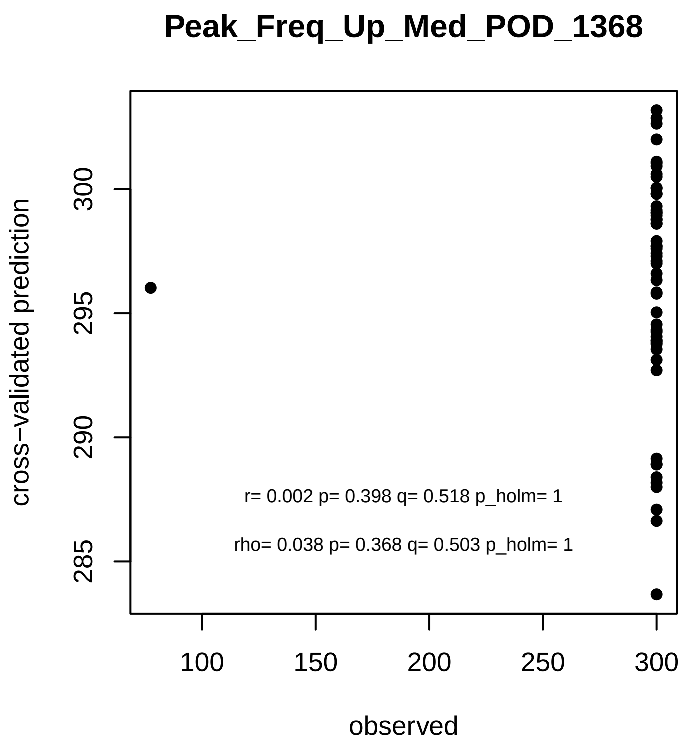
Peak_Freq_Up_Med_POD_1368
r= 0.002 p= 0.398 q= 0.518 p_holm= 1
rho= 0.038 p= 0.368 q= 0.503 p_holm= 1
cross−validated prediction
observed

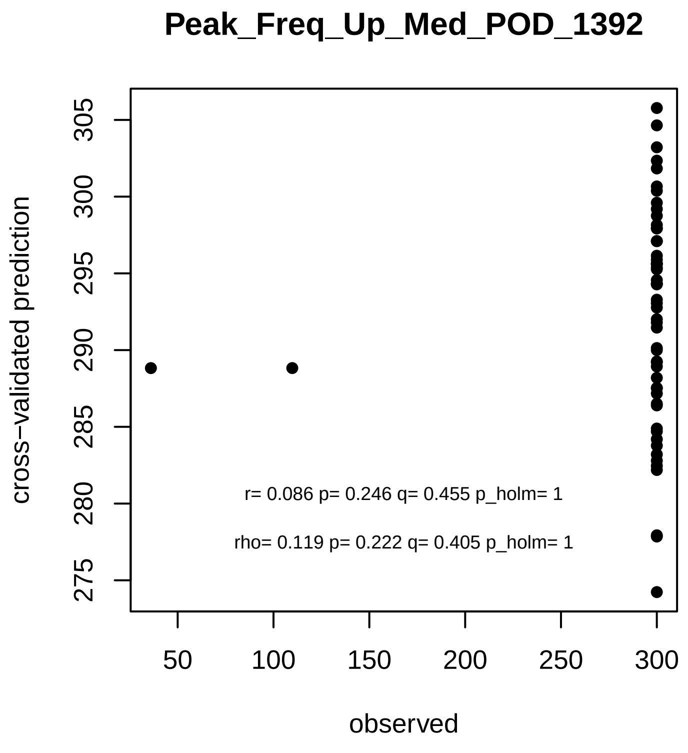
Peak_Freq_Up_Med_POD_1392
r= 0.086 p= 0.246 q= 0.455 p_holm= 1
rho= 0.119 p= 0.222 q= 0.405 p_holm= 1
cross−validated prediction
observed

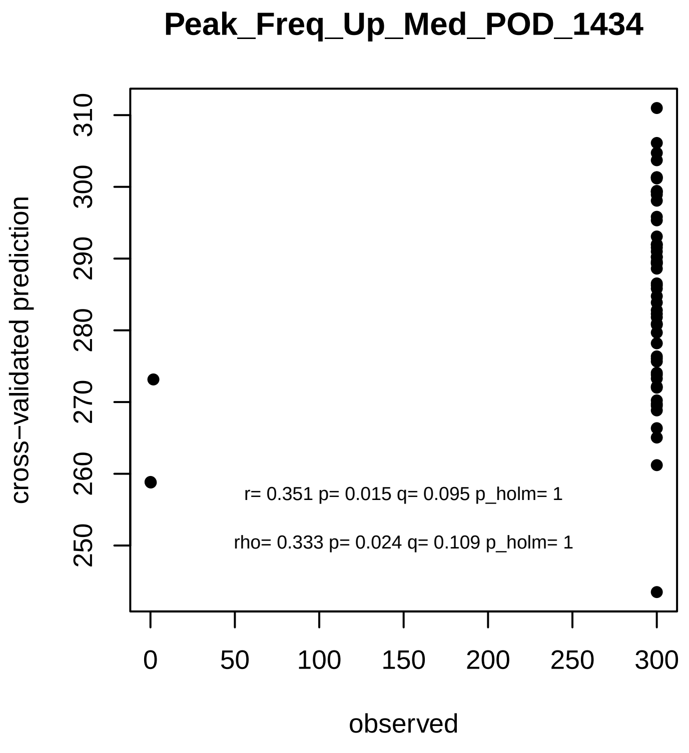
Peak_Freq_Up_Med_POD_1434
r= 0.351 p= 0.015 q= 0.095 p_holm= 1
rho= 0.333 p= 0.024 q= 0.109 p_holm= 1
cross−validated prediction
observed

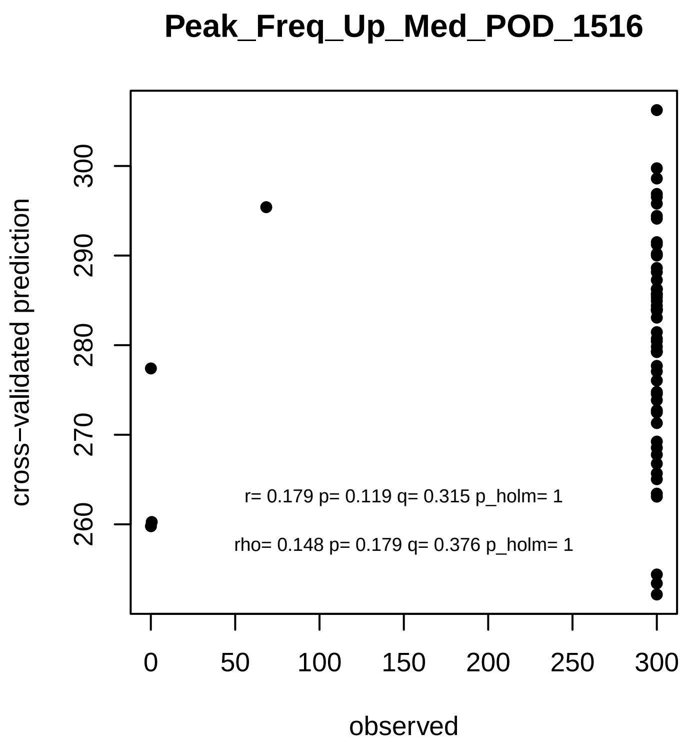
Peak_Freq_Up_Med_POD_1516
r= 0.179 p= 0.119 q= 0.315 p_holm= 1
rho= 0.148 p= 0.179 q= 0.376 p_holm= 1
cross−validated prediction
observed

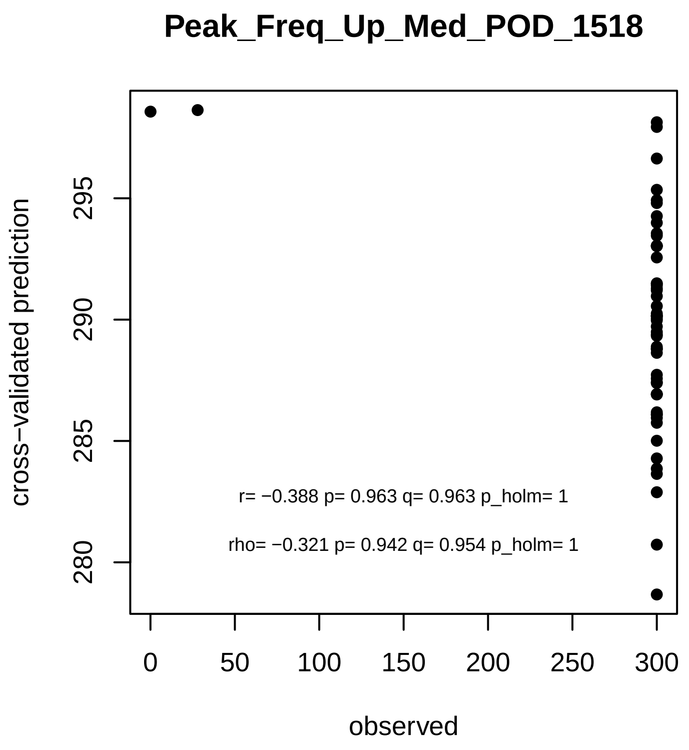
Peak_Freq_Up_Med_POD_1518
r= −0.388 p= 0.963 q= 0.963 p_holm= 1
rho= −0.321 p= 0.942 q= 0.954 p_holm= 1
cross−validated prediction
observed

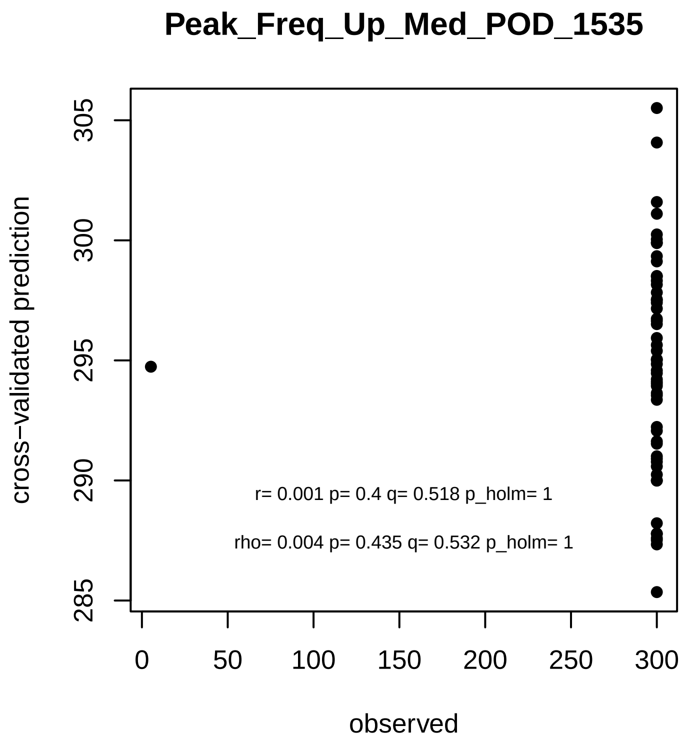
Peak_Freq_Up_Med_POD_1535
r= 0.001 p= 0.4 q= 0.518 p_holm= 1
rho= 0.004 p= 0.435 q= 0.532 p_holm= 1
cross−validated prediction
observed

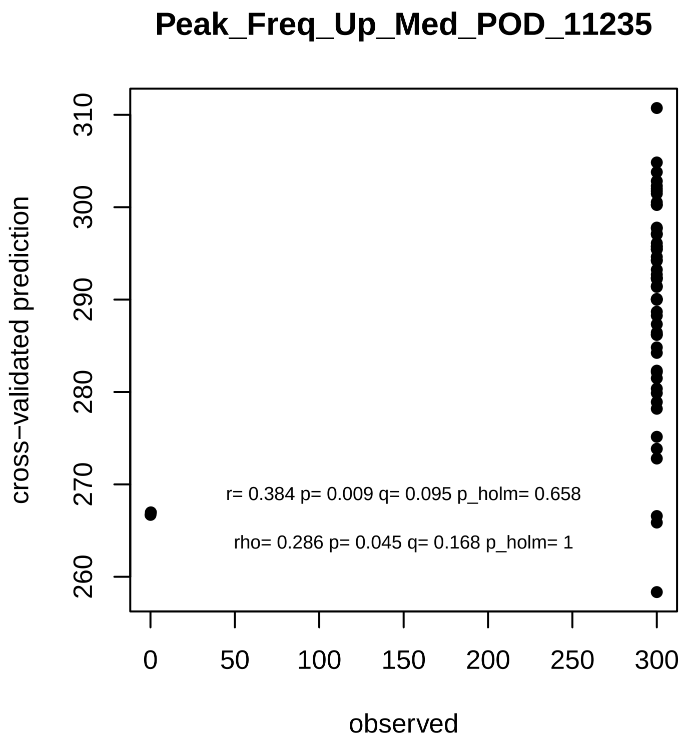
Peak_Freq_Up_Med_POD_11235
r= 0.384 p= 0.009 q= 0.095 p_holm= 0.658
rho= 0.286 p= 0.045 q= 0.168 p_holm= 1
cross−validated prediction
observed

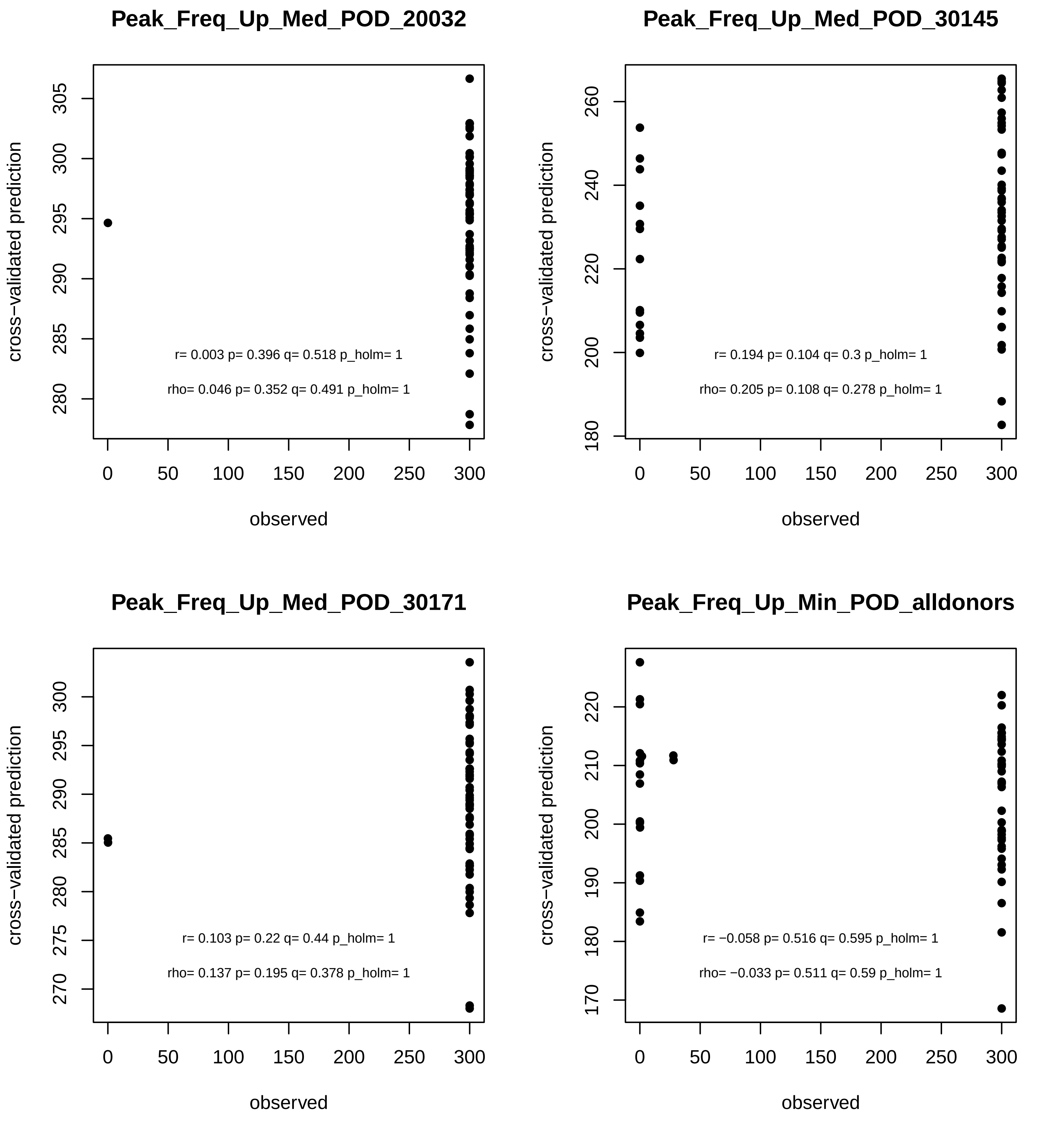
Peak_Freq_Up_Med_POD_20032
r= 0.003 p= 0.396 q= 0.518 p_holm= 1
rho= 0.046 p= 0.352 q= 0.491 p_holm= 1
Peak_Freq_Up_Med_POD_30145
r= 0.194 p= 0.104 q= 0.3 p_holm= 1
rho= 0.205 p= 0.108 q= 0.278 p_holm= 1
Peak_Freq_Up_Med_POD_30171
r= 0.103 p= 0.22 q= 0.44 p_holm= 1
rho= 0.137 p= 0.195 q= 0.378 p_holm= 1
Peak_Freq_Up_Min_POD_alldonors
r= −0.058 p= 0.516 q= 0.595 p_holm= 1
rho= −0.033 p= 0.511 q= 0.59 p_holm= 1
cross−validated prediction
observed

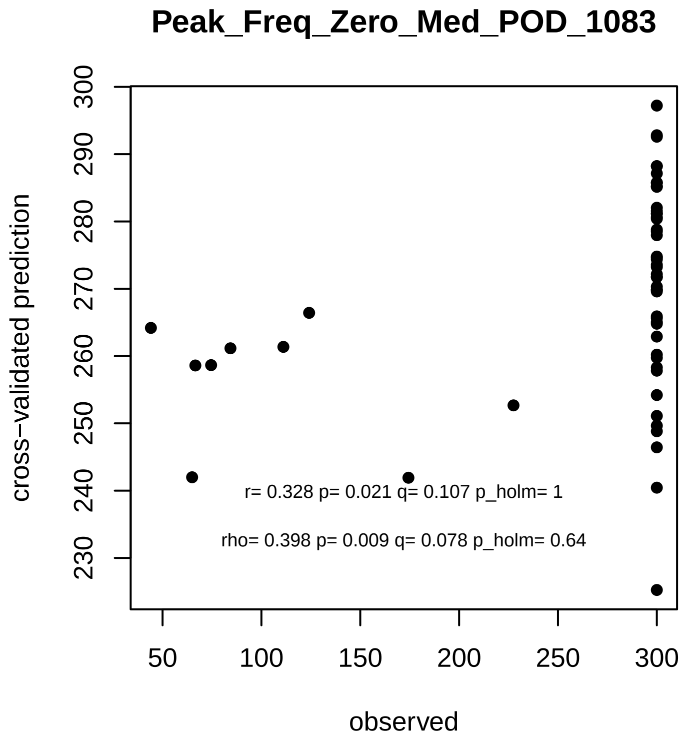
Peak_Freq_Zero_Med_POD_1083
cross−validated prediction
observed
r= 0.328 p= 0.021 q= 0.107 p_holm= 1
rho= 0.398 p= 0.009 q= 0.078 p_holm= 0.64

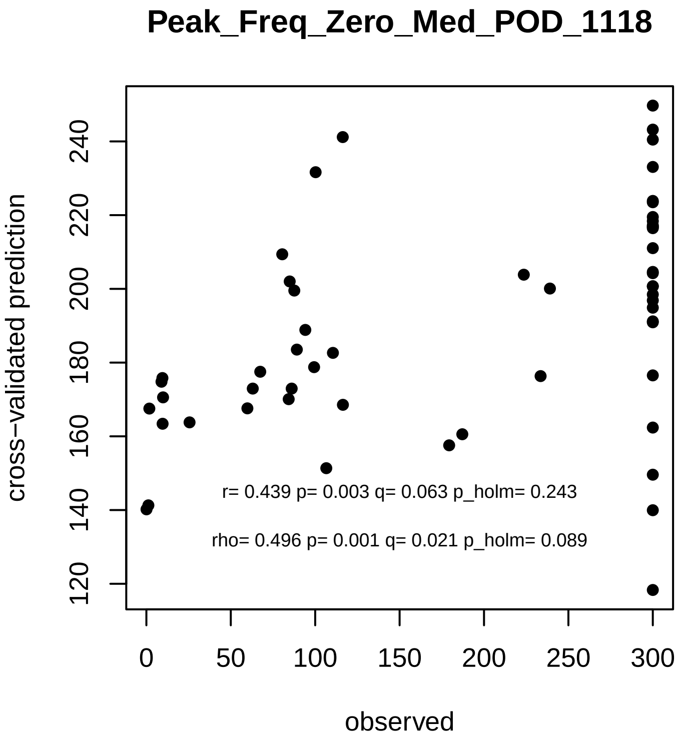
Peak_Freq_Zero_Med_POD_1118
cross−validated prediction
observed
r= 0.439 p= 0.003 q= 0.063 p_holm= 0.243
rho= 0.496 p= 0.001 q= 0.021 p_holm= 0.089

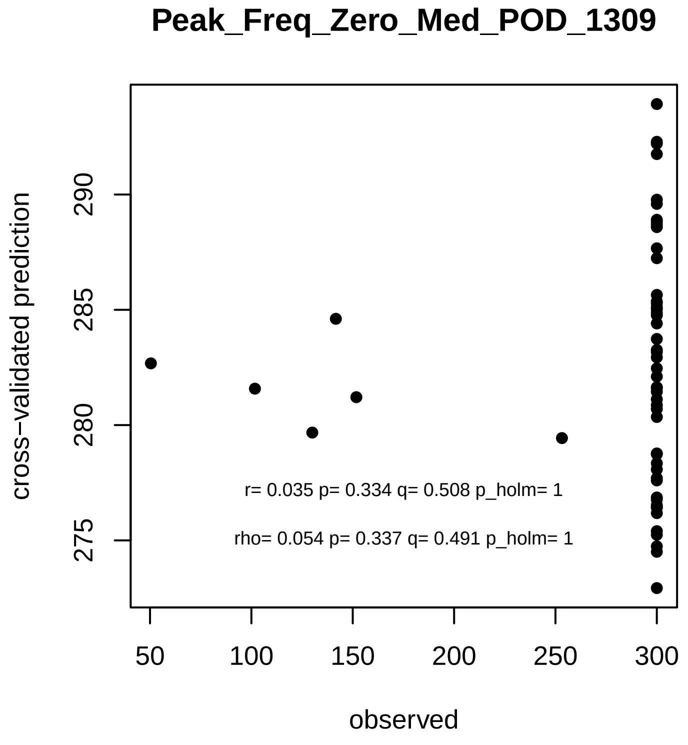
Peak_Freq_Zero_Med_POD_1309
cross−validated prediction
observed
r= 0.035 p= 0.334 q= 0.508 p_holm= 1
rho= 0.054 p= 0.337 q= 0.491 p_holm= 1

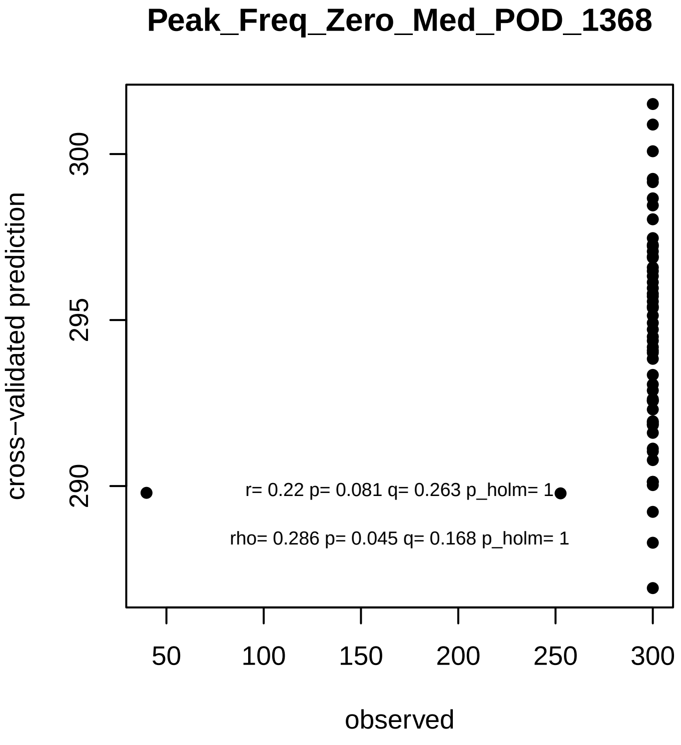
Peak_Freq_Zero_Med_POD_1368
cross−validated prediction
observed
r= 0.22 p= 0.081 q= 0.263 p_holm= 1
rho= 0.286 p= 0.045 q= 0.168 p_holm= 1

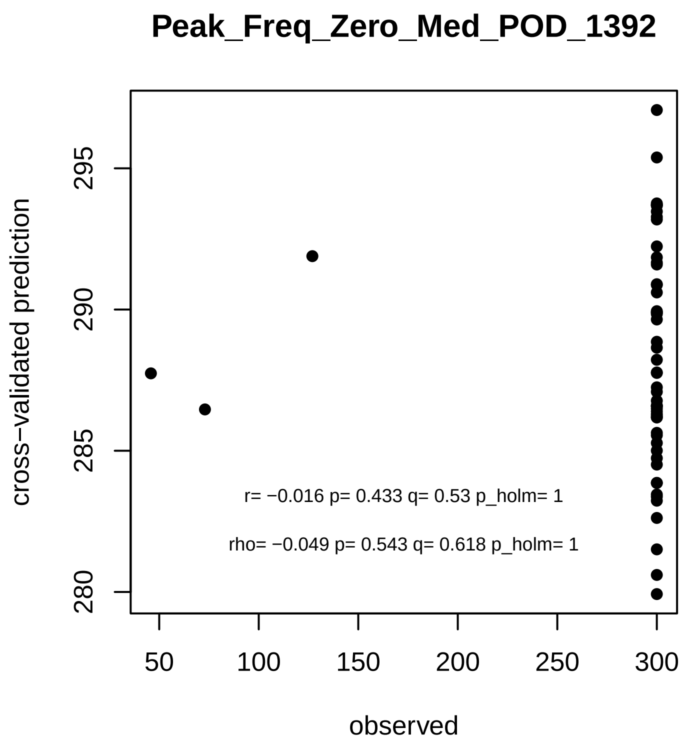
Peak_Freq_Zero_Med_POD_1392
r= −0.016 p= 0.433 q= 0.53 p_holm= 1
rho= −0.049 p= 0.543 q= 0.618 p_holm= 1
cross−validated prediction
observed

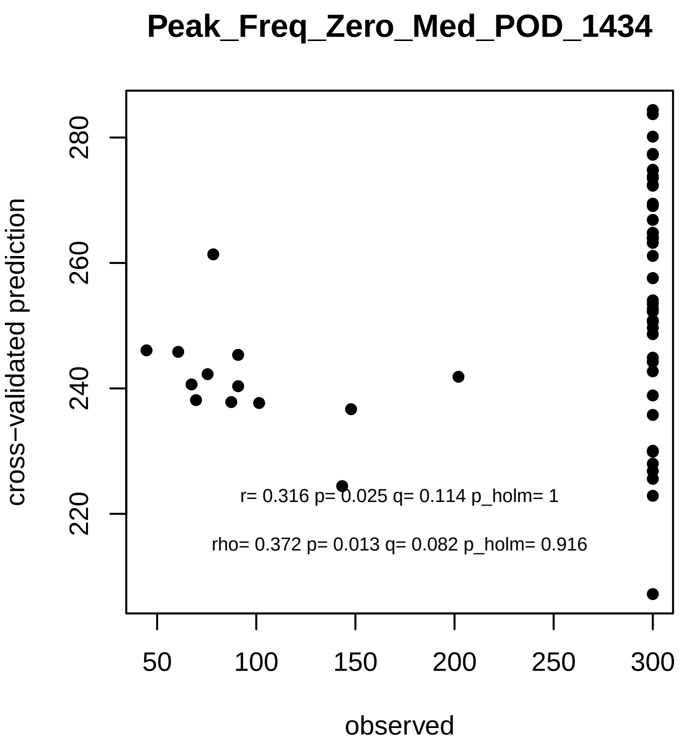
Peak_Freq_Zero_Med_POD_1434
r= 0.316 p= 0.025 q= 0.114 p_holm= 1
rho= 0.372 p= 0.013 q= 0.082 p_holm= 0.916
cross−validated prediction
observed

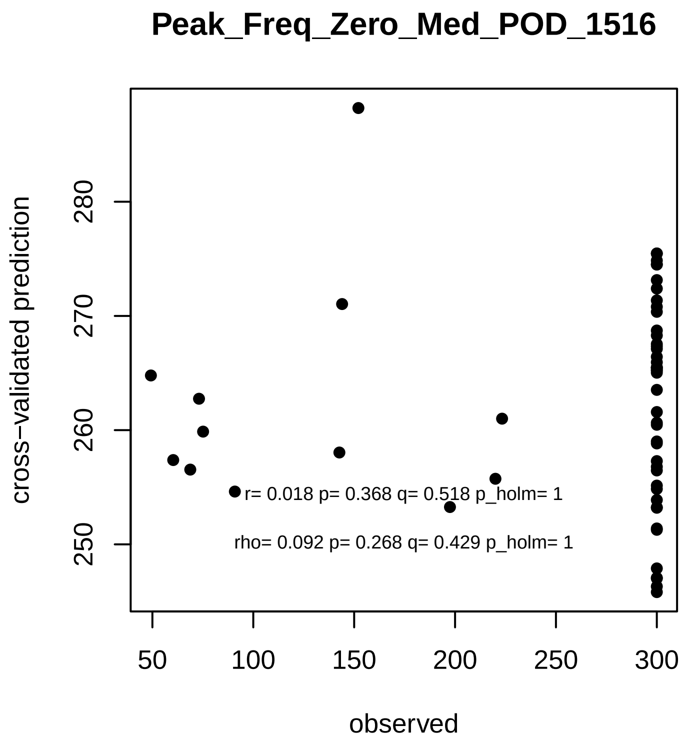
Peak_Freq_Zero_Med_POD_1516
r= 0.018 p= 0.368 q= 0.518 p_holm= 1
rho= 0.092 p= 0.268 q= 0.429 p_holm= 1
cross−validated prediction
observed

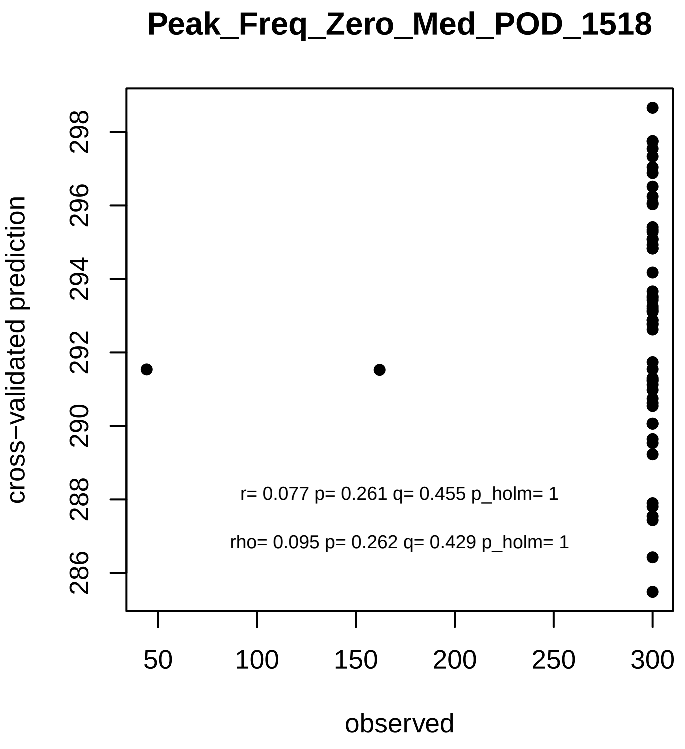
Peak_Freq_Zero_Med_POD_1518
r= 0.077 p= 0.261 q= 0.455 p_holm= 1
rho= 0.095 p= 0.262 q= 0.429 p_holm= 1
cross−validated prediction
observed

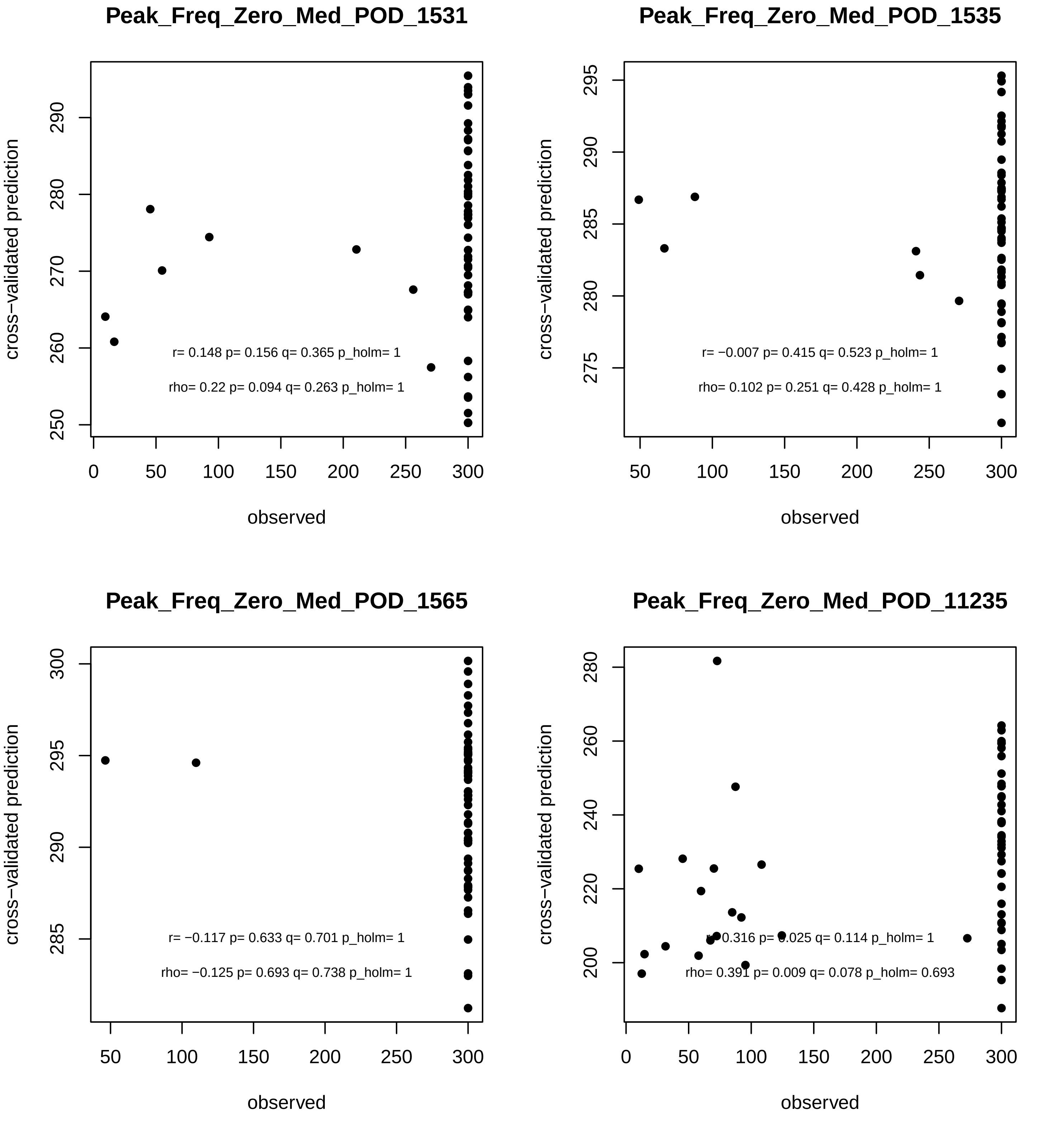
Peak_Freq_Zero_Med_POD_1531
cross−validated prediction
observed
r= 0.148 p= 0.156 q= 0.365 p_holm= 1
rho= 0.22 p= 0.094 q= 0.263 p_holm= 1
Peak_Freq_Zero_Med_POD_1535
cross−validated prediction
observed
r= −0.007 p= 0.415 q= 0.523 p_holm= 1
rho= 0.102 p= 0.251 q= 0.428 p_holm= 1
Peak_Freq_Zero_Med_POD_1565
cross−validated prediction
observed
r= −0.117 p= 0.633 q= 0.701 p_holm= 1
rho= −0.125 p= 0.693 q= 0.738 p_holm= 1
Peak_Freq_Zero_Med_POD_11235
cross−validated prediction
observed
0.316 p= 0.025 q= 0.114 p_holm= 1
rho= 0.391 p= 0.009 q= 0.078 p_holm= 0.693

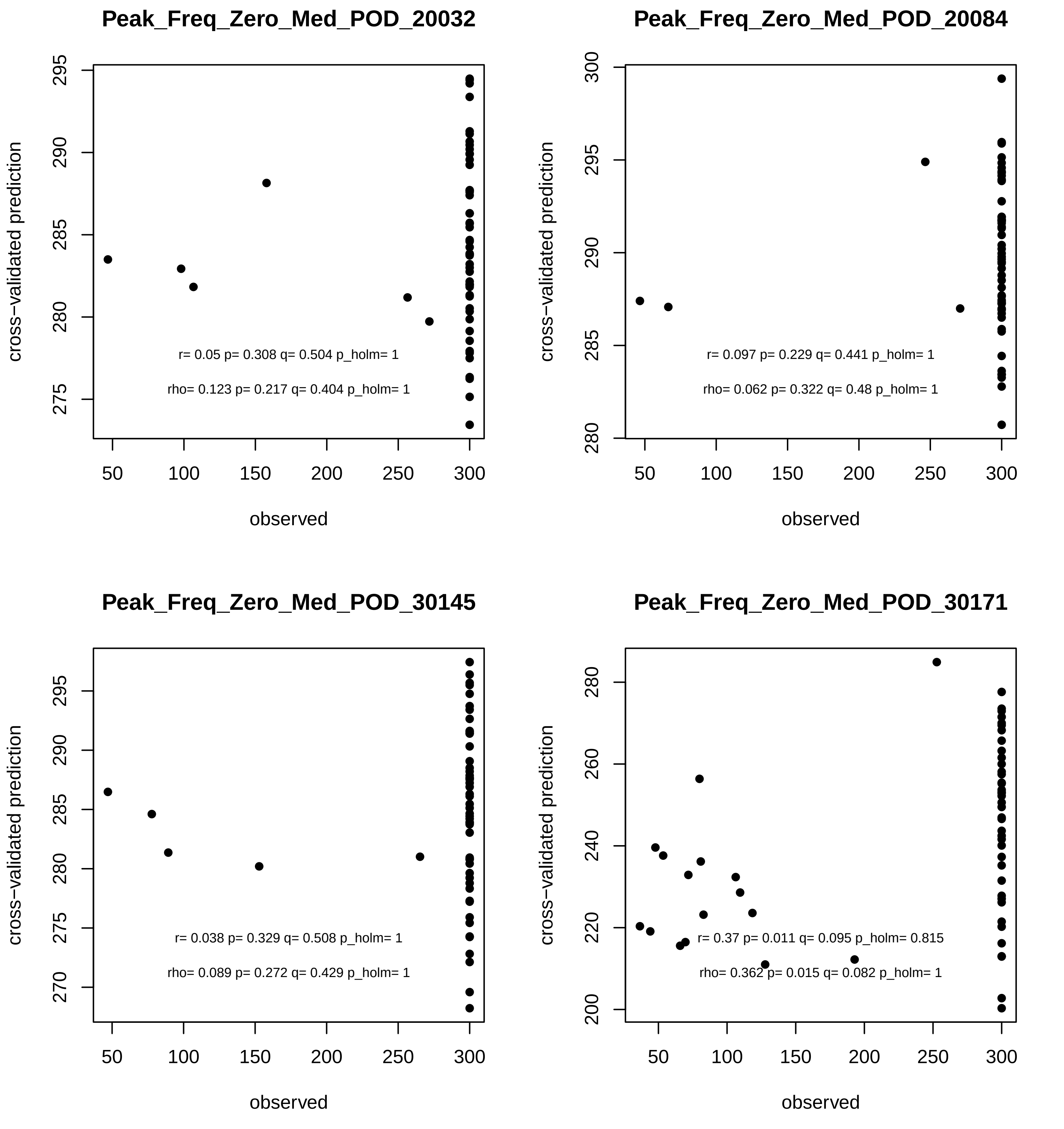
Peak_Freq_Zero_Med_POD_20032
r= 0.05 p= 0.308 q= 0.504 p_holm= 1
rho= 0.123 p= 0.217 q= 0.404 p_holm= 1
Peak_Freq_Zero_Med_POD_20084
r= 0.097 p= 0.229 q= 0.441 p_holm= 1
rho= 0.062 p= 0.322 q= 0.48 p_holm= 1
Peak_Freq_Zero_Med_POD_30145
r= 0.038 p= 0.329 q= 0.508 p_holm= 1
rho= 0.089 p= 0.272 q= 0.429 p_holm= 1
Peak_Freq_Zero_Med_POD_30171
r= 0.37 p= 0.011 q= 0.095 p_holm= 0.815
rho= 0.362 p= 0.015 q= 0.082 p_holm= 1
cross−validated prediction
observed

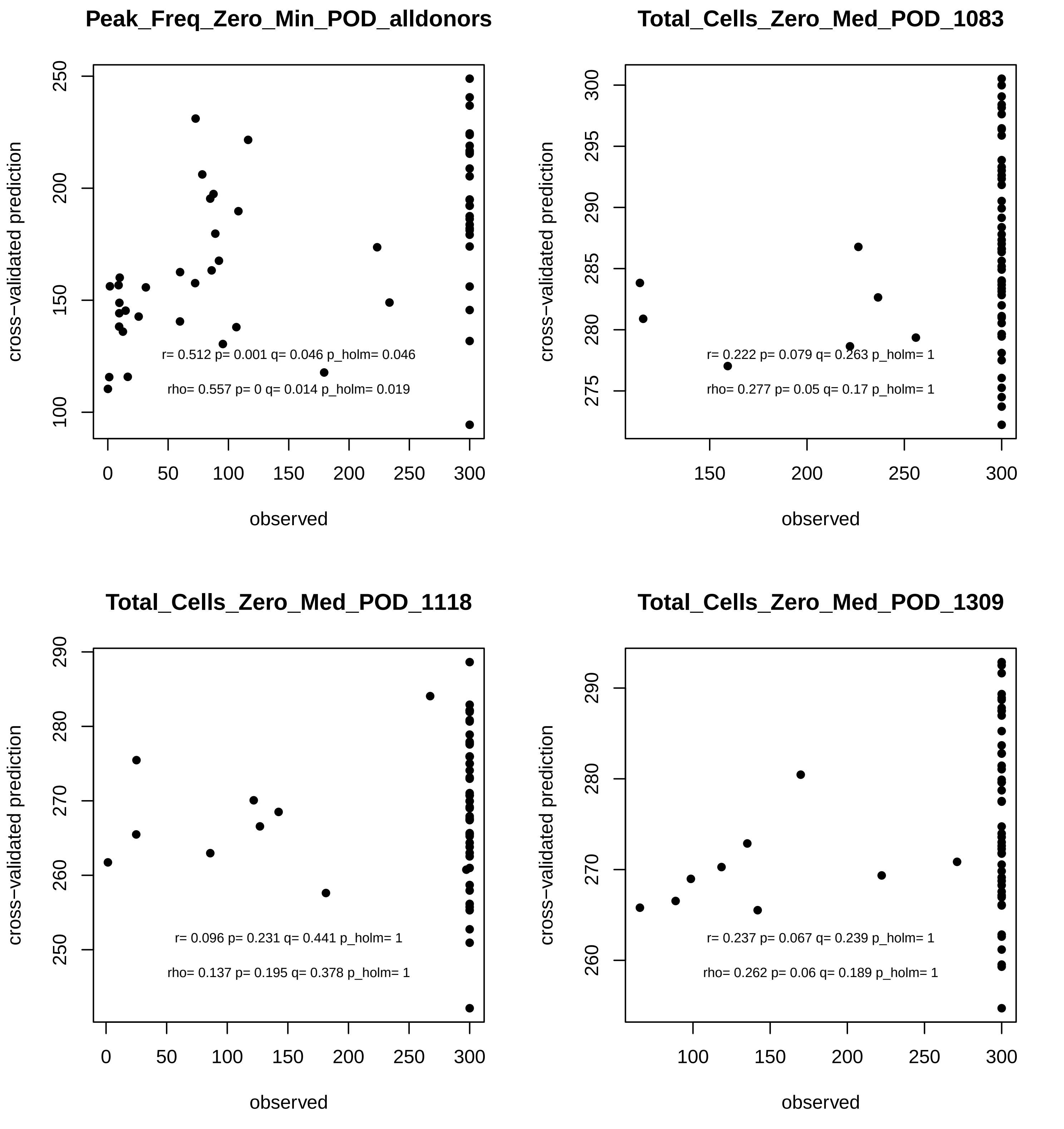
Peak_Freq_Zero_Min_POD_alldonors
r= 0.512 p= 0.001 q= 0.046 p_holm= 0.046
rho= 0.557 p= 0 q= 0.014 p_holm= 0.019
cross−validated prediction
observed
Total_Cells_Zero_Med_POD_1083
r= 0.222 p= 0.079 q= 0.263 p_holm= 1
rho= 0.277 p= 0.05 q= 0.17 p_holm= 1
cross−validated prediction
observed
Total_Cells_Zero_Med_POD_1118
r= 0.096 p= 0.231 q= 0.441 p_holm= 1
rho= 0.137 p= 0.195 q= 0.378 p_holm= 1
cross−validated prediction
observed
Total_Cells_Zero_Med_POD_1309
r= 0.237 p= 0.067 q= 0.239 p_holm= 1
rho= 0.262 p= 0.06 q= 0.189 p_holm= 1
cross−validated prediction
observed

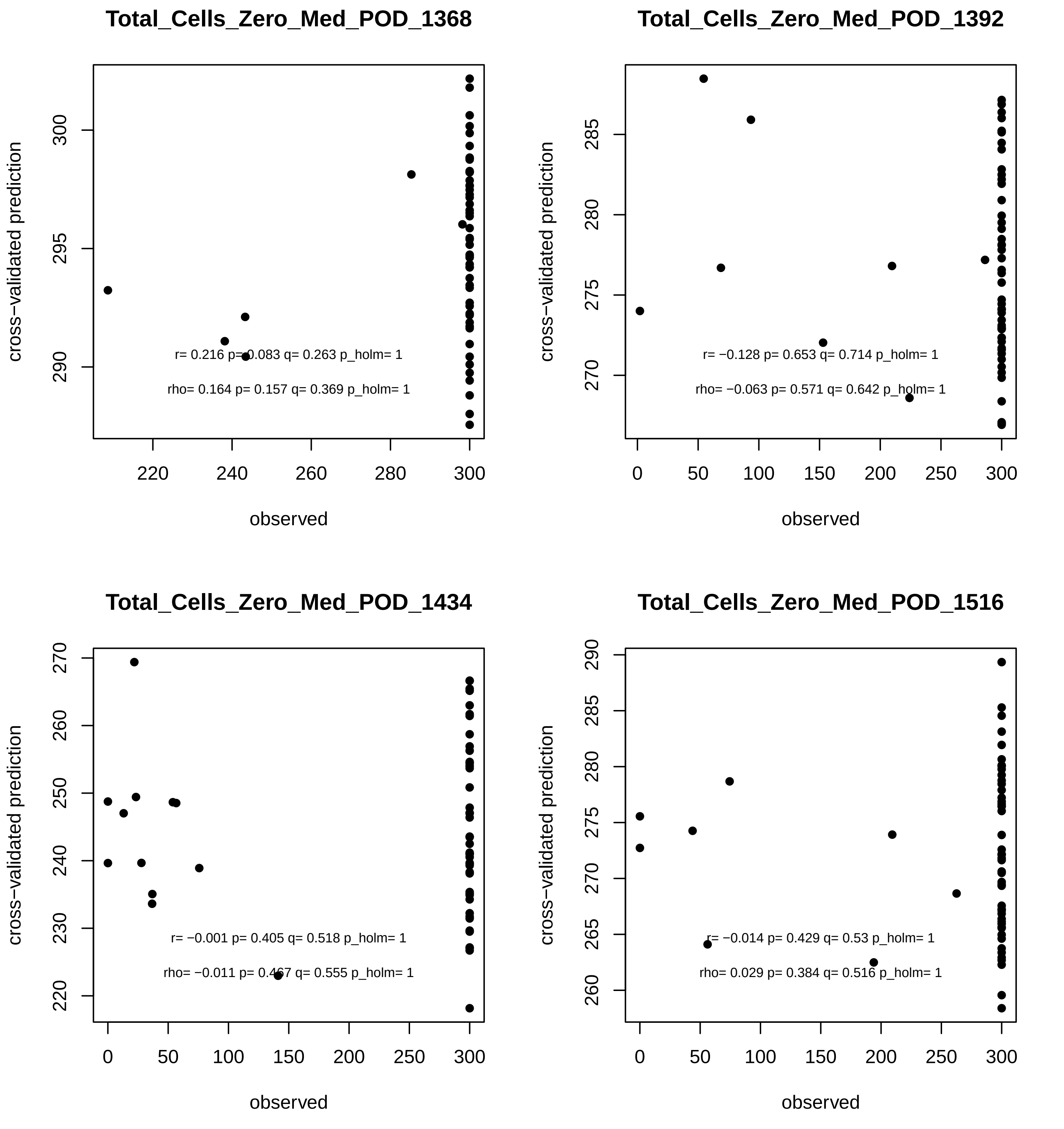

Total_Cells_Zero_Med_POD_1368
r= 0.216 p= 0.083 q= 0.263 p_holm= 1
rho= 0.164 p= 0.157 q= 0.369 p_holm= 1
cross−validated prediction
observed
Total_Cells_Zero_Med_POD_1392
r= −0.128 p= 0.653 q= 0.714 p_holm= 1
rho= −0.063 p= 0.571 q= 0.642 p_holm= 1
cross−validated prediction
observed
Total_Cells_Zero_Med_POD_1434
r= −0.001 p= 0.405 q= 0.518 p_holm= 1
rho= −0.011 p= 0.467 q= 0.555 p_holm= 1
cross−validated prediction
observed
Total_Cells_Zero_Med_POD_1516
r= −0.014 p= 0.429 q= 0.53 p_holm= 1
rho= 0.029 p= 0.384 q= 0.516 p_holm= 1
cross−validated prediction
observed

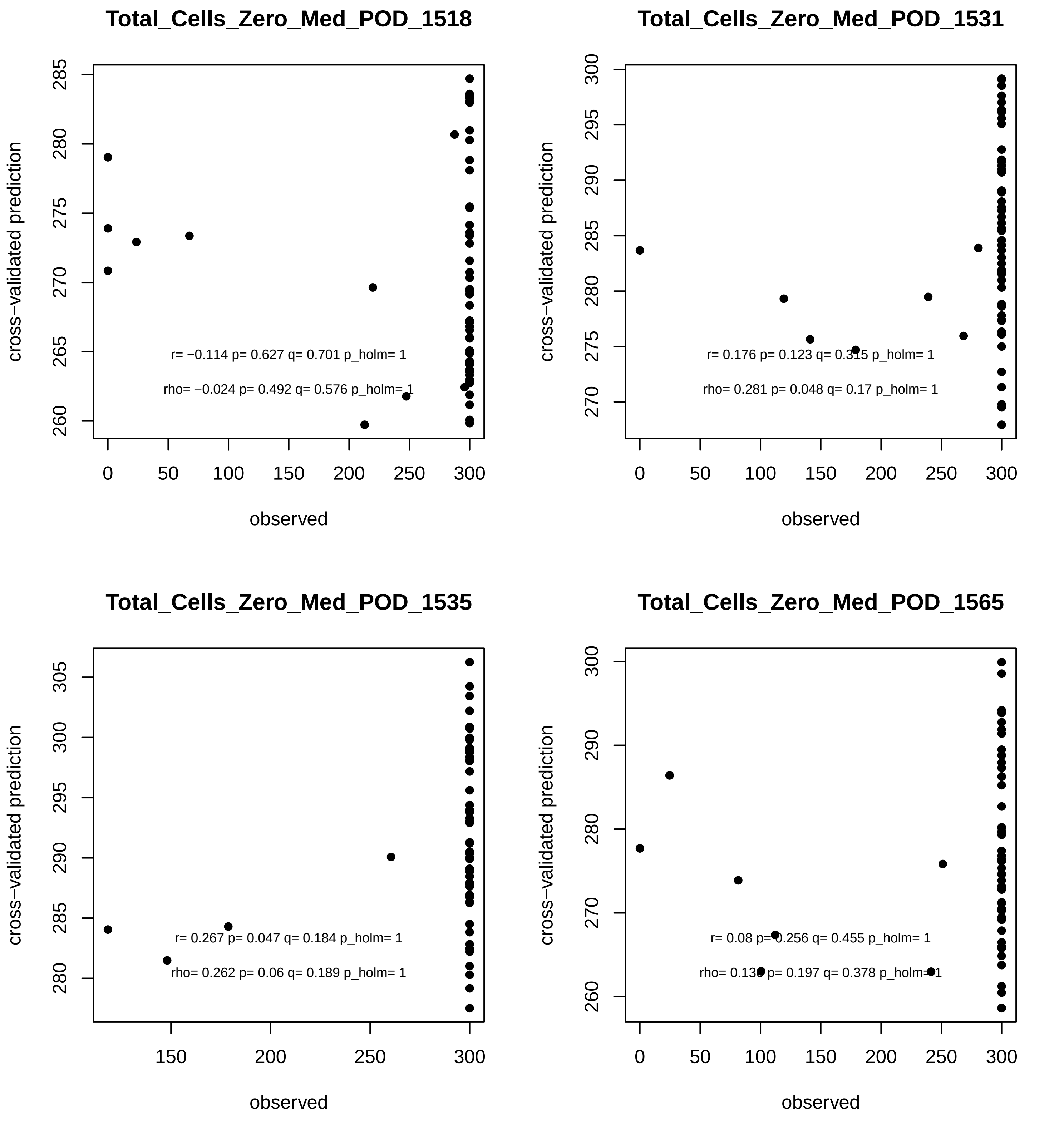
Total_Cells_Zero_Med_POD_1518
r= −0.114 p= 0.627 q= 0.701 p_holm= 1
rho= −0.024 p= 0.492 q= 0.576 p_holm= 1
Total_Cells_Zero_Med_POD_1531
r= 0.176 p= 0.123 q= 0.315 p_holm= 1
rho= 0.281 p= 0.048 q= 0.17 p_holm= 1
Total_Cells_Zero_Med_POD_1535
r= 0.267 p= 0.047 q= 0.184 p_holm= 1
rho= 0.262 p= 0.06 q= 0.189 p_holm= 1
Total_Cells_Zero_Med_POD_1565
r= 0.08 p= 0.256 q= 0.455 p_holm= 1
p= 0.197 q= 0.378 p_holm= 1
cross−validated prediction
observed

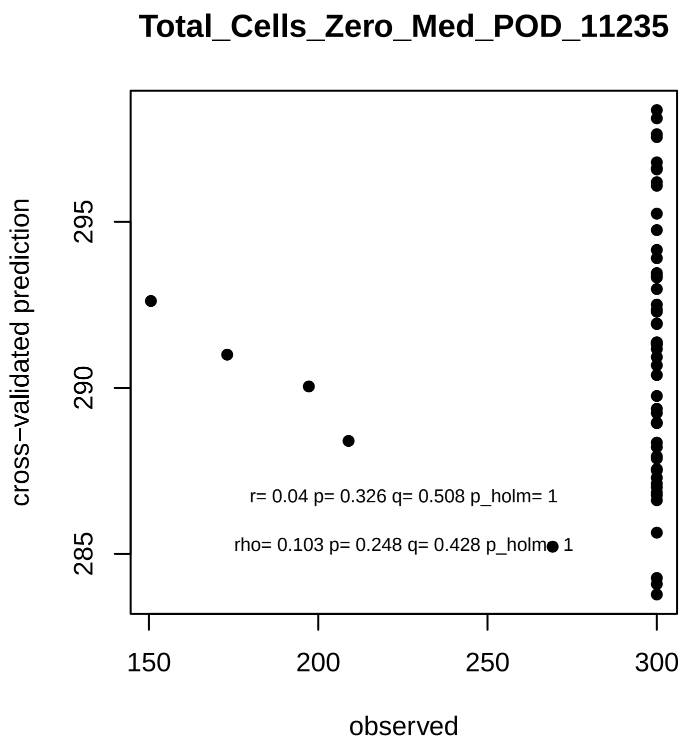
Total_Cells_Zero_Med_POD_11235
cross−validated prediction
285
290
295
r= 0.04 p= 0.326 q= 0.508 p_holm= 1
rho= 0.103 p= 0.248 q= 0.428 p_holm= 1
150
200
250
300
observed

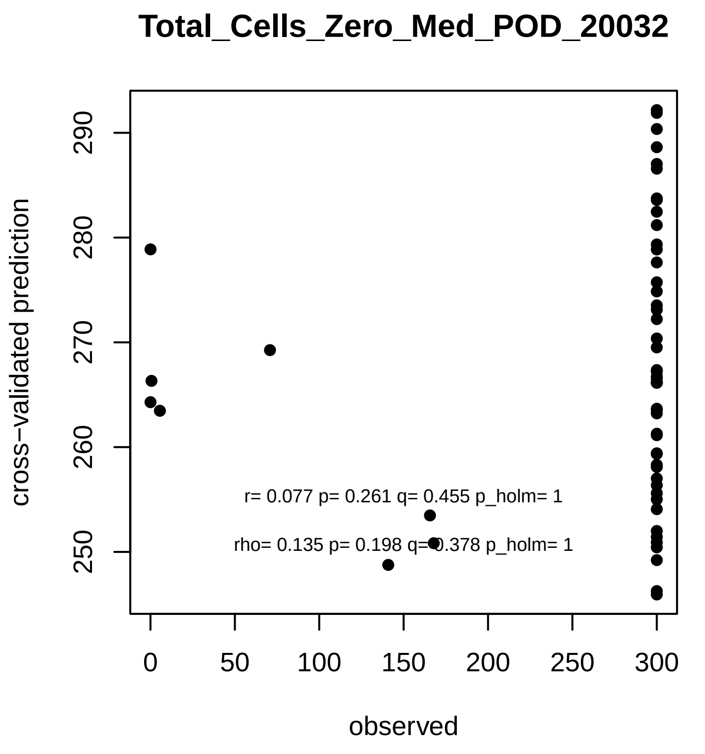
Total_Cells_Zero_Med_POD_20032
cross−validated prediction
250
260
270
280
290
r= 0.077 p= 0.261 q= 0.455 p_holm= 1
rho= 0.135 p= 0.198 q= 0.378 p_holm= 1
0
50
100
150
200
250
300
observed

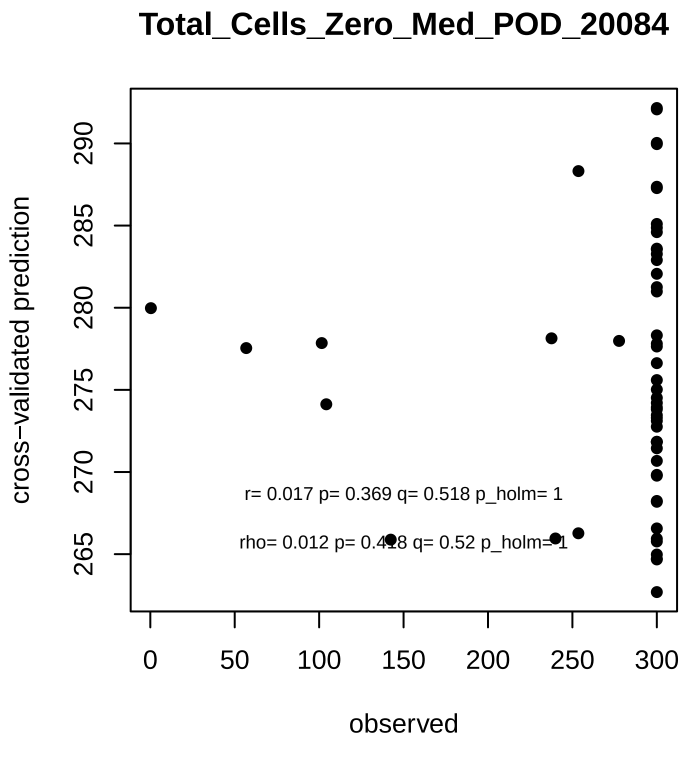
Total_Cells_Zero_Med_POD_20084
cross−validated prediction
265
270
275
280
285
290
r= 0.017 p= 0.369 q= 0.518 p_holm= 1
rho= 0.012 p= 0.418 q= 0.52 p_holm= 1
0
50
100
150
200
250
300
observed

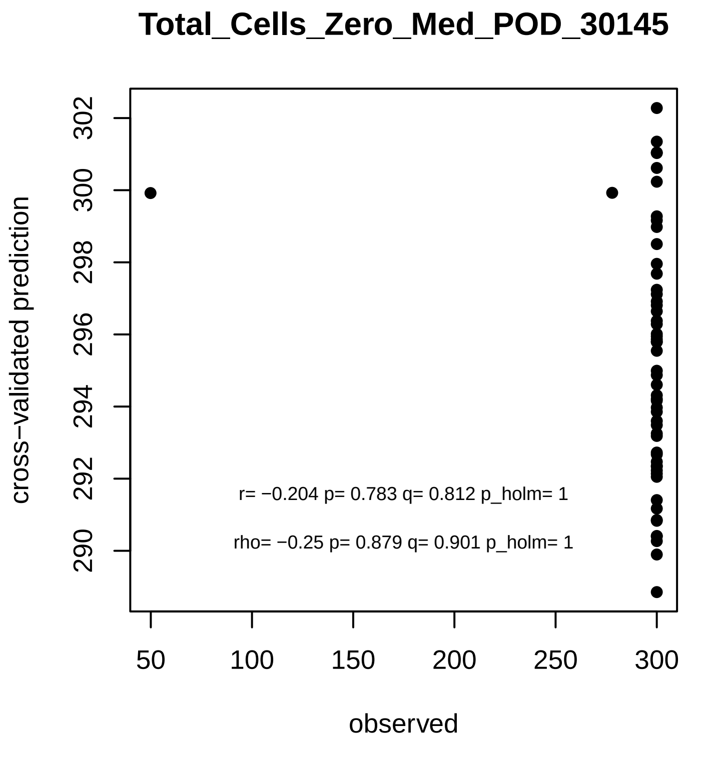
Total_Cells_Zero_Med_POD_30145
cross−validated prediction
290
292
294
296
298
300
302
r= −0.204 p= 0.783 q= 0.812 p_holm= 1
rho= −0.25 p= 0.879 q= 0.901 p_holm= 1
50
100
150
200
250
300
observed

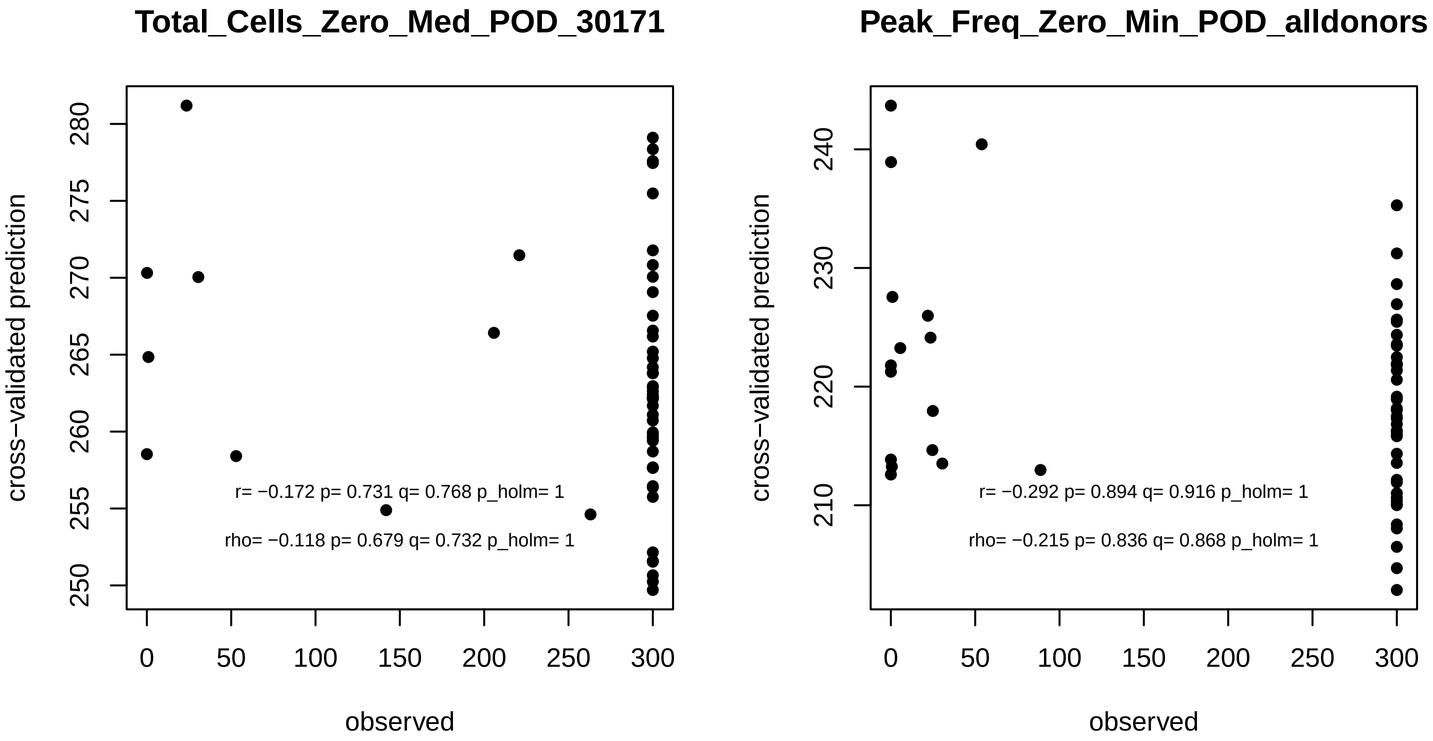

Total_Cells_Zero_Med_POD_30171
cross−validated prediction
observed
r= −0.172 p= 0.731 q= 0.768 p_holm= 1
rho= −0.118 p= 0.679 q= 0.732 p_holm= 1
Peak_Freq_Zero_Min_POD_alldonors
cross−validated prediction
observed
r= −0.292 p= 0.894 q= 0.916 p_holm= 1
rho= −0.215 p= 0.836 q= 0.868 p_holm= 1
