# Supplementary material for: Characterizing PFAS hazards and risks: a human population-based in vitro cardiotoxicity assessment strategy
Source: Hum Genomics. 2024 Sep 2;18:92. doi: 10.1186/s40246-024-00665-x (PMC11368000; doi:10.1186/s40246-024-00665-x)
Supplement: Supplementary file 5 — Supplementary Material 5 [file 40246_2024_665_MOESM5_ESM.pdf]

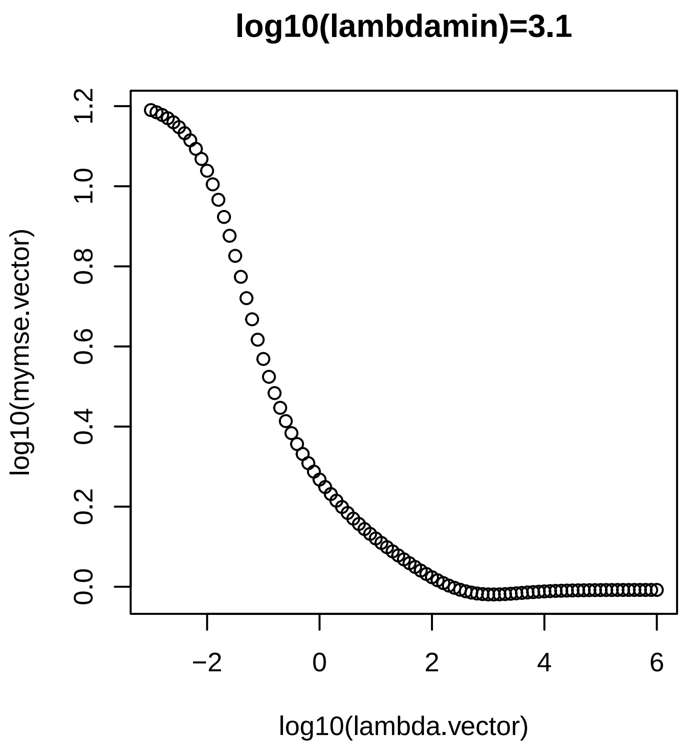

log10(lambdamin)=3.1
log10(mymse.vector)
0.0
0.2
0.4
0.6
0.8
1.0
1.2
−2
0
2
4
6
log10(lambda.vector)

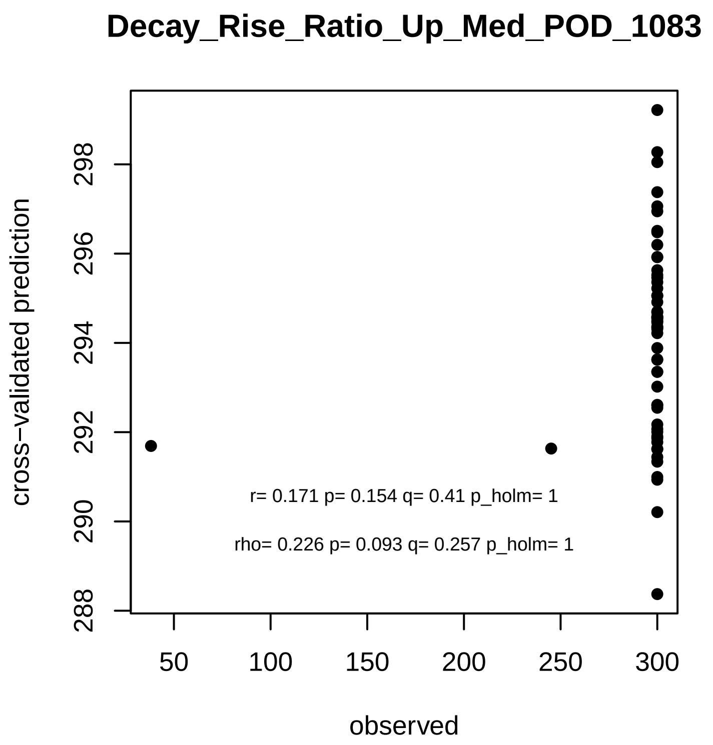

Decay_Rise_Ratio_Up_Med_POD_1083
cross−validated prediction
observed
r= 0.171 p= 0.154 q= 0.41 p_holm= 1
rho= 0.226 p= 0.093 q= 0.257 p_holm= 1

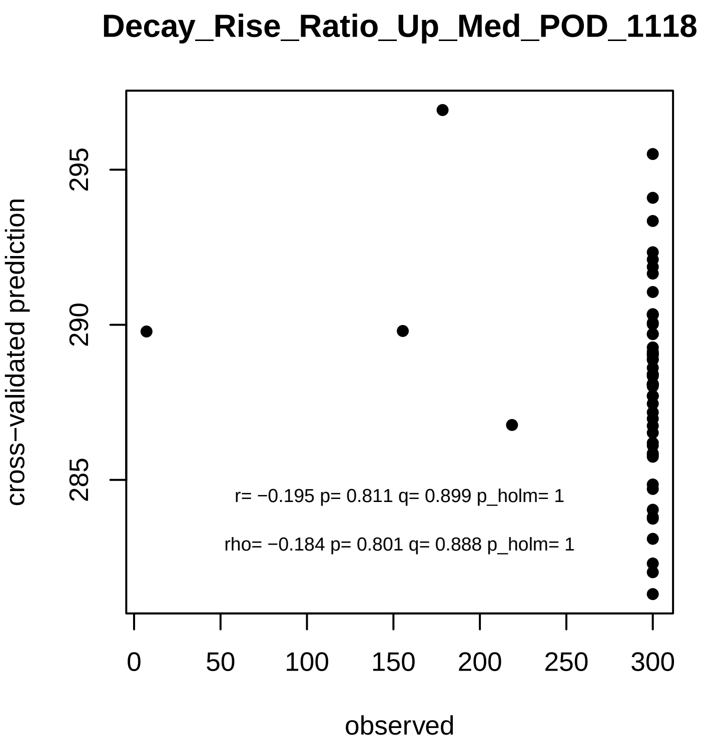

Decay_Rise_Ratio_Up_Med_POD_1118
cross−validated prediction
observed
r= −0.195 p= 0.811 q= 0.899 p_holm= 1
rho= −0.184 p= 0.801 q= 0.888 p_holm= 1

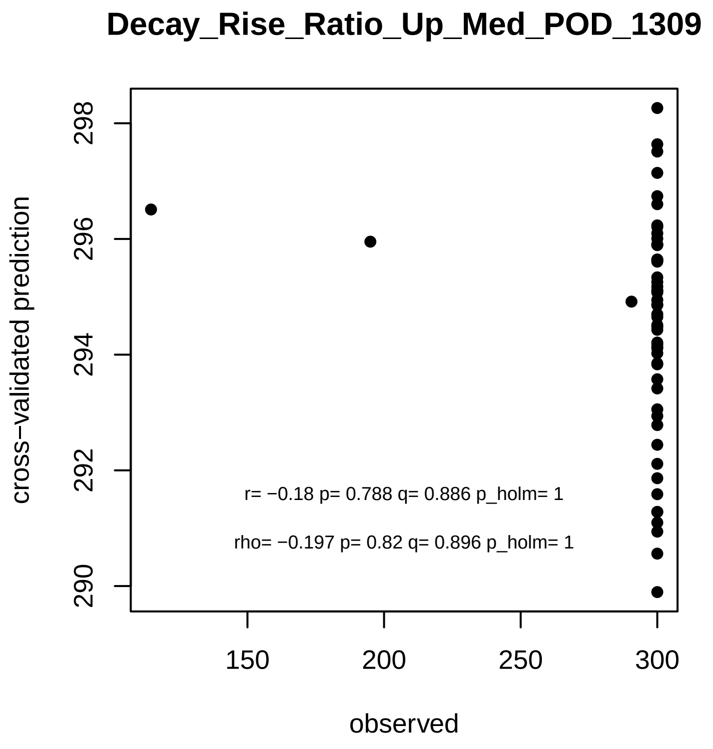

Decay_Rise_Ratio_Up_Med_POD_1309
cross−validated prediction
observed
r= −0.18 p= 0.788 q= 0.886 p_holm= 1
rho= −0.197 p= 0.82 q= 0.896 p_holm= 1

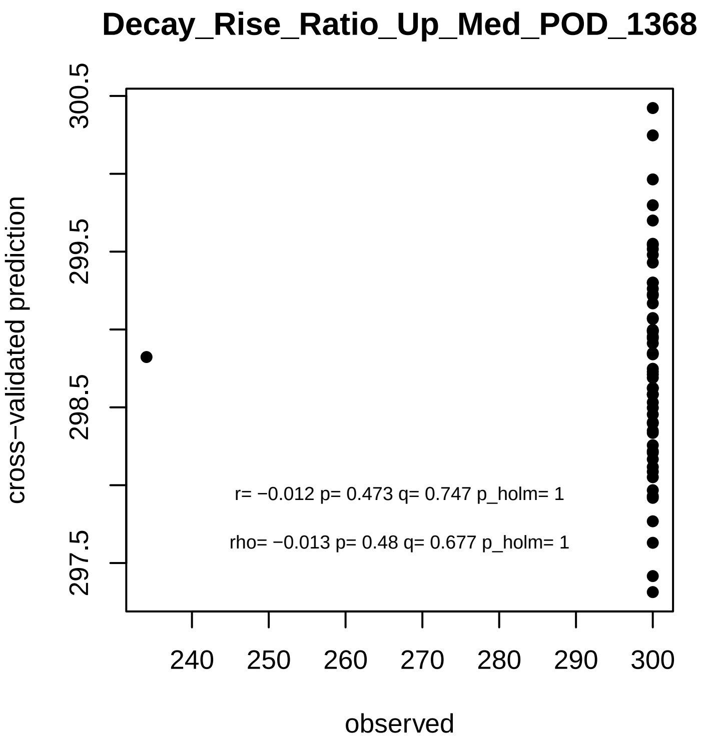

Decay_Rise_Ratio_Up_Med_POD_1368
cross−validated prediction
observed
r= −0.012 p= 0.473 q= 0.747 p_holm= 1
rho= −0.013 p= 0.48 q= 0.677 p_holm= 1

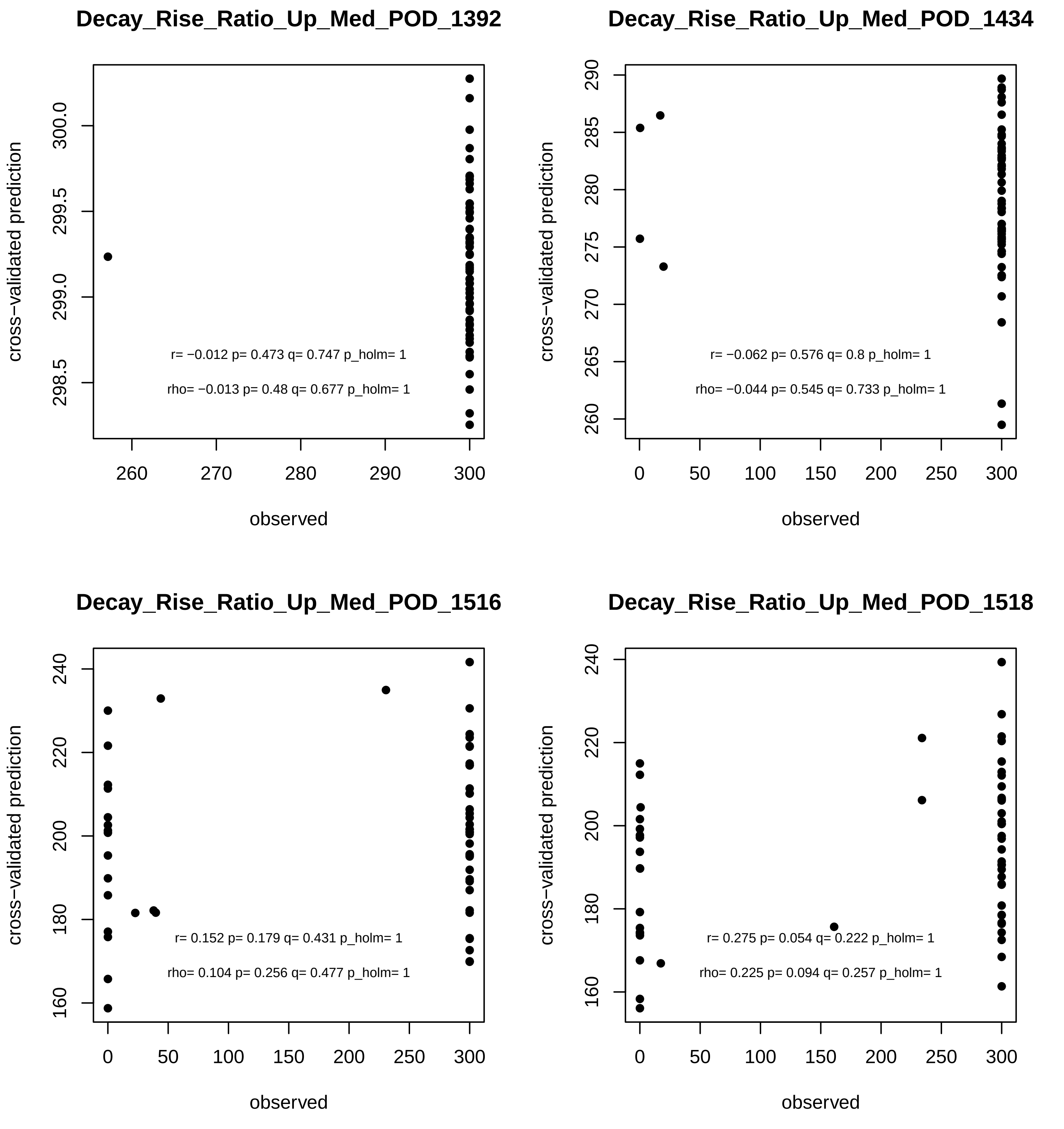
Decay_Rise_Ratio_Up_Med_POD_1392
cross−validated prediction
observed
r= −0.012 p= 0.473 q= 0.747 p_holm= 1
rho= −0.013 p= 0.48 q= 0.677 p_holm= 1
Decay_Rise_Ratio_Up_Med_POD_1434
cross−validated prediction
observed
r= −0.062 p= 0.576 q= 0.8 p_holm= 1
rho= −0.044 p= 0.545 q= 0.733 p_holm= 1
Decay_Rise_Ratio_Up_Med_POD_1516
cross−validated prediction
observed
r= 0.152 p= 0.179 q= 0.431 p_holm= 1
rho= 0.104 p= 0.256 q= 0.477 p_holm= 1
Decay_Rise_Ratio_Up_Med_POD_1518
cross−validated prediction
observed
r= 0.275 p= 0.054 q= 0.222 p_holm= 1
rho= 0.225 p= 0.094 q= 0.257 p_holm= 1

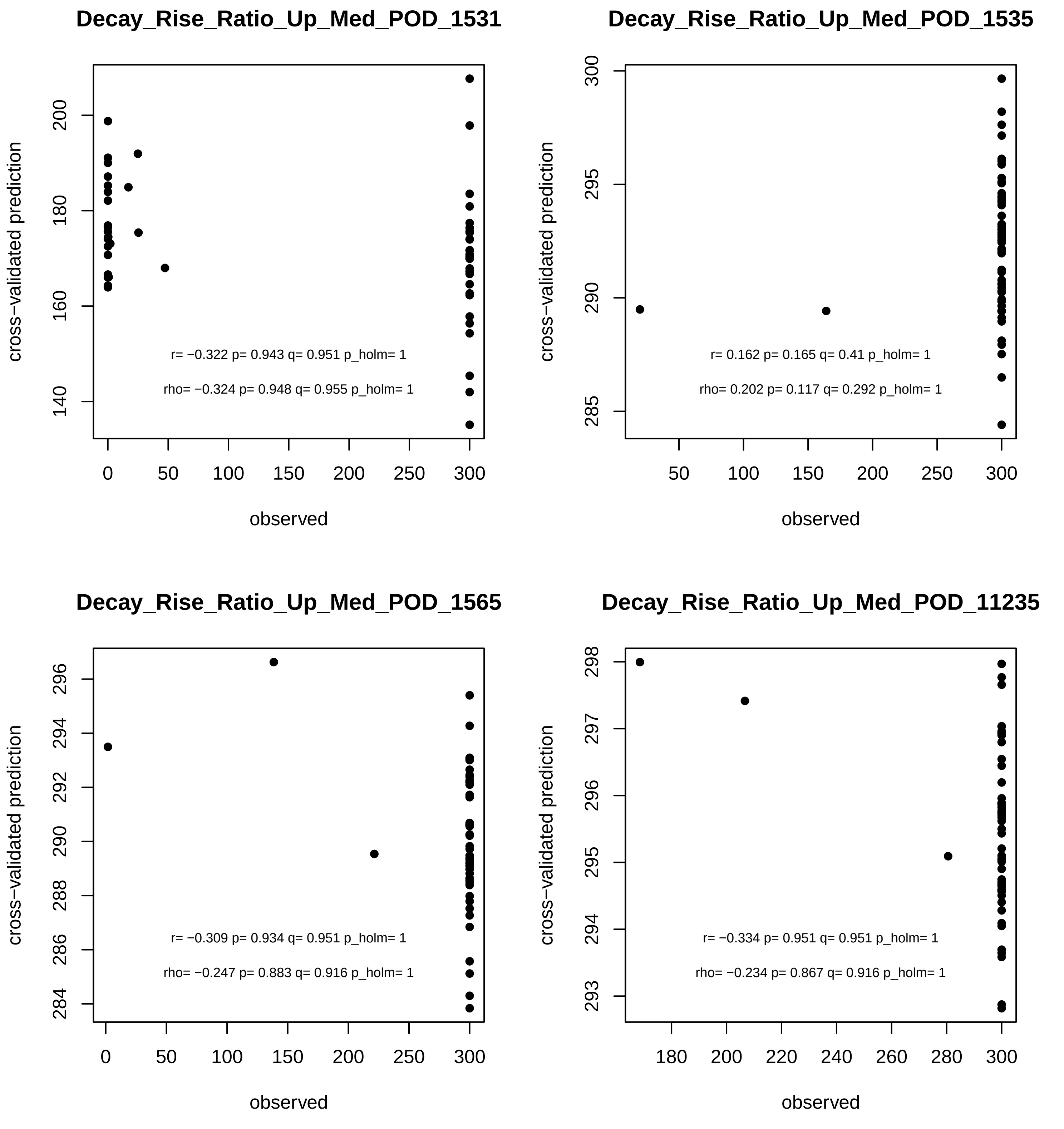
Decay_Rise_Ratio_Up_Med_POD_1531
cross−validated prediction
observed
r= −0.322 p= 0.943 q= 0.951 p_holm= 1
rho= −0.324 p= 0.948 q= 0.955 p_holm= 1
Decay_Rise_Ratio_Up_Med_POD_1535
r= 0.162 p= 0.165 q= 0.41 p_holm= 1
rho= 0.202 p= 0.117 q= 0.292 p_holm= 1
Decay_Rise_Ratio_Up_Med_POD_1565
r= −0.309 p= 0.934 q= 0.951 p_holm= 1
rho= −0.247 p= 0.883 q= 0.916 p_holm= 1
Decay_Rise_Ratio_Up_Med_POD_11235
r= −0.334 p= 0.951 q= 0.951 p_holm= 1
rho= −0.234 p= 0.867 q= 0.916 p_holm= 1

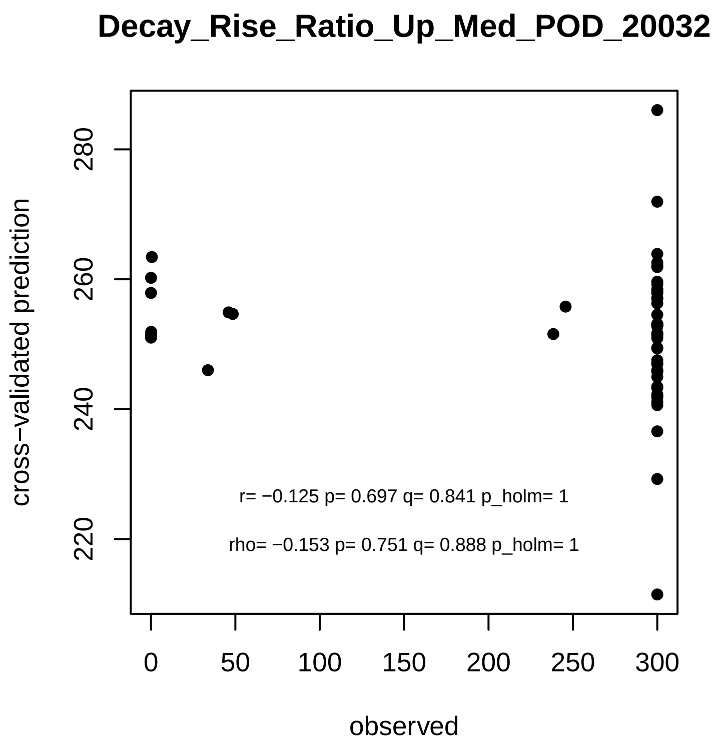
Decay_Rise_Ratio_Up_Med_POD_20032
cross−validated prediction
observed
r= −0.125 p= 0.697 q= 0.841 p_holm= 1
rho= −0.153 p= 0.751 q= 0.888 p_holm= 1

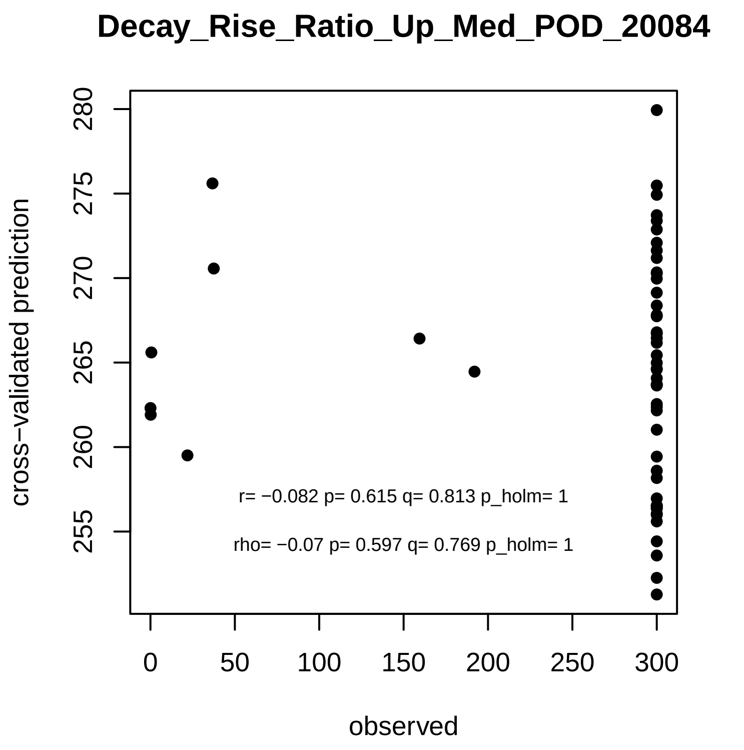
Decay_Rise_Ratio_Up_Med_POD_20084
cross−validated prediction
observed
r= −0.082 p= 0.615 q= 0.813 p_holm= 1
rho= −0.07 p= 0.597 q= 0.769 p_holm= 1

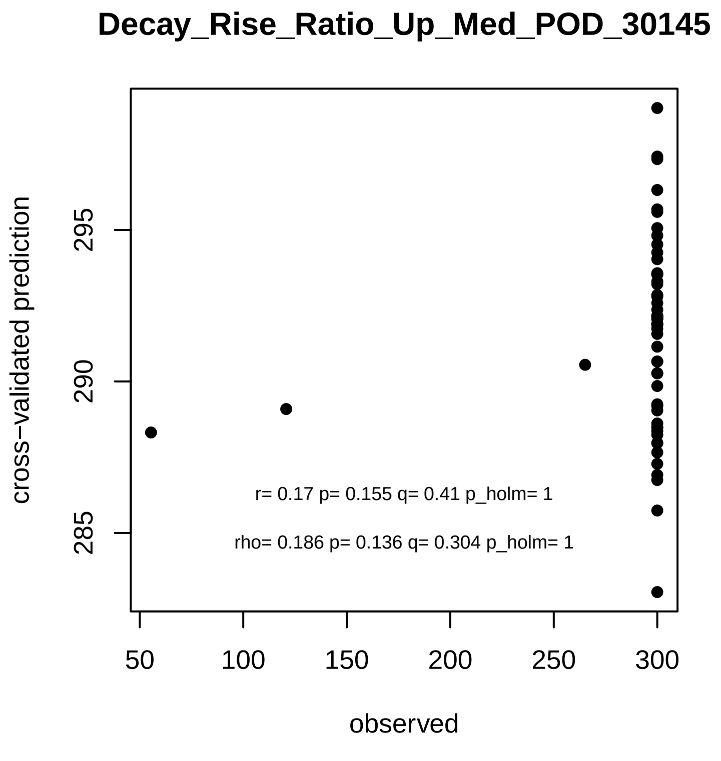
Decay_Rise_Ratio_Up_Med_POD_30145
cross−validated prediction
observed
r= 0.17 p= 0.155 q= 0.41 p_holm= 1
rho= 0.186 p= 0.136 q= 0.304 p_holm= 1

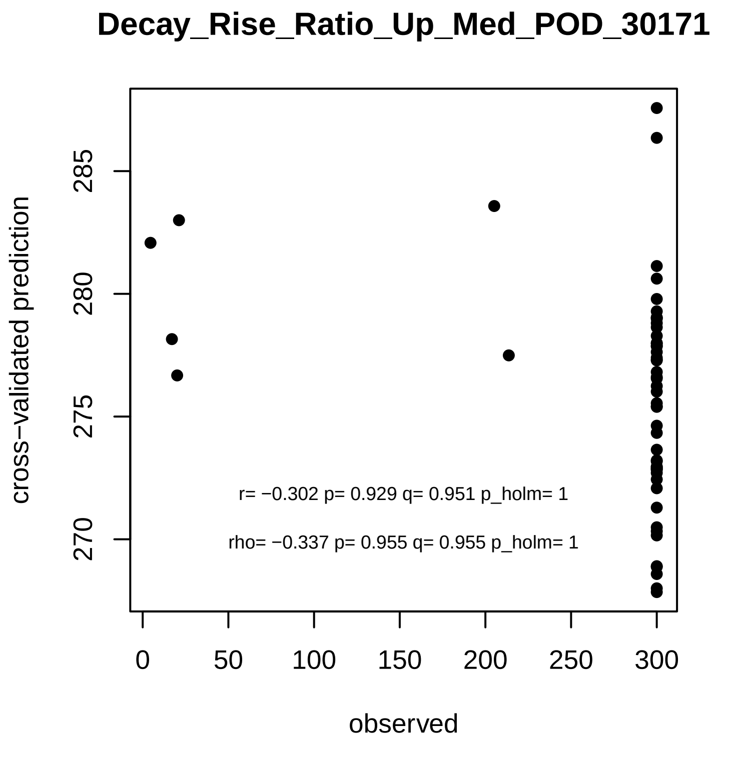
Decay_Rise_Ratio_Up_Med_POD_30171
cross−validated prediction
observed
r= −0.302 p= 0.929 q= 0.951 p_holm= 1
rho= −0.337 p= 0.955 q= 0.955 p_holm= 1

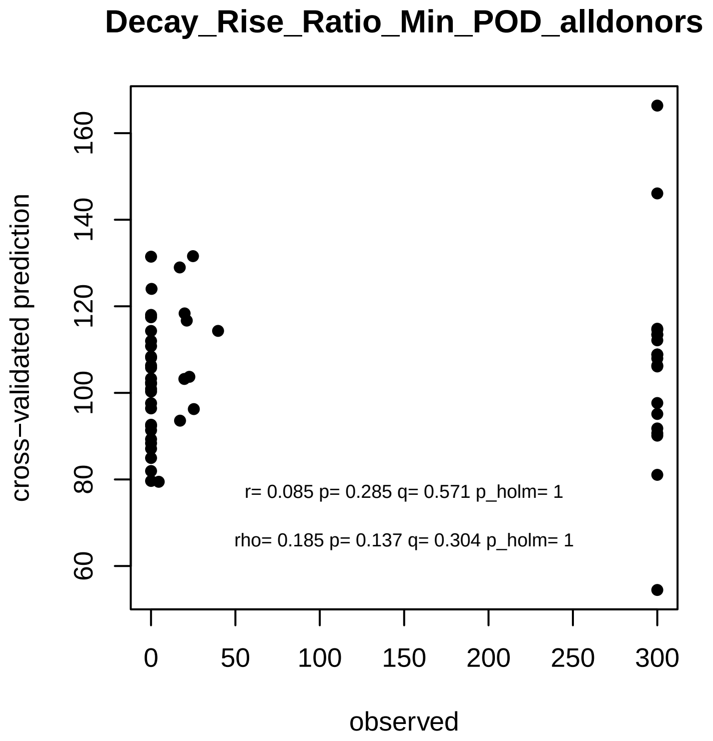
Decay_Rise_Ratio_Min_POD_alldonors
cross−validated prediction
observed
r= 0.085 p= 0.285 q= 0.571 p_holm= 1
rho= 0.185 p= 0.137 q= 0.304 p_holm= 1

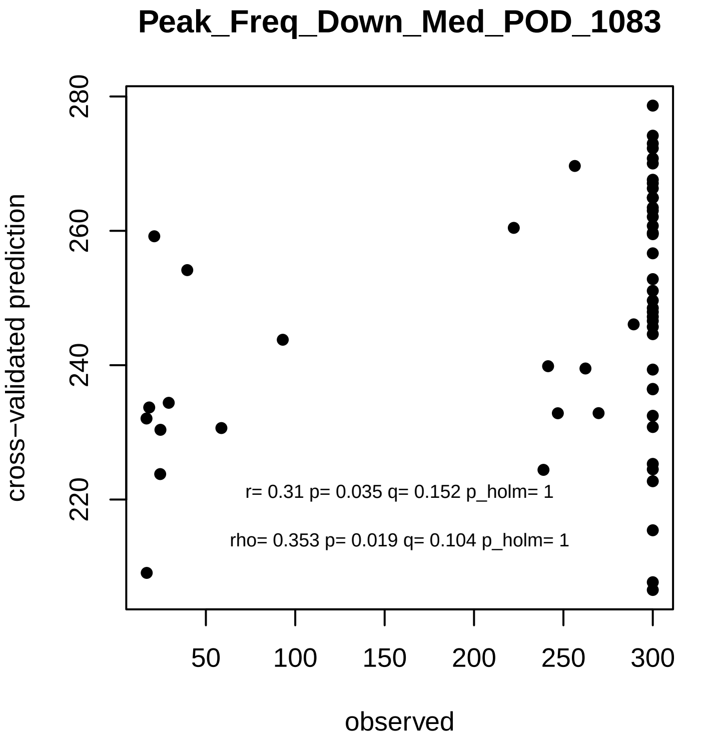
Peak_Freq_Down_Med_POD_1083
cross−validated prediction
observed
r= 0.31 p= 0.035 q= 0.152 p_holm= 1
rho= 0.353 p= 0.019 q= 0.104 p_holm= 1

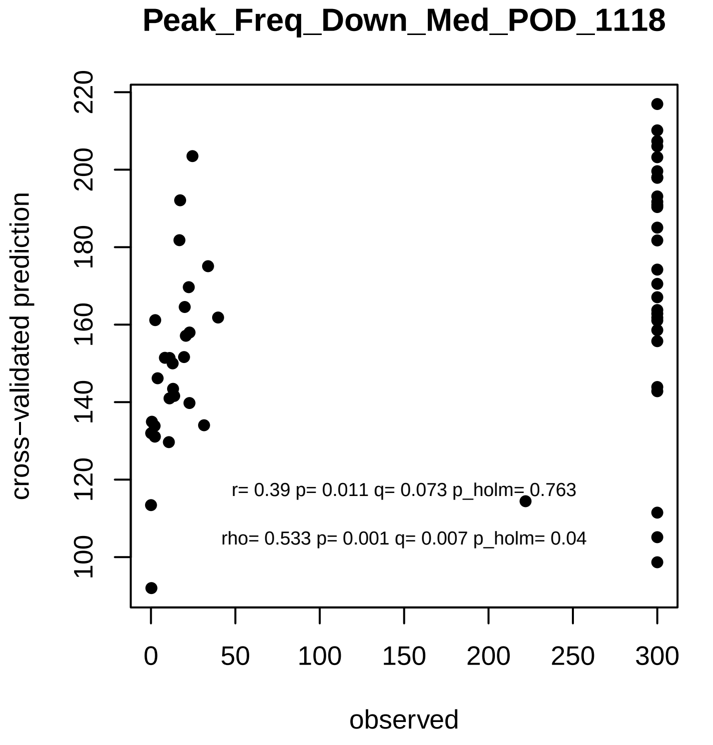
Peak_Freq_Down_Med_POD_1118
cross−validated prediction
observed
r= 0.39 p= 0.011 q= 0.073 p_holm= 0.763
rho= 0.533 p= 0.001 q= 0.007 p_holm= 0.04

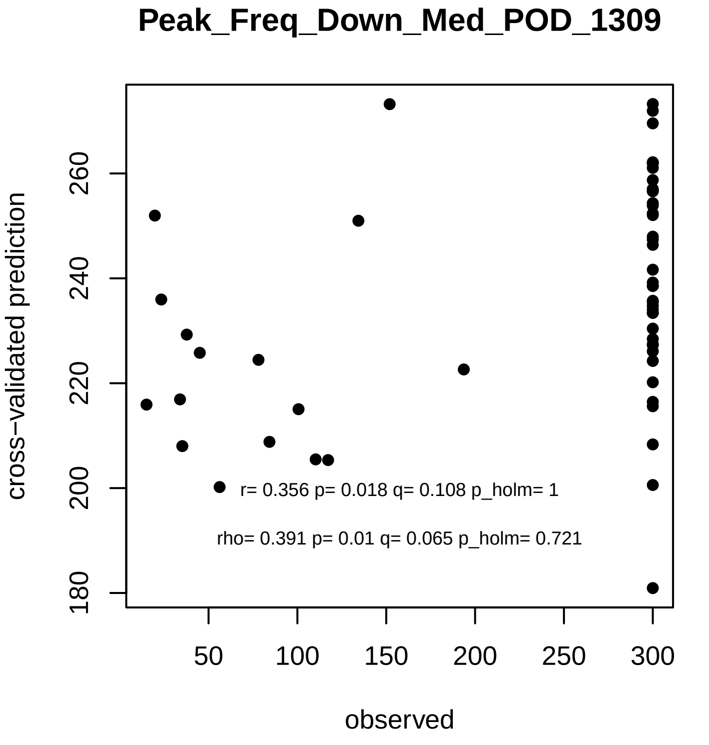
Peak_Freq_Down_Med_POD_1309
cross−validated prediction
observed
r= 0.356 p= 0.018 q= 0.108 p_holm= 1
rho= 0.391 p= 0.01 q= 0.065 p_holm= 0.721

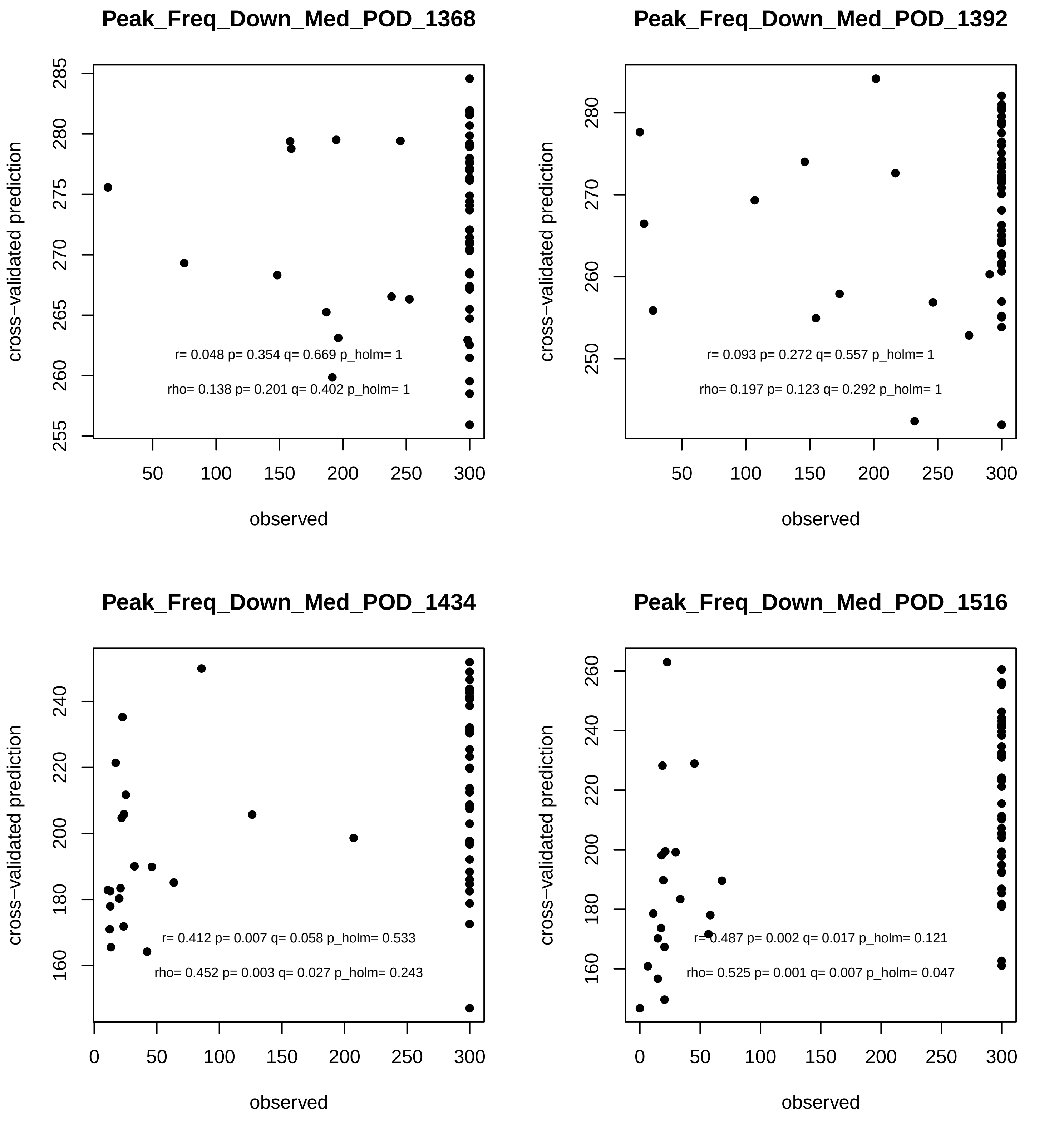
Peak_Freq_Down_Med_POD_1368
r= 0.048 p= 0.354 q= 0.669 p_holm= 1
rho= 0.138 p= 0.201 q= 0.402 p_holm= 1
Peak_Freq_Down_Med_POD_1392
r= 0.093 p= 0.272 q= 0.557 p_holm= 1
rho= 0.197 p= 0.123 q= 0.292 p_holm= 1
Peak_Freq_Down_Med_POD_1434
r= 0.412 p= 0.007 q= 0.058 p_holm= 0.533
rho= 0.452 p= 0.003 q= 0.027 p_holm= 0.243
Peak_Freq_Down_Med_POD_1516
r= 0.487 p= 0.002 q= 0.017 p_holm= 0.121
rho= 0.525 p= 0.001 q= 0.007 p_holm= 0.047
cross−validated prediction
observed

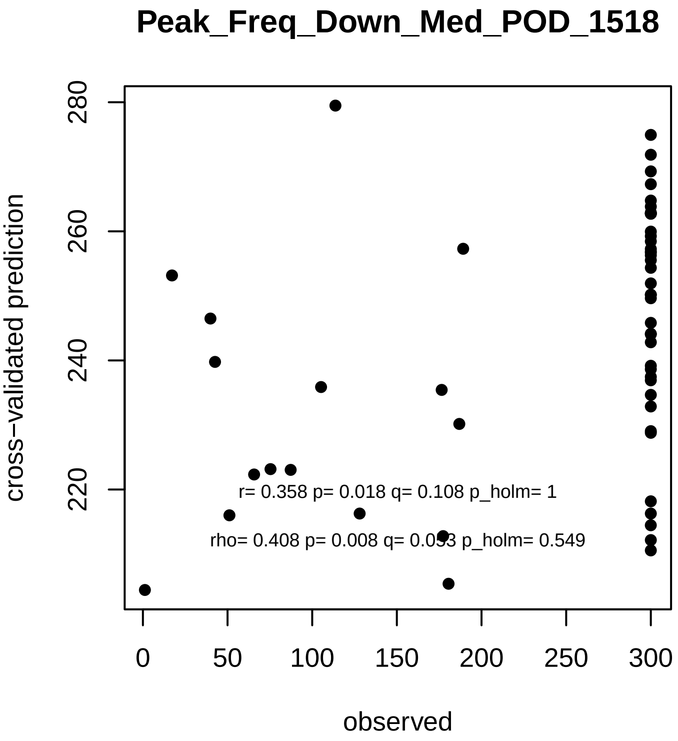
Peak_Freq_Down_Med_POD_1518
cross−validated prediction
280
260
240
220
r= 0.358 p= 0.018 q= 0.108 p_holm= 1
rho= 0.408 p= 0.008
p_holm= 0.549
0
50
100
150
200
250
300
observed

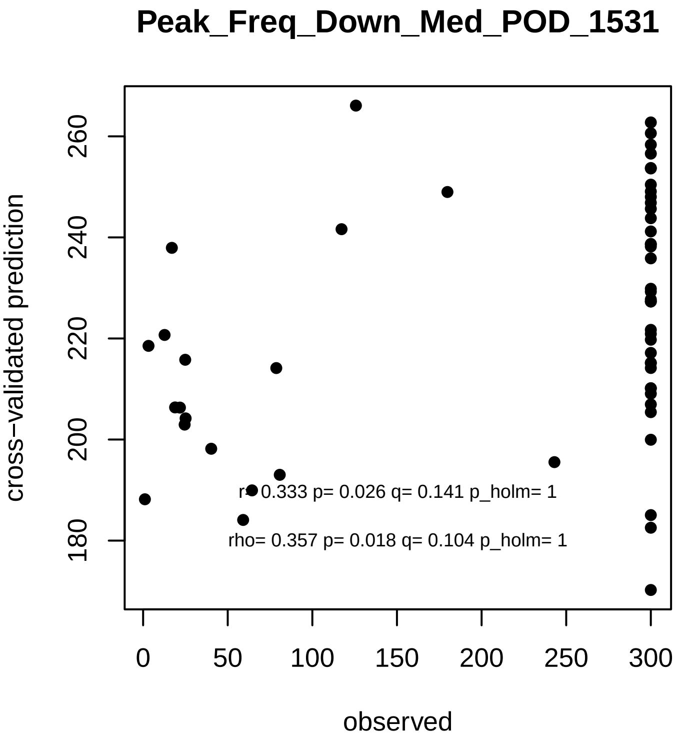
Peak_Freq_Down_Med_POD_1531
cross−validated prediction
260
240
220
200
180
r= 0.333 p= 0.026 q= 0.141 p_holm= 1
rho= 0.357 p= 0.018 q= 0.104 p_holm= 1
0
50
100
150
200
250
300
observed

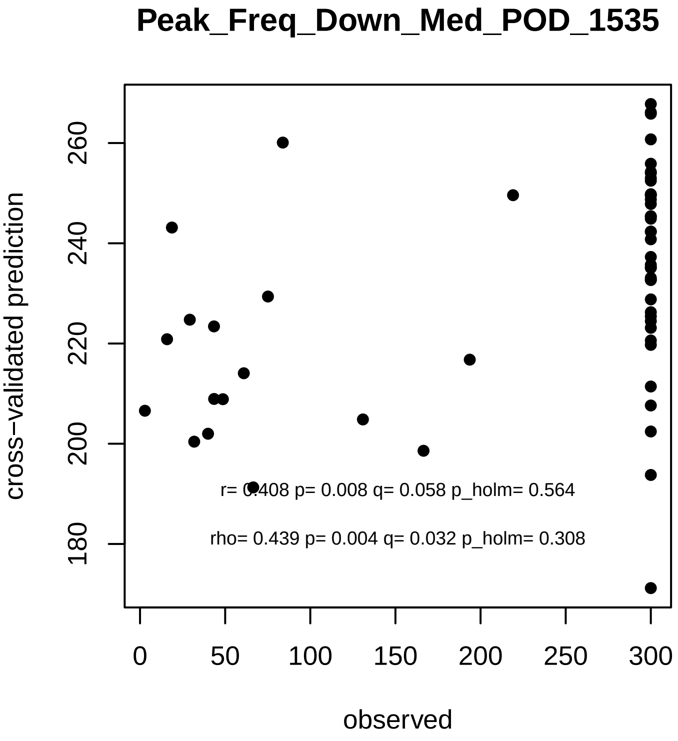
Peak_Freq_Down_Med_POD_1535
cross−validated prediction
260
240
220
200
180
r= 0.408 p= 0.008 q= 0.058 p_holm= 0.564
rho= 0.439 p= 0.004 q= 0.032 p_holm= 0.308
0
50
100
150
200
250
300
observed

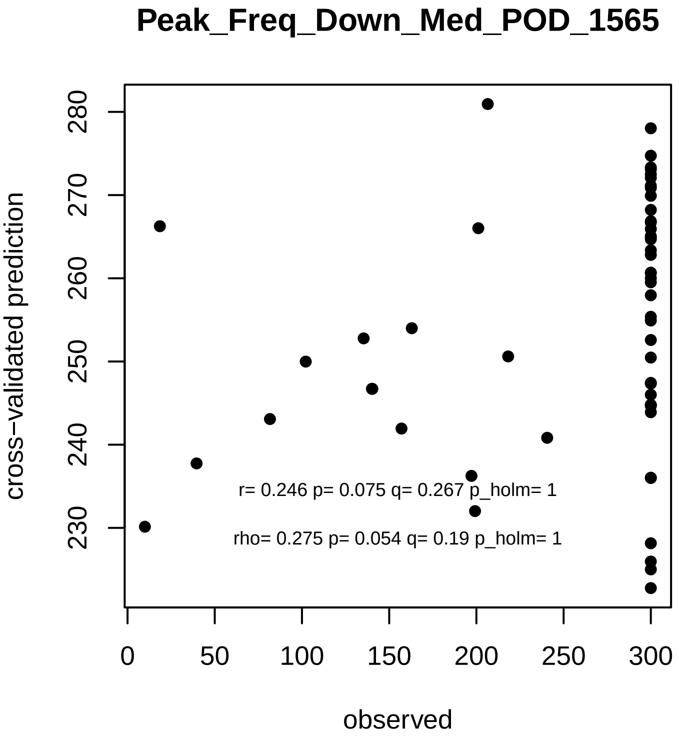
Peak_Freq_Down_Med_POD_1565
cross−validated prediction
280
270
260
250
240
230
r= 0.246 p= 0.075 q= 0.267 p_holm= 1
rho= 0.275 p= 0.054 q= 0.19 p_holm= 1
0
50
100
150
200
250
300
observed

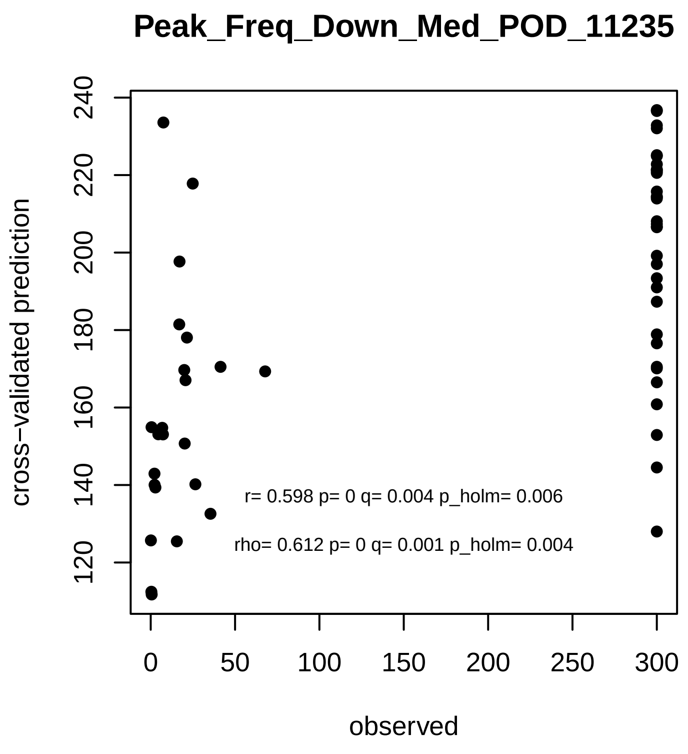
Peak_Freq_Down_Med_POD_11235
r= 0.598 p= 0 q= 0.004 p_holm= 0.006
rho= 0.612 p= 0 q= 0.001 p_holm= 0.004
cross−validated prediction
observed

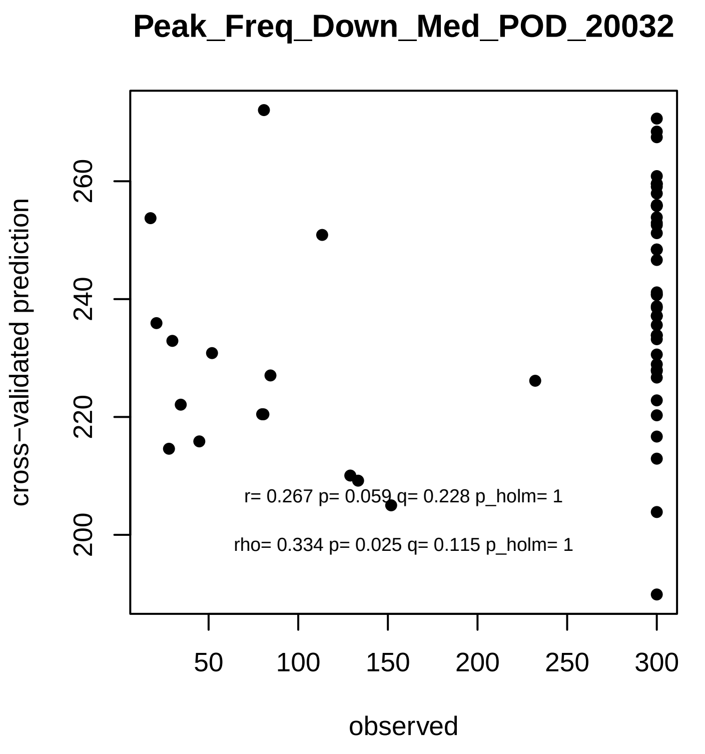
Peak_Freq_Down_Med_POD_20032
r= 0.267 p= 0.059 q= 0.228 p_holm= 1
rho= 0.334 p= 0.025 q= 0.115 p_holm= 1
cross−validated prediction
observed

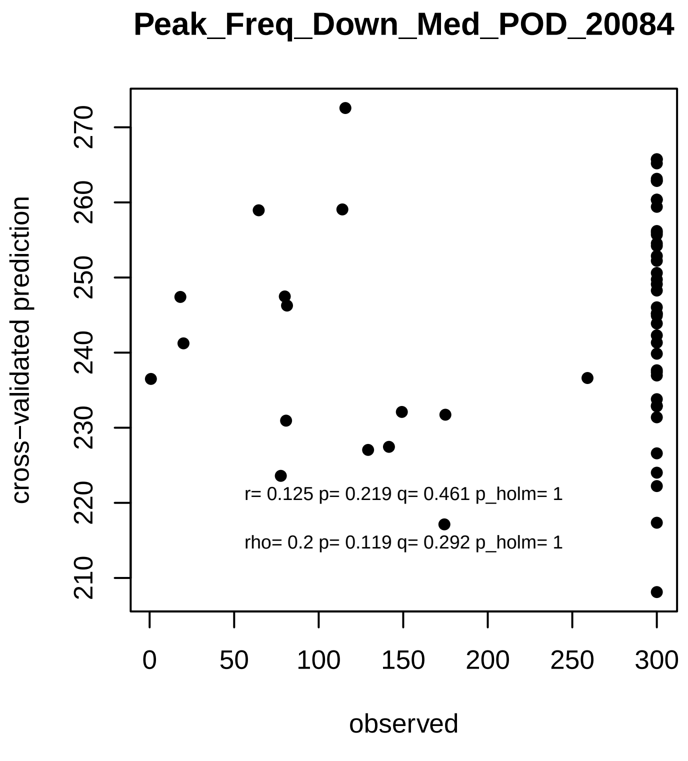
Peak_Freq_Down_Med_POD_20084
r= 0.125 p= 0.219 q= 0.461 p_holm= 1
rho= 0.2 p= 0.119 q= 0.292 p_holm= 1
cross−validated prediction
observed

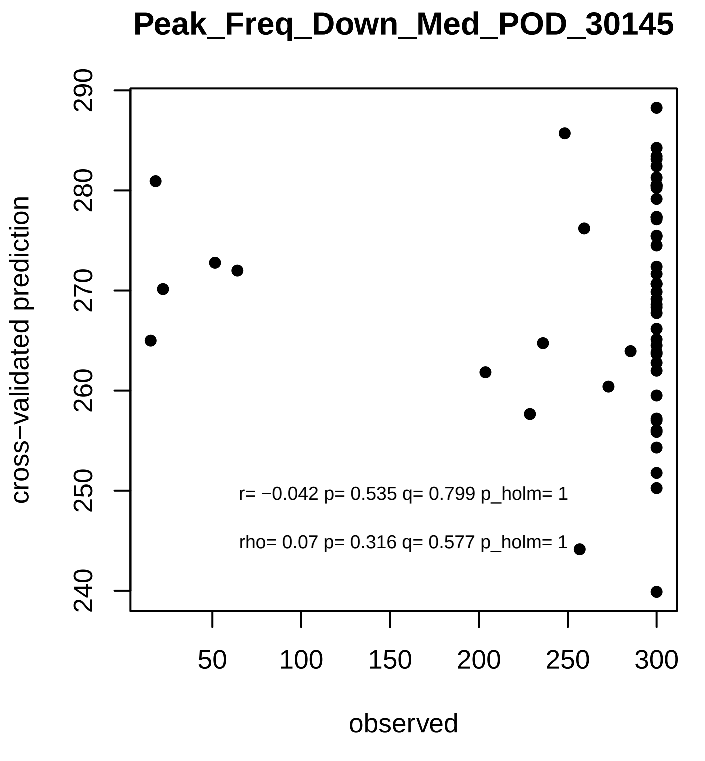
Peak_Freq_Down_Med_POD_30145
r= −0.042 p= 0.535 q= 0.799 p_holm= 1
rho= 0.07 p= 0.316 q= 0.577 p_holm= 1
cross−validated prediction
observed

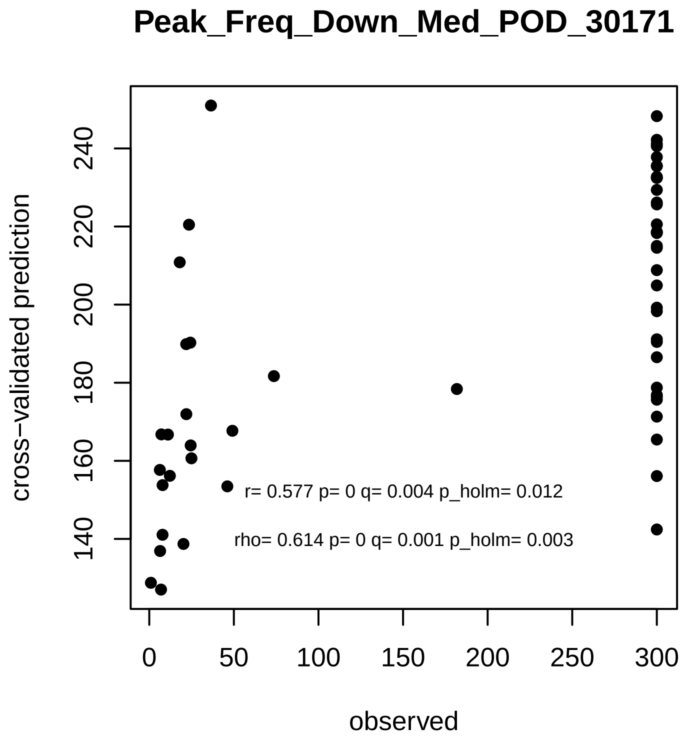
Peak_Freq_Down_Med_POD_30171
r= 0.577 p= 0 q= 0.004 p_holm= 0.012
rho= 0.614 p= 0 q= 0.001 p_holm= 0.003
cross−validated prediction
observed

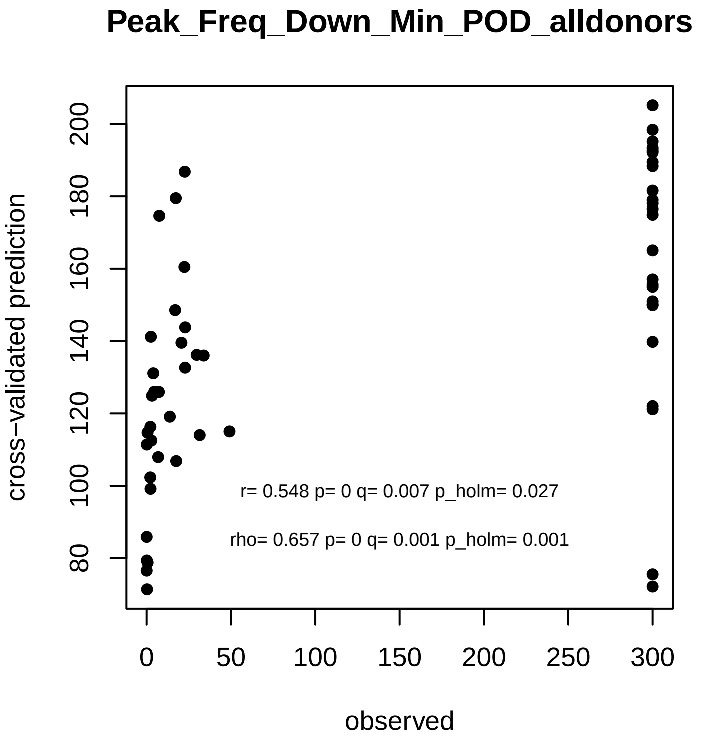
Peak_Freq_Down_Min_POD_alldonors
r= 0.548 p= 0 q= 0.007 p_holm= 0.027
rho= 0.657 p= 0 q= 0.001 p_holm= 0.001
cross−validated prediction
observed

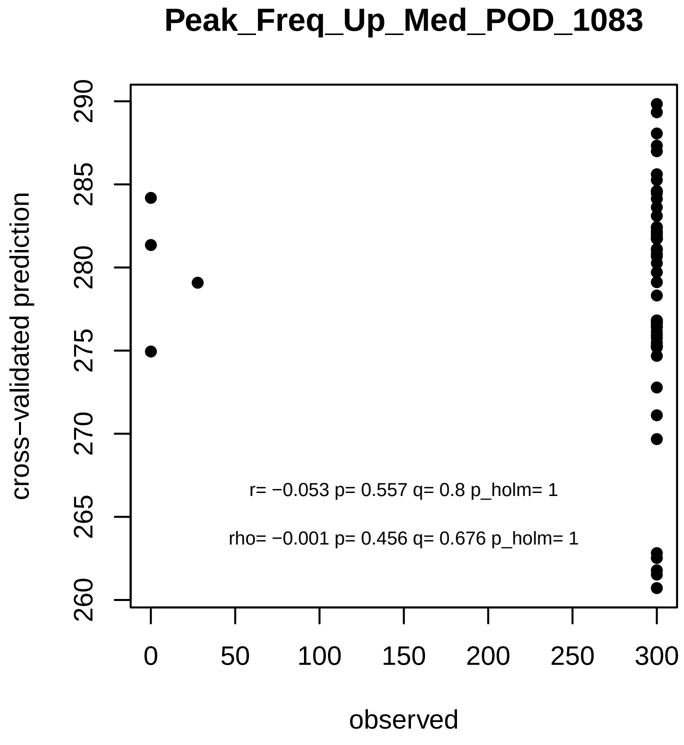
Peak_Freq_Up_Med_POD_1083
r= −0.053 p= 0.557 q= 0.8 p_holm= 1
rho= −0.001 p= 0.456 q= 0.676 p_holm= 1
cross−validated prediction
observed

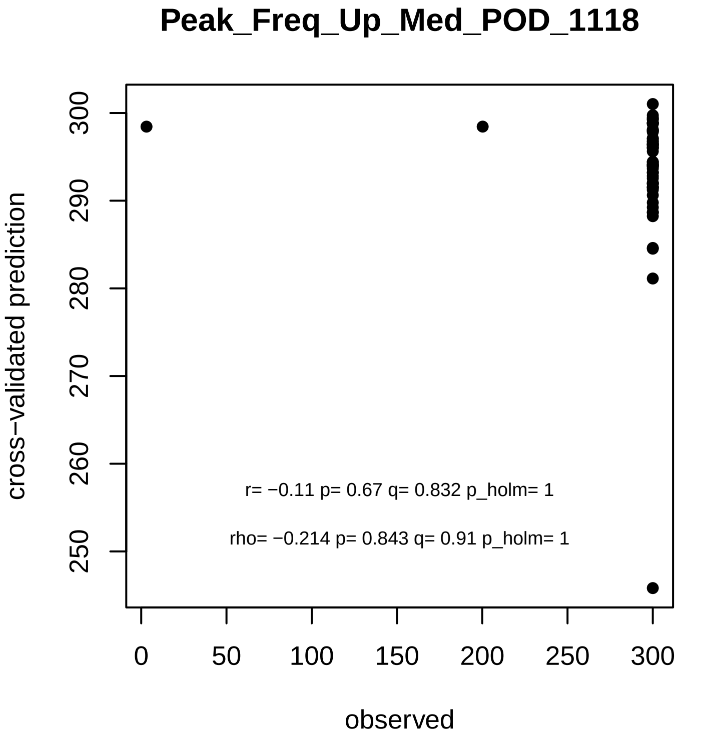
Peak_Freq_Up_Med_POD_1118
r= −0.11 p= 0.67 q= 0.832 p_holm= 1
rho= −0.214 p= 0.843 q= 0.91 p_holm= 1
cross−validated prediction
observed

Peak_Freq_Up_Med_POD_1309

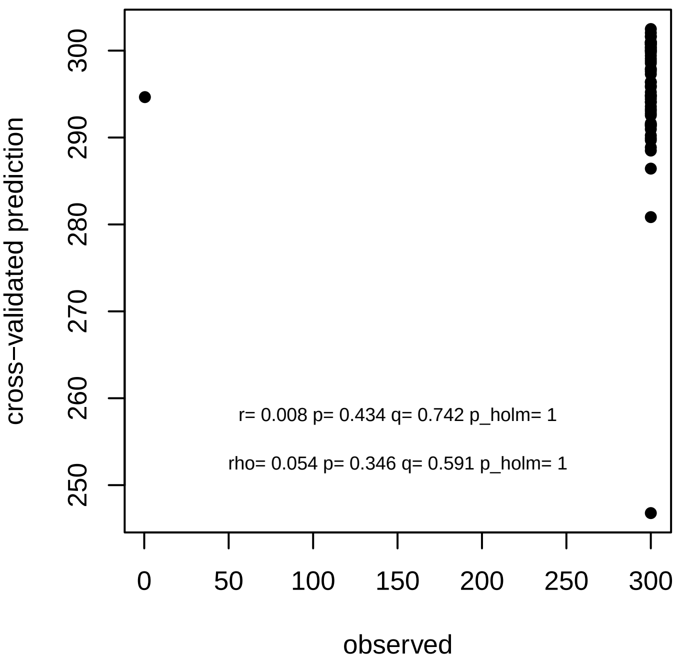

Peak_Freq_Up_Med_POD_1368

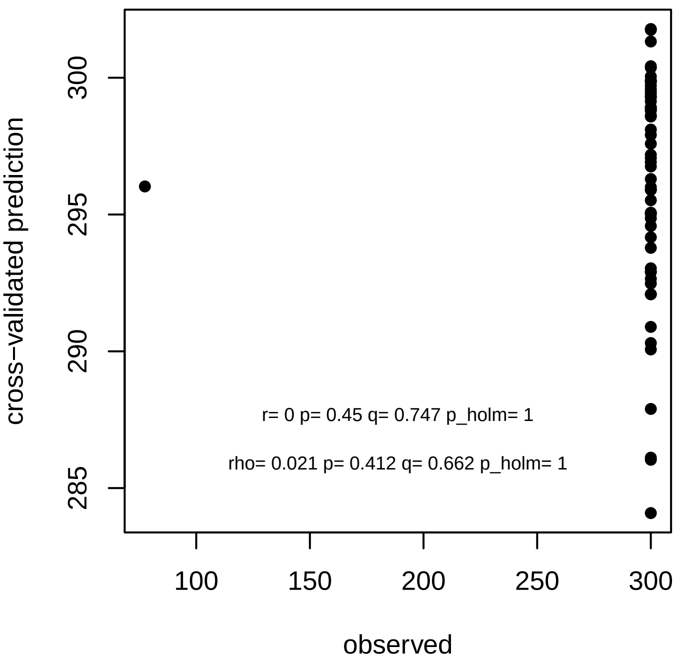

Peak_Freq_Up_Med_POD_1392

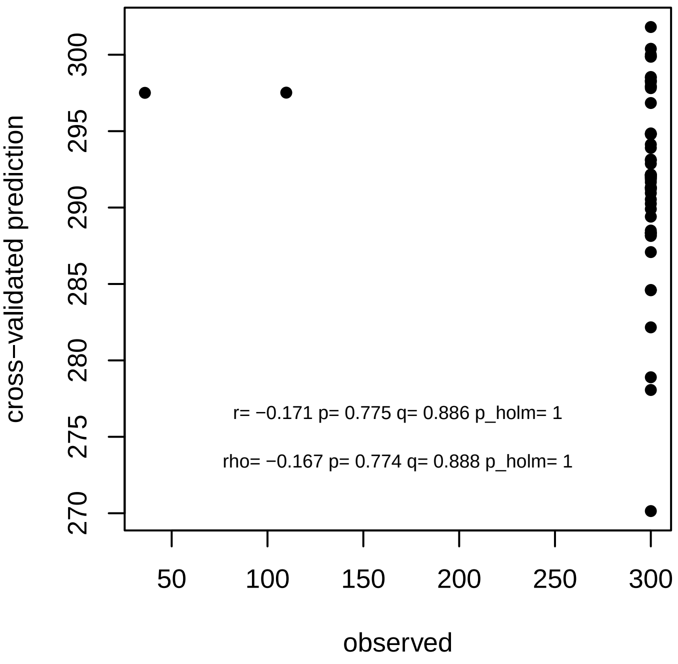

Peak_Freq_Up_Med_POD_1434

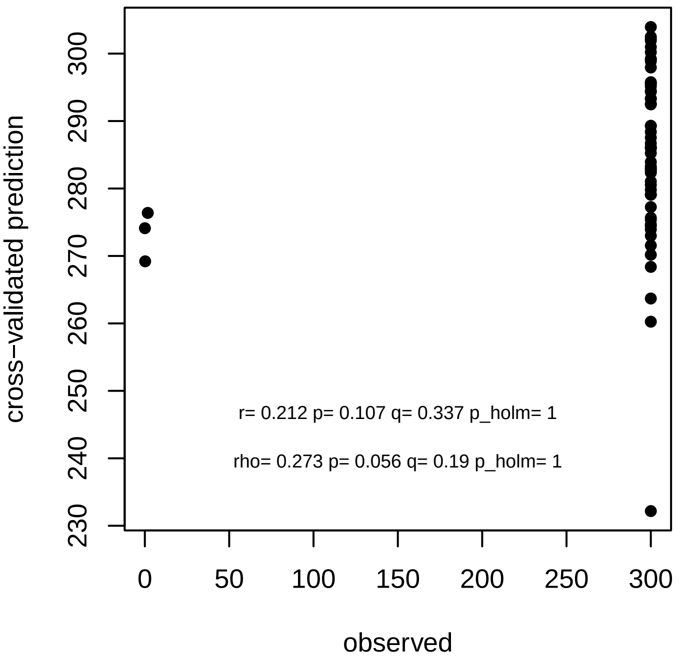

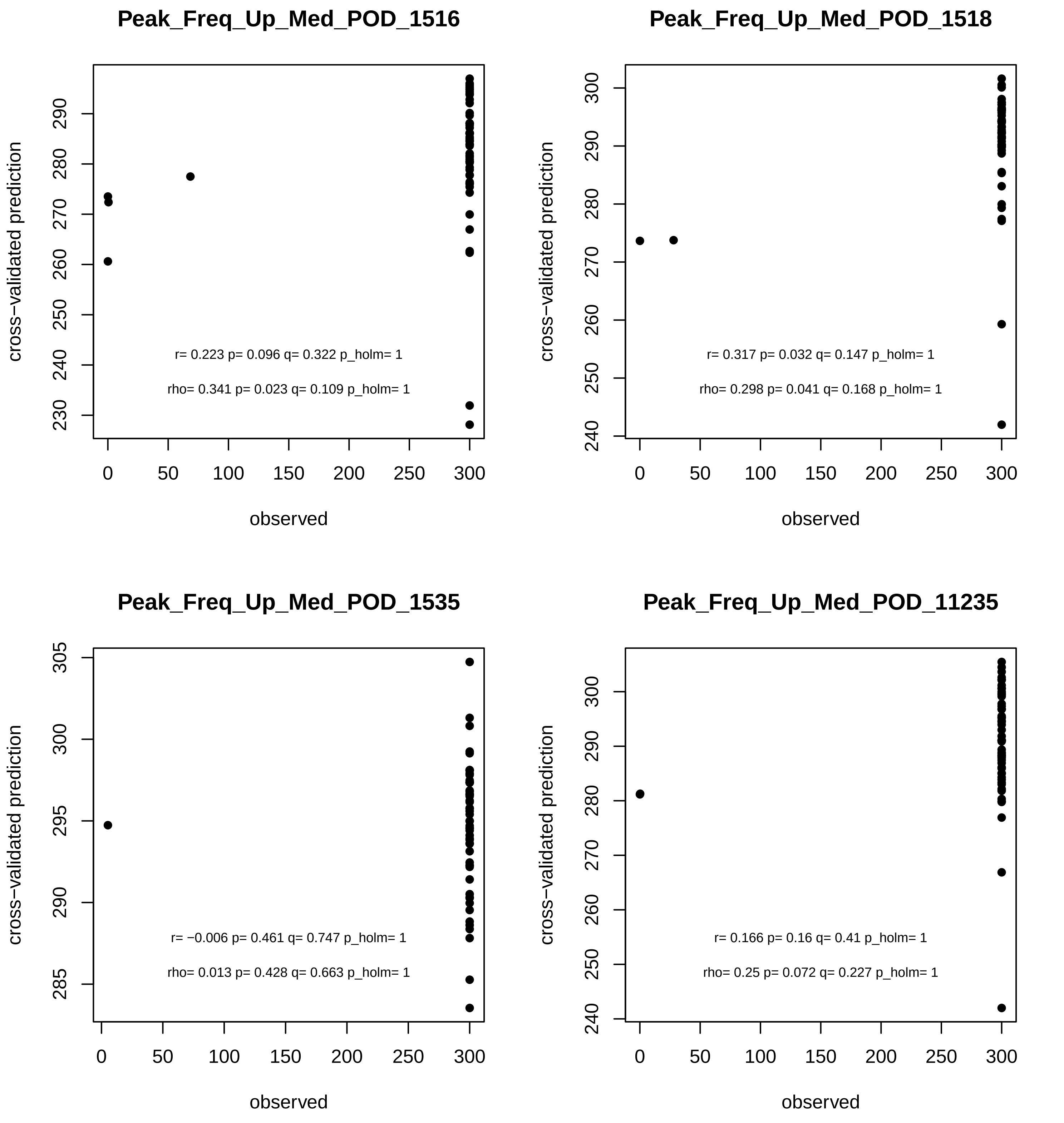
Peak_Freq_Up_Med_POD_1516
r= 0.223 p= 0.096 q= 0.322 p_holm= 1
rho= 0.341 p= 0.023 q= 0.109 p_holm= 1
cross−validated prediction
observed
Peak_Freq_Up_Med_POD_1518
r= 0.317 p= 0.032 q= 0.147 p_holm= 1
rho= 0.298 p= 0.041 q= 0.168 p_holm= 1
cross−validated prediction
observed
Peak_Freq_Up_Med_POD_1535
r= −0.006 p= 0.461 q= 0.747 p_holm= 1
rho= 0.013 p= 0.428 q= 0.663 p_holm= 1
cross−validated prediction
observed
Peak_Freq_Up_Med_POD_11235
r= 0.166 p= 0.16 q= 0.41 p_holm= 1
rho= 0.25 p= 0.072 q= 0.227 p_holm= 1
cross−validated prediction
observed

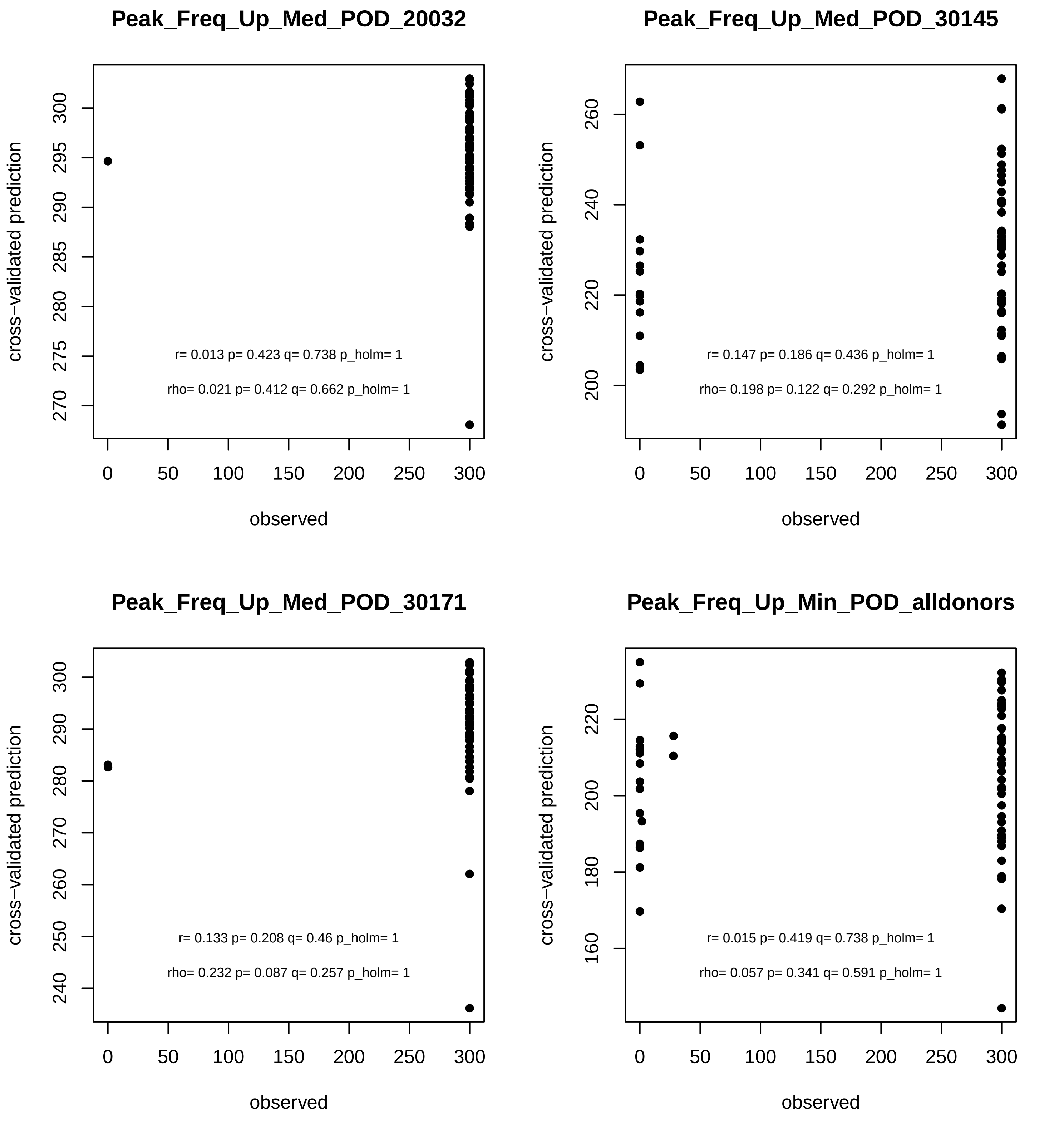
Peak_Freq_Up_Med_POD_20032
r= 0.013 p= 0.423 q= 0.738 p_holm= 1
rho= 0.021 p= 0.412 q= 0.662 p_holm= 1
cross−validated prediction
observed
Peak_Freq_Up_Med_POD_30145
r= 0.147 p= 0.186 q= 0.436 p_holm= 1
rho= 0.198 p= 0.122 q= 0.292 p_holm= 1
cross−validated prediction
observed
Peak_Freq_Up_Med_POD_30171
r= 0.133 p= 0.208 q= 0.46 p_holm= 1
rho= 0.232 p= 0.087 q= 0.257 p_holm= 1
cross−validated prediction
observed
Peak_Freq_Up_Min_POD_alldonors
r= 0.015 p= 0.419 q= 0.738 p_holm= 1
rho= 0.057 p= 0.341 q= 0.591 p_holm= 1
cross−validated prediction
observed

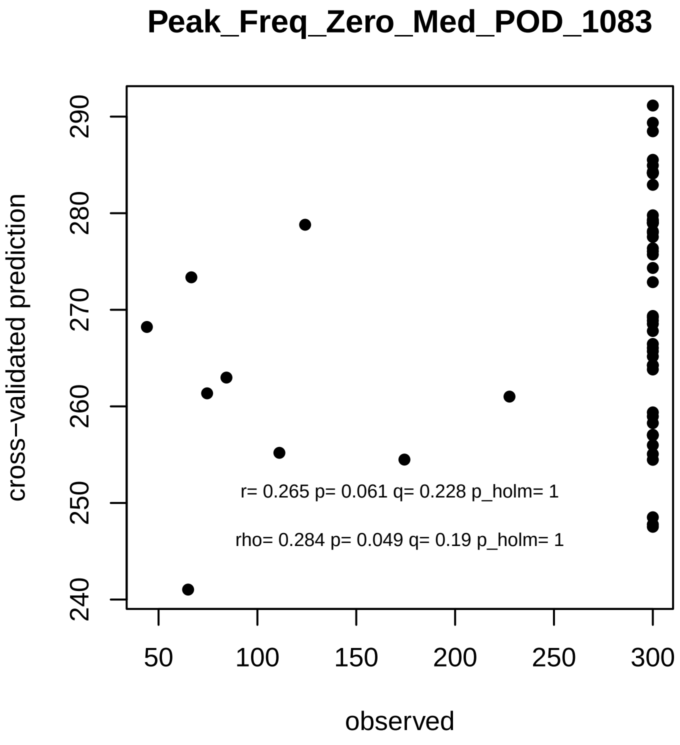
Peak_Freq_Zero_Med_POD_1083
cross−validated prediction
observed
r= 0.265 p= 0.061 q= 0.228 p_holm= 1
rho= 0.284 p= 0.049 q= 0.19 p_holm= 1

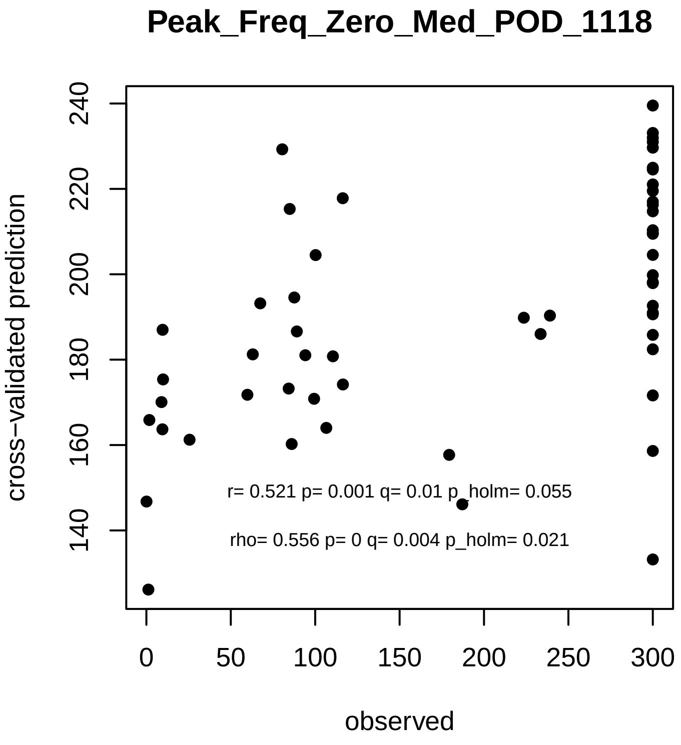
Peak_Freq_Zero_Med_POD_1118
cross−validated prediction
observed
r= 0.521 p= 0.001 q= 0.01 p_holm= 0.055
rho= 0.556 p= 0 q= 0.004 p_holm= 0.021

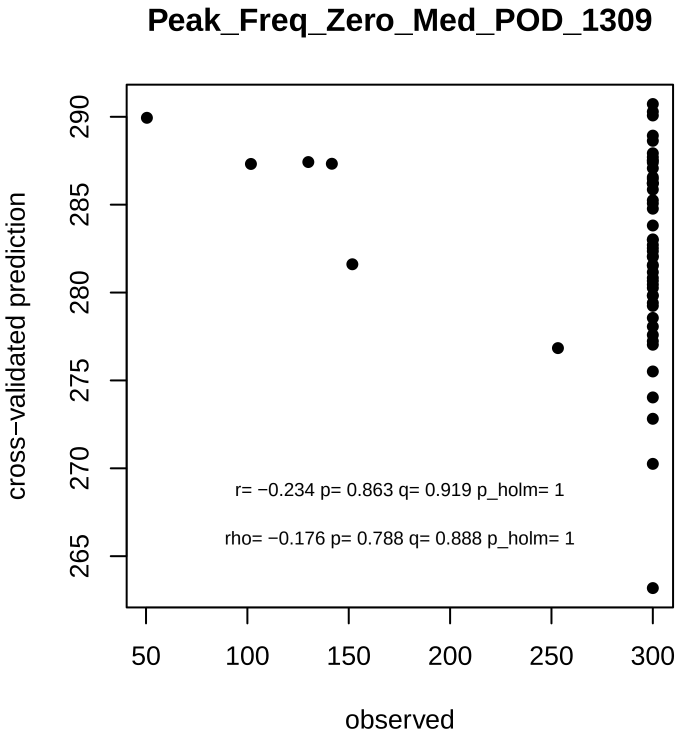
Peak_Freq_Zero_Med_POD_1309
cross−validated prediction
observed
r= −0.234 p= 0.863 q= 0.919 p_holm= 1
rho= −0.176 p= 0.788 q= 0.888 p_holm= 1

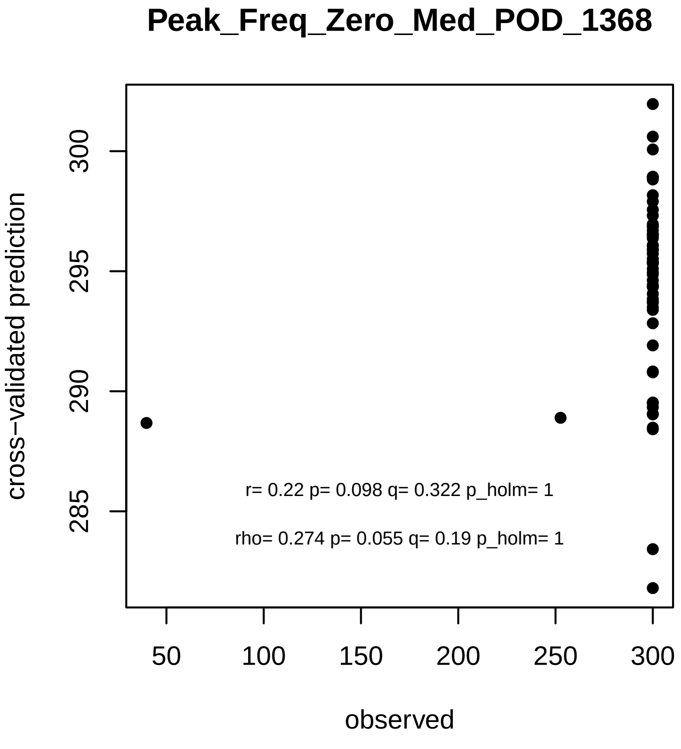
Peak_Freq_Zero_Med_POD_1368
cross−validated prediction
observed
r= 0.22 p= 0.098 q= 0.322 p_holm= 1
rho= 0.274 p= 0.055 q= 0.19 p_holm= 1

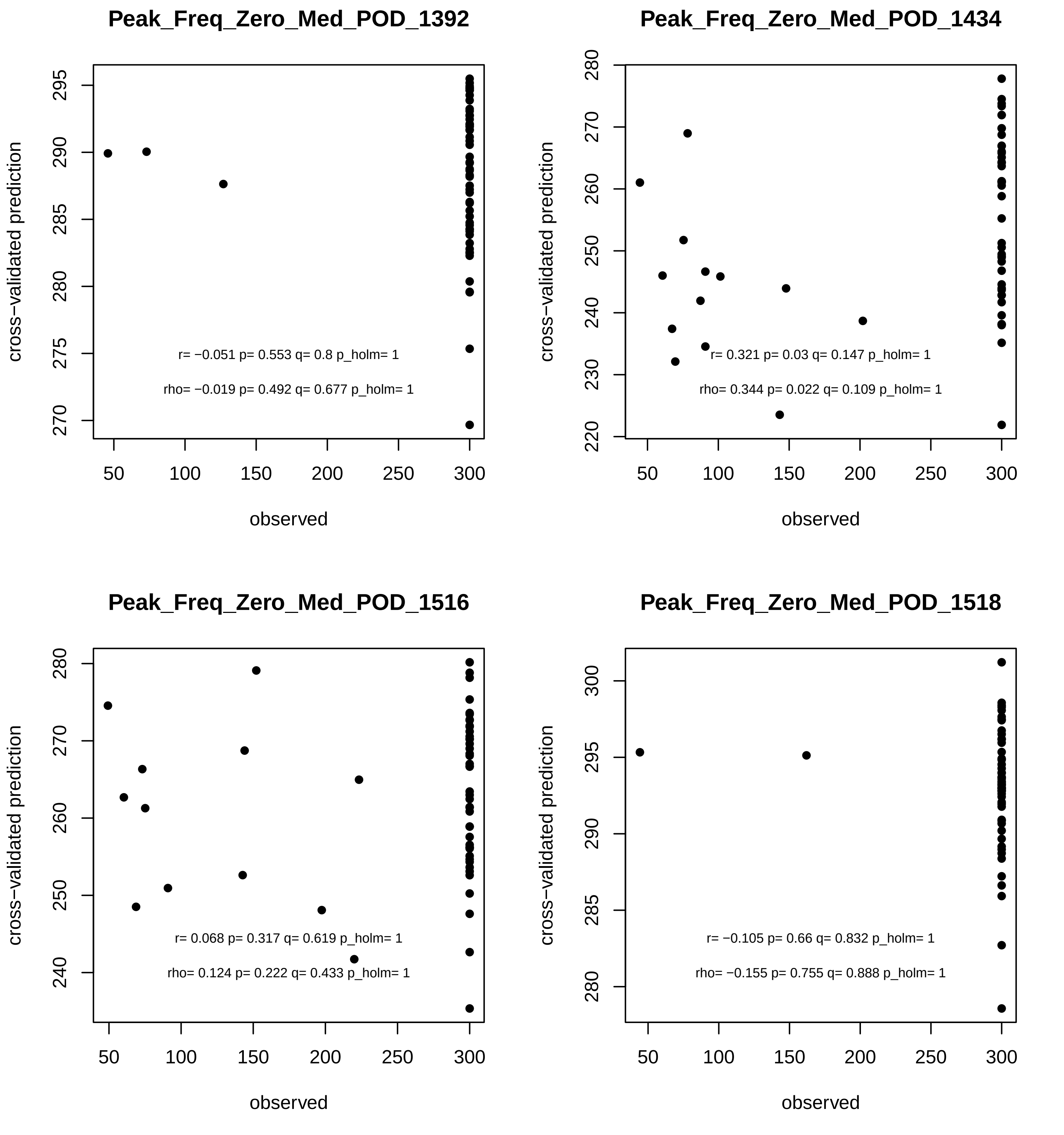
Peak_Freq_Zero_Med_POD_1392
r= −0.051 p= 0.553 q= 0.8 p_holm= 1
rho= −0.019 p= 0.492 q= 0.677 p_holm= 1
Peak_Freq_Zero_Med_POD_1434
r= 0.321 p= 0.03 q= 0.147 p_holm= 1
rho= 0.344 p= 0.022 q= 0.109 p_holm= 1
Peak_Freq_Zero_Med_POD_1516
r= 0.068 p= 0.317 q= 0.619 p_holm= 1
rho= 0.124 p= 0.222 q= 0.433 p_holm= 1
Peak_Freq_Zero_Med_POD_1518
r= −0.105 p= 0.66 q= 0.832 p_holm= 1
rho= −0.155 p= 0.755 q= 0.888 p_holm= 1
cross−validated prediction
observed

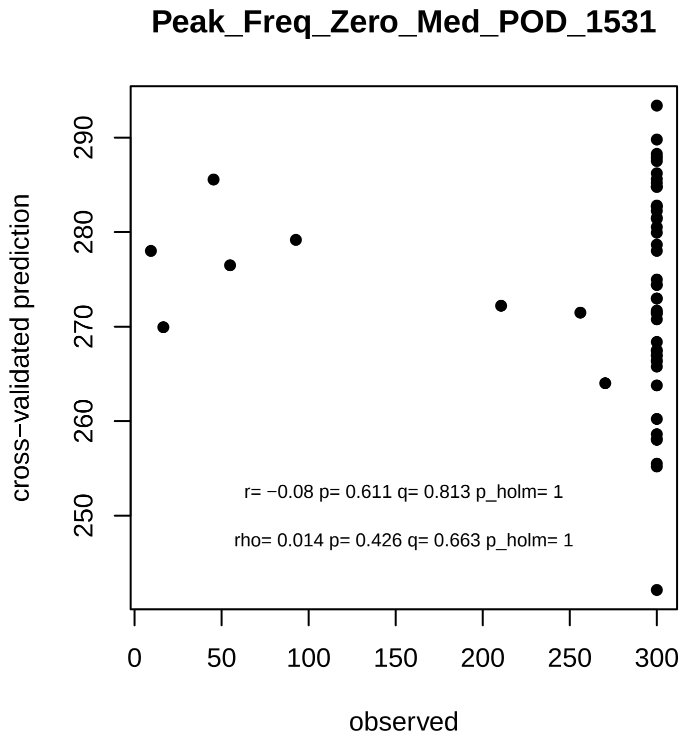
Peak_Freq_Zero_Med_POD_1531
r= −0.08 p= 0.611 q= 0.813 p_holm= 1
rho= 0.014 p= 0.426 q= 0.663 p_holm= 1
cross−validated prediction
observed

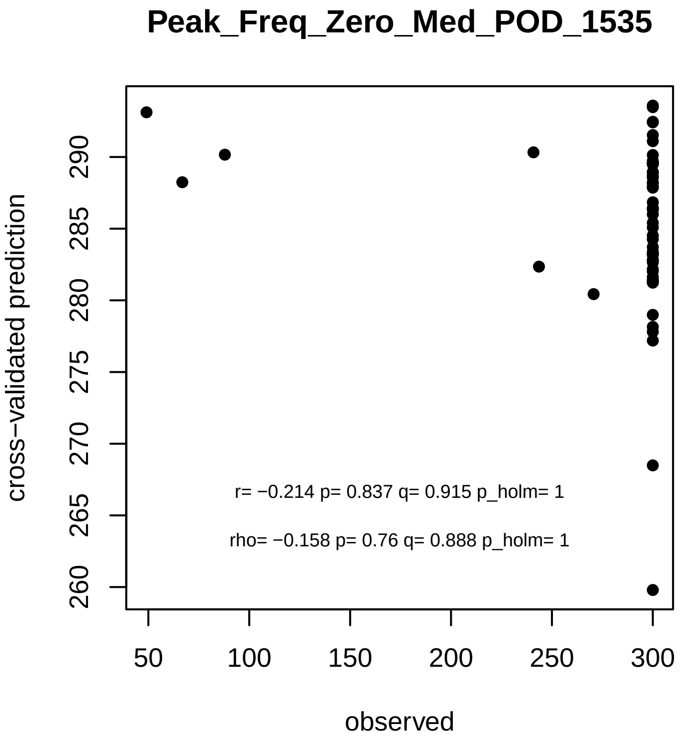
Peak_Freq_Zero_Med_POD_1535
r= −0.214 p= 0.837 q= 0.915 p_holm= 1
rho= −0.158 p= 0.76 q= 0.888 p_holm= 1
cross−validated prediction
observed

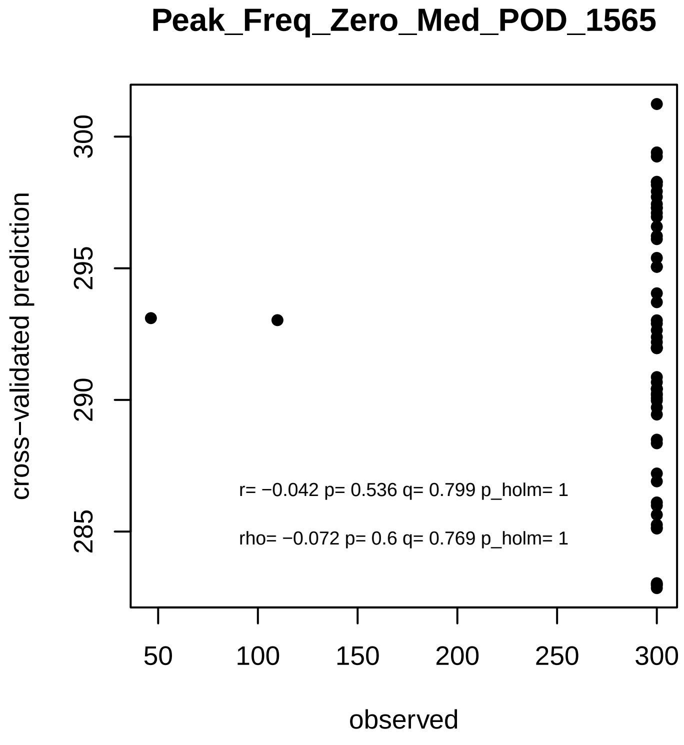
Peak_Freq_Zero_Med_POD_1565
r= −0.042 p= 0.536 q= 0.799 p_holm= 1
rho= −0.072 p= 0.6 q= 0.769 p_holm= 1
cross−validated prediction
observed

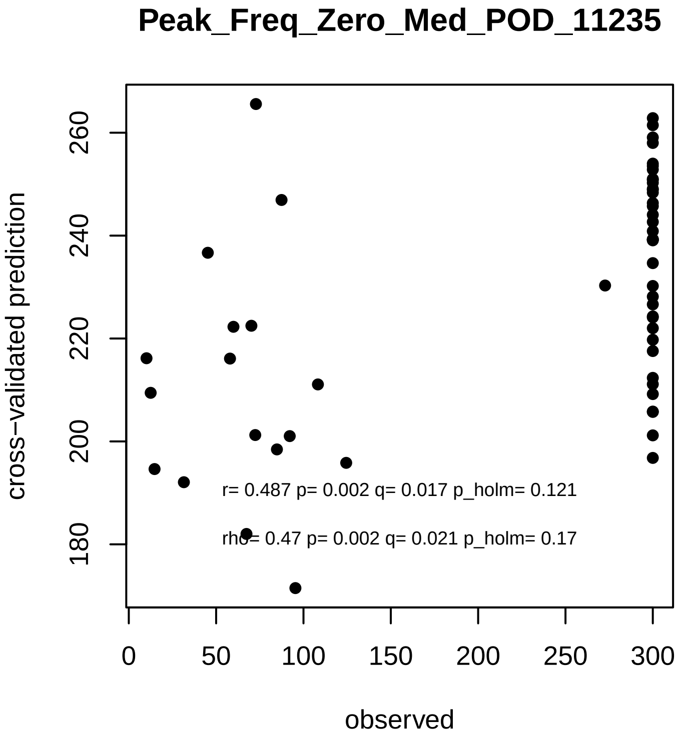
Peak_Freq_Zero_Med_POD_11235
r= 0.487 p= 0.002 q= 0.017 p_holm= 0.121
rho= 0.47 p= 0.002 q= 0.021 p_holm= 0.17
cross−validated prediction
observed

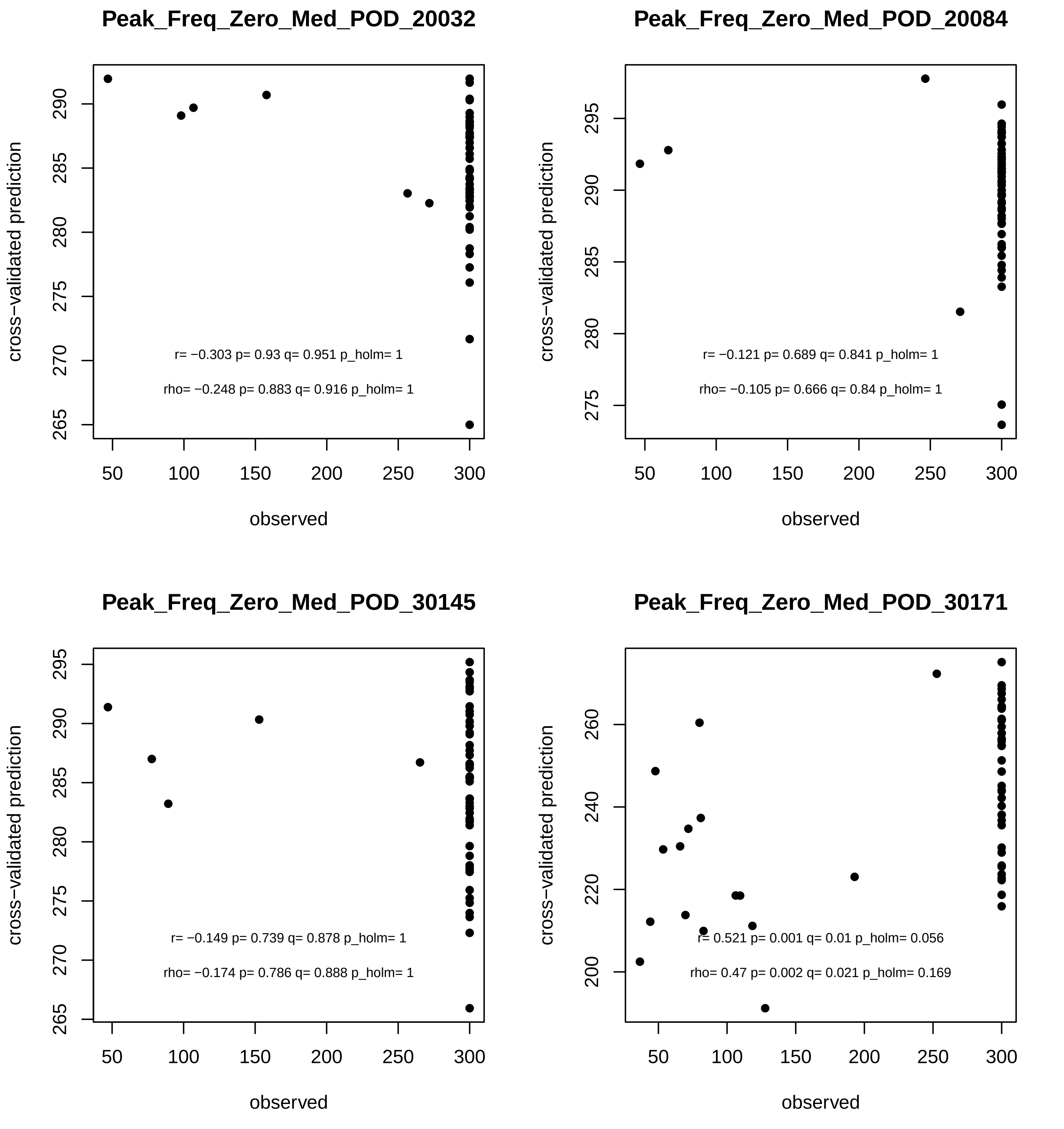

Peak_Freq_Zero_Med_POD_20032
r= −0.303 p= 0.93 q= 0.951 p_holm= 1
rho= −0.248 p= 0.883 q= 0.916 p_holm= 1
Peak_Freq_Zero_Med_POD_20084
r= −0.121 p= 0.689 q= 0.841 p_holm= 1
rho= −0.105 p= 0.666 q= 0.84 p_holm= 1
Peak_Freq_Zero_Med_POD_30145
r= −0.149 p= 0.739 q= 0.878 p_holm= 1
rho= −0.174 p= 0.786 q= 0.888 p_holm= 1
Peak_Freq_Zero_Med_POD_30171
r= 0.521 p= 0.001 q= 0.01 p_holm= 0.056
rho= 0.47 p= 0.002 q= 0.021 p_holm= 0.169
cross−validated prediction
observed

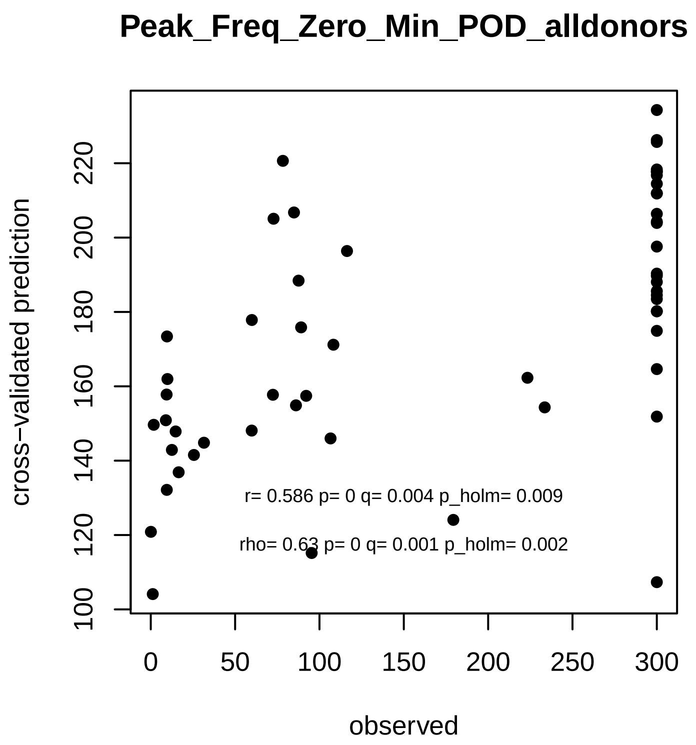
Peak_Freq_Zero_Min_POD_alldonors
cross−validated prediction
observed
r= 0.586 p= 0 q= 0.004 p_holm= 0.009
rho= 0.63 p= 0 q= 0.001 p_holm= 0.002

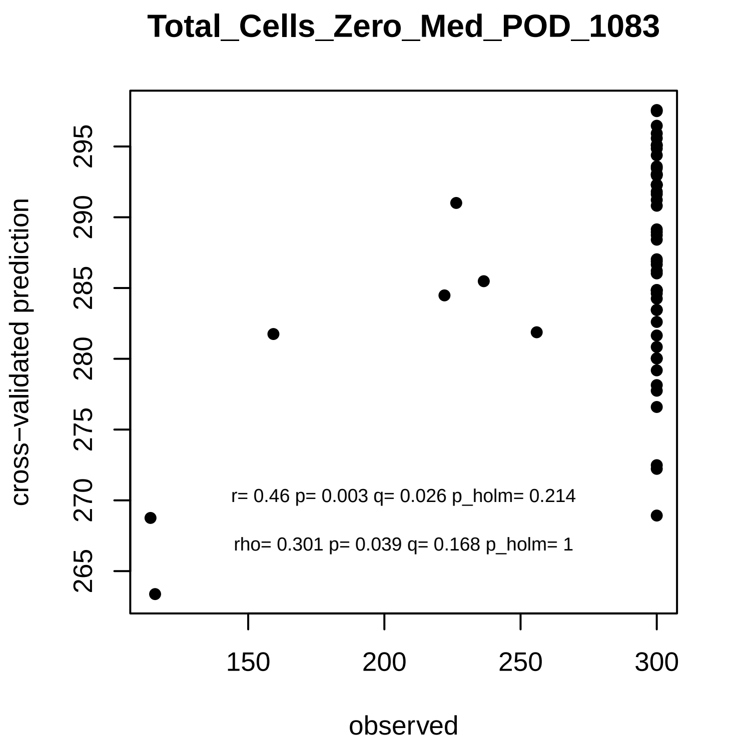
Total_Cells_Zero_Med_POD_1083
cross−validated prediction
observed
r= 0.46 p= 0.003 q= 0.026 p_holm= 0.214
rho= 0.301 p= 0.039 q= 0.168 p_holm= 1

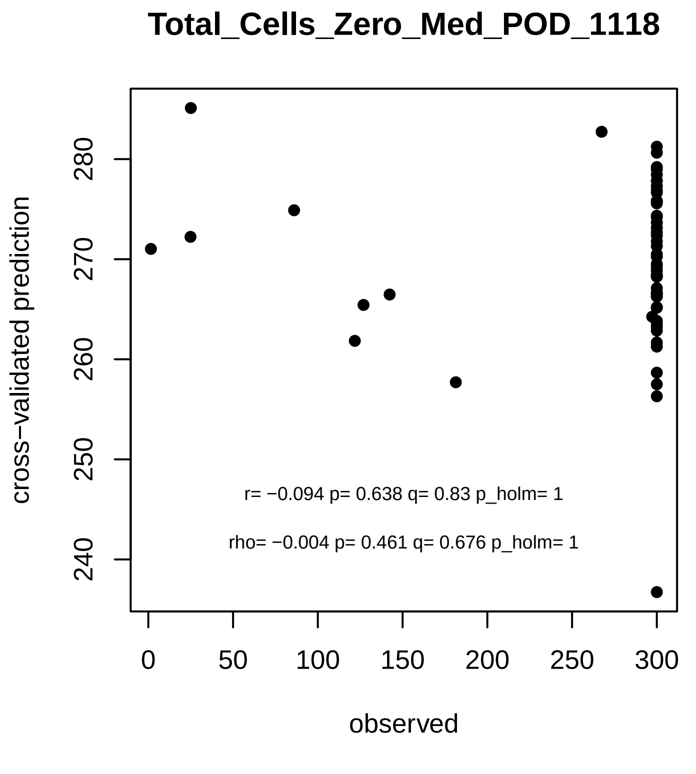
Total_Cells_Zero_Med_POD_1118
cross−validated prediction
observed
r= −0.094 p= 0.638 q= 0.83 p_holm= 1
rho= −0.004 p= 0.461 q= 0.676 p_holm= 1

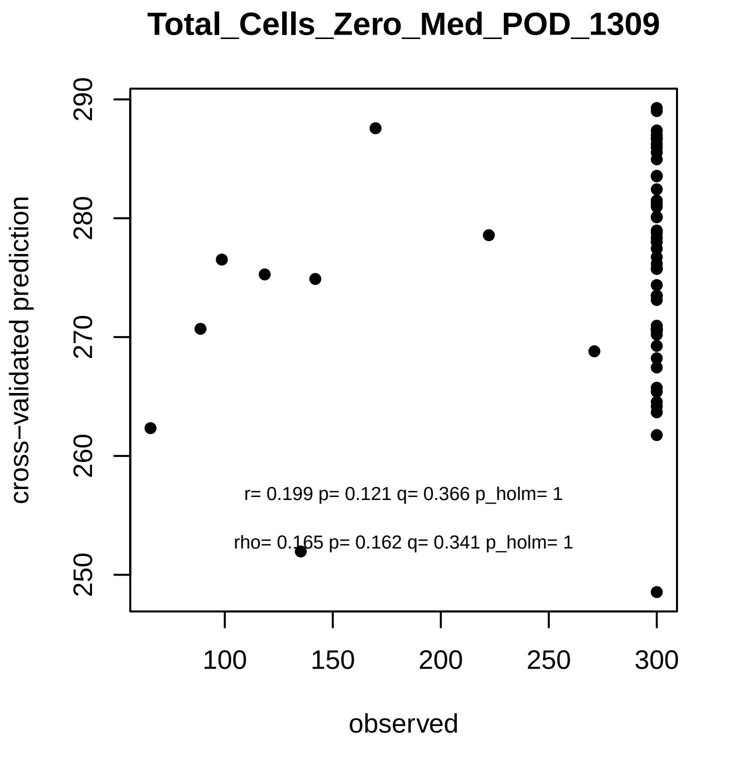
Total_Cells_Zero_Med_POD_1309
cross−validated prediction
observed
r= 0.199 p= 0.121 q= 0.366 p_holm= 1
rho= 0.165 p= 0.162 q= 0.341 p_holm= 1

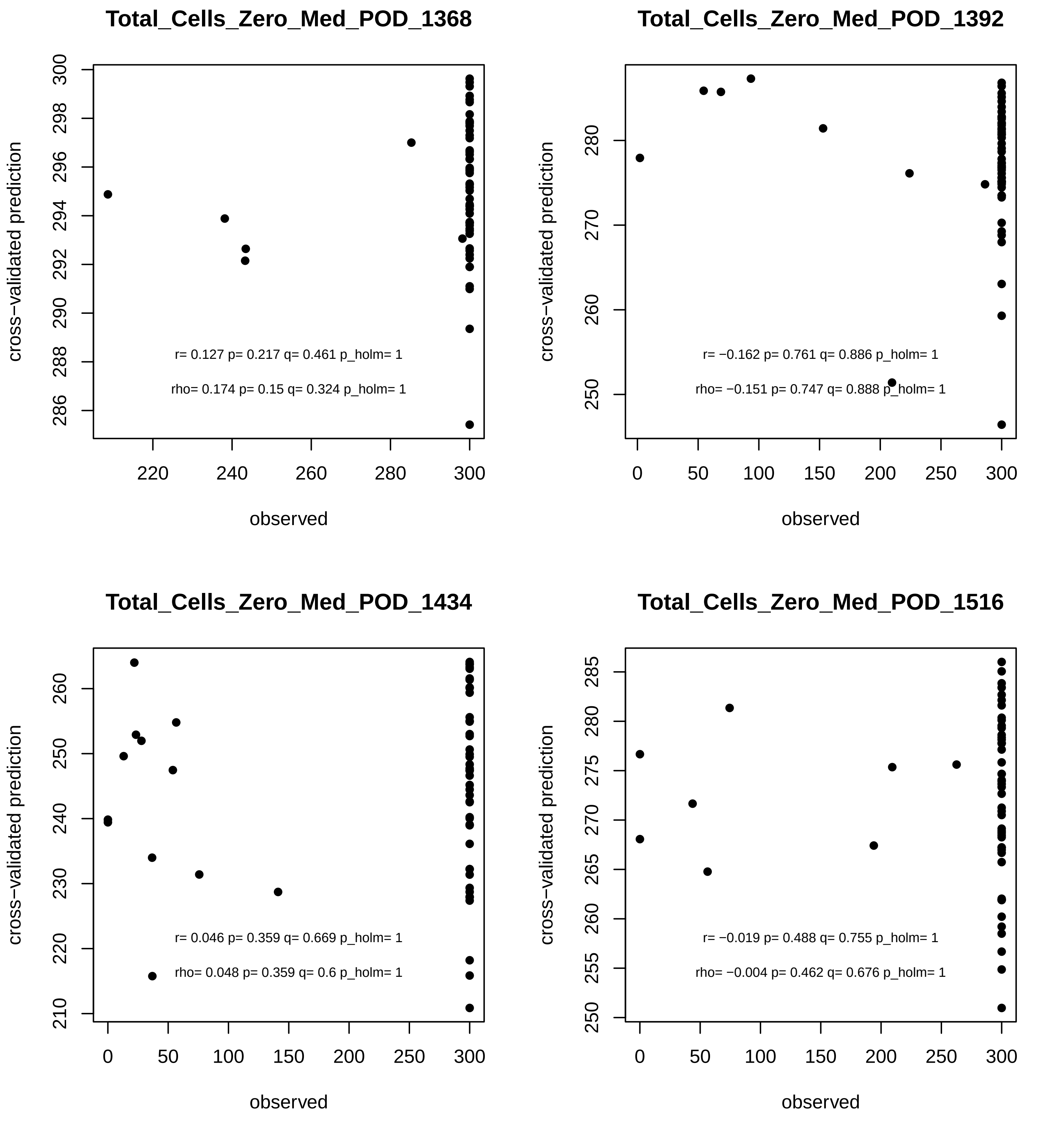
Total_Cells_Zero_Med_POD_1368
r= 0.127 p= 0.217 q= 0.461 p_holm= 1
rho= 0.174 p= 0.15 q= 0.324 p_holm= 1
Total_Cells_Zero_Med_POD_1392
r= −0.162 p= 0.761 q= 0.886 p_holm= 1
rho= −0.151 p= 0.747 q= 0.888 p_holm= 1
Total_Cells_Zero_Med_POD_1434
r= 0.046 p= 0.359 q= 0.669 p_holm= 1
rho= 0.048 p= 0.359 q= 0.6 p_holm= 1
Total_Cells_Zero_Med_POD_1516
r= −0.019 p= 0.488 q= 0.755 p_holm= 1
rho= −0.004 p= 0.462 q= 0.676 p_holm= 1
cross−validated prediction
observed

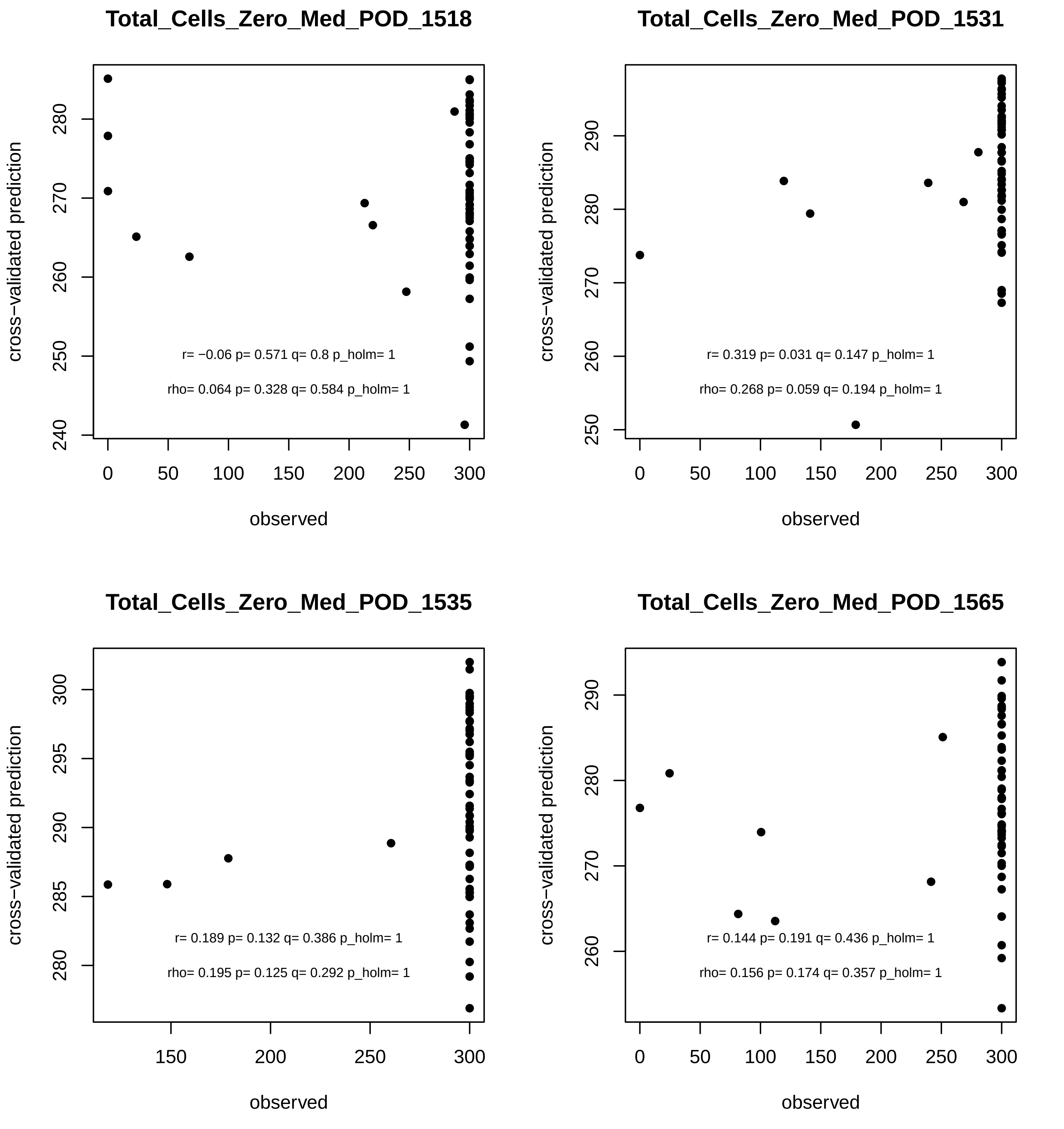
Total_Cells_Zero_Med_POD_1518
cross−validated prediction
observed
r= −0.06 p= 0.571 q= 0.8 p_holm= 1
rho= 0.064 p= 0.328 q= 0.584 p_holm= 1
Total_Cells_Zero_Med_POD_1531
cross−validated prediction
observed
r= 0.319 p= 0.031 q= 0.147 p_holm= 1
rho= 0.268 p= 0.059 q= 0.194 p_holm= 1
Total_Cells_Zero_Med_POD_1535
cross−validated prediction
observed
r= 0.189 p= 0.132 q= 0.386 p_holm= 1
rho= 0.195 p= 0.125 q= 0.292 p_holm= 1
Total_Cells_Zero_Med_POD_1565
cross−validated prediction
observed
r= 0.144 p= 0.191 q= 0.436 p_holm= 1
rho= 0.156 p= 0.174 q= 0.357 p_holm= 1

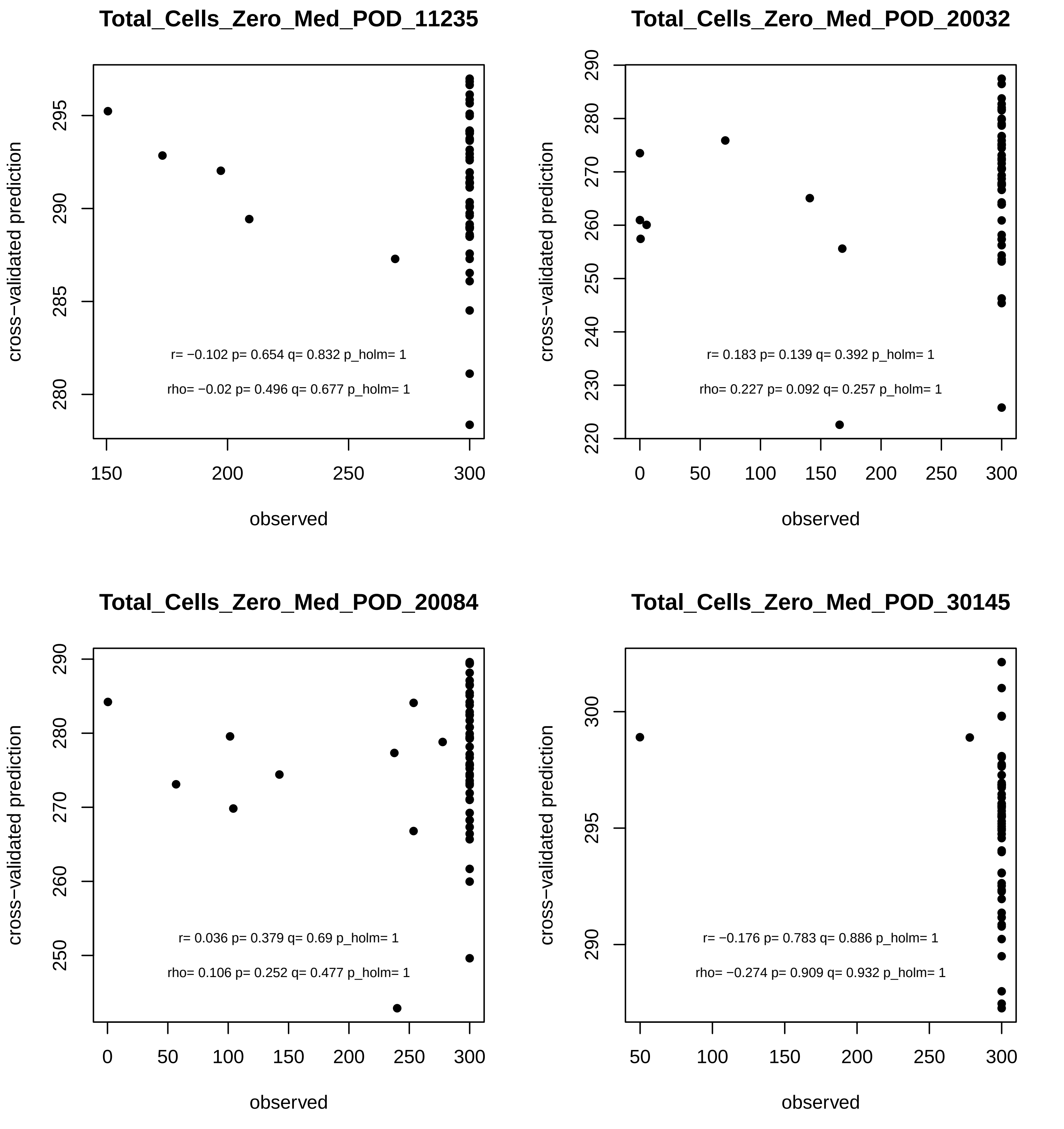
Total_Cells_Zero_Med_POD_11235
r= −0.102 p= 0.654 q= 0.832 p_holm= 1
rho= −0.02 p= 0.496 q= 0.677 p_holm= 1
Total_Cells_Zero_Med_POD_20032
r= 0.183 p= 0.139 q= 0.392 p_holm= 1
rho= 0.227 p= 0.092 q= 0.257 p_holm= 1
Total_Cells_Zero_Med_POD_20084
r= 0.036 p= 0.379 q= 0.69 p_holm= 1
rho= 0.106 p= 0.252 q= 0.477 p_holm= 1
Total_Cells_Zero_Med_POD_30145
r= −0.176 p= 0.783 q= 0.886 p_holm= 1
rho= −0.274 p= 0.909 q= 0.932 p_holm= 1
cross−validated prediction
observed

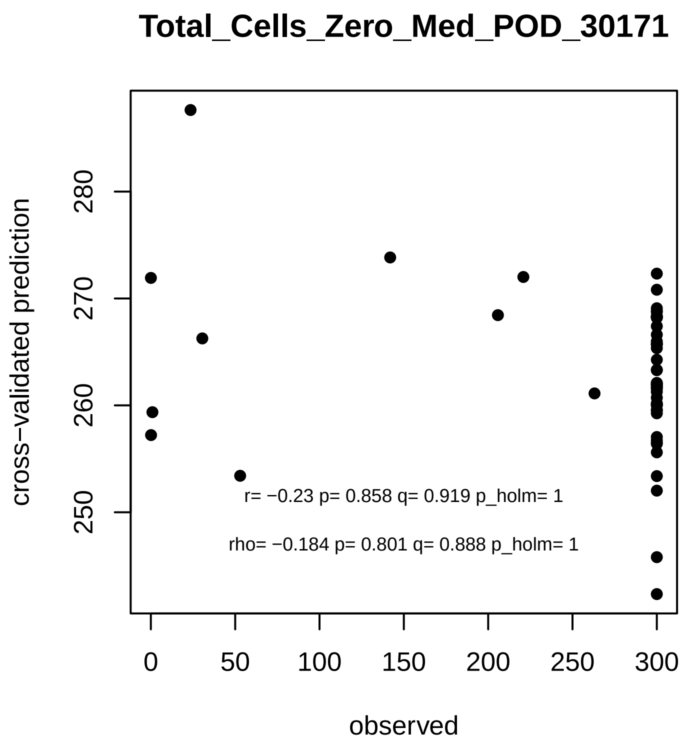

Total_Cells_Zero_Med_POD_30171
cross−validated prediction
observed
r= −0.23 p= 0.858 q= 0.919 p_holm= 1
rho= −0.184 p= 0.801 q= 0.888 p_holm= 1

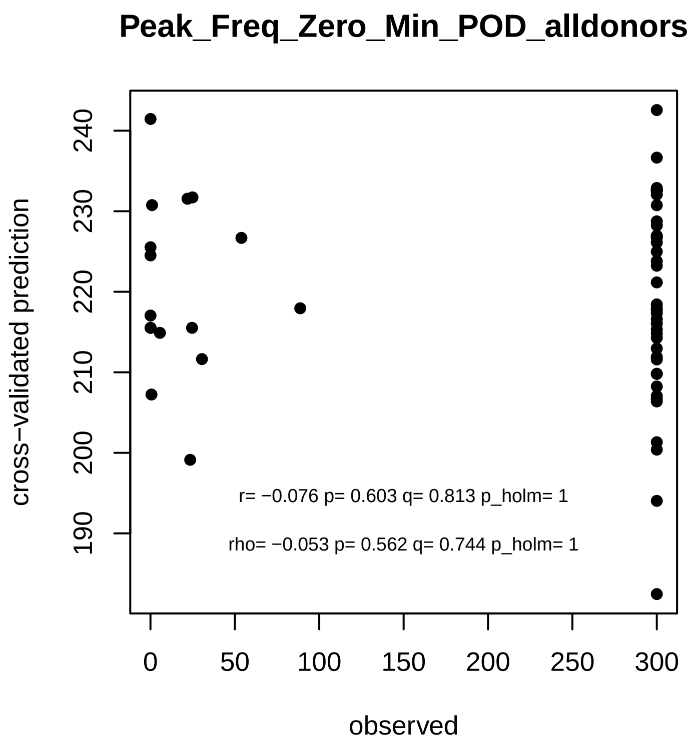

Peak_Freq_Zero_Min_POD_alldonors
cross−validated prediction
observed
r= −0.076 p= 0.603 q= 0.813 p_holm= 1
rho= −0.053 p= 0.562 q= 0.744 p_holm= 1
