# Supplementary material for: Characterizing PFAS hazards and risks: a human population-based in vitro cardiotoxicity assessment strategy
Source: Hum Genomics. 2024 Sep 2;18:92. doi: 10.1186/s40246-024-00665-x (PMC11368000; doi:10.1186/s40246-024-00665-x)
Supplement: Supplementary file 7 — Supplementary Material 7 [file 40246_2024_665_MOESM7_ESM.pdf]

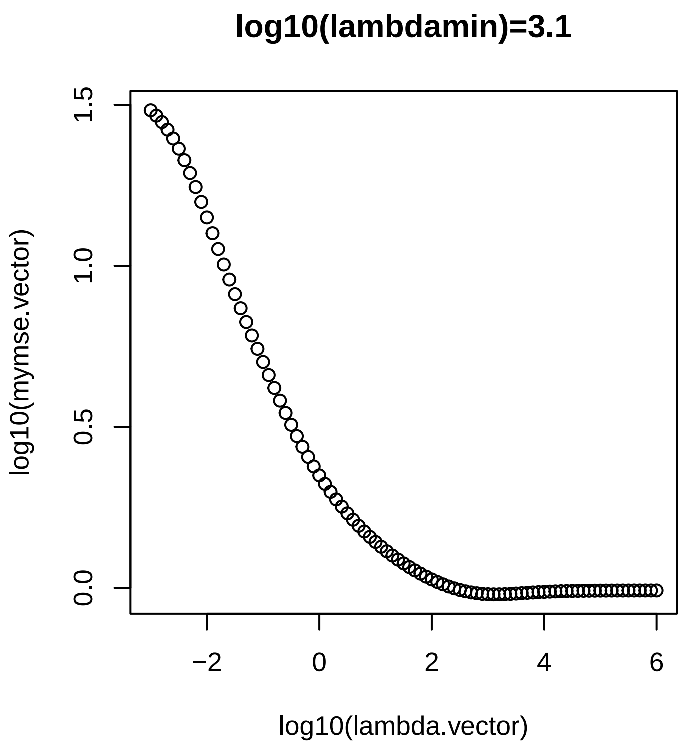
log10(lambdamin)=3.1
log10(mymse.vector)
0.0
0.5
1.0
1.5
−2
0
2
4
6
log10(lambda.vector)

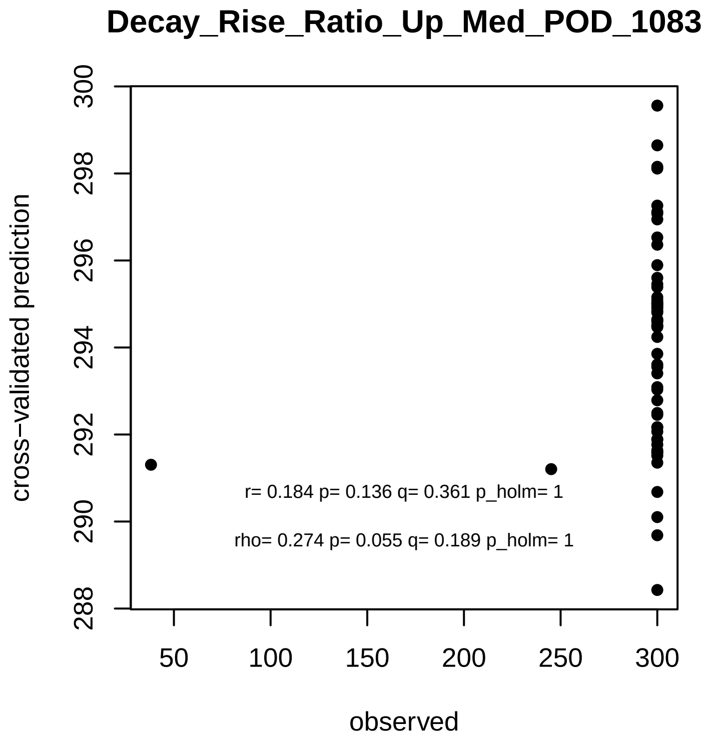
Decay_Rise_Ratio_Up_Med_POD_1083
cross−validated prediction
observed
r= 0.184 p= 0.136 q= 0.361 p_holm= 1
rho= 0.274 p= 0.055 q= 0.189 p_holm= 1

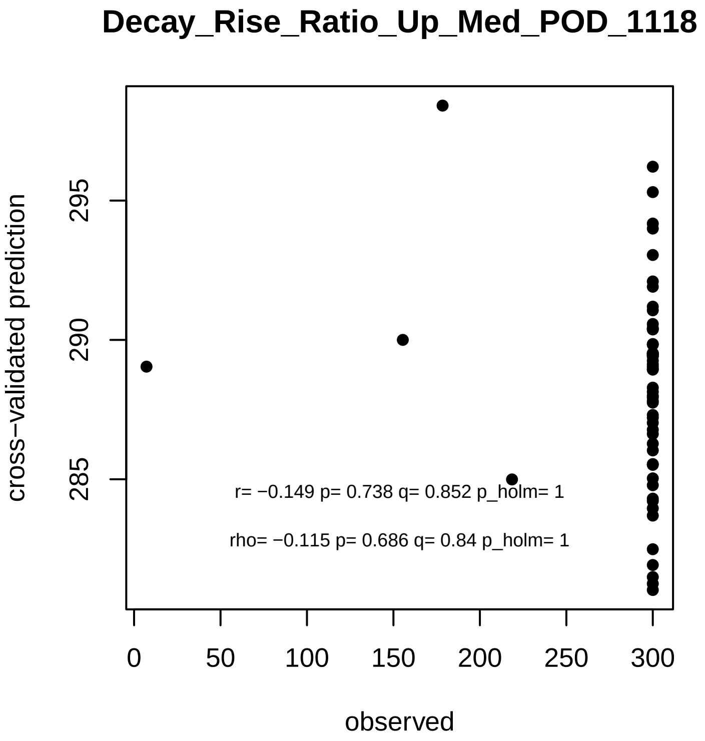
Decay_Rise_Ratio_Up_Med_POD_1118
cross−validated prediction
observed
r= −0.149 p= 0.738 q= 0.852 p_holm= 1
rho= −0.115 p= 0.686 q= 0.84 p_holm= 1

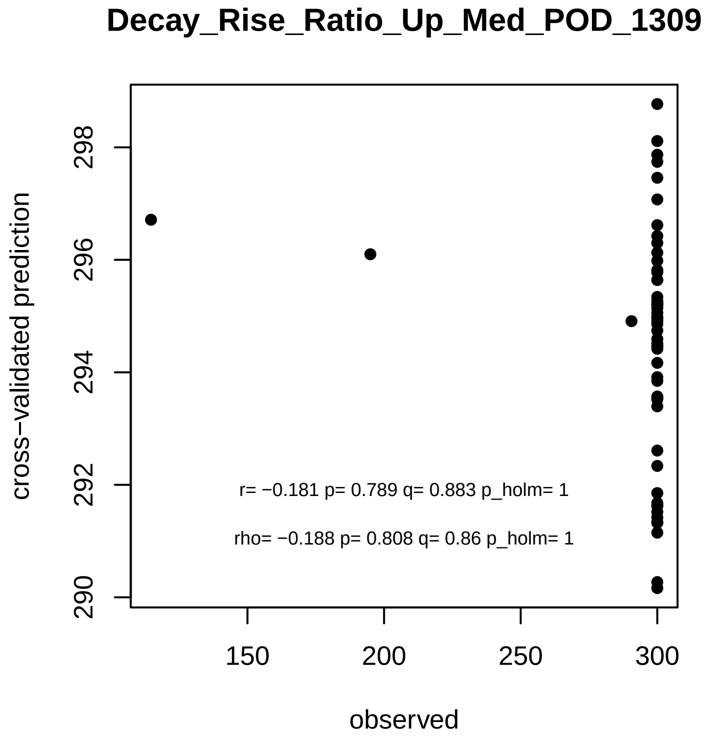
Decay_Rise_Ratio_Up_Med_POD_1309
cross−validated prediction
observed
r= −0.181 p= 0.789 q= 0.883 p_holm= 1
rho= −0.188 p= 0.808 q= 0.86 p_holm= 1

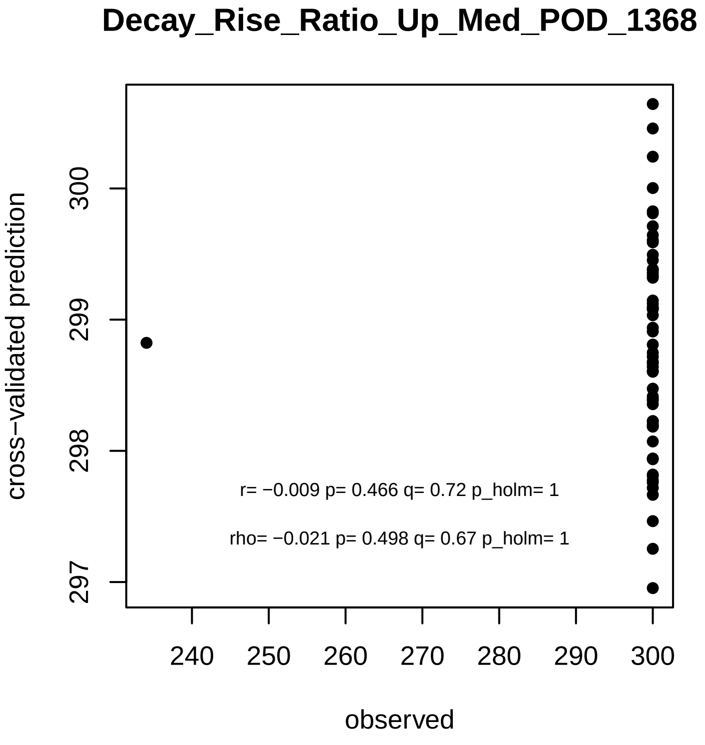
Decay_Rise_Ratio_Up_Med_POD_1368
cross−validated prediction
observed
r= −0.009 p= 0.466 q= 0.72 p_holm= 1
rho= −0.021 p= 0.498 q= 0.67 p_holm= 1

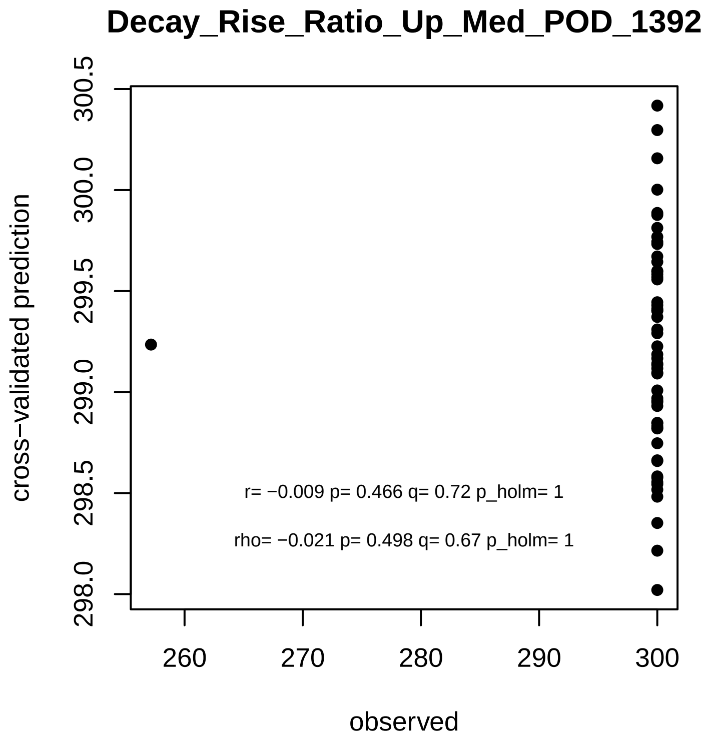
Decay_Rise_Ratio_Up_Med_POD_1392
cross−validated prediction
observed
r= −0.009 p= 0.466 q= 0.72 p_holm= 1
rho= −0.021 p= 0.498 q= 0.67 p_holm= 1

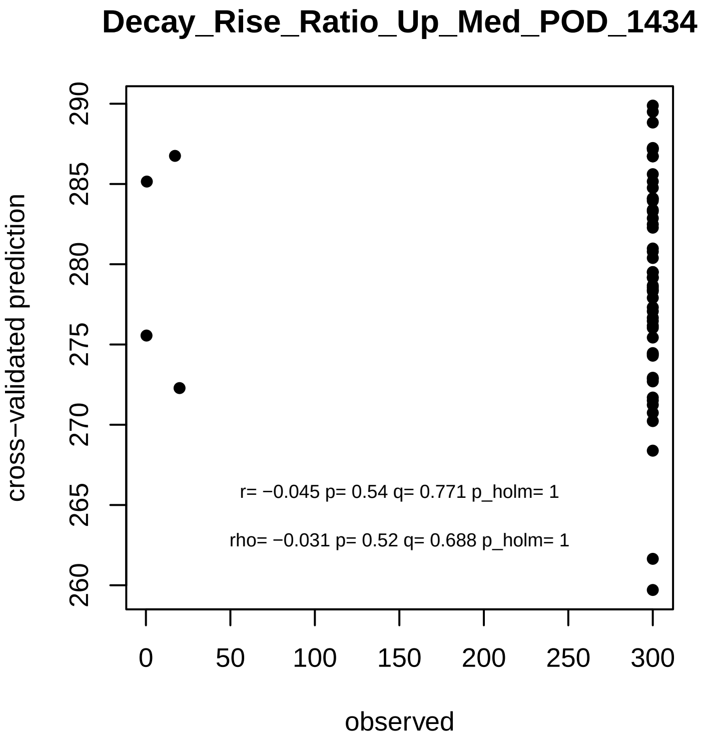
Decay_Rise_Ratio_Up_Med_POD_1434
cross−validated prediction
observed
r= −0.045 p= 0.54 q= 0.771 p_holm= 1
rho= −0.031 p= 0.52 q= 0.688 p_holm= 1

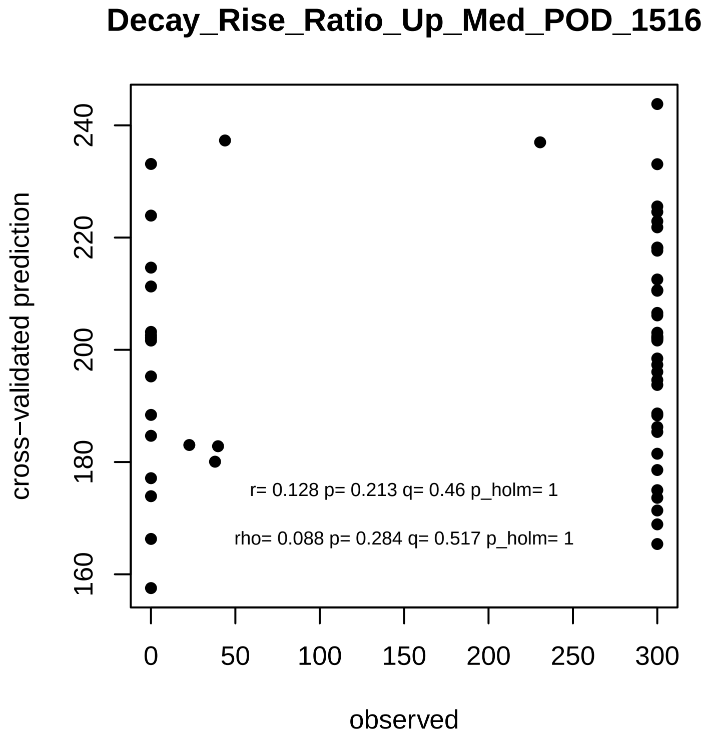
Decay_Rise_Ratio_Up_Med_POD_1516
cross−validated prediction
observed
r= 0.128 p= 0.213 q= 0.46 p_holm= 1
rho= 0.088 p= 0.284 q= 0.517 p_holm= 1

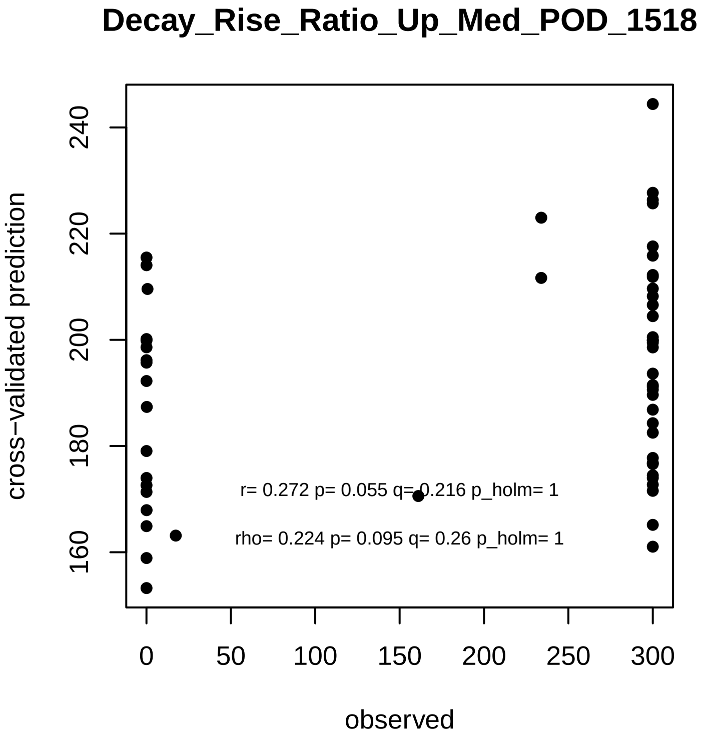
Decay_Rise_Ratio_Up_Med_POD_1518
cross−validated prediction
observed
r= 0.272 p= 0.055 q= 0.216 p_holm= 1
rho= 0.224 p= 0.095 q= 0.26 p_holm= 1

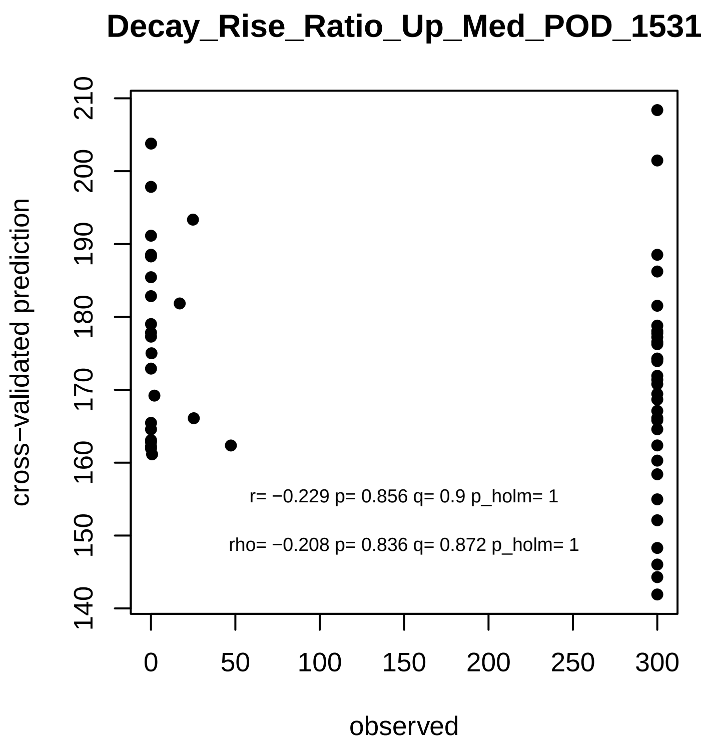
Decay_Rise_Ratio_Up_Med_POD_1531
cross−validated prediction
observed
r= −0.229 p= 0.856 q= 0.9 p_holm= 1
rho= −0.208 p= 0.836 q= 0.872 p_holm= 1

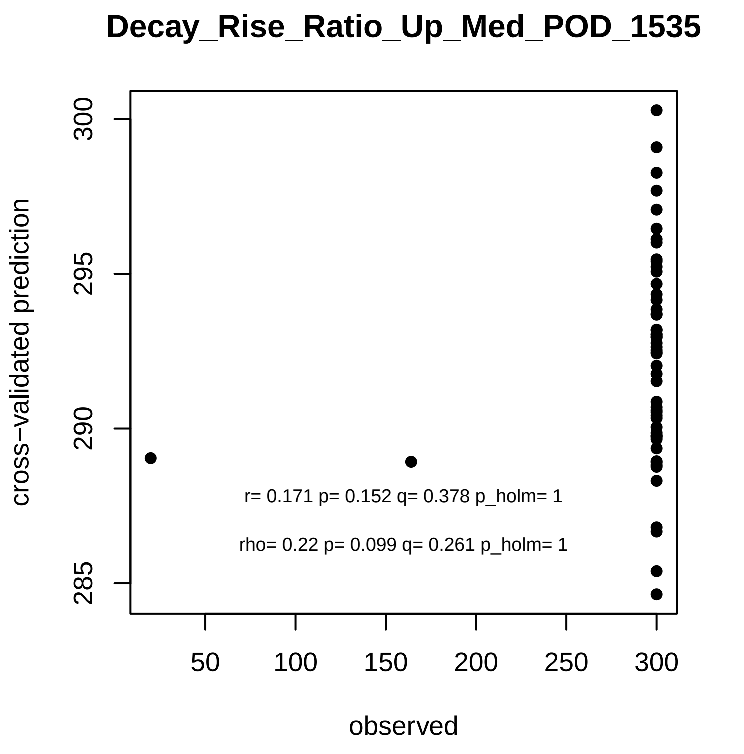
Decay_Rise_Ratio_Up_Med_POD_1535
cross−validated prediction
observed
r= 0.171 p= 0.152 q= 0.378 p_holm= 1
rho= 0.22 p= 0.099 q= 0.261 p_holm= 1

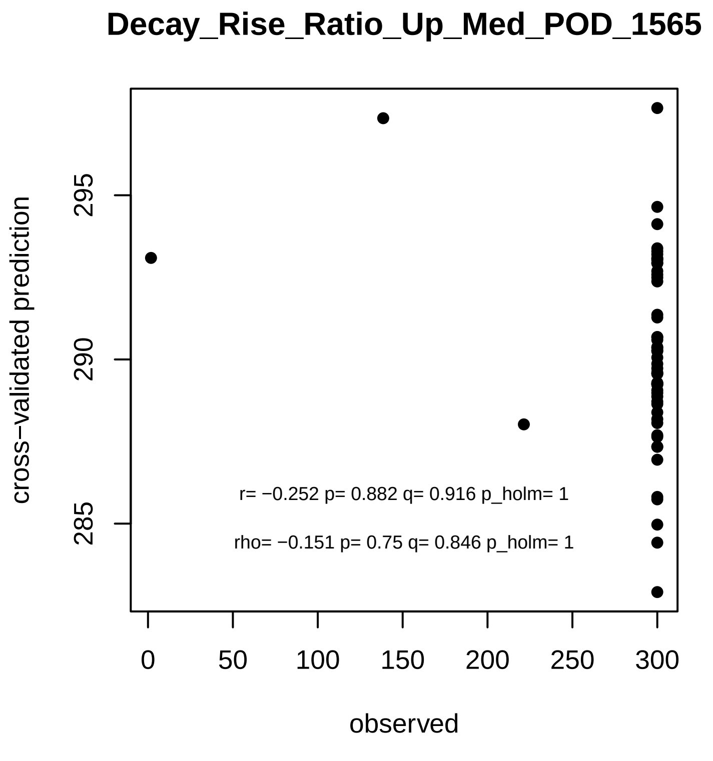
Decay_Rise_Ratio_Up_Med_POD_1565
cross−validated prediction
observed
r= −0.252 p= 0.882 q= 0.916 p_holm= 1
rho= −0.151 p= 0.75 q= 0.846 p_holm= 1

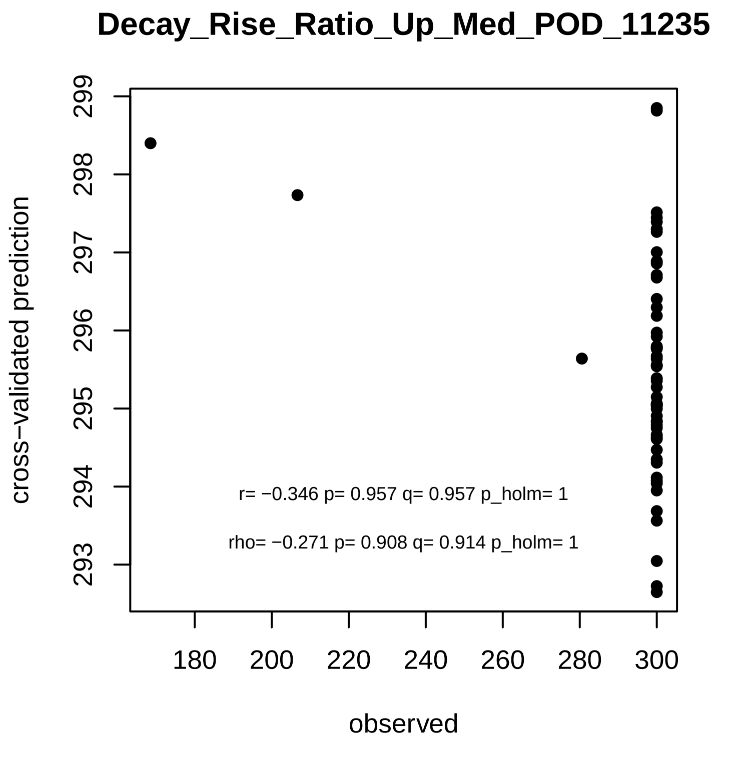
Decay_Rise_Ratio_Up_Med_POD_11235
cross−validated prediction
observed
r= −0.346 p= 0.957 q= 0.957 p_holm= 1
rho= −0.271 p= 0.908 q= 0.914 p_holm= 1

Decay_Rise_Ratio_Up_Med_POD_20032

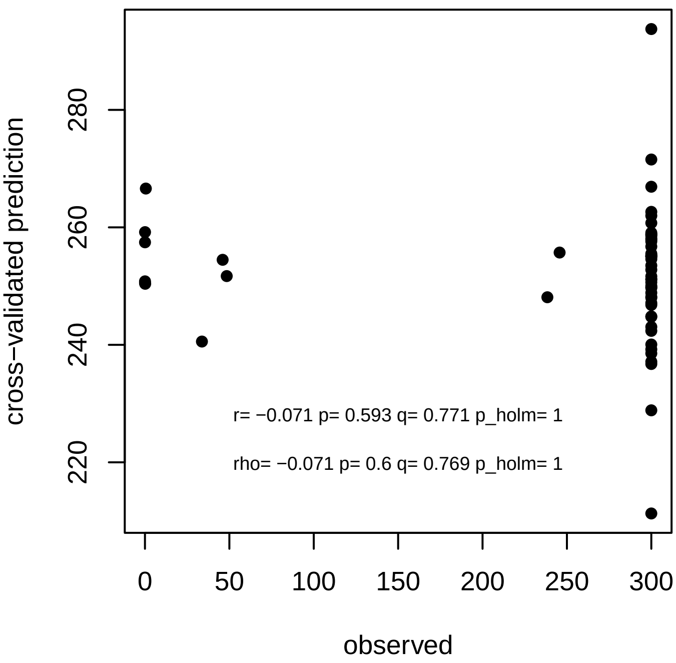

Decay_Rise_Ratio_Up_Med_POD_20084

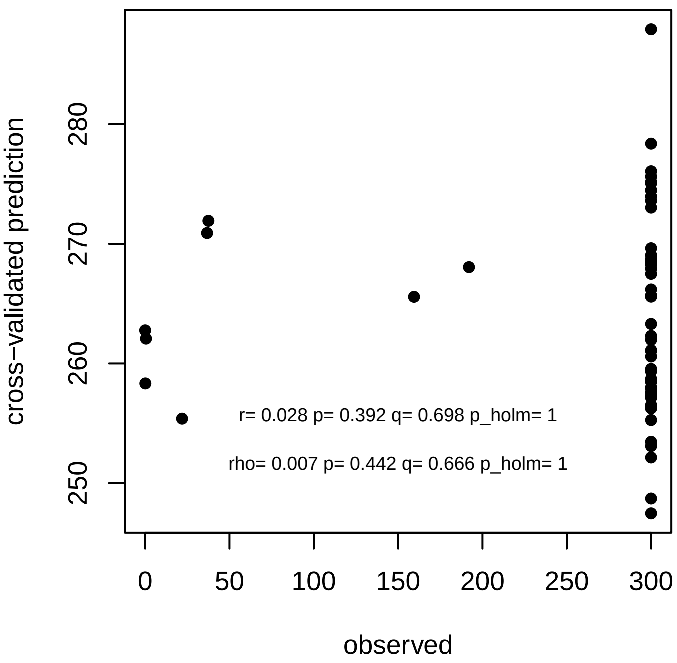

Decay_Rise_Ratio_Up_Med_POD_30145

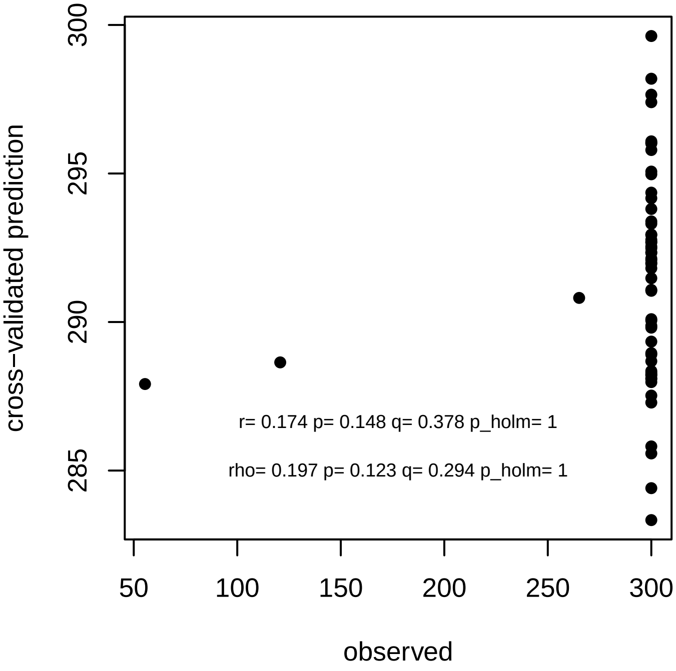

Decay_Rise_Ratio_Up_Med_POD_30171

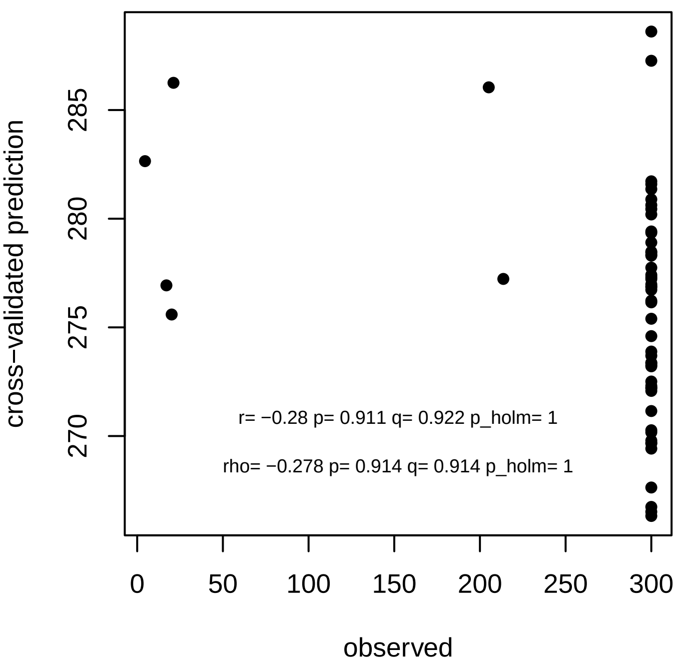

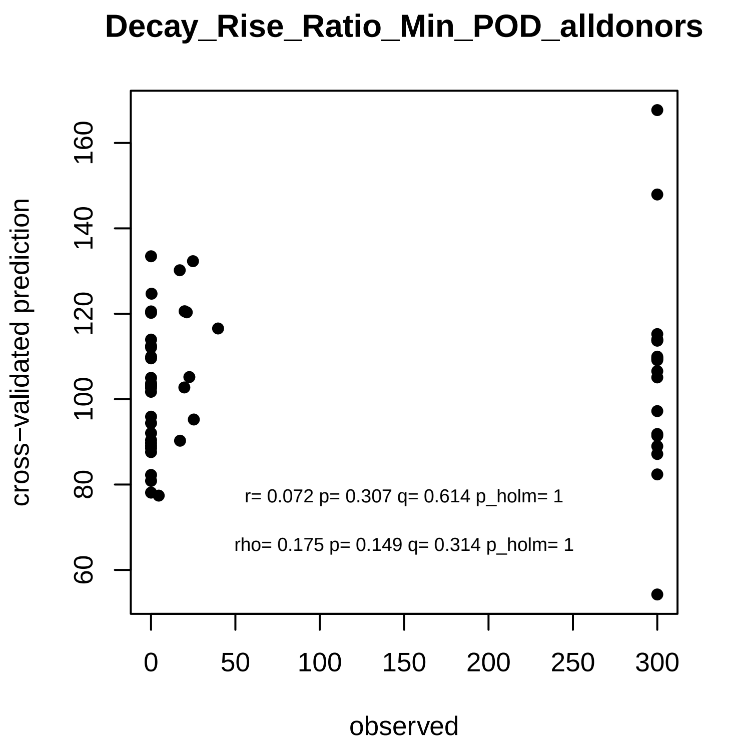

Decay_Rise_Ratio_Min_POD_alldonors
cross−validated prediction
observed
r= 0.072 p= 0.307 q= 0.614 p_holm= 1
rho= 0.175 p= 0.149 q= 0.314 p_holm= 1

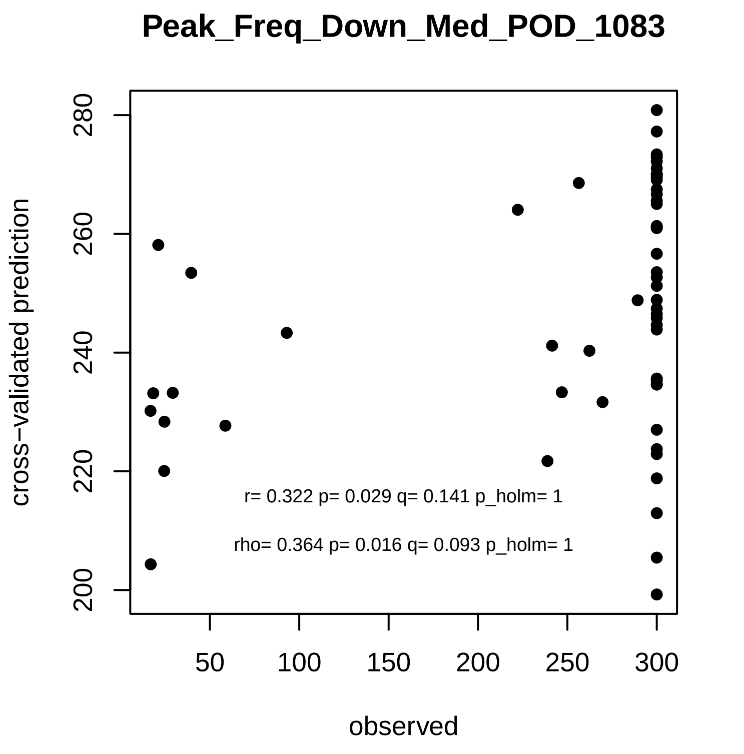

Peak_Freq_Down_Med_POD_1083
cross−validated prediction
observed
r= 0.322 p= 0.029 q= 0.141 p_holm= 1
rho= 0.364 p= 0.016 q= 0.093 p_holm= 1

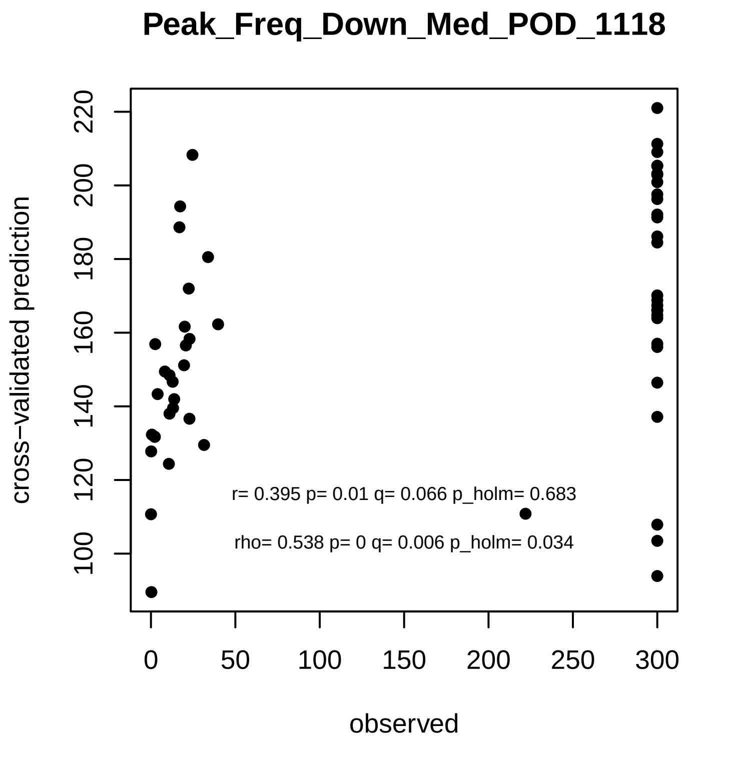

Peak_Freq_Down_Med_POD_1118
cross−validated prediction
observed
r= 0.395 p= 0.01 q= 0.066 p_holm= 0.683
rho= 0.538 p= 0 q= 0.006 p_holm= 0.034

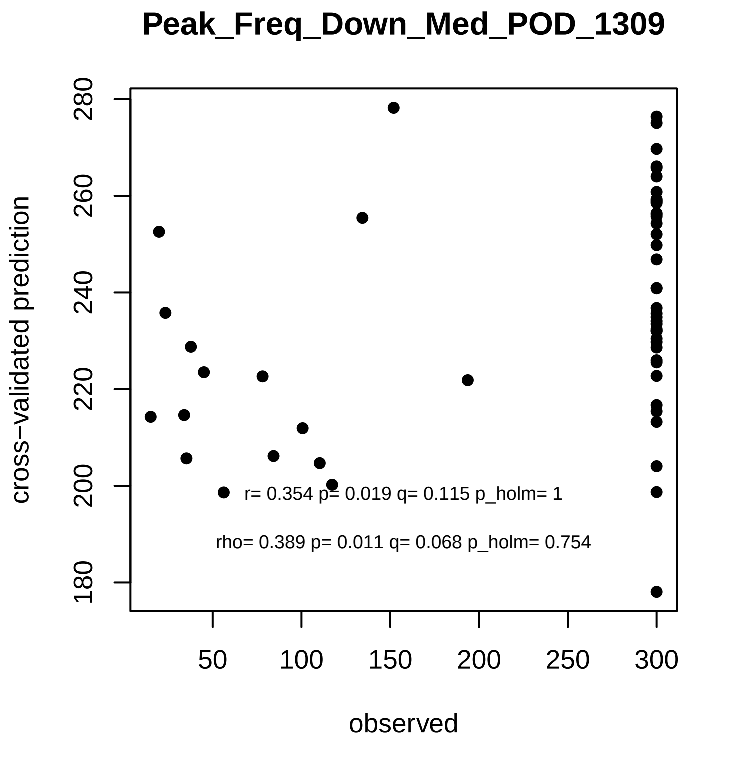

Peak_Freq_Down_Med_POD_1309
cross−validated prediction
observed
r= 0.354 p= 0.019 q= 0.115 p_holm= 1
rho= 0.389 p= 0.011 q= 0.068 p_holm= 0.754

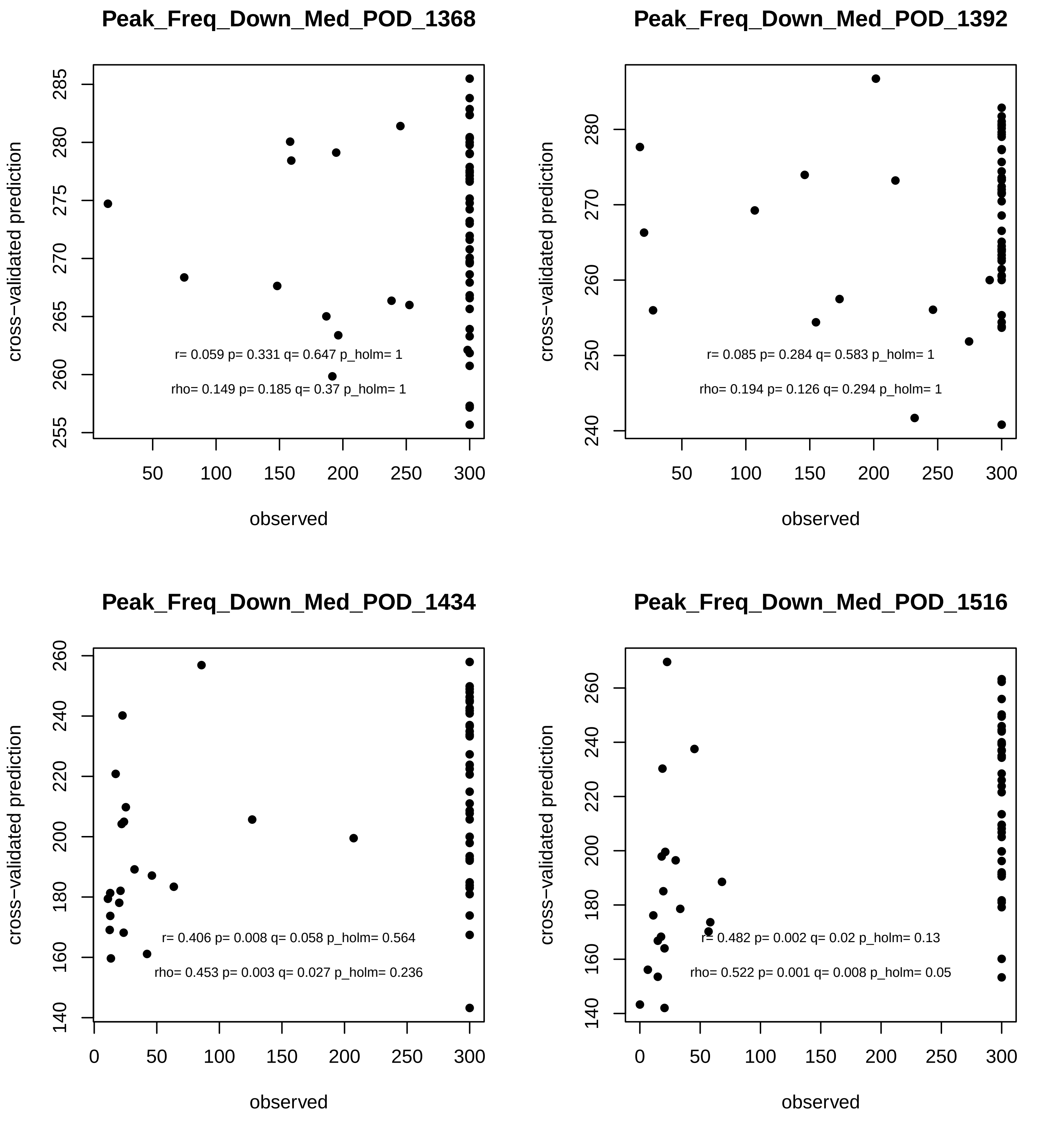

Peak_Freq_Down_Med_POD_1368
r= 0.059 p= 0.331 q= 0.647 p_holm= 1
rho= 0.149 p= 0.185 q= 0.37 p_holm= 1
Peak_Freq_Down_Med_POD_1392
r= 0.085 p= 0.284 q= 0.583 p_holm= 1
rho= 0.194 p= 0.126 q= 0.294 p_holm= 1
Peak_Freq_Down_Med_POD_1434
r= 0.406 p= 0.008 q= 0.058 p_holm= 0.564
rho= 0.453 p= 0.003 q= 0.027 p_holm= 0.236
Peak_Freq_Down_Med_POD_1516
r= 0.482 p= 0.002 q= 0.02 p_holm= 0.13
rho= 0.522 p= 0.001 q= 0.008 p_holm= 0.05
cross−validated prediction
observed

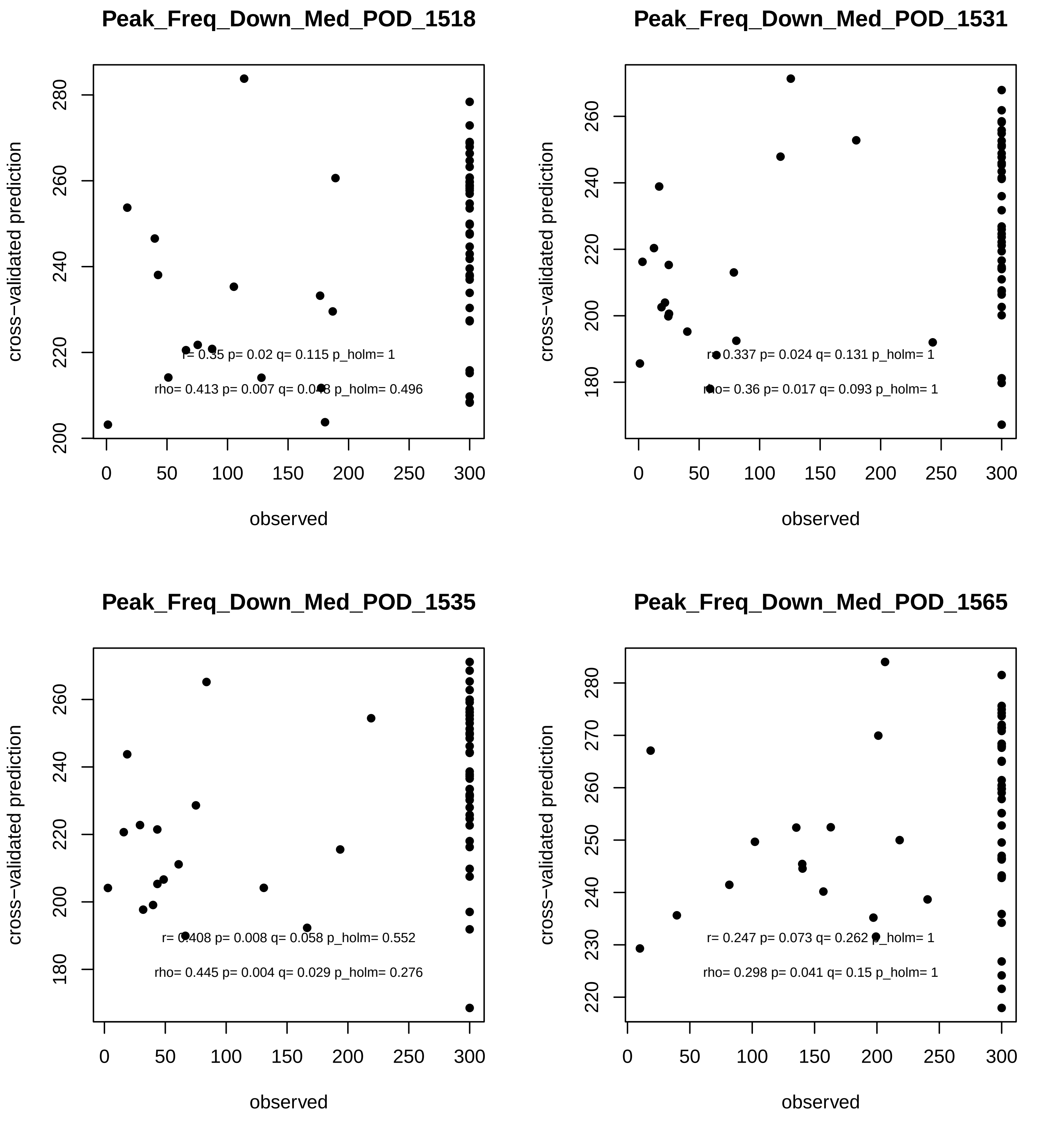
Peak_Freq_Down_Med_POD_1518
cross−validated prediction
observed
r= 0.35 p= 0.02 q= 0.115 p_holm= 1
rho= 0.413 p= 0.007 q= 0.048 p_holm= 0.496
Peak_Freq_Down_Med_POD_1531
cross−validated prediction
observed
r= 0.337 p= 0.024 q= 0.131 p_holm= 1
rho= 0.36 p= 0.017 q= 0.093 p_holm= 1
Peak_Freq_Down_Med_POD_1535
cross−validated prediction
observed
r= 0.408 p= 0.008 q= 0.058 p_holm= 0.552
rho= 0.445 p= 0.004 q= 0.029 p_holm= 0.276
Peak_Freq_Down_Med_POD_1565
cross−validated prediction
observed
r= 0.247 p= 0.073 q= 0.262 p_holm= 1
rho= 0.298 p= 0.041 q= 0.15 p_holm= 1

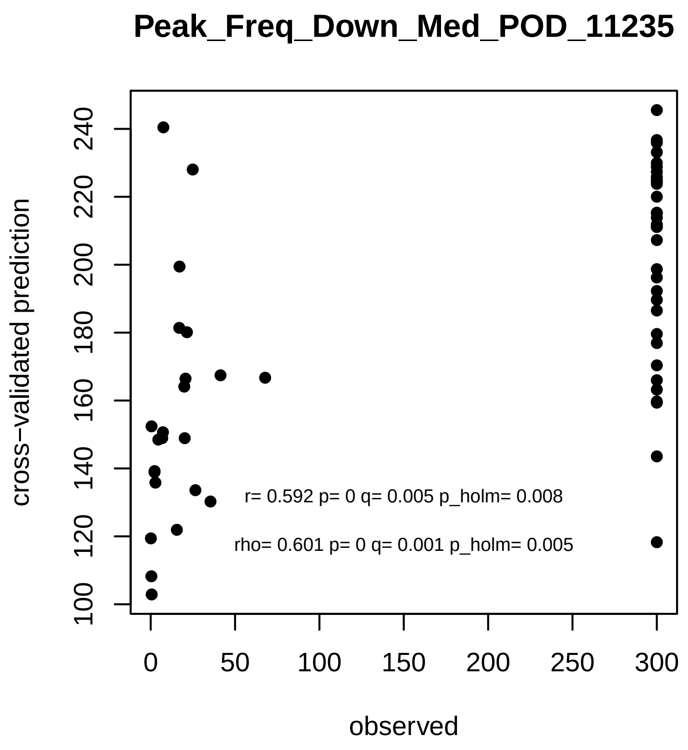
Peak_Freq_Down_Med_POD_11235
r= 0.592 p= 0 q= 0.005 p_holm= 0.008
rho= 0.601 p= 0 q= 0.001 p_holm= 0.005
cross−validated prediction
observed

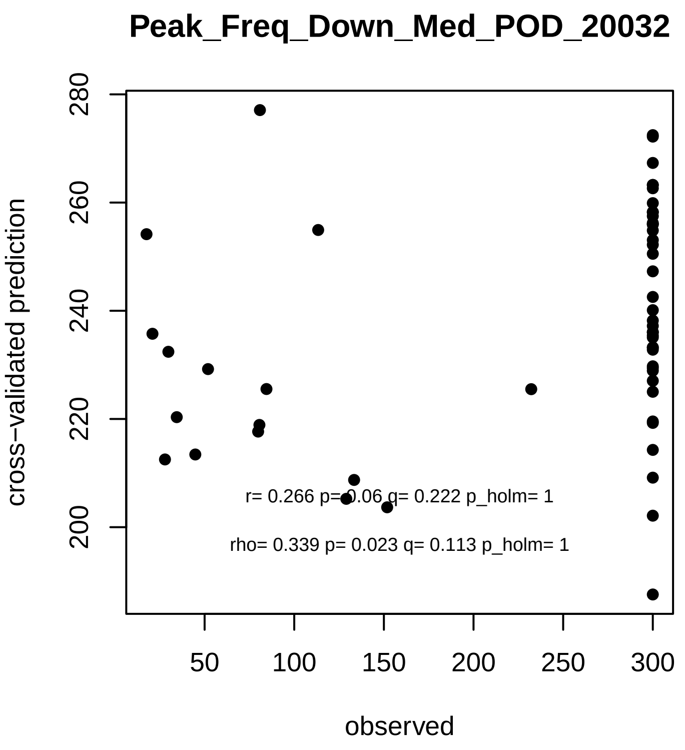
Peak_Freq_Down_Med_POD_20032
r= 0.266 p= 0.06 q= 0.222 p_holm= 1
rho= 0.339 p= 0.023 q= 0.113 p_holm= 1
cross−validated prediction
observed

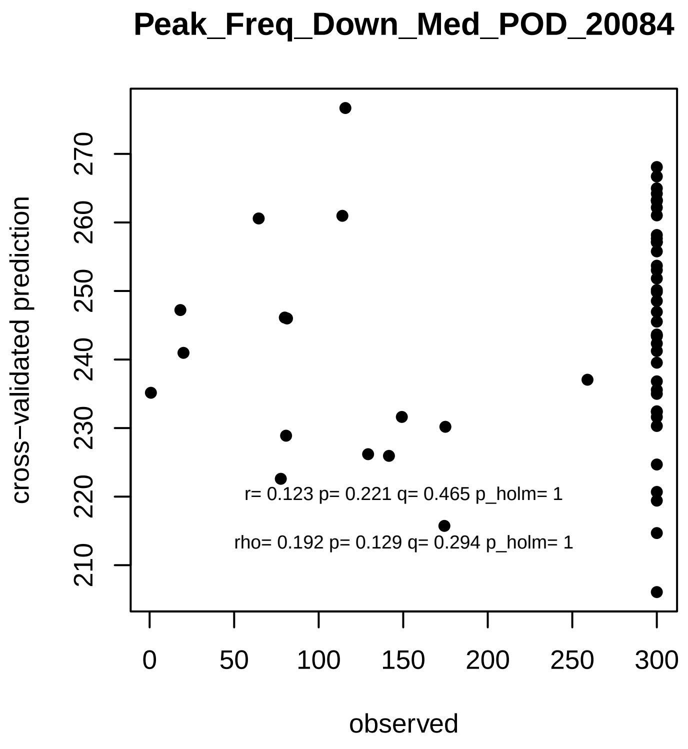
Peak_Freq_Down_Med_POD_20084
r= 0.123 p= 0.221 q= 0.465 p_holm= 1
rho= 0.192 p= 0.129 q= 0.294 p_holm= 1
cross−validated prediction
observed

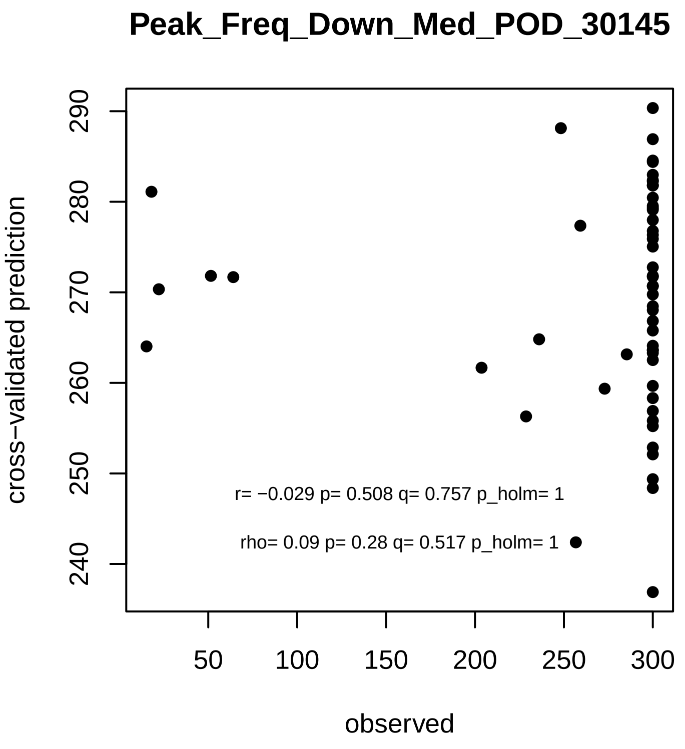
Peak_Freq_Down_Med_POD_30145
r= −0.029 p= 0.508 q= 0.757 p_holm= 1
rho= 0.09 p= 0.28 q= 0.517 p_holm= 1
cross−validated prediction
observed

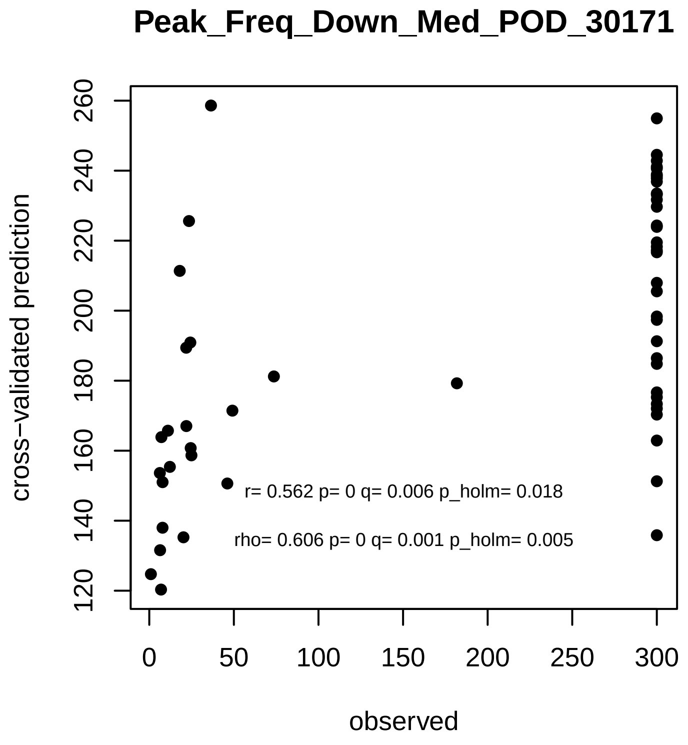
Peak_Freq_Down_Med_POD_30171
cross−validated prediction
observed
r= 0.562 p= 0 q= 0.006 p_holm= 0.018
rho= 0.606 p= 0 q= 0.001 p_holm= 0.005

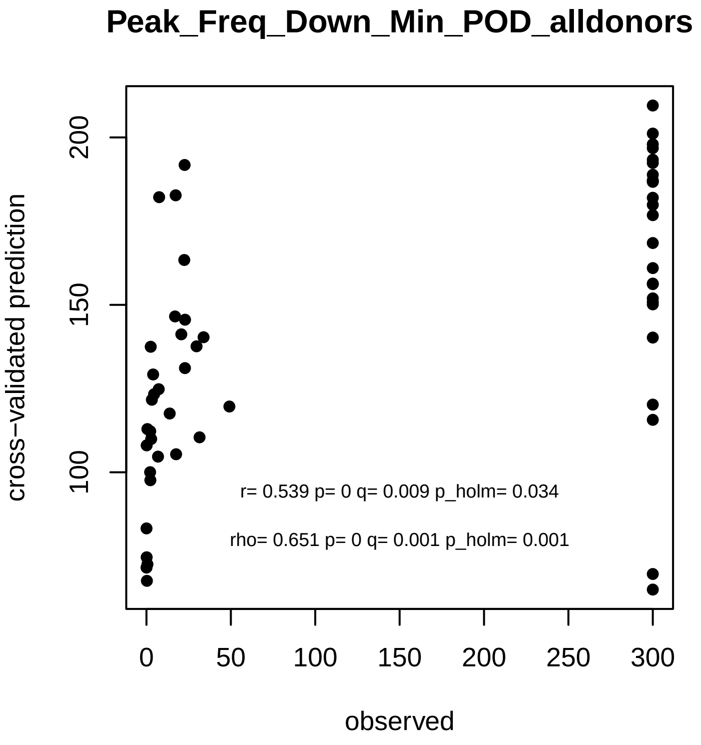
Peak_Freq_Down_Min_POD_alldonors
cross−validated prediction
observed
r= 0.539 p= 0 q= 0.009 p_holm= 0.034
rho= 0.651 p= 0 q= 0.001 p_holm= 0.001

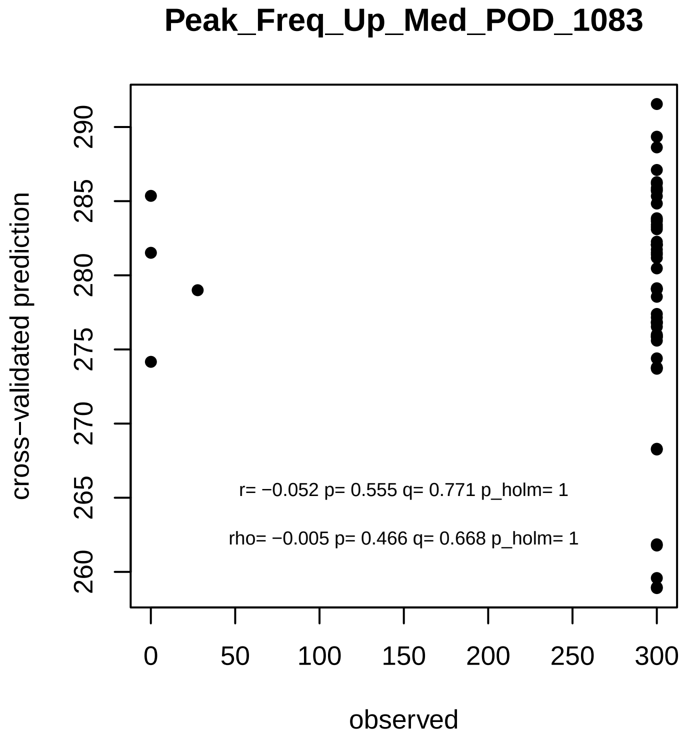
Peak_Freq_Up_Med_POD_1083
cross−validated prediction
observed
r= −0.052 p= 0.555 q= 0.771 p_holm= 1
rho= −0.005 p= 0.466 q= 0.668 p_holm= 1

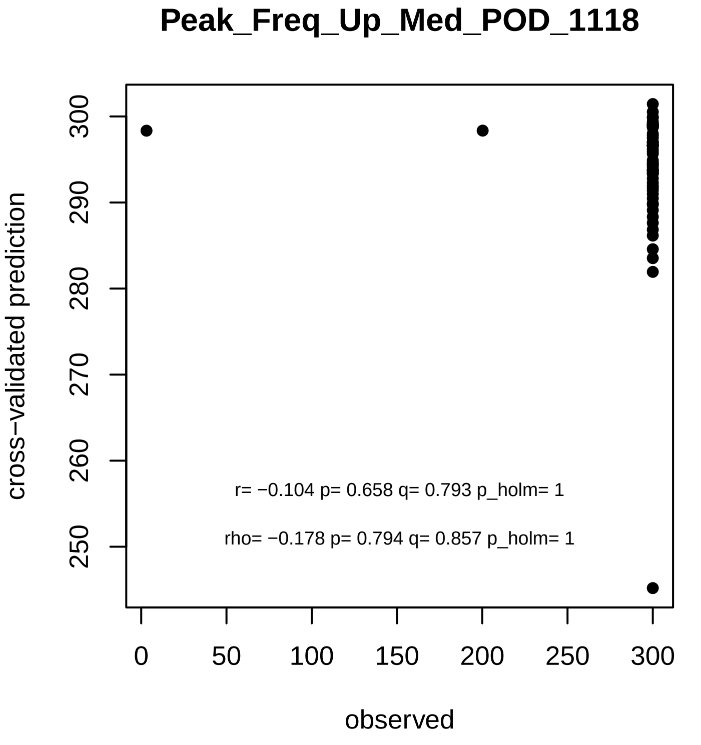
Peak_Freq_Up_Med_POD_1118
cross−validated prediction
observed
r= −0.104 p= 0.658 q= 0.793 p_holm= 1
rho= −0.178 p= 0.794 q= 0.857 p_holm= 1

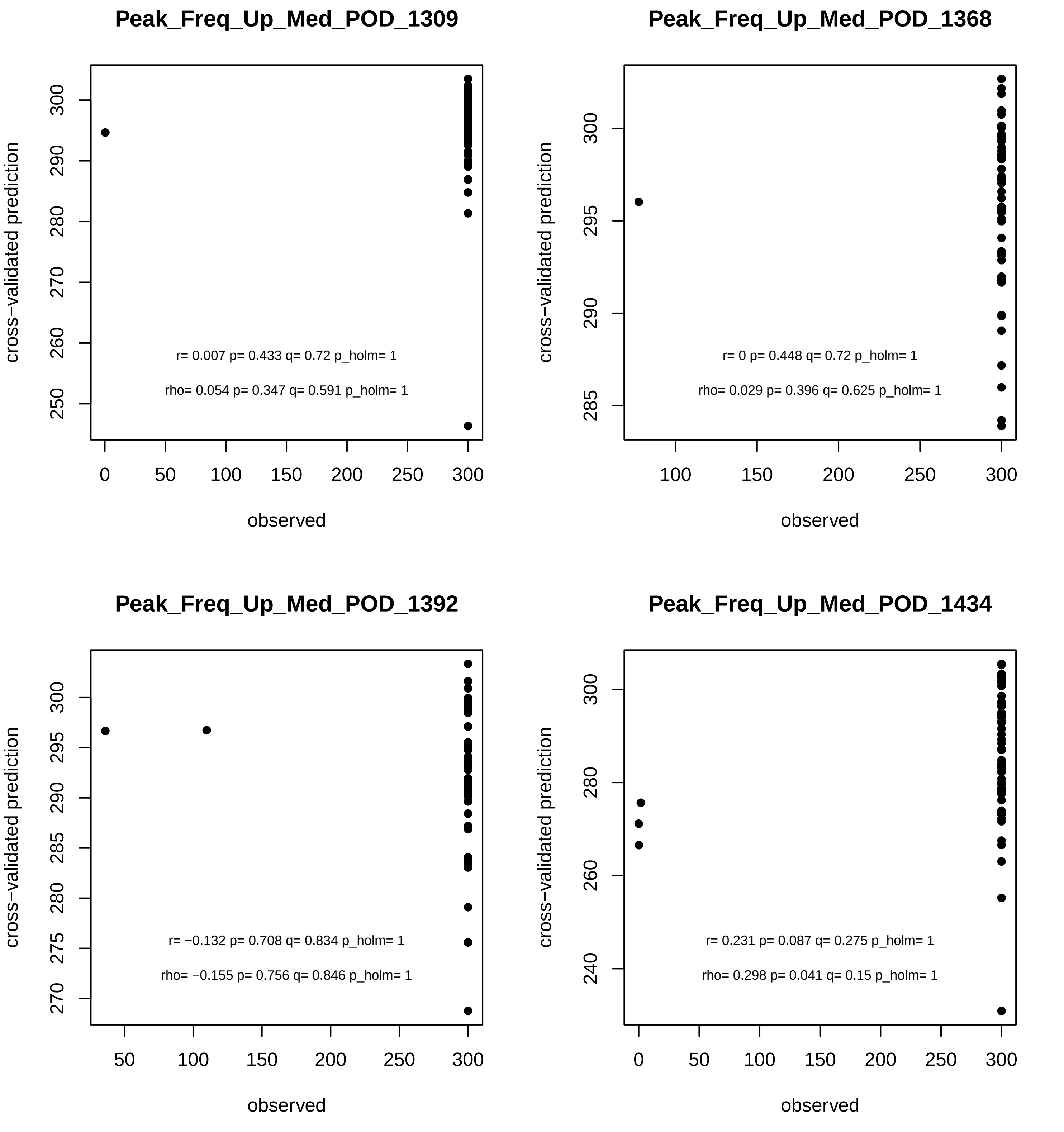
Peak_Freq_Up_Med_POD_1309
r= 0.007 p= 0.433 q= 0.72 p_holm= 1
rho= 0.054 p= 0.347 q= 0.591 p_holm= 1
cross−validated prediction
observed
Peak_Freq_Up_Med_POD_1368
r= 0 p= 0.448 q= 0.72 p_holm= 1
rho= 0.029 p= 0.396 q= 0.625 p_holm= 1
cross−validated prediction
observed
Peak_Freq_Up_Med_POD_1392
r= −0.132 p= 0.708 q= 0.834 p_holm= 1
rho= −0.155 p= 0.756 q= 0.846 p_holm= 1
cross−validated prediction
observed
Peak_Freq_Up_Med_POD_1434
r= 0.231 p= 0.087 q= 0.275 p_holm= 1
rho= 0.298 p= 0.041 q= 0.15 p_holm= 1
cross−validated prediction
observed

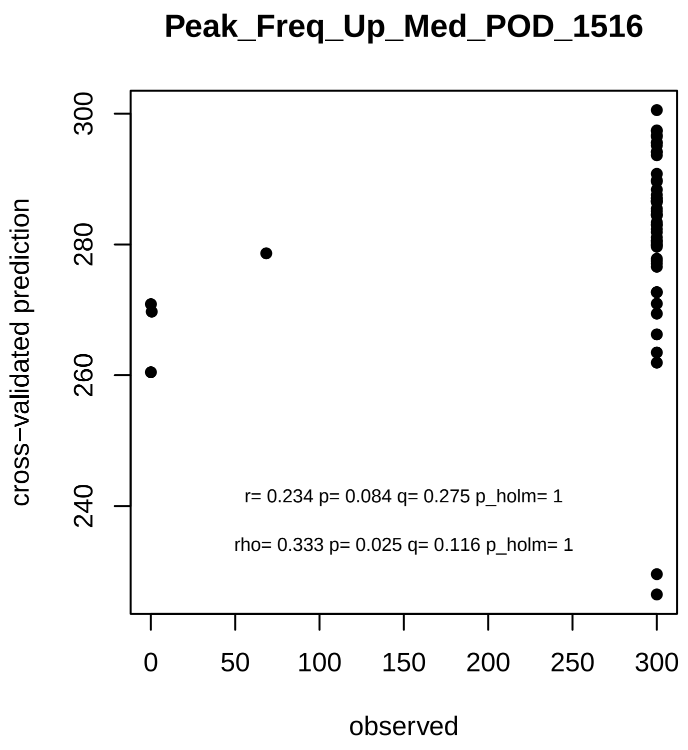
Peak_Freq_Up_Med_POD_1516
r= 0.234 p= 0.084 q= 0.275 p_holm= 1
rho= 0.333 p= 0.025 q= 0.116 p_holm= 1
cross−validated prediction
observed

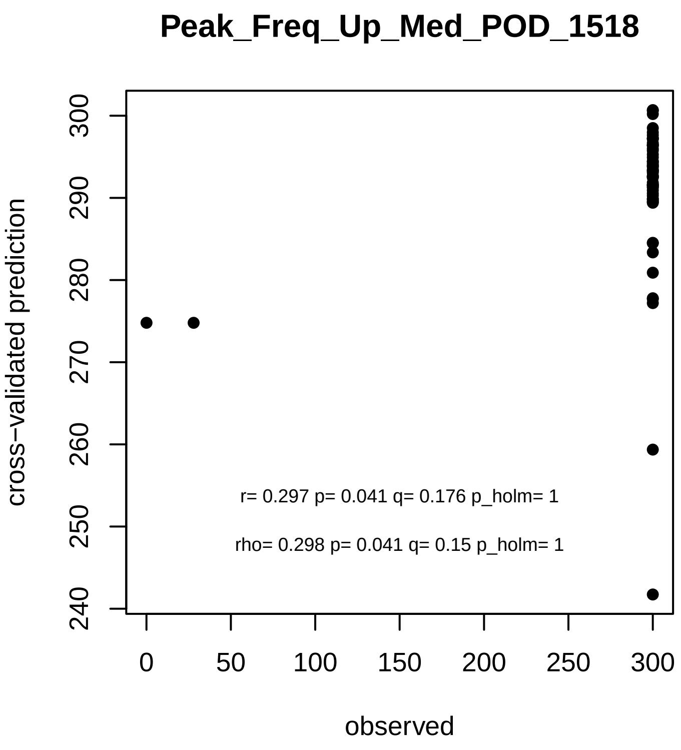
Peak_Freq_Up_Med_POD_1518
r= 0.297 p= 0.041 q= 0.176 p_holm= 1
rho= 0.298 p= 0.041 q= 0.15 p_holm= 1
cross−validated prediction
observed

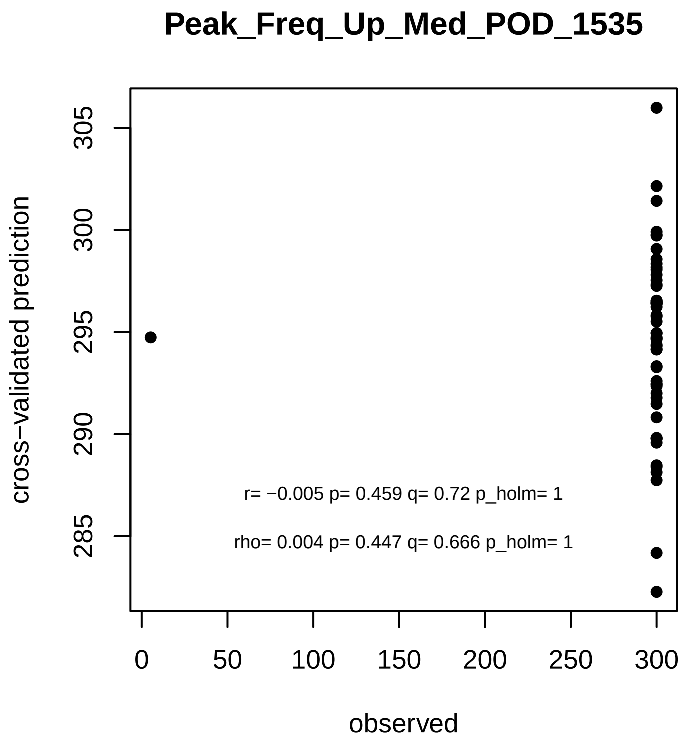
Peak_Freq_Up_Med_POD_1535
r= −0.005 p= 0.459 q= 0.72 p_holm= 1
rho= 0.004 p= 0.447 q= 0.666 p_holm= 1
cross−validated prediction
observed

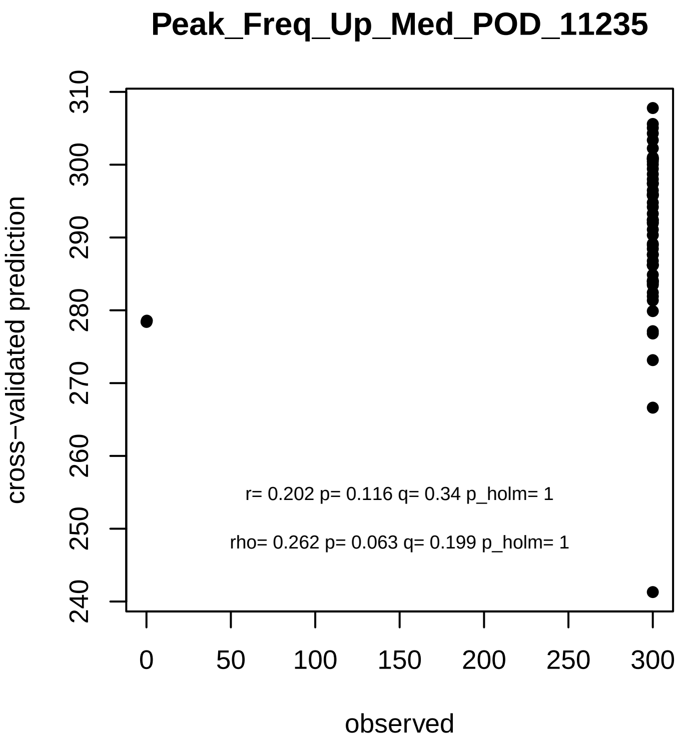
Peak_Freq_Up_Med_POD_11235
r= 0.202 p= 0.116 q= 0.34 p_holm= 1
rho= 0.262 p= 0.063 q= 0.199 p_holm= 1
cross−validated prediction
observed

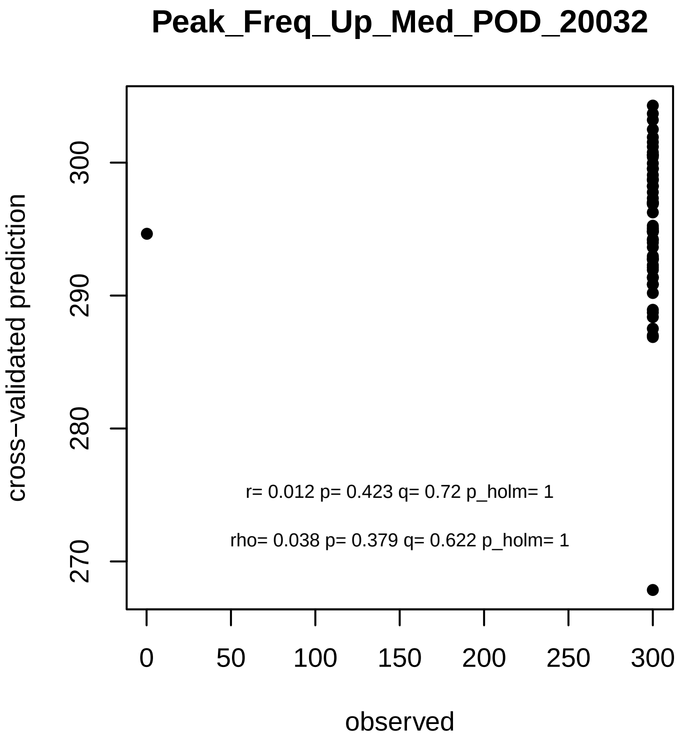
Peak_Freq_Up_Med_POD_20032
cross−validated prediction
observed
r= 0.012 p= 0.423 q= 0.72 p_holm= 1
rho= 0.038 p= 0.379 q= 0.622 p_holm= 1

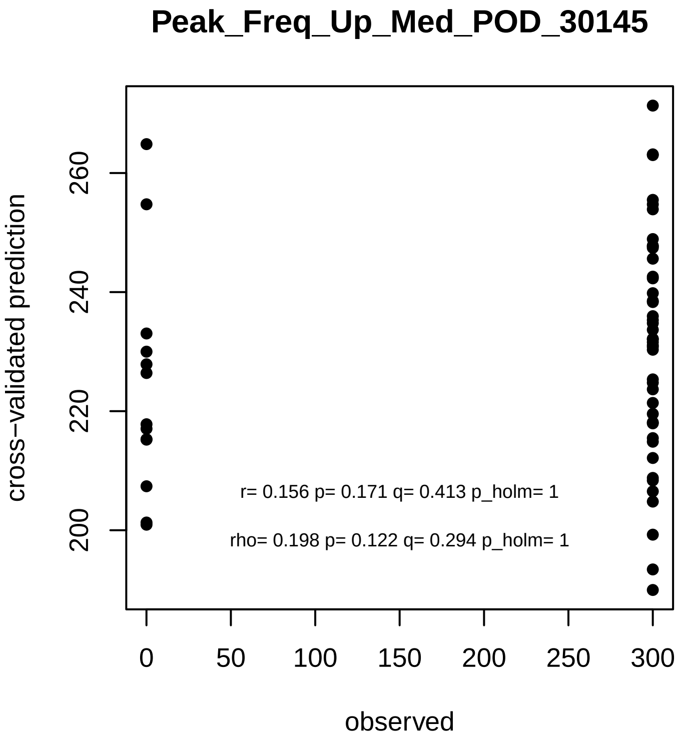
Peak_Freq_Up_Med_POD_30145
cross−validated prediction
observed
r= 0.156 p= 0.171 q= 0.413 p_holm= 1
rho= 0.198 p= 0.122 q= 0.294 p_holm= 1

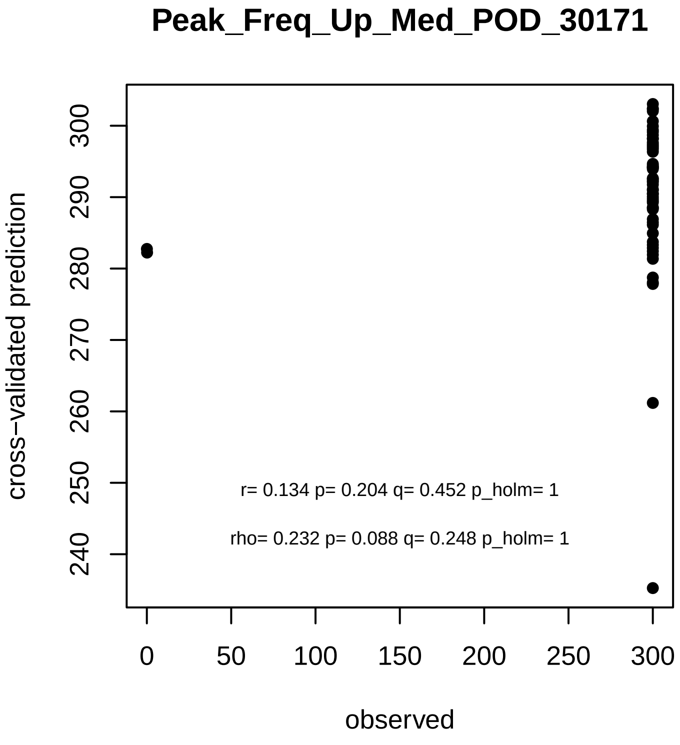
Peak_Freq_Up_Med_POD_30171
cross−validated prediction
observed
r= 0.134 p= 0.204 q= 0.452 p_holm= 1
rho= 0.232 p= 0.088 q= 0.248 p_holm= 1

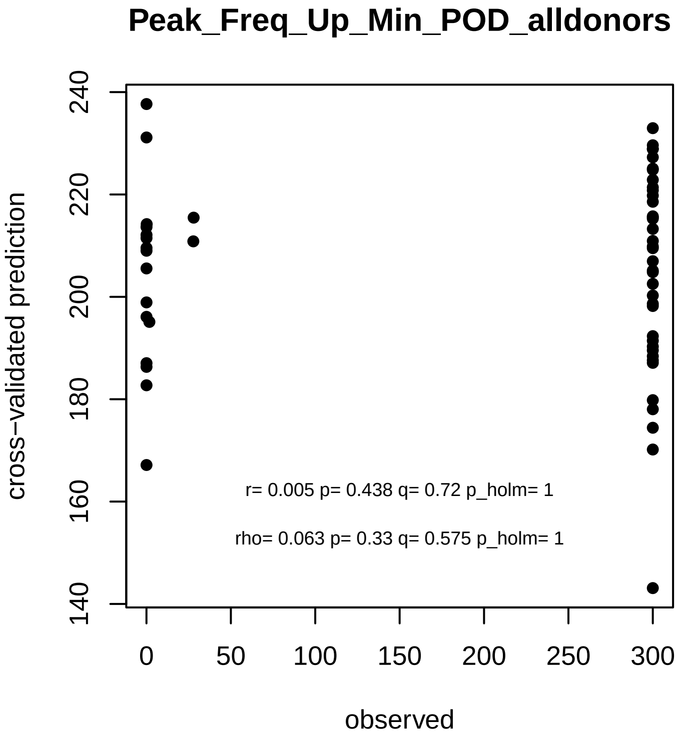
Peak_Freq_Up_Min_POD_alldonors
cross−validated prediction
observed
r= 0.005 p= 0.438 q= 0.72 p_holm= 1
rho= 0.063 p= 0.33 q= 0.575 p_holm= 1

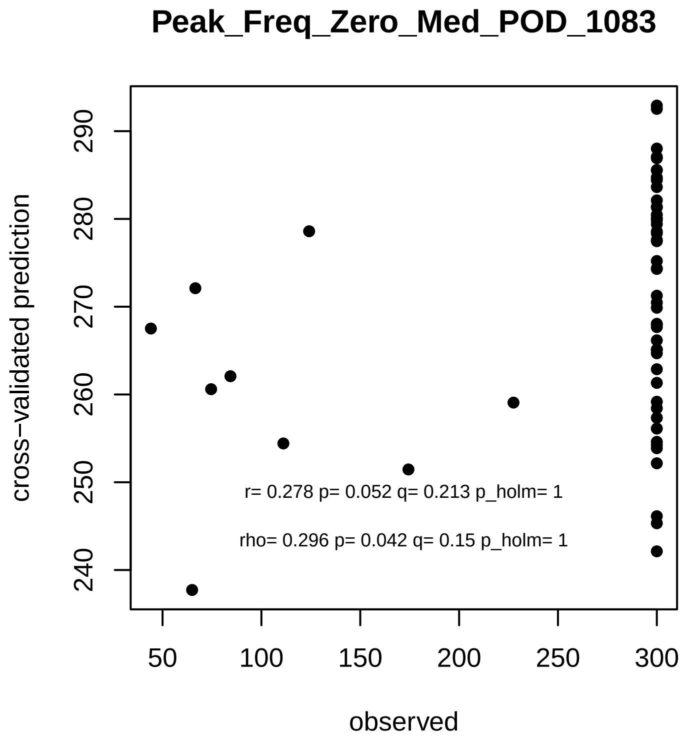
Peak_Freq_Zero_Med_POD_1083
cross−validated prediction
observed
r= 0.278 p= 0.052 q= 0.213 p_holm= 1
rho= 0.296 p= 0.042 q= 0.15 p_holm= 1

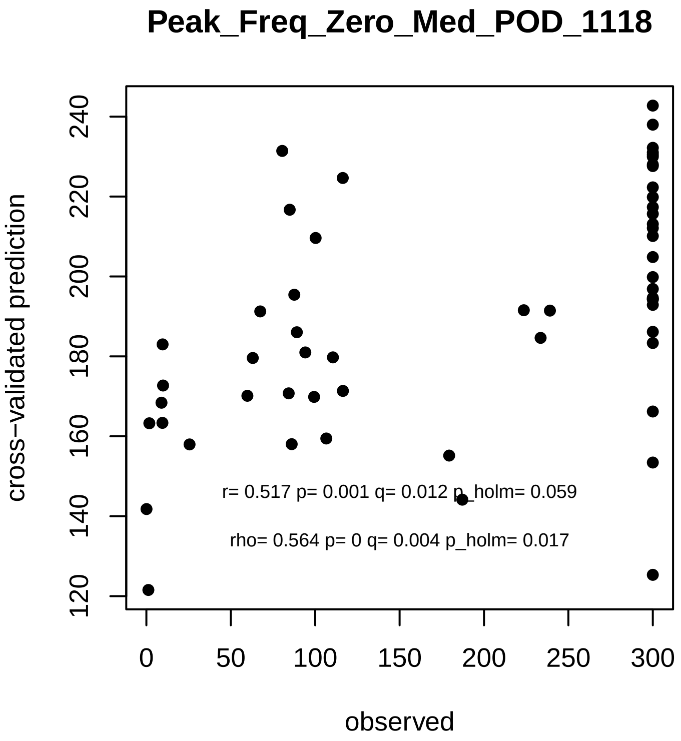
Peak_Freq_Zero_Med_POD_1118
cross−validated prediction
observed
r= 0.517 p= 0.001 q= 0.012 p_holm= 0.059
rho= 0.564 p= 0 q= 0.004 p_holm= 0.017

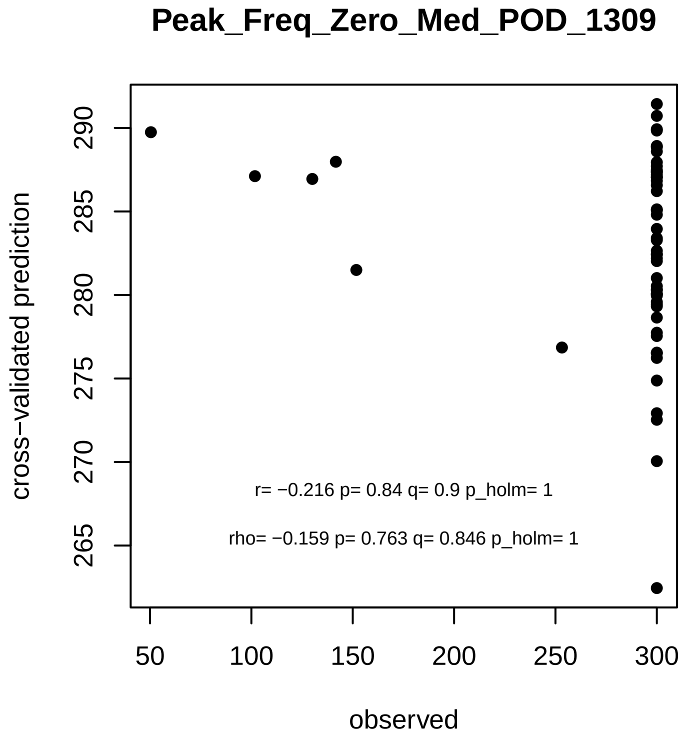
Peak_Freq_Zero_Med_POD_1309
cross−validated prediction
observed
r= −0.216 p= 0.84 q= 0.9 p_holm= 1
rho= −0.159 p= 0.763 q= 0.846 p_holm= 1

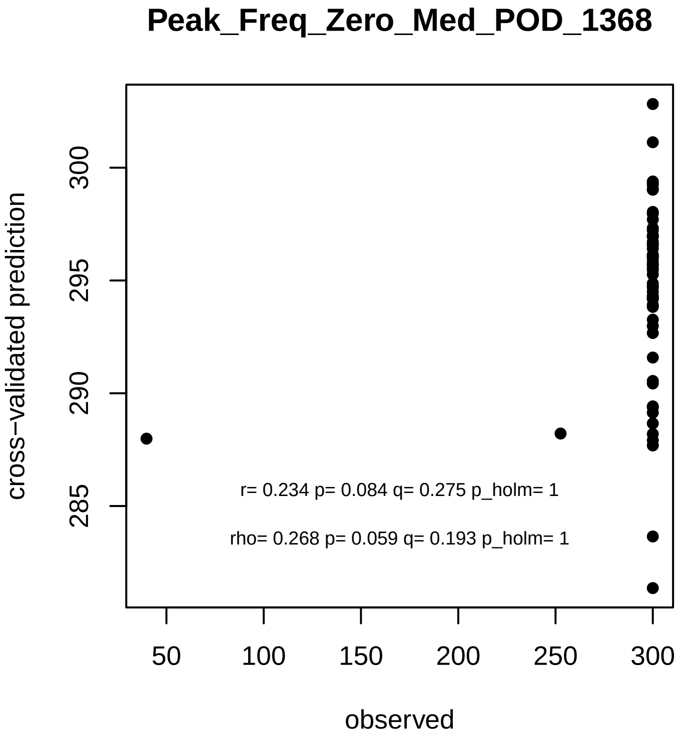
Peak_Freq_Zero_Med_POD_1368
cross−validated prediction
observed
r= 0.234 p= 0.084 q= 0.275 p_holm= 1
rho= 0.268 p= 0.059 q= 0.193 p_holm= 1

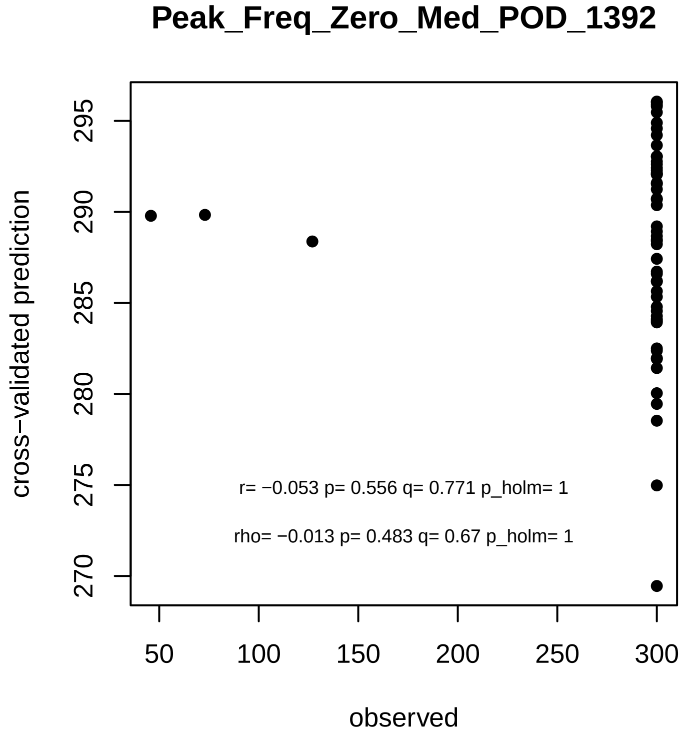
Peak_Freq_Zero_Med_POD_1392
cross−validated prediction
observed
r= −0.053 p= 0.556 q= 0.771 p_holm= 1
rho= −0.013 p= 0.483 q= 0.67 p_holm= 1

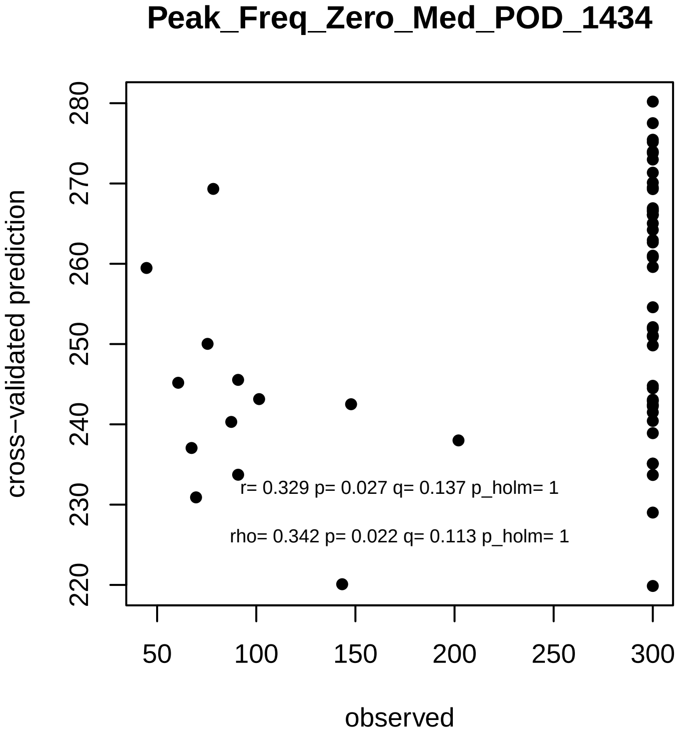
Peak_Freq_Zero_Med_POD_1434
cross−validated prediction
observed
r= 0.329 p= 0.027 q= 0.137 p_holm= 1
rho= 0.342 p= 0.022 q= 0.113 p_holm= 1

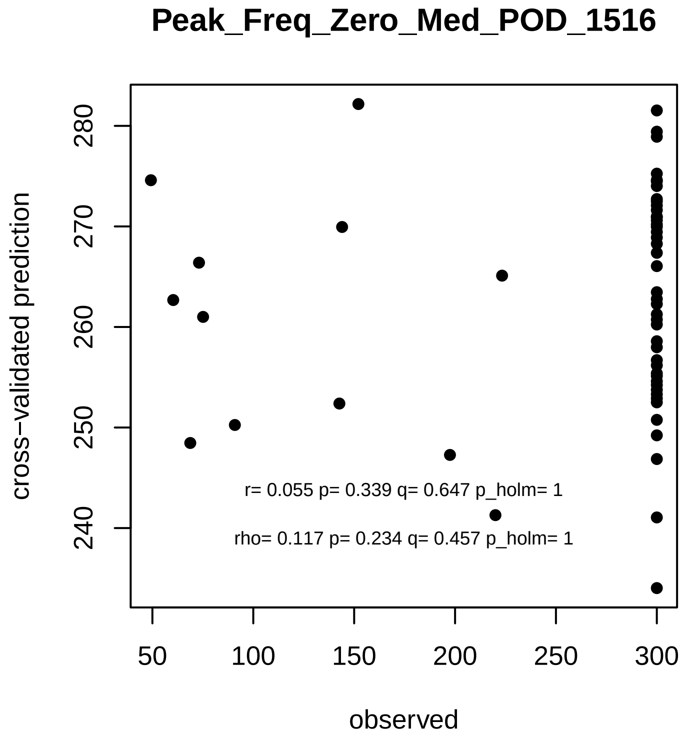
Peak_Freq_Zero_Med_POD_1516
cross−validated prediction
observed
r= 0.055 p= 0.339 q= 0.647 p_holm= 1
rho= 0.117 p= 0.234 q= 0.457 p_holm= 1

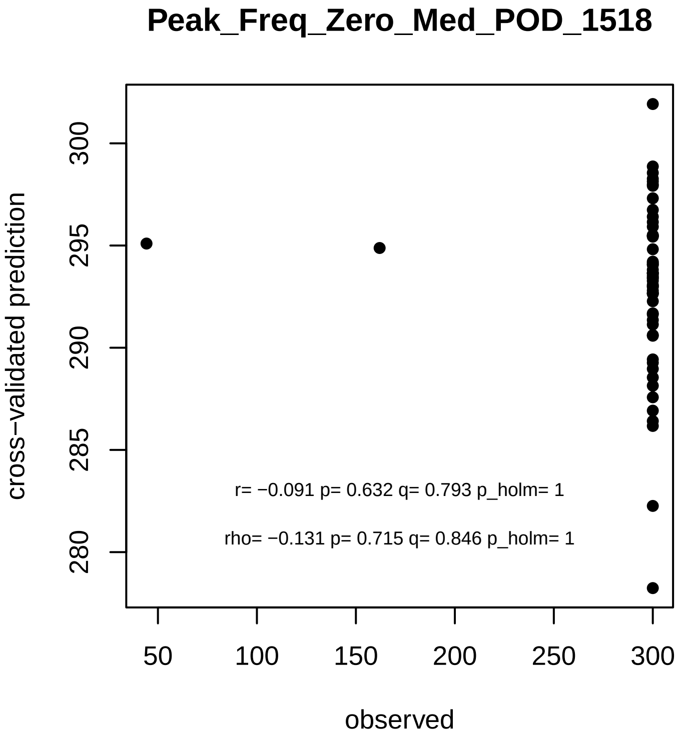
Peak_Freq_Zero_Med_POD_1518
cross−validated prediction
observed
r= −0.091 p= 0.632 q= 0.793 p_holm= 1
rho= −0.131 p= 0.715 q= 0.846 p_holm= 1

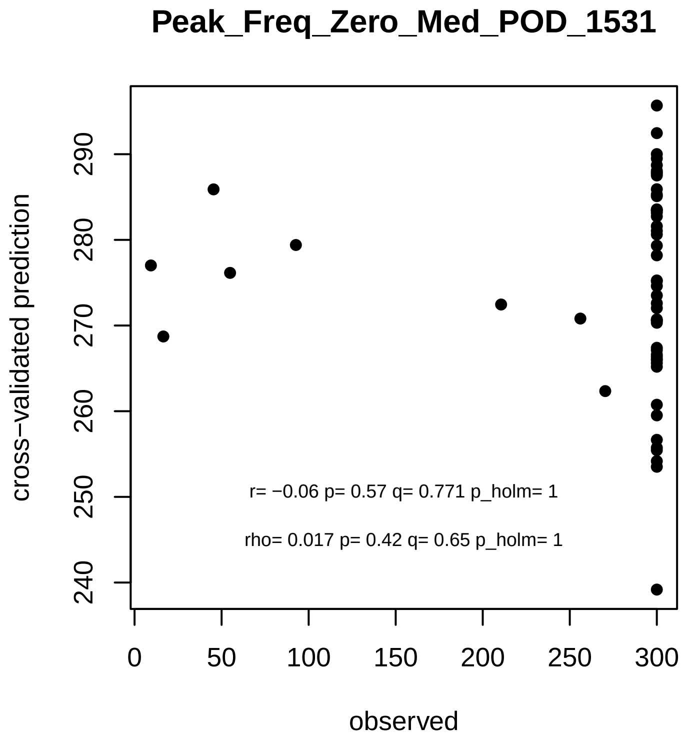
Peak_Freq_Zero_Med_POD_1531
cross−validated prediction
observed
240
250
260
270
280
290
0
50
100
150
200
250
300
r= −0.06 p= 0.57 q= 0.771 p_holm= 1
rho= 0.017 p= 0.42 q= 0.65 p_holm= 1

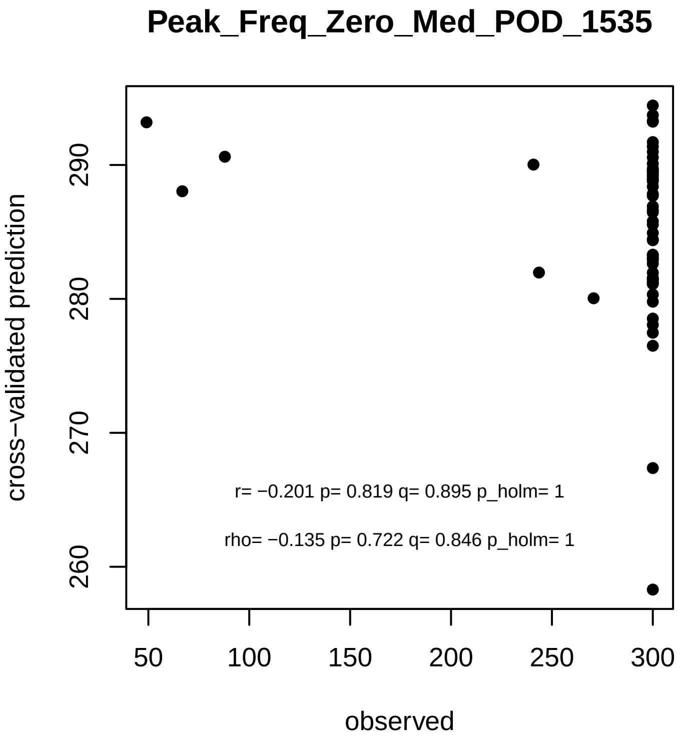
Peak_Freq_Zero_Med_POD_1535
cross−validated prediction
observed
260
270
280
290
50
100
150
200
250
300
r= −0.201 p= 0.819 q= 0.895 p_holm= 1
rho= −0.135 p= 0.722 q= 0.846 p_holm= 1

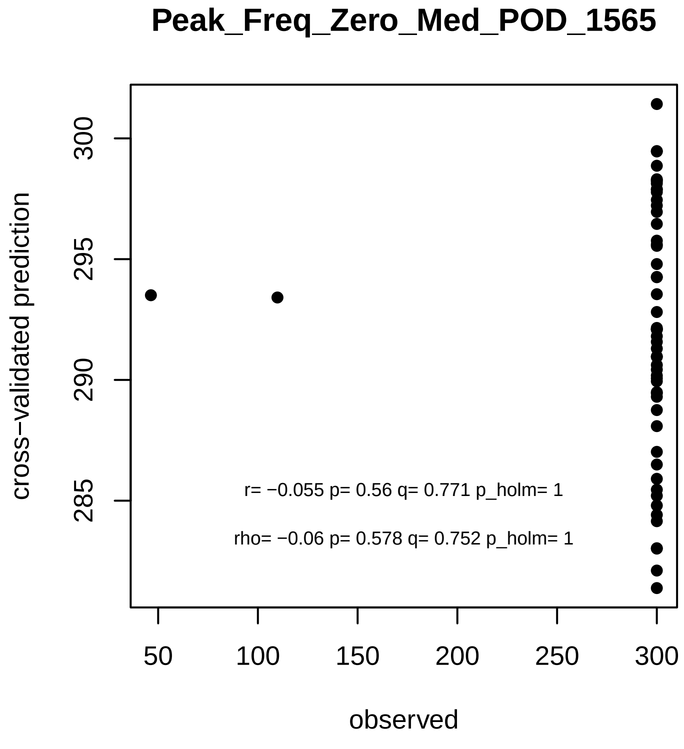
Peak_Freq_Zero_Med_POD_1565
cross−validated prediction
observed
285
290
295
300
50
100
150
200
250
300
r= −0.055 p= 0.56 q= 0.771 p_holm= 1
rho= −0.06 p= 0.578 q= 0.752 p_holm= 1

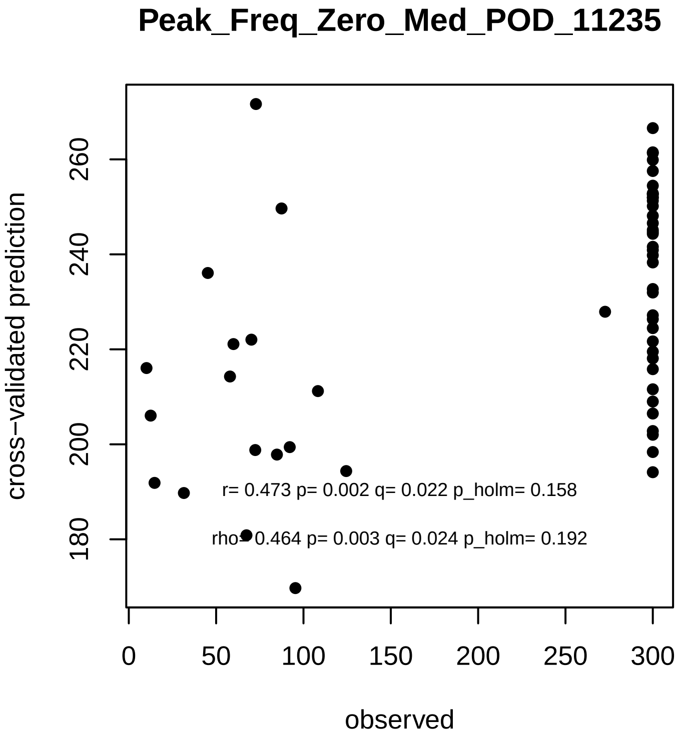
Peak_Freq_Zero_Med_POD_11235
cross−validated prediction
observed
180
200
220
240
260
0
50
100
150
200
250
300
r= 0.473 p= 0.002 q= 0.022 p_holm= 0.158
rho= 0.464 p= 0.003 q= 0.024 p_holm= 0.192

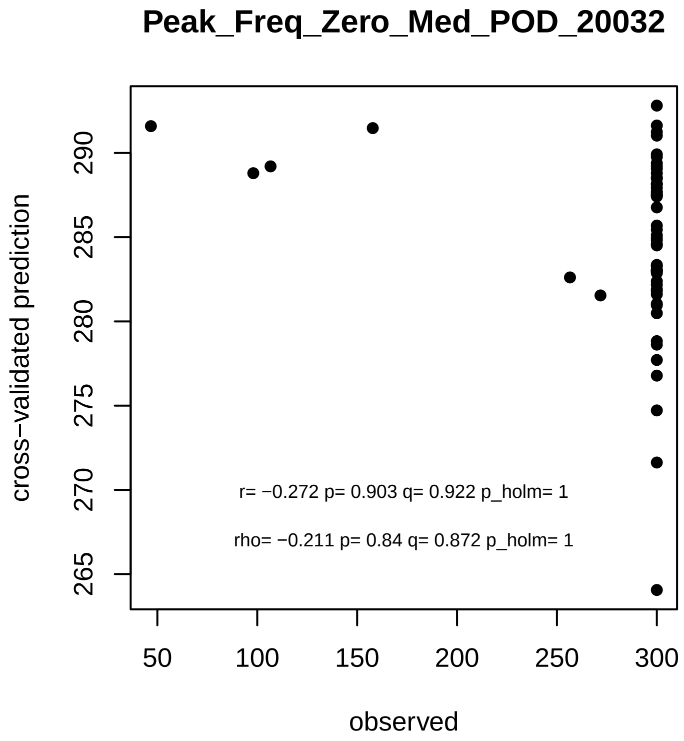
Peak_Freq_Zero_Med_POD_20032
cross−validated prediction
observed
r= −0.272 p= 0.903 q= 0.922 p_holm= 1
rho= −0.211 p= 0.84 q= 0.872 p_holm= 1

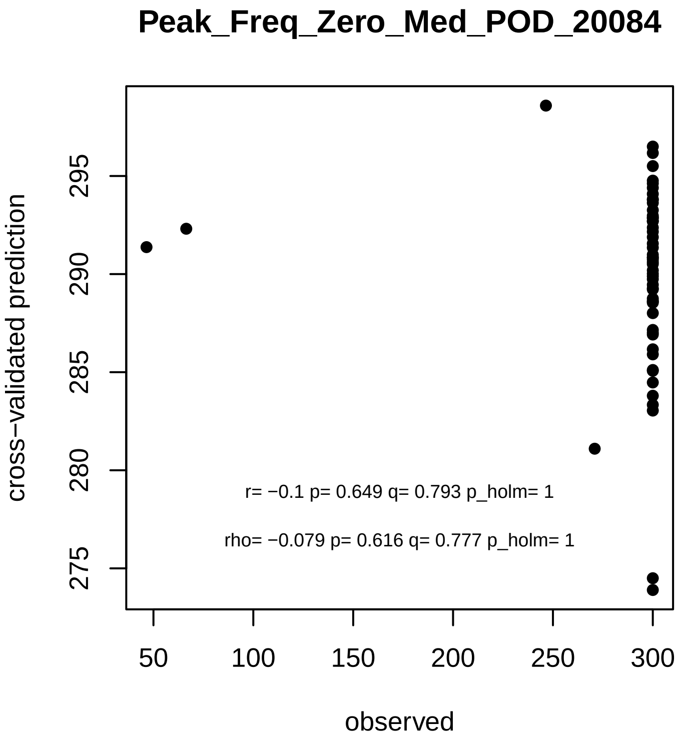
Peak_Freq_Zero_Med_POD_20084
cross−validated prediction
observed
r= −0.1 p= 0.649 q= 0.793 p_holm= 1
rho= −0.079 p= 0.616 q= 0.777 p_holm= 1

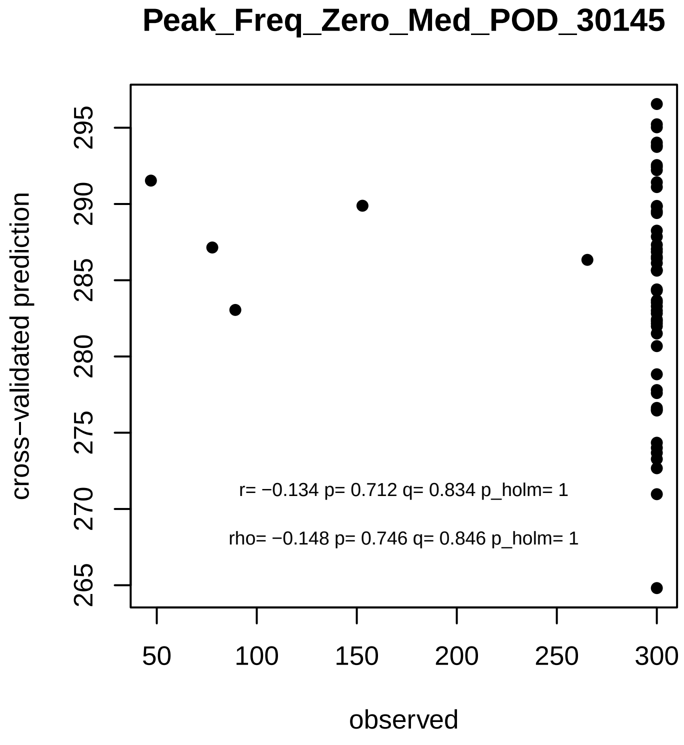
Peak_Freq_Zero_Med_POD_30145
cross−validated prediction
observed
r= −0.134 p= 0.712 q= 0.834 p_holm= 1
rho= −0.148 p= 0.746 q= 0.846 p_holm= 1

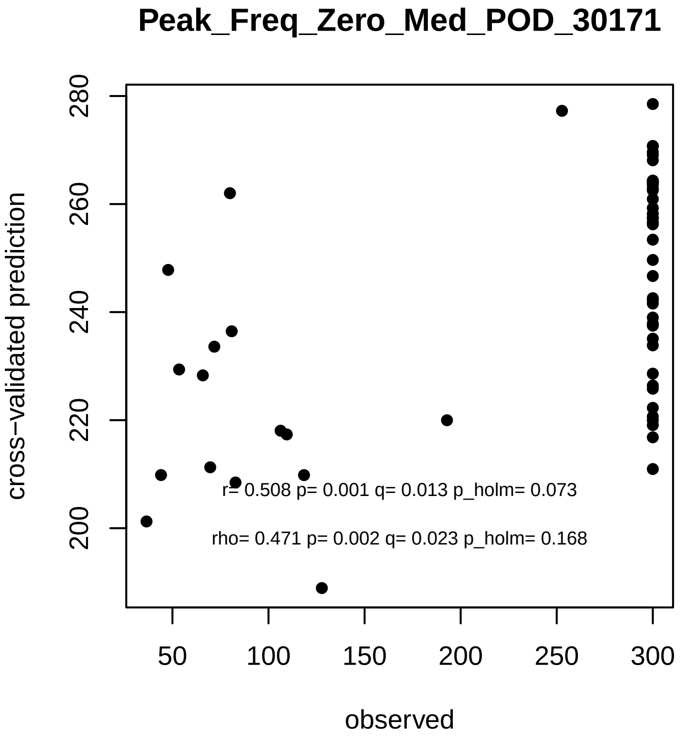
Peak_Freq_Zero_Med_POD_30171
cross−validated prediction
observed
r= 0.508 p= 0.001 q= 0.013 p_holm= 0.073
rho= 0.471 p= 0.002 q= 0.023 p_holm= 0.168

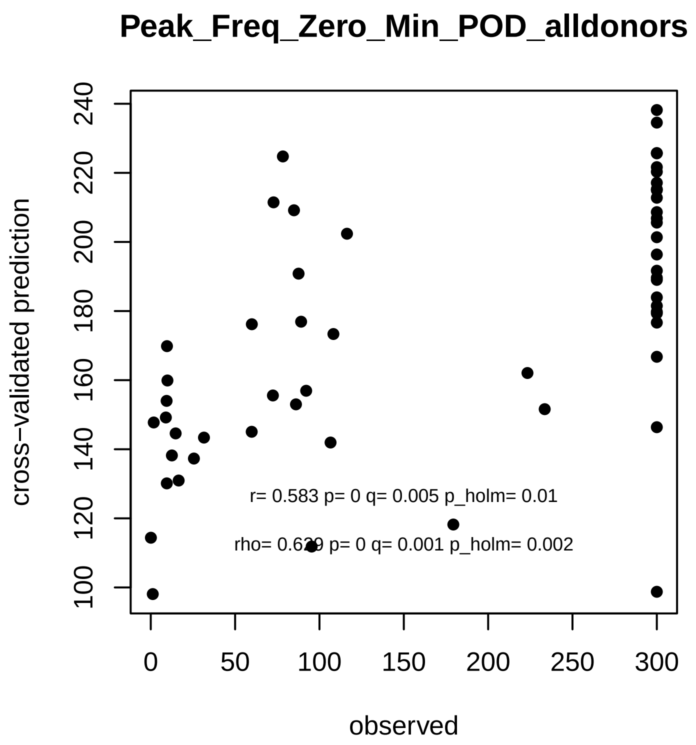
Peak_Freq_Zero_Min_POD_alldonors
r= 0.583 p= 0 q= 0.005 p_holm= 0.01
p= 0 q= 0.001 p_holm= 0.002
observed
cross−validated prediction

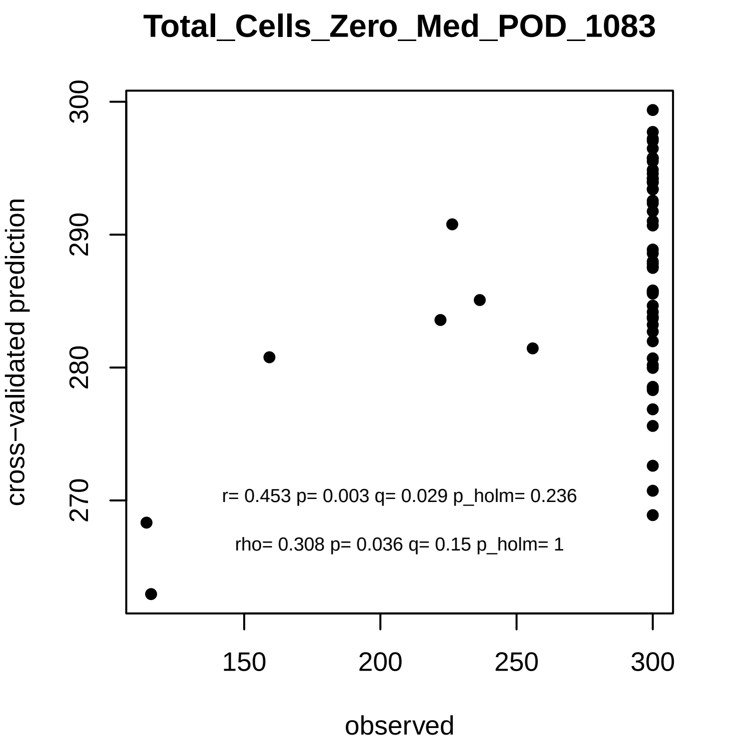
Total_Cells_Zero_Med_POD_1083
r= 0.453 p= 0.003 q= 0.029 p_holm= 0.236
rho= 0.308 p= 0.036 q= 0.15 p_holm= 1
observed
cross−validated prediction

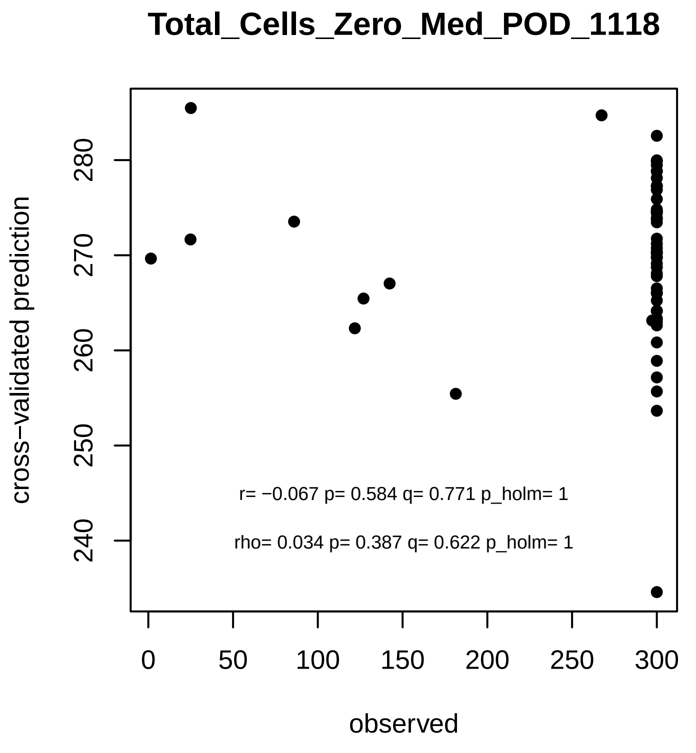
Total_Cells_Zero_Med_POD_1118
r= −0.067 p= 0.584 q= 0.771 p_holm= 1
rho= 0.034 p= 0.387 q= 0.622 p_holm= 1
observed
cross−validated prediction

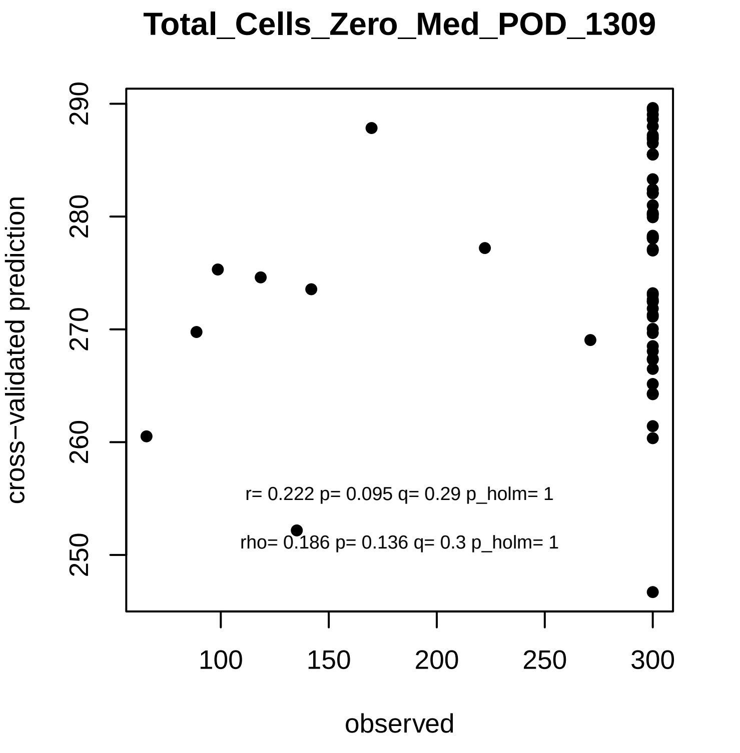
Total_Cells_Zero_Med_POD_1309
r= 0.222 p= 0.095 q= 0.29 p_holm= 1
rho= 0.186 p= 0.136 q= 0.3 p_holm= 1
observed
cross−validated prediction

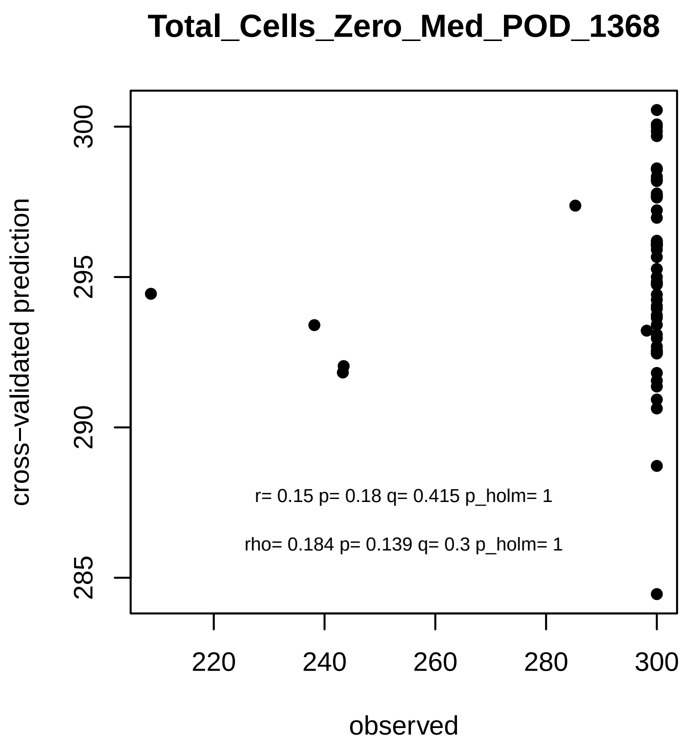
Total_Cells_Zero_Med_POD_1368
r= 0.15 p= 0.18 q= 0.415 p_holm= 1
rho= 0.184 p= 0.139 q= 0.3 p_holm= 1
cross−validated prediction
observed

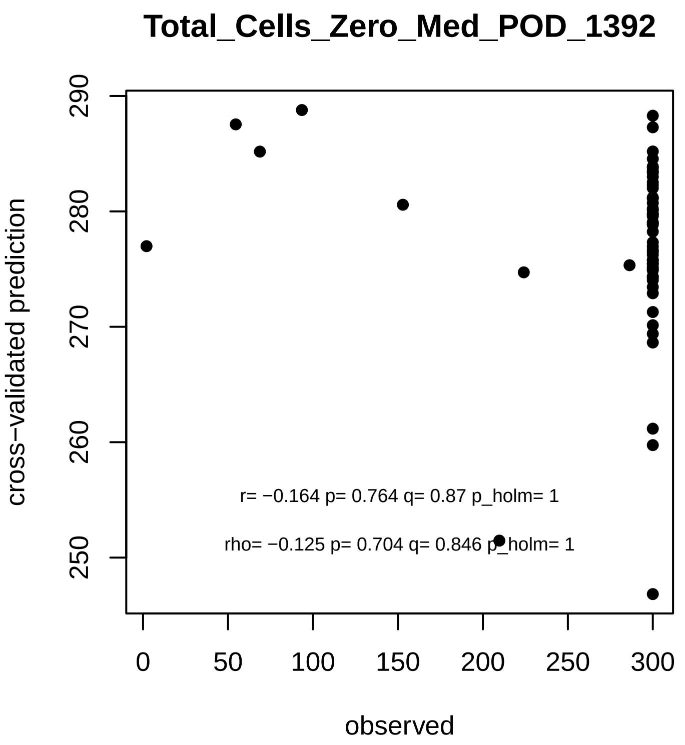
Total_Cells_Zero_Med_POD_1392
r= −0.164 p= 0.764 q= 0.87 p_holm= 1
rho= −0.125 p= 0.704 q= 0.846 p_holm= 1
cross−validated prediction
observed

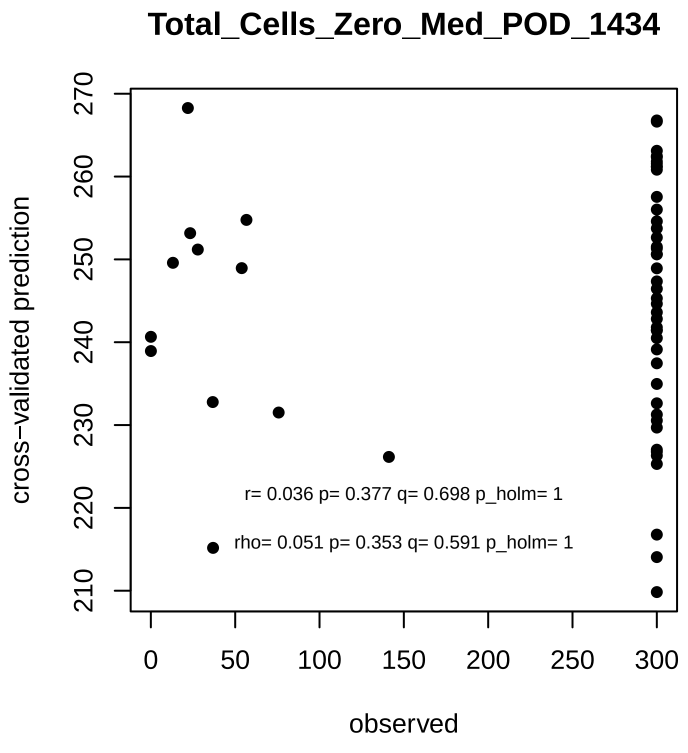
Total_Cells_Zero_Med_POD_1434
r= 0.036 p= 0.377 q= 0.698 p_holm= 1
rho= 0.051 p= 0.353 q= 0.591 p_holm= 1
cross−validated prediction
observed

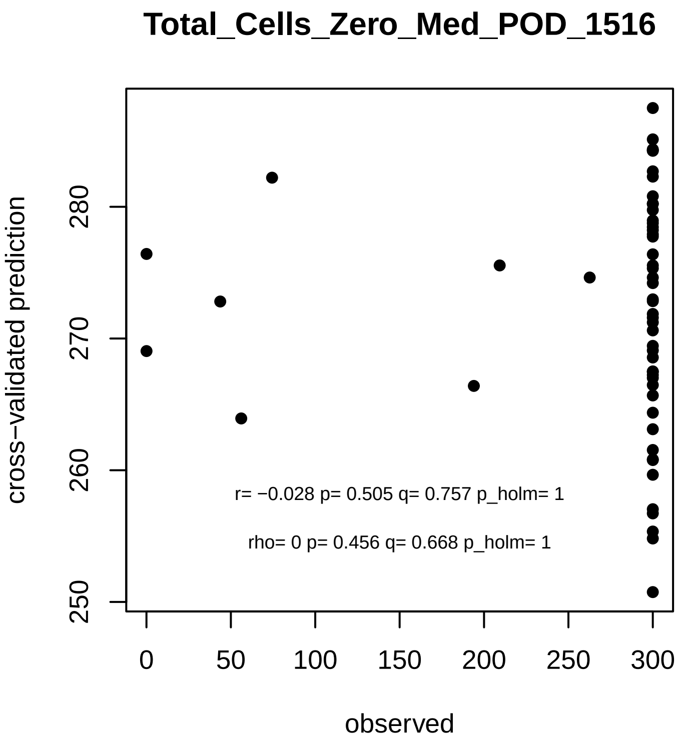
Total_Cells_Zero_Med_POD_1516
r= −0.028 p= 0.505 q= 0.757 p_holm= 1
rho= 0 p= 0.456 q= 0.668 p_holm= 1
cross−validated prediction
observed

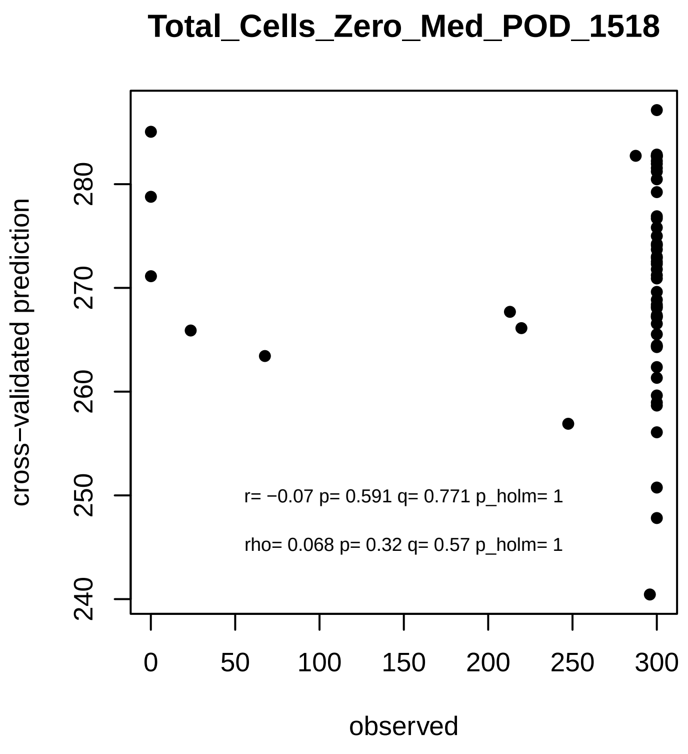
Total_Cells_Zero_Med_POD_1518
cross−validated prediction
observed
r= −0.07 p= 0.591 q= 0.771 p_holm= 1
rho= 0.068 p= 0.32 q= 0.57 p_holm= 1

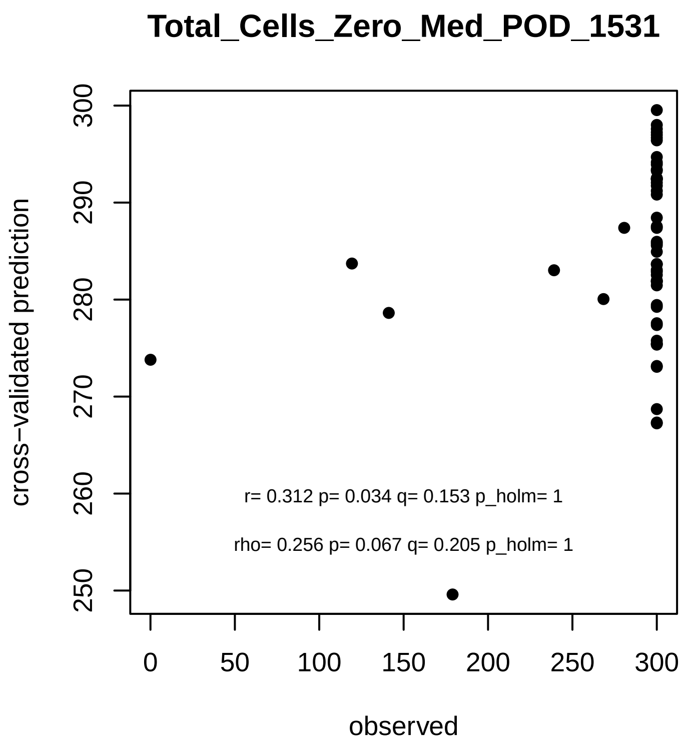
Total_Cells_Zero_Med_POD_1531
cross−validated prediction
observed
r= 0.312 p= 0.034 q= 0.153 p_holm= 1
rho= 0.256 p= 0.067 q= 0.205 p_holm= 1

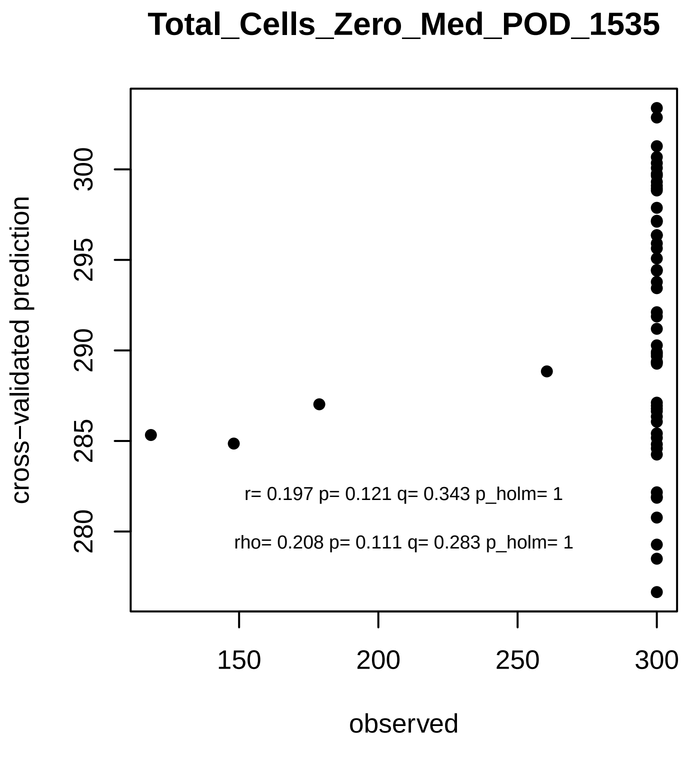
Total_Cells_Zero_Med_POD_1535
cross−validated prediction
observed
r= 0.197 p= 0.121 q= 0.343 p_holm= 1
rho= 0.208 p= 0.111 q= 0.283 p_holm= 1

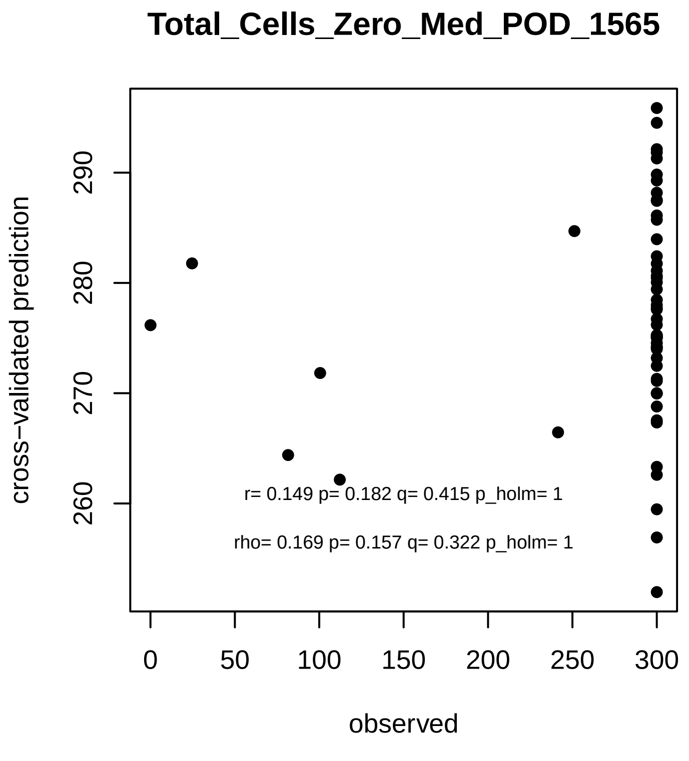
Total_Cells_Zero_Med_POD_1565
cross−validated prediction
observed
r= 0.149 p= 0.182 q= 0.415 p_holm= 1
rho= 0.169 p= 0.157 q= 0.322 p_holm= 1

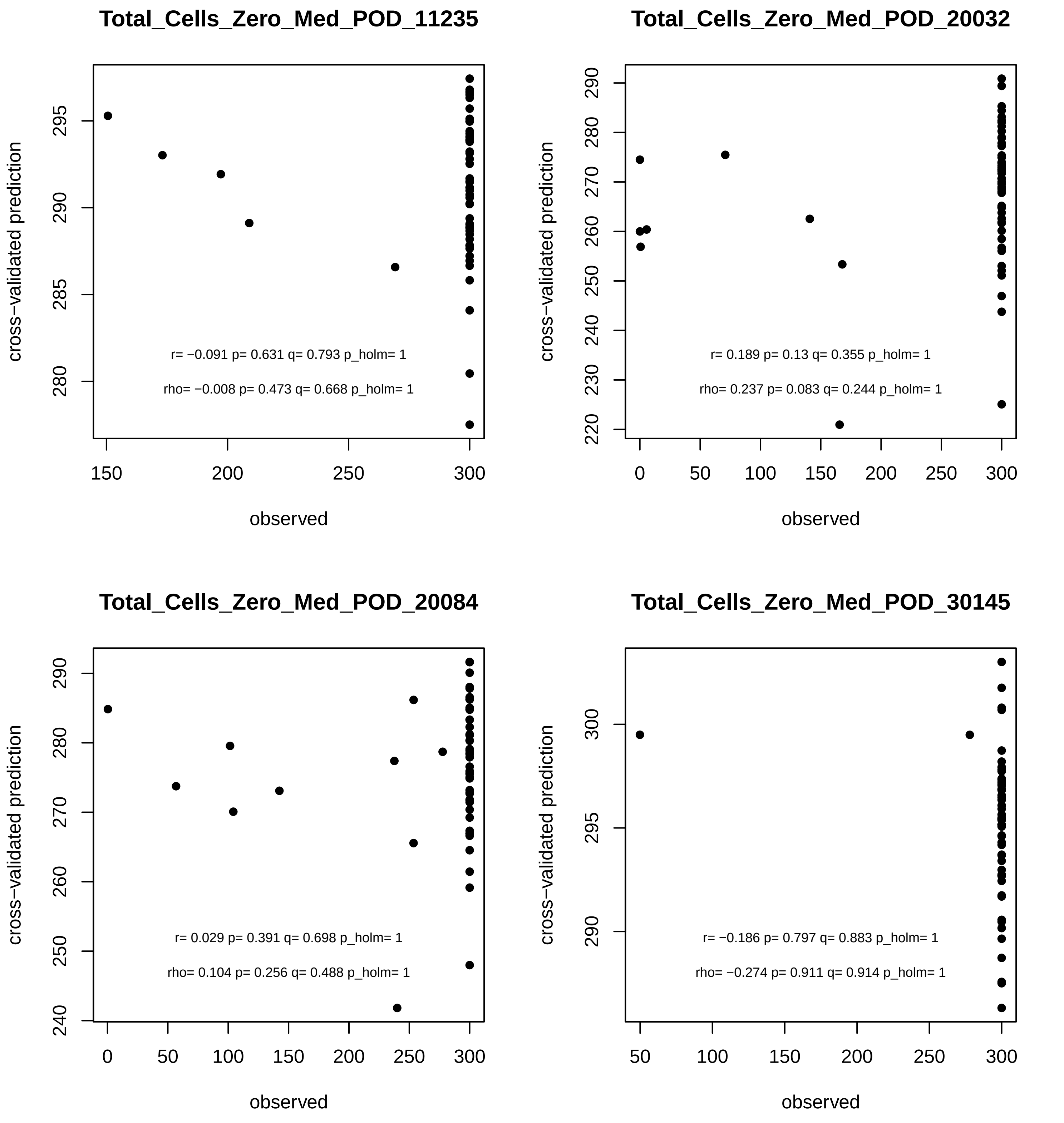
Total_Cells_Zero_Med_POD_11235
r= −0.091 p= 0.631 q= 0.793 p_holm= 1
rho= −0.008 p= 0.473 q= 0.668 p_holm= 1
cross−validated prediction
observed
Total_Cells_Zero_Med_POD_20032
r= 0.189 p= 0.13 q= 0.355 p_holm= 1
rho= 0.237 p= 0.083 q= 0.244 p_holm= 1
cross−validated prediction
observed
Total_Cells_Zero_Med_POD_20084
r= 0.029 p= 0.391 q= 0.698 p_holm= 1
rho= 0.104 p= 0.256 q= 0.488 p_holm= 1
cross−validated prediction
observed
Total_Cells_Zero_Med_POD_30145
r= −0.186 p= 0.797 q= 0.883 p_holm= 1
rho= −0.274 p= 0.911 q= 0.914 p_holm= 1
cross−validated prediction
observed

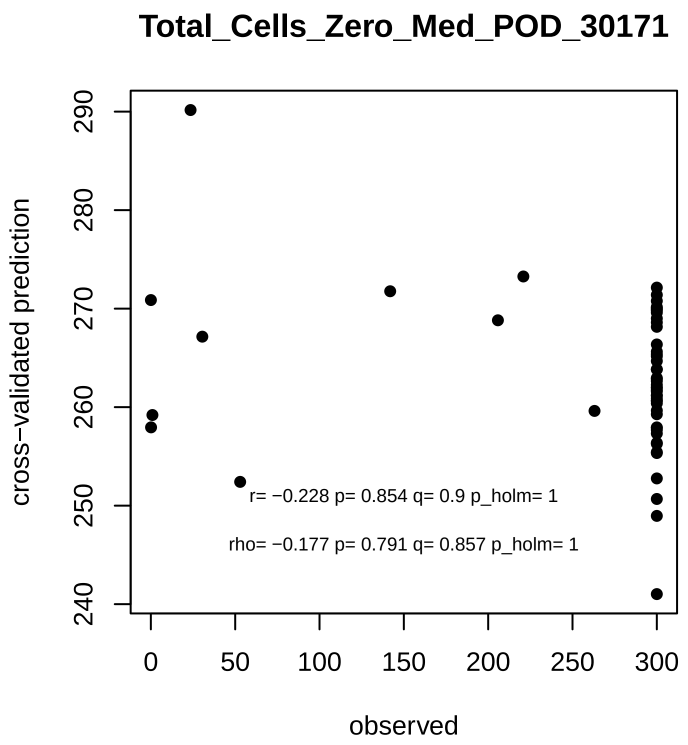
Total_Cells_Zero_Med_POD_30171
cross−validated prediction
observed
r= −0.228 p= 0.854 q= 0.9 p_holm= 1
rho= −0.177 p= 0.791 q= 0.857 p_holm= 1

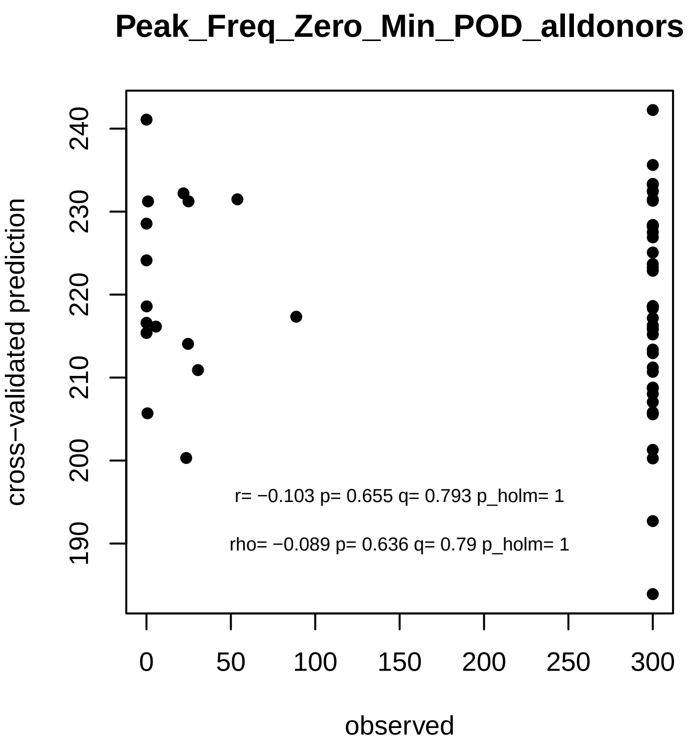
Peak_Freq_Zero_Min_POD_alldonors
cross−validated prediction
observed
r= −0.103 p= 0.655 q= 0.793 p_holm= 1
rho= −0.089 p= 0.636 q= 0.79 p_holm= 1
