# Supplementary material for: Characterizing PFAS hazards and risks: a human population-based in vitro cardiotoxicity assessment strategy
Source: Hum Genomics. 2024 Sep 2;18:92. doi: 10.1186/s40246-024-00665-x (PMC11368000; doi:10.1186/s40246-024-00665-x)

Figure S7. Structural features that were found to be significantly associated with PFAS bioactivity. Shown below are PFAS structures highlighting the Saagar structural features that are listed in Table 3.

TABLE OF CONTENTS

Descriptor Category	SAAGAR Descriptor ID	Number of Chemicals	Page #
ATOMS	SGR10068	9	1-6
	SGR10032	3	7-8
ATOM PAIRS	SGR10786	8	10-13
	SGR10112	7	14-17
	SGR10199	3	18-19
BIOAVAILIBILITY	SGR10633	17	21-29
	SGR10708	17	30-38
	SGR10275	12	39-44
	SGR10169	8	45-48
	SGR10795	4	49-50
	SGR10290	3	51-52
	SGR10418	3	53-54
	SGR10493	3	55-56
	SGR10684	3	57-58
	SGR10786	3	59-60
	SGR10354	2	61
	SGR10668	2	62
	SGR10703	2	63
FUNCTIONAL GROUPS	SGR10072	14	65-71
	SGR10761	14	72-78
	SGR10295	12	79-84
	SGR10203	8	85-88
	SGR10153	7	89-92
	SGR10109	3	93-94
	SGR10343	3	95-96
	SGR10099	2	97
	SGR10289	2	98
	SGR10587	2	99
TOPOLOGY	SGR10704	2	101
	SGR10749	2	102

ATOMS

SGR10068 (9 chem)
SGR10032 (3 chem)

SGR10068

S3: Perfluoro-3,6-dioxaoctane-1,8-dioic acid (PFHx2Et2OA)
SMILES: OC(=O)C(F)(F)OC(F)(F)C(F)(F)OC(F)(F)C(O)=O

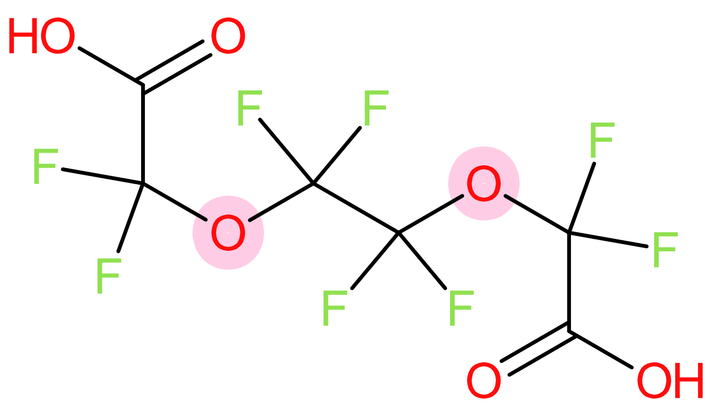

S9: Perfluoro(4-methoxybutanoic) acid (PFMBA)
SMILES: OC(=O)C(F)(F)C(F)(F)C(F)(F)OC(F)(F)F

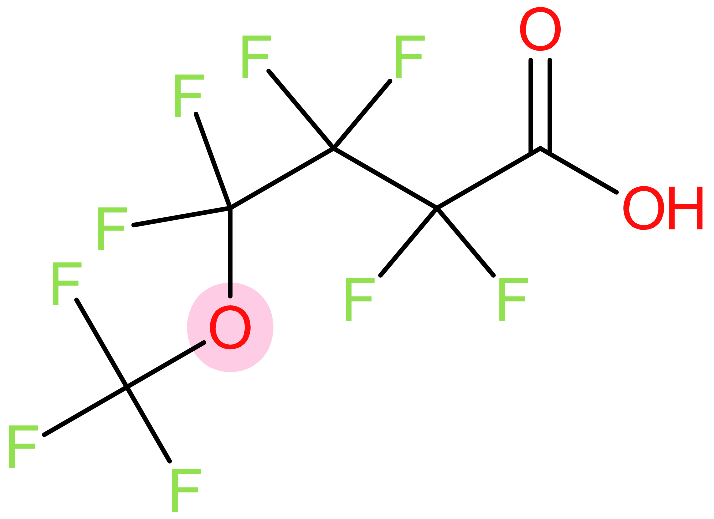

S11: Perfluoro-3,6-dioxaheptanoic acid (NFDHA)
SMILES: OC(=O)C(F)(F)OC(F)(F)C(F)(F)OC(F)(F)F

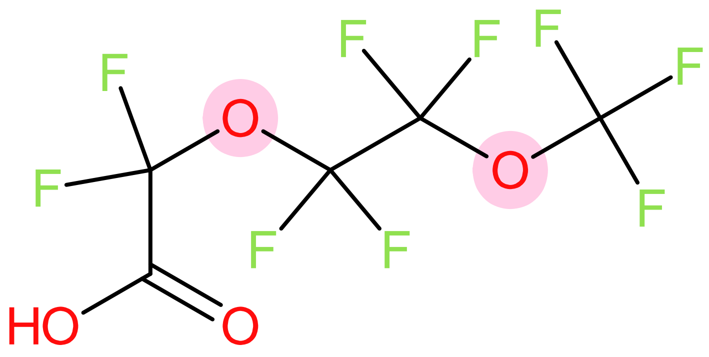

S22: Perfluoro-3,6,9-trioxatridecanoic acid (PFPE-6)
SMILES: OC(=O)C(F)(F)OC(F)(F)C(F)(F)OC(F)(F)C(F)(F)OC(F)(F)C(F)(F)C(F)(F)C(F)(F)F

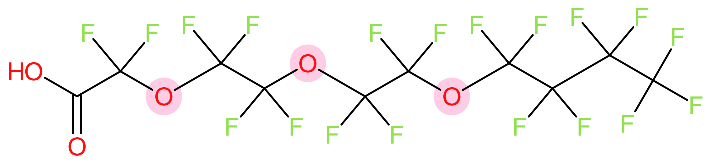

S36: Perfluoro-4-isopropoxybutanoic acid (PFPE-1)
SMILES: OC(=O)C(F)(F)C(F)(F)C(F)(F)OC(F)(C(F)(F)F)C(F)(F)F

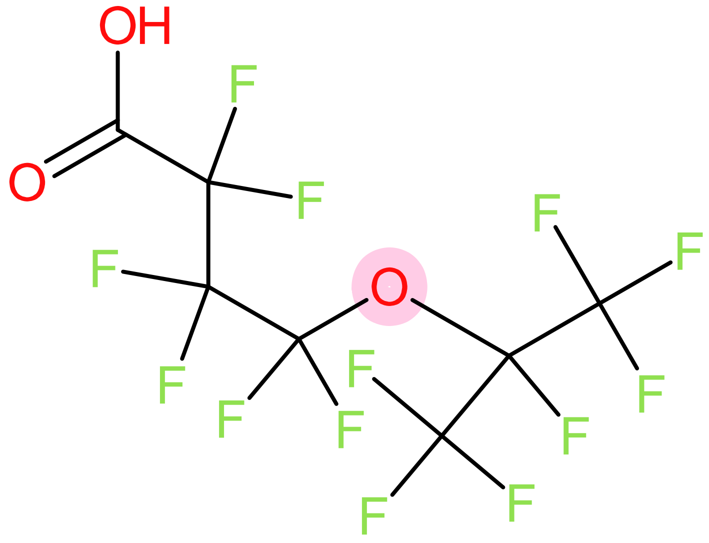

S38: 2,2,2-Trifluoroethyl perfluorobutanesulfonate (ET-PFBS)
SMILES: FC(F)(F)COS(=O)(=O)C(F)(F)C(F)(F)C(F)(F)C(F)(F)F

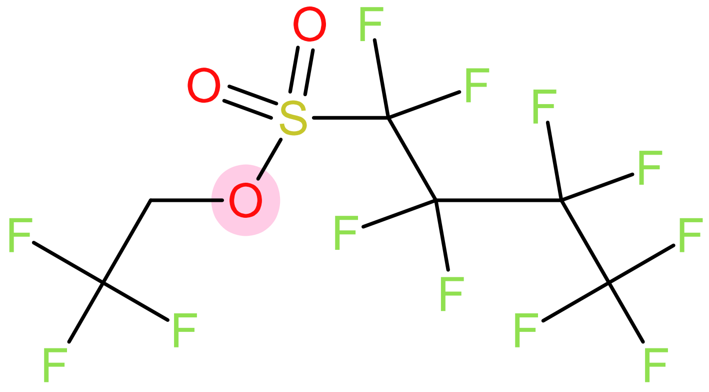

S39: Methyl perfluoro(3-(1-ethenyloxypropan-2-yloxy)propanoate) (MePF2ETOA)
SMILES: COC(=O)C(F)(F)C(F)(F)OC(F)(C(F)(F)F)C(F)(F)OC(F)=C(F)F

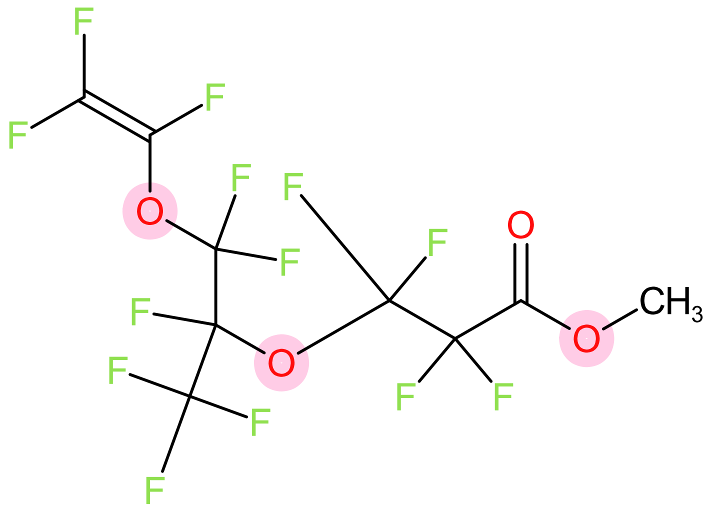

S49: Perfluoro-3-methoxypropanoic acid (PFMPA)
SMILES: OC(=O)C(F)(F)C(F)(F)OC(F)(F)F

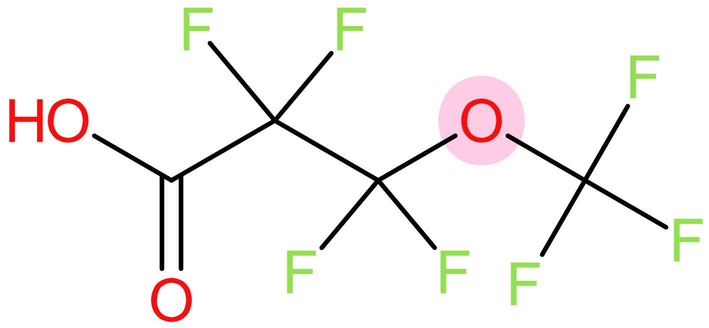

S56: Fluorinated triethylene glycol monomethyl ether (C7F3ETOH)
SMILES: OCC(F)(F)OC(F)(F)C(F)(F)OC(F)(F)C(F)(F)OC(F)(F)F

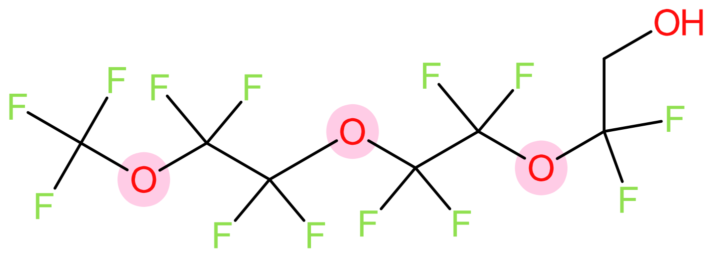

SGR10032

S1: 1-Pentafluoroethylethanol (PFBOH)
SMILES: CC(O)C(F)(F)C(F)(F)F

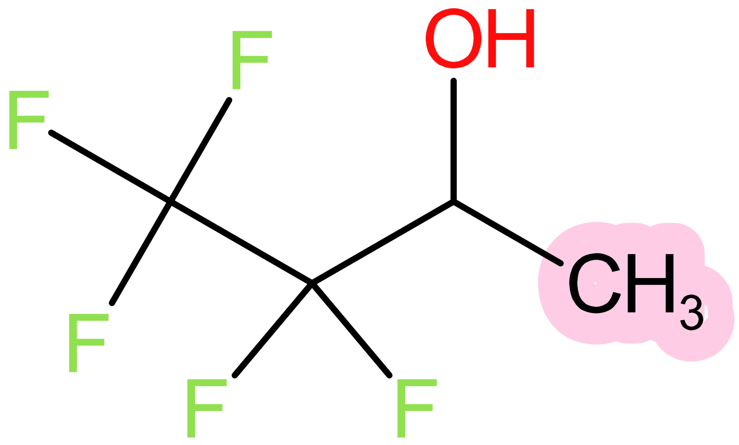

S39: Methyl perfluoro(3-(1-ethenyloxypropan-2-yloxy)propanoate) (MePF2ETOA)
SMILES: COC(=O)C(F)(F)C(F)(F)OC(F)(C(F)(F)F)C(F)(F)OC(F)=C(F)F

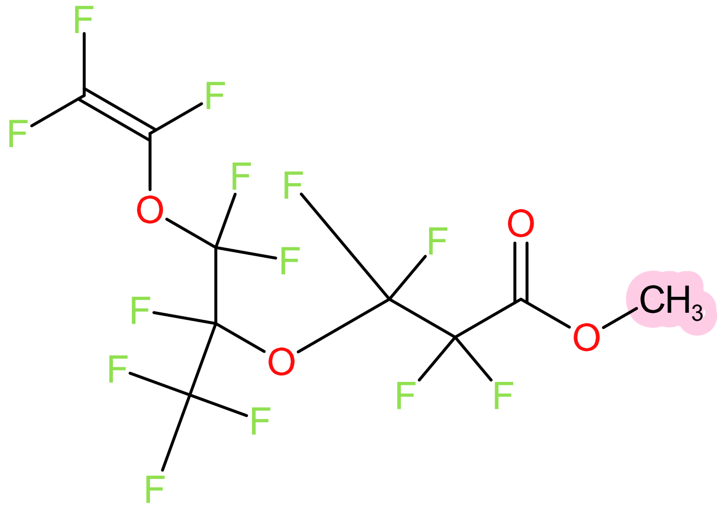

S43: N-Methyl-N-(2-hydroxyethyl)perfluorooctanesulfonamide (MeFOSE)

SMILES: CN(CCO)S(=O)(=O)C(F)(F)C(F)(F)C(F)(F)C(F)(F)C(F)(F)C(F)(F)C(F)(F)C(F)(F)F

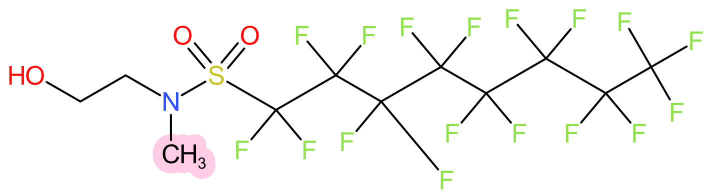

ATOM PAIRS

SGR10786 (8 chem)
SGR10112 (7 chem)
SGR10199 (3 chem)

SGR10786

S11: Perfluoro-3,6-dioxaheptanoic acid (NFDHA)
SMILES: OC(=O)C(F)(F)OC(F)(F)C(F)(F)OC(F)(F)F

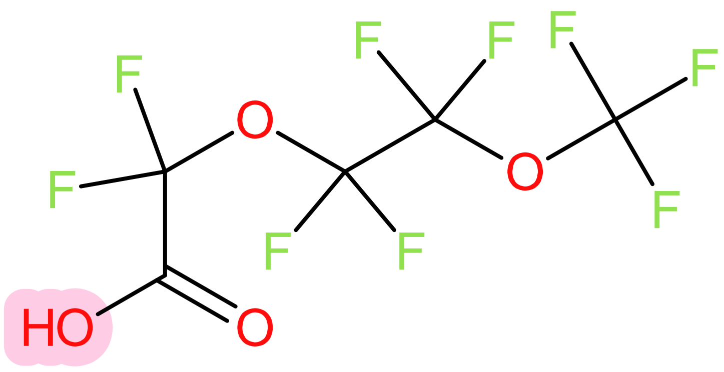

S2: 3-(Perfluoro-2-butyl)propane-1,2-diol (PFHp2OH)
SMILES: OCC(O)CC(F)(F)C(F)(F)C(F)(F)C(F)(F)F

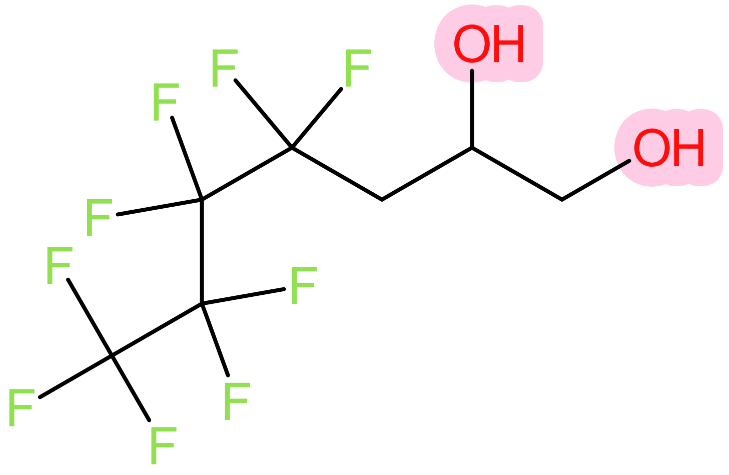

S22: Perfluoro-3,6,9-trioxatridecanoic acid (PFPE-6)
SMILES: OC(=O)C(F)(F)OC(F)(F)C(F)(F)OC(F)(F)C(F)(F)OC(F)(F)C(F)(F)C(F)(F)C(F)(F)F

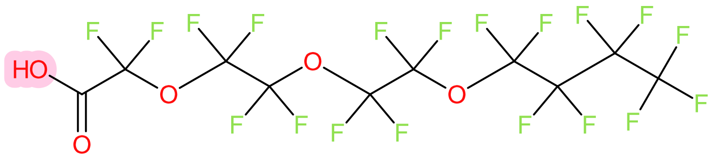

S3: Perfluoro-3,6-dioxaoctane-1,8-dioic acid (PFHx2Et2OA)
SMILES: OC(=O)C(F)(F)OC(F)(F)C(F)(F)OC(F)(F)C(O)=O

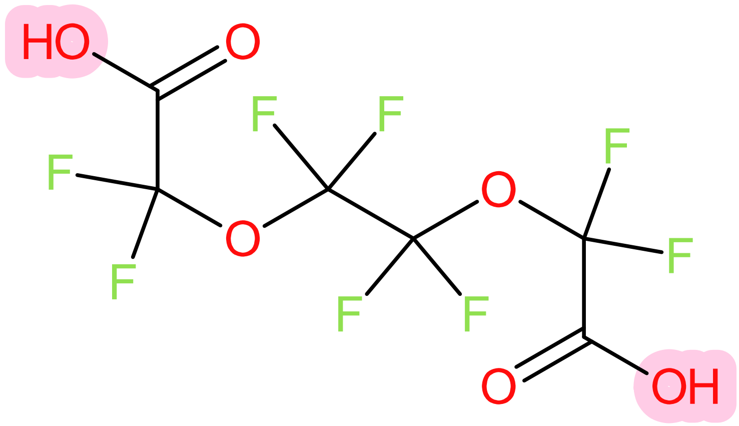

S40: 1-(Perfluorofluorooctyl)propane-2,3-diol (PFUd2OH)
SMILES: OCC(O)CC(F)(F)C(F)(F)C(F)(F)C(F)(F)C(F)(F)C(F)(F)C(F)(F)C(F)(F)F

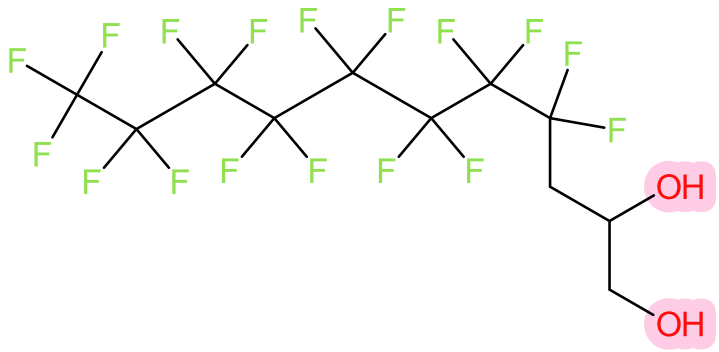

S43: N-Methyl-N-(2-hydroxyethyl)perfluorooctanesulfonamide (MeFOSE)
SMILES: CN(CCO)S(=O)(=O)C(F)(F)C(F)(F)C(F)(F)C(F)(F)C(F)(F)C(F)(F)C(F)(F)C(F)(F)F

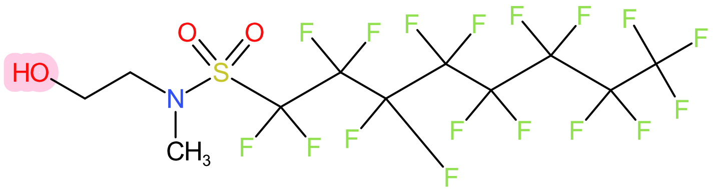

S49: Perfluoro-3-methoxypropanoic acid (PFMPA)
SMILES: OC(=O)C(F)(F)C(F)(F)OC(F)(F)F

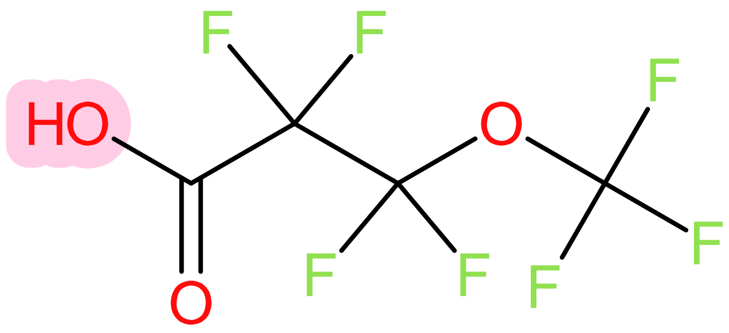

S56: Fluorinated triethylene glycol monomethyl ether (C7F3ETOH)
SMILES: OCC(F)(F)OC(F)(F)C(F)(F)OC(F)(F)C(F)(F)OC(F)(F)F

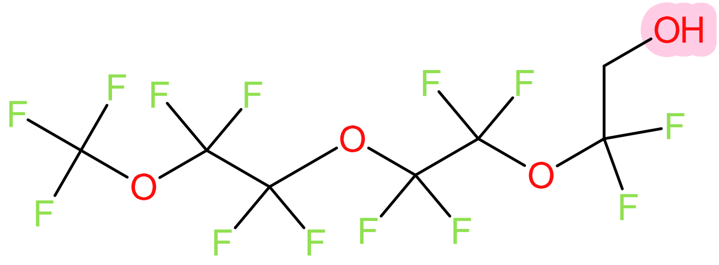

SGR10112

S2: 3-(Perfluoro-2-butyl)propane-1,2-diol (PFHp2OH)
SMILES: OCC(O)CC(F)(F)C(F)(F)C(F)(F)C(F)(F)F

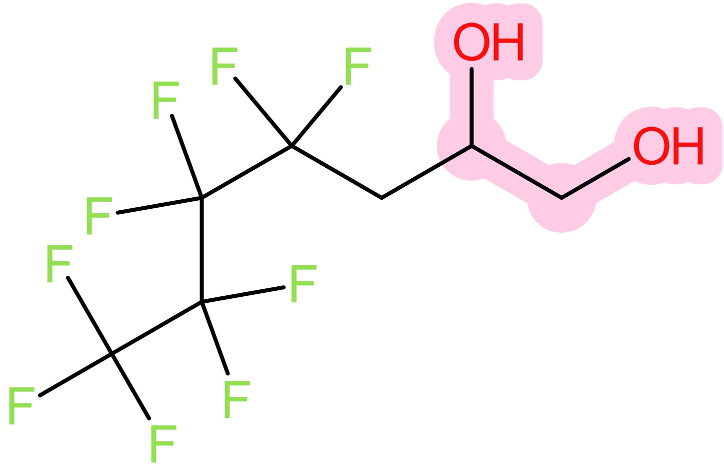

S3: Perfluoro-3,6-dioxaoctane-1,8-dioic acid (PFHx2EtOA)
SMILES: OC(=O)C(F)(F)OC(F)(F)C(F)(F)OC(F)(F)C(O)=O

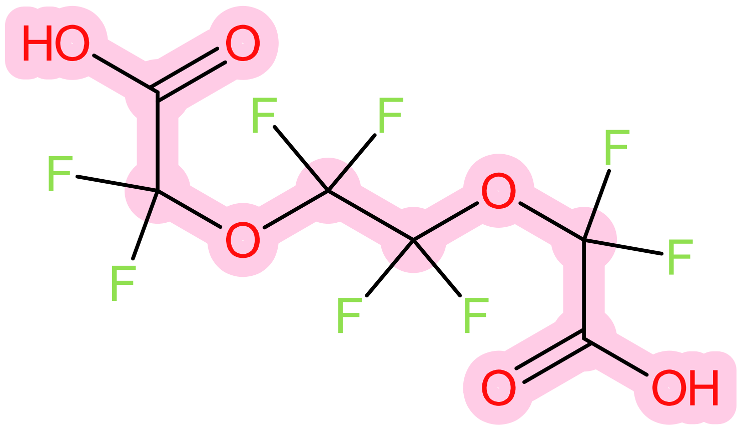

S11: Perfluoro-3,6-dioxaheptanoic acid (NFDHA)
SMILES: OC(=O)C(F)(F)OC(F)(F)C(F)(F)OC(F)(F)F

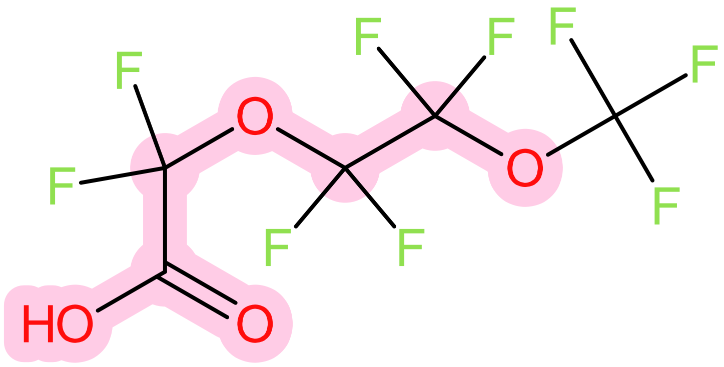

S22: Perfluoro-3,6,9-trioxatridecanoic acid (PFPE-6)
SMILES: OC(=O)C(F)(F)OC(F)(F)C(F)(F)OC(F)(F)C(F)(F)OC(F)(F)C(F)(F)C(F)(F)C(F)(F)F

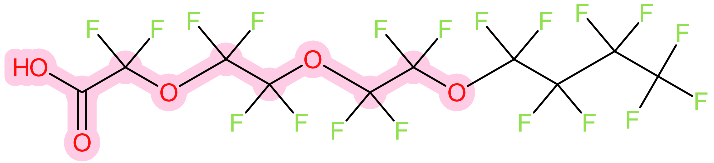

S39: Methyl perfluoro(3-(1-ethenyloxypropan-2-yloxy)propanoate) (MePF2ETOA)
SMILES: COC(=O)C(F)(F)C(F)(F)OC(F)(C(F)(F)F)C(F)(F)OC(F)=C(F)F

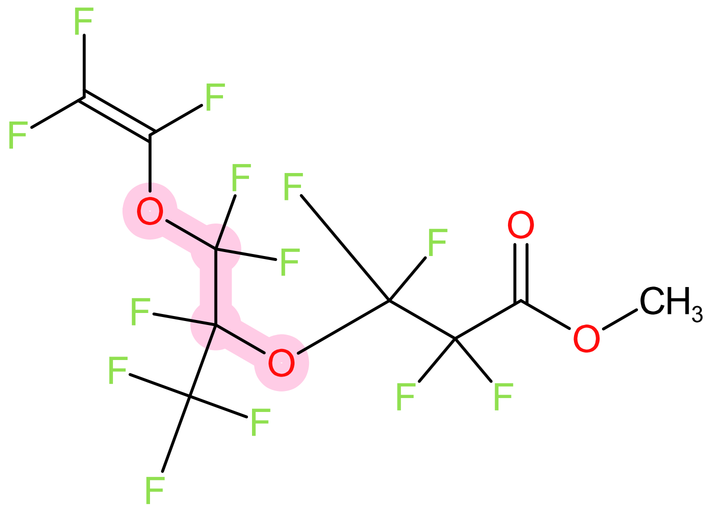

S40: 1-(Perfluorofluorooctyl)propane-2,3-diol (PFUd2OH)
SMILES: OCC(O)CC(F)(F)C(F)(F)C(F)(F)C(F)(F)C(F)(F)C(F)(F)C(F)(F)C(F)(F)F

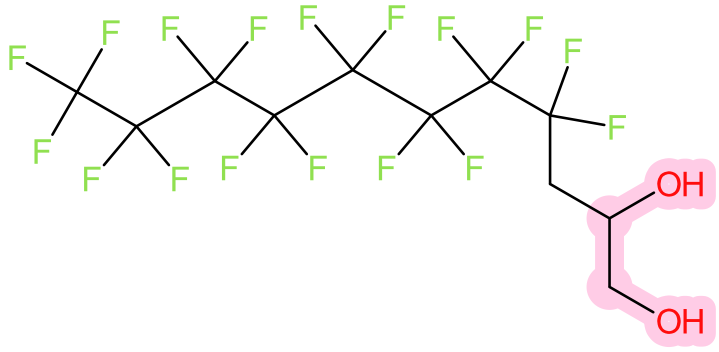

S56: Fluorinated triethylene glycol monomethyl ether (C7F3ETOH)
SMILES: OCC(F)(F)OC(F)(F)C(F)(F)OC(F)(F)C(F)(F)OC(F)(F)F

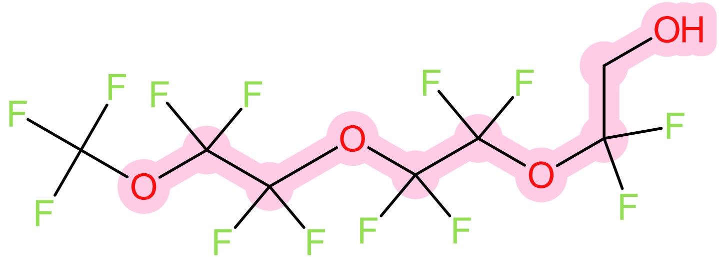

SGR10199

S9: Perfluoro(4-methoxybutanoic) acid (PFMBA)

SMILES: OC(=O)C(F)(F)C(F)(F)C(F)(F)OC(F)(F)F

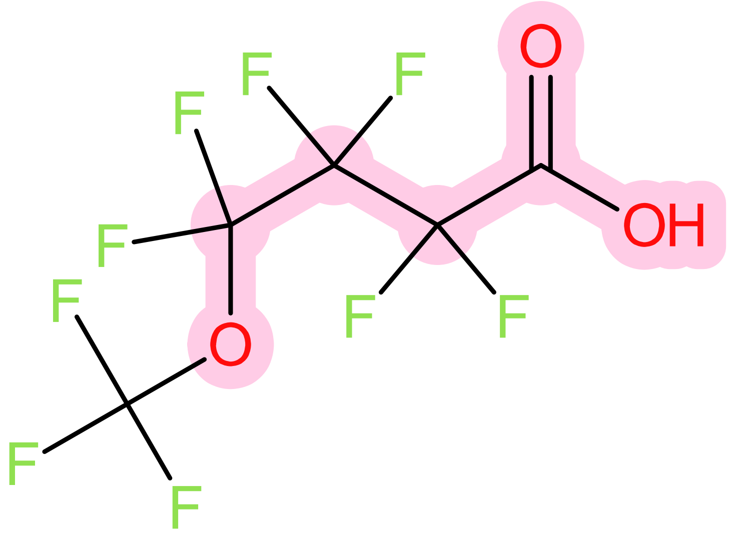

S36: Perfluoro-4-isopropoxybutanoic acid (PFPE-1)

SMILES: OC(=O)C(F)(F)C(F)(F)C(F)(F)OC(F)(C(F)(F)F)C(F)(F)F

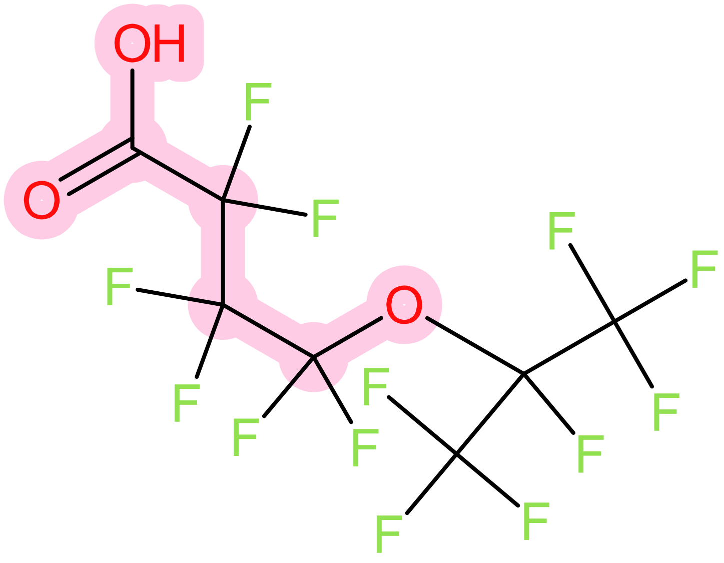

S43: N-Methyl-N-(2-hydroxyethyl)perfluorooctanesulfonamide (MeFOSE)

SMILES: CN(CCO)S(=O)(=O)C(F)(F)C(F)(F)C(F)(F)C(F)(F)C(F)(F)C(F)(F)C(F)(F)C(F)(F)F

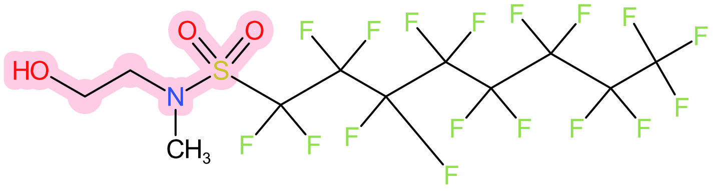

BIOAVAILIBILITY

SGR10633 (17 chem)
SGR10708 (17 chem)
SGR10275 (12 chem)
SGR10169 (8 chem)
SGR10795 (4 chem)
SGR10290 (3 chem)
SGR10418 (3 chem)
SGR10493 (3 chem)
SGR10684 (3 chem)
SGR10736 (3 chem)
SGR10354 (2 chem)
SGR10668 (2 chem)
SGR10703 (2 chem)

SGR10633

S2: 3-(Perfluoro-2-butyl)propane-1,2-diol (PFHp2OH)
SMILES: OCC(O)CC(F)(F)C(F)(F)C(F)(F)C(F)(F)F

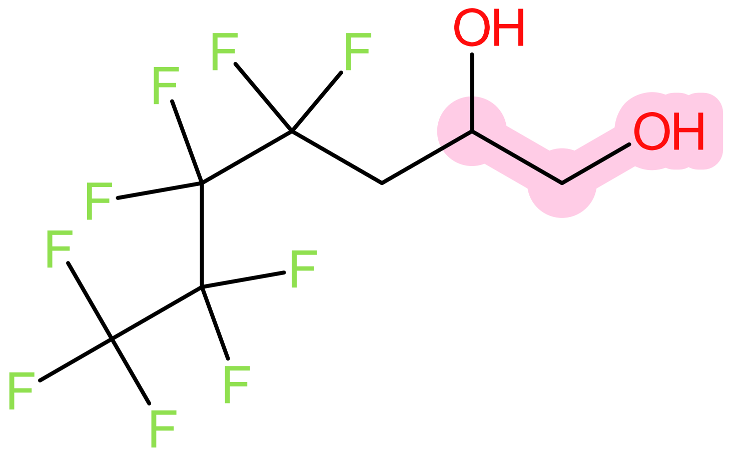

S7: Dodecafluoroheptanol (7H 6:1 FTOH)
SMILES: OCC(F)(F)C(F)(F)C(F)(F)C(F)(F)C(F)(F)C(F)F

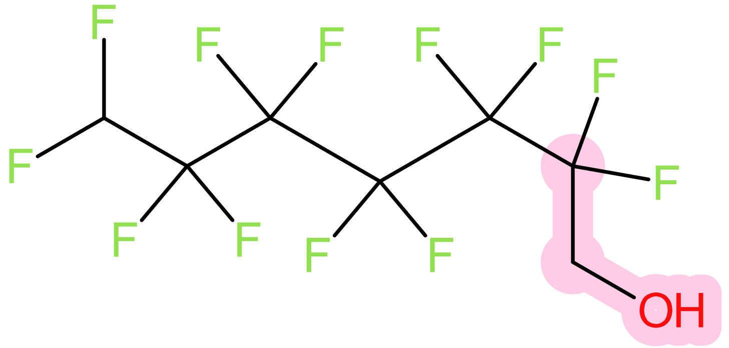

S12: Heptafluorobutanol (HpFBOH)
SMILES: OCC(F)(F)C(F)(F)C(F)(F)F

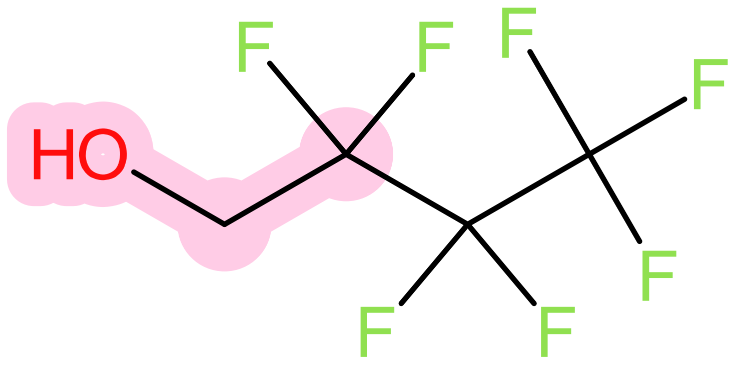

S13: 4:2 Fluorotelomer sulfonic acid (4:2 FTS)
SMILES: OS(=O)(=O)CCC(F)(F)C(F)(F)C(F)(F)C(F)(F)F

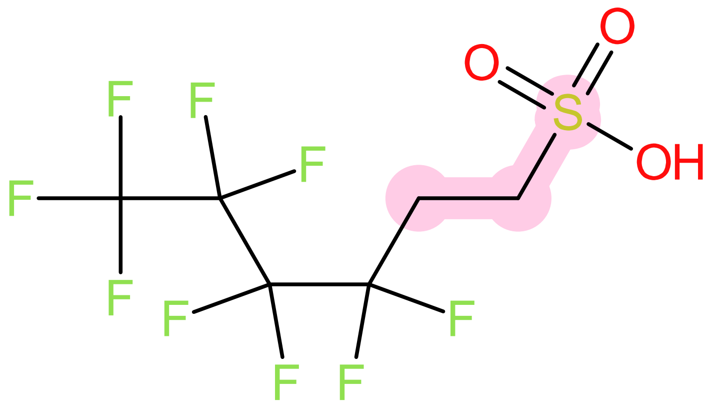

S14: Hexafluoroamylene glycol (CFHx2OH)
SMILES: OCC(F)(F)C(F)(F)C(F)(F)CO

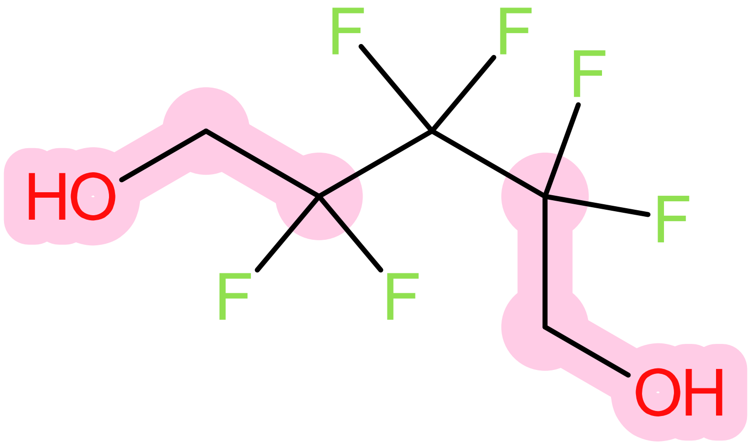

S21: 4:2 Fluorotelomer alcohol (4:2 FTOH)
SMILES: OCCC(F)(F)C(F)(F)C(F)(F)C(F)(F)F

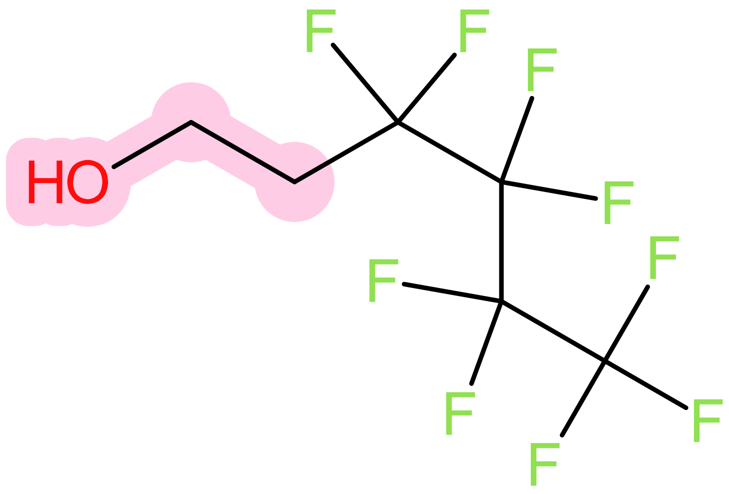

S23: 1H,1H,5H-Perfluoropentanol (PFPOH)
SMILES: OCC(F)(F)C(F)(F)C(F)(F)C(F)F

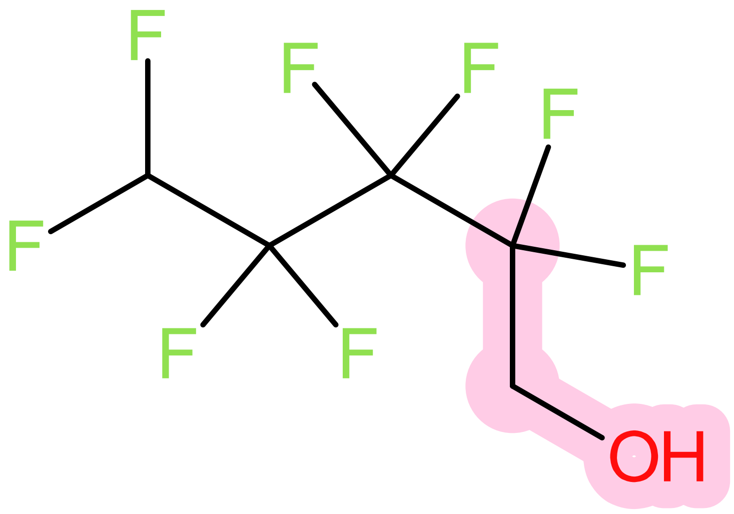

S24: 8:2 Fluorotelomer alcohol (8:2 FTOH)
SMILES: OCCC(F)(F)C(F)(F)C(F)(F)C(F)(F)C(F)(F)C(F)(F)C(F)(F)C(F)(F)F

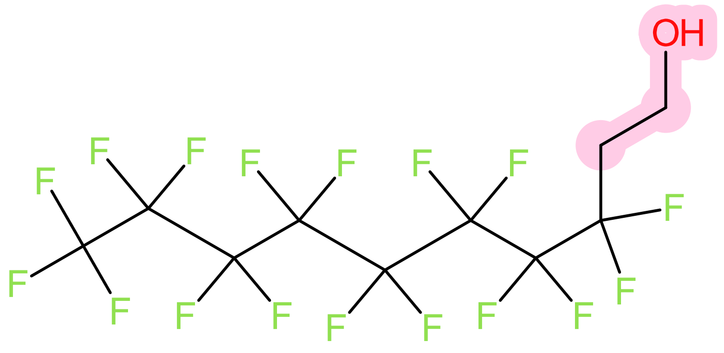

S26: 6:2 Fluorotelomer alcohol (6:2 FTOH)
SMILES: OCCC(F)(F)C(F)(F)C(F)(F)C(F)(F)C(F)(F)C(F)(F)F

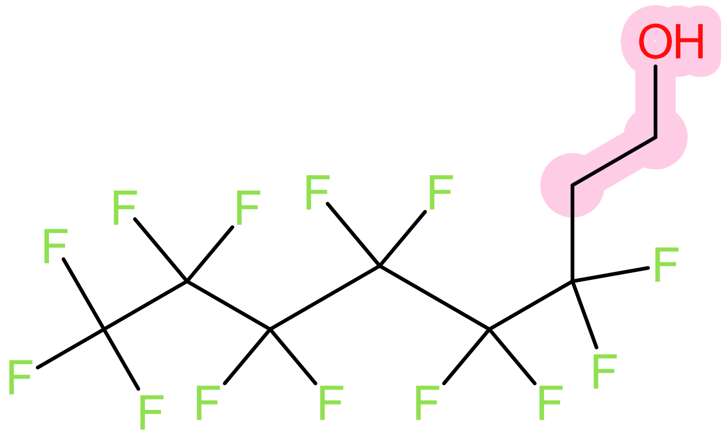

S32: 6:2 Fluorotelomer sulfonic acid (6:2 FTS)
SMILES: OS(=O)(=O)CCC(F)(F)C(F)(F)C(F)(F)C(F)(F)C(F)(F)C(F)(F)F

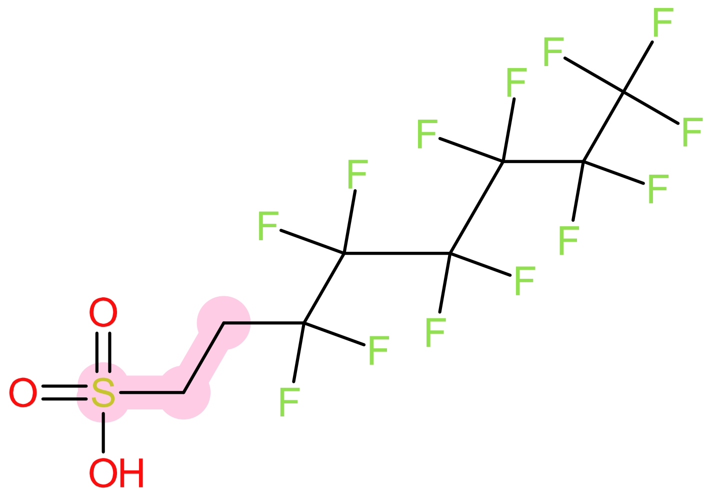

S33: 8:2 Fluorotelomer sulfonic acid (8:2 FTS)
SMILES: OS(=O)(=O)CCC(F)(F)C(F)(F)C(F)(F)C(F)(F)C(F)(F)C(F)(F)C(F)(F)C(F)(F)F

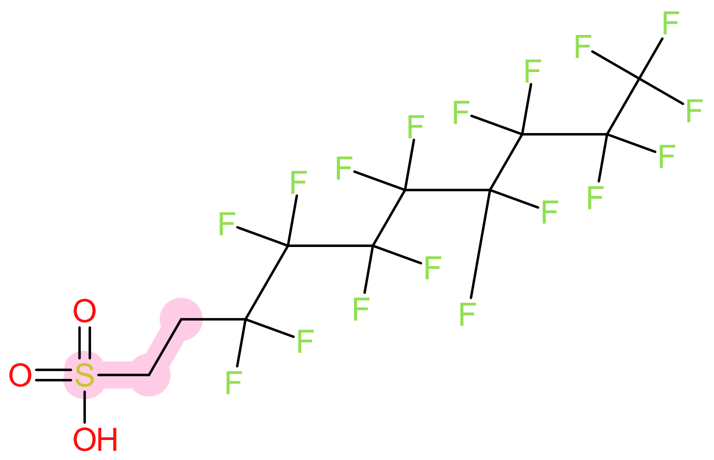

S38: 2,2,2-Trifluoroethyl perfluorobutanesulfonate (ET-PFBS)
SMILES: FC(F)(F)COS(=O)(=O)C(F)(F)C(F)(F)C(F)(F)C(F)(F)F

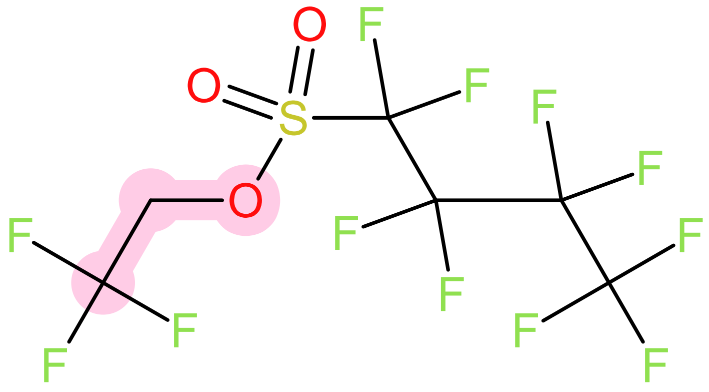

S40: 1-(Perfluorofluorooctyl)propane-2,3-diol (PFUd2OH)
SMILES: OCC(O)CC(F)(F)C(F)(F)C(F)(F)C(F)(F)C(F)(F)C(F)(F)C(F)(F)C(F)(F)F

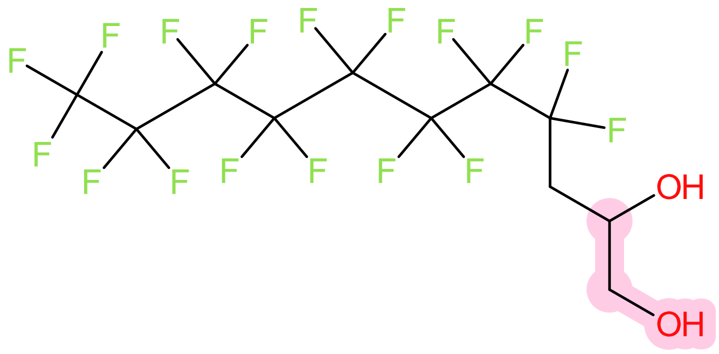

S43: N-Methyl-N-(2-hydroxyethyl)perfluorooctanesulfonamide (MeFOSE)
SMILES: CN(CCO)S(=O)(=O)C(F)(F)C(F)(F)C(F)(F)C(F)(F)C(F)(F)C(F)(F)C(F)(F)C(F)(F)F

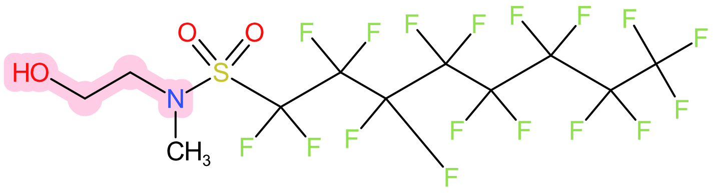

S47: 6:1 Fluorotelomer alcohol (6:1 FTOH)
SMILES: OCC(F)(F)C(F)(F)C(F)(F)C(F)(F)C(F)(F)C(F)(F)F

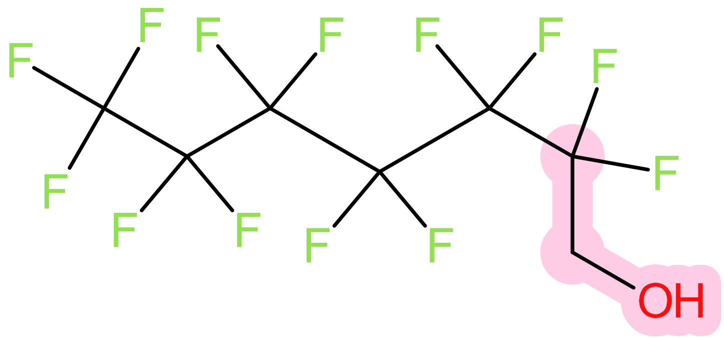

S52: ((Perfluorooctyl)ethyl)phosphonic acid (8:2 FTPA)
SMILES: OP(O)(=O)CCC(F)(F)C(F)(F)C(F)(F)C(F)(F)C(F)(F)C(F)(F)C(F)(F)C(F)(F)F

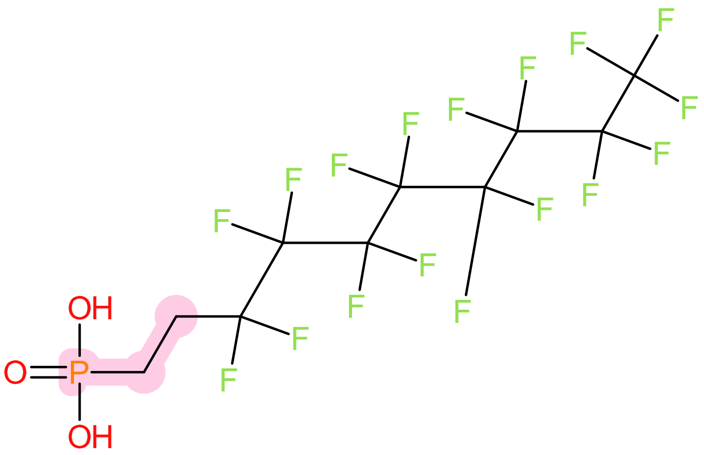

S56: Fluorinated triethylene glycol monomethyl ether (C7F3ETOH)
ID_56 OCC(F)(F)OC(F)(F)C(F)(F)OC(F)(F)C(F)(F)OC(F)(F)F

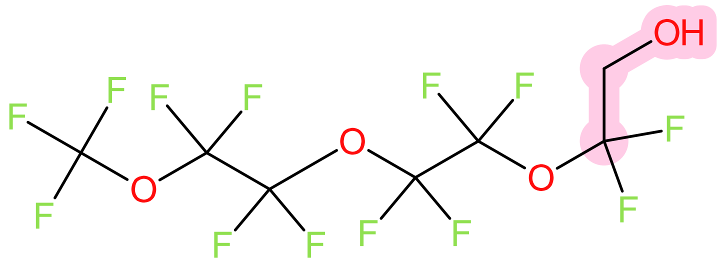

SGR10708

S2: 3-(Perfluoro-2-butyl)propane-1,2-diol (PFHp2OH)
SMILES: OCC(O)CC(F)(F)C(F)(F)C(F)(F)C(F)(F)F

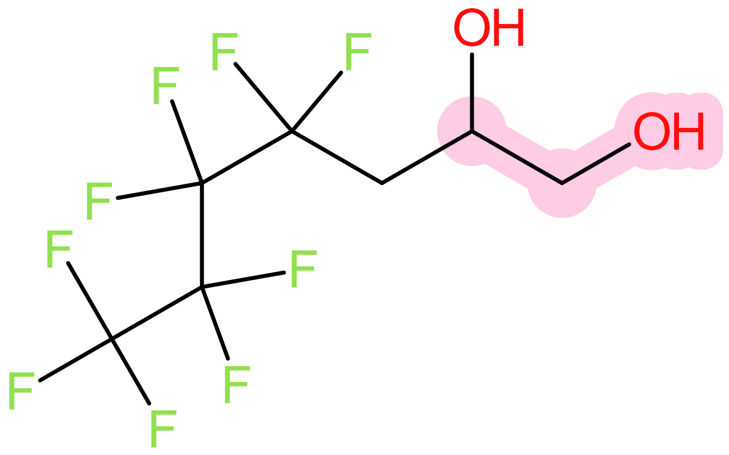

S7: Dodecafluoroheptanol (7H 6:1 FTOH)
SMILES: OCC(F)(F)C(F)(F)C(F)(F)C(F)(F)C(F)(F)C(F)F

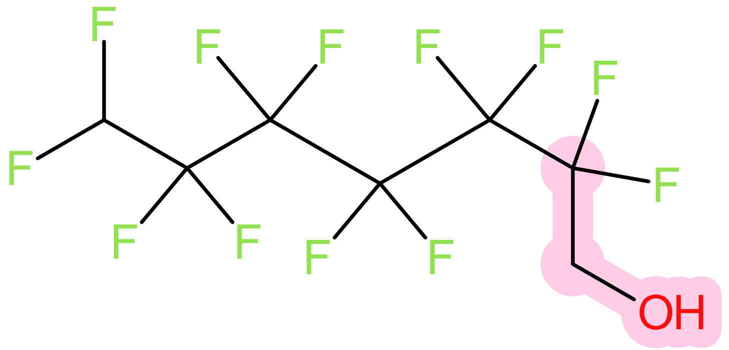

S12: Heptafluorobutanol (HpFBOH)
SMILES: OCC(F)(F)C(F)(F)C(F)(F)F

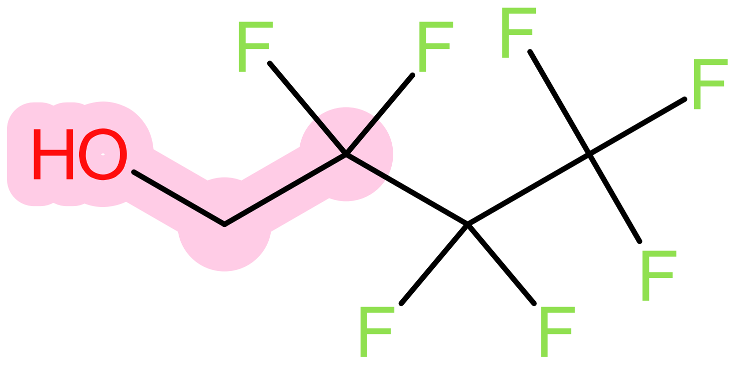

S13: 4:2 Fluorotelomer sulfonic acid (4:2 FTS)
SMILES: OS(=O)(=O)CCC(F)(F)C(F)(F)C(F)(F)C(F)(F)F

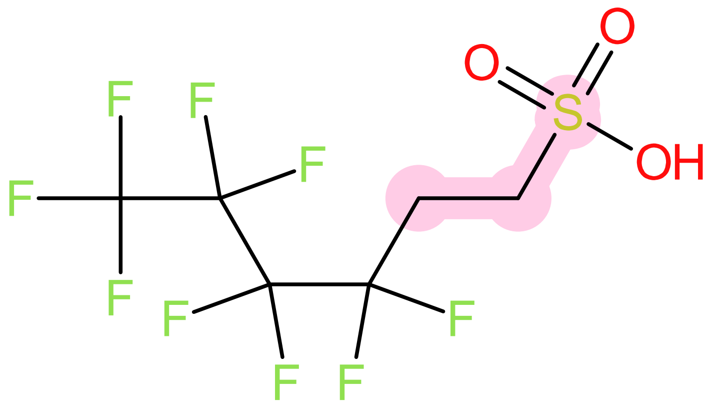

S14: Hexafluoroamylene glycol (CFHx2OH)
SMILES: OCC(F)(F)C(F)(F)C(F)(F)CO

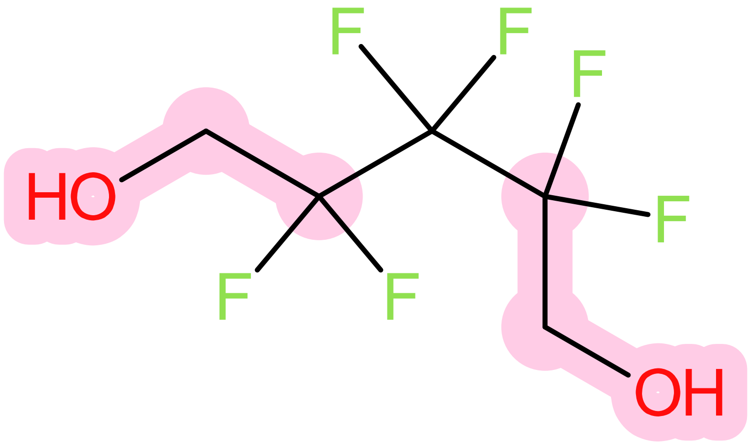

S21: 4:2 Fluorotelomer alcohol (4:2 FTOH)
SMILES: OCCC(F)(F)C(F)(F)C(F)(F)C(F)(F)F

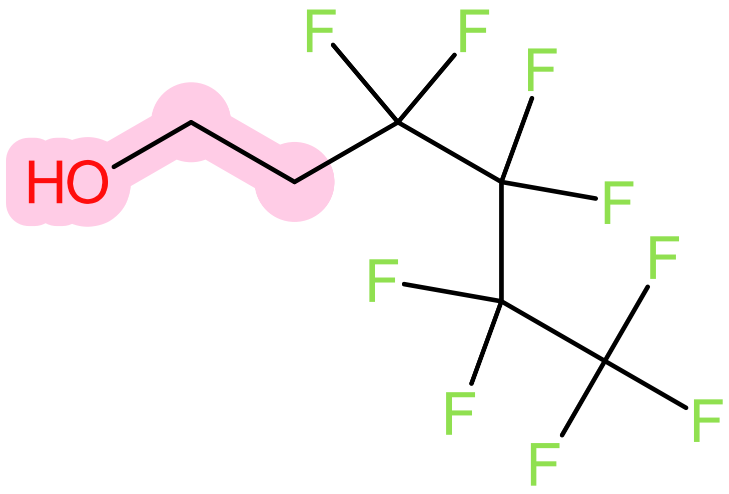

S23: 1H,1H,5H-Perfluoropentanol (PFPOH)
SMILES: OCC(F)(F)C(F)(F)C(F)(F)C(F)F

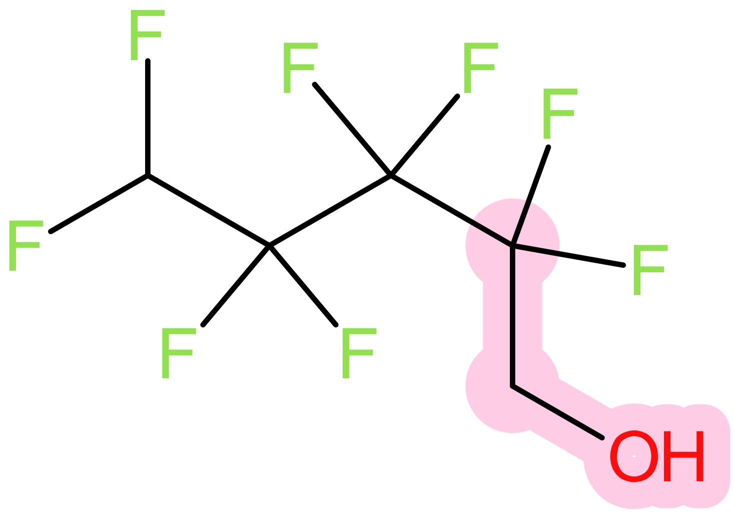

S24: 8:2 Fluorotelomer alcohol (8:2 FTOH)
SMILES: OCCC(F)(F)C(F)(F)C(F)(F)C(F)(F)C(F)(F)C(F)(F)C(F)(F)C(F)(F)F

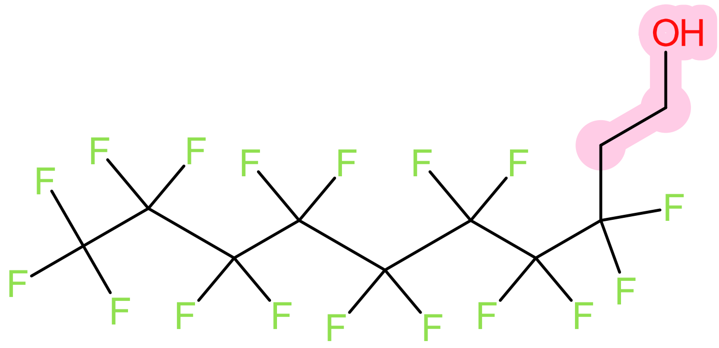

S26: 6:2 Fluorotelomer alcohol (6:2 FTOH)
ID_26 OCCC(F)(F)C(F)(F)C(F)(F)C(F)(F)C(F)(F)C(F)(F)F

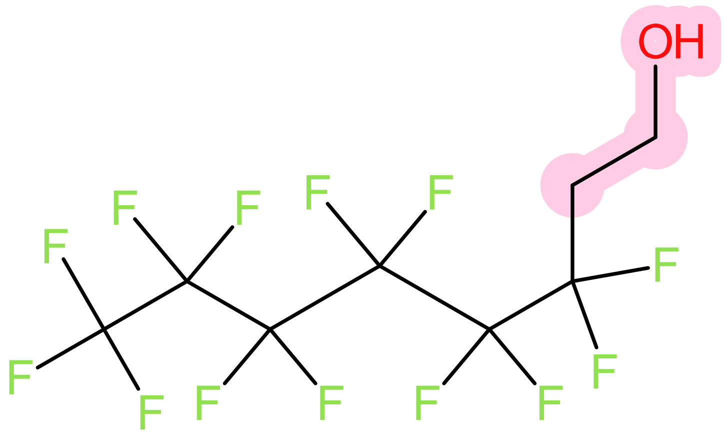

S32: 6:2 Fluorotelomer sulfonic acid (6:2 FTS)
SMILES: OS(=O)(=O)CCC(F)(F)C(F)(F)C(F)(F)C(F)(F)C(F)(F)C(F)(F)F

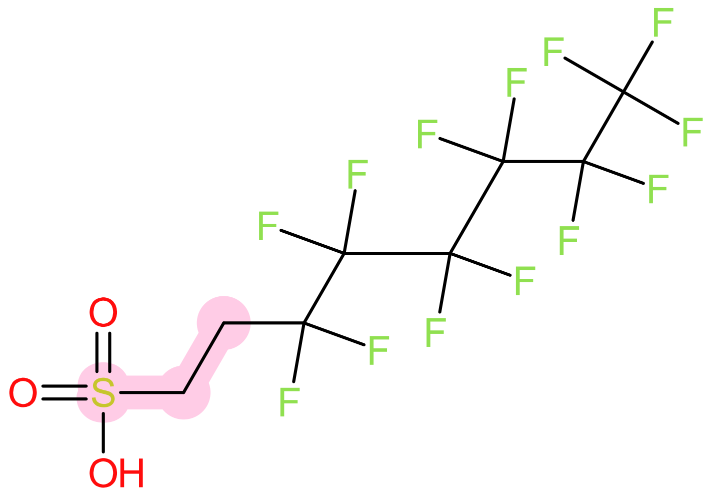

S33: 8:2 Fluorotelomer sulfonic acid (8:2 FTS)
SMILES: OS(=O)(=O)CCC(F)(F)C(F)(F)C(F)(F)C(F)(F)C(F)(F)C(F)(F)C(F)(F)C(F)(F)F

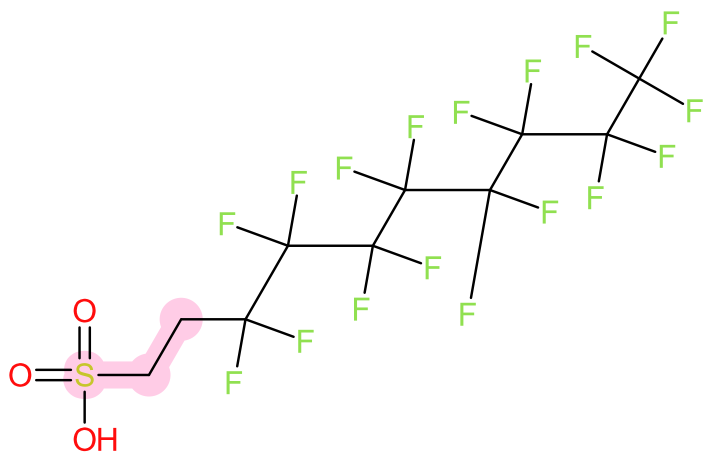

S38: 2,2,2-Trifluoroethyl perfluorobutanesulfonate (ET-PFBS)
SMILES: FC(F)(F)COS(=O)(=O)C(F)(F)C(F)(F)C(F)(F)C(F)(F)F

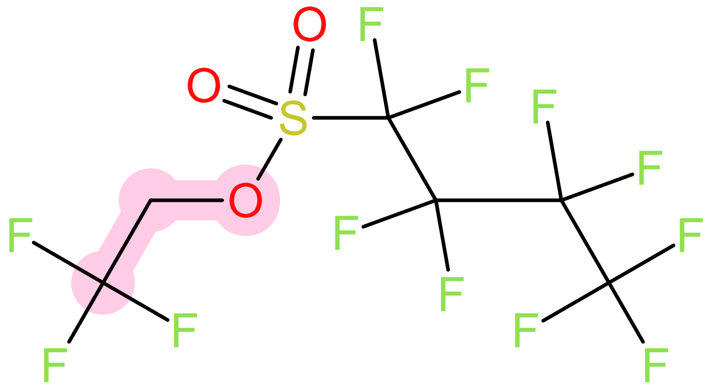

S40: 1-(Perfluorofluorooctyl)propane-2,3-diol (PFUd2OH)
SMILES: OCC(O)CC(F)(F)C(F)(F)C(F)(F)C(F)(F)C(F)(F)C(F)(F)C(F)(F)C(F)(F)F

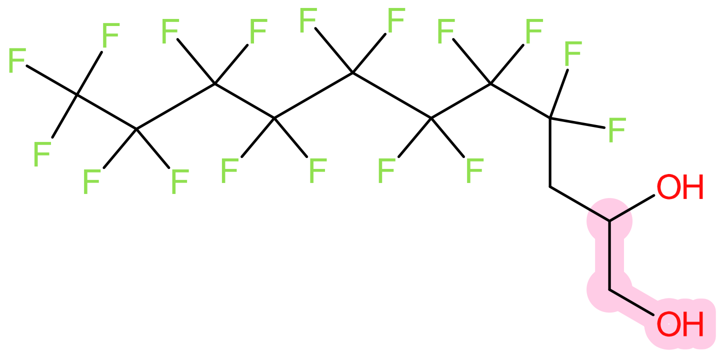

S43: N-Methyl-N-(2-hydroxyethyl)perfluorooctanesulfonamide (MeFOSE)
SMILES: CN(CCO)S(=O)(=O)C(F)(F)C(F)(F)C(F)(F)C(F)(F)C(F)(F)C(F)(F)C(F)(F)C(F)(F)F

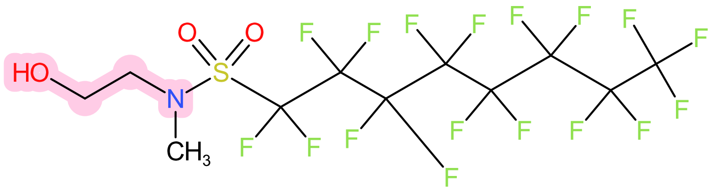

S47: 6:1 Fluorotelomer alcohol (6:1 FTOH)
SMILES: OCC(F)(F)C(F)(F)C(F)(F)C(F)(F)C(F)(F)C(F)(F)F

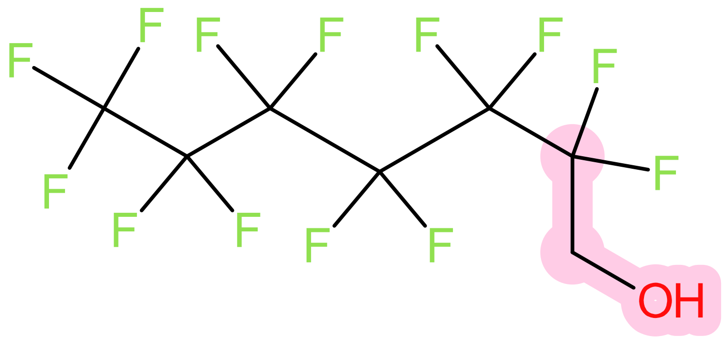

S52: ((Perfluorooctyl)ethyl)phosphonic acid (8:2 FTPA)
SMILES: OP(O)(=O)CCC(F)(F)C(F)(F)C(F)(F)C(F)(F)C(F)(F)C(F)(F)C(F)(F)C(F)(F)F

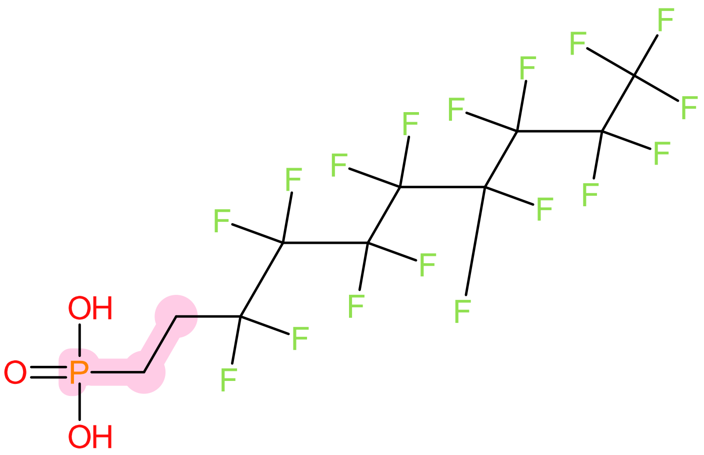

S56: Fluorinated triethylene glycol monomethyl ether (C7F3ETOH)
SMILES: OCC(F)(F)OC(F)(F)C(F)(F)OC(F)(F)C(F)(F)OC(F)(F)F

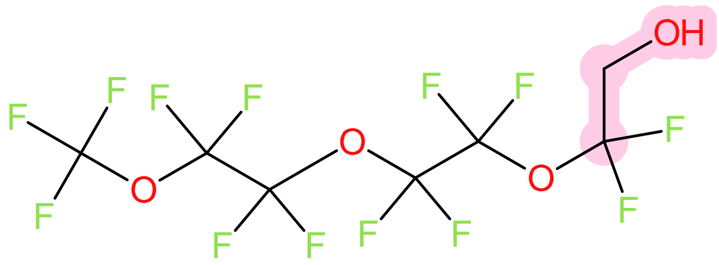

SGR10275

S2: 3-(Perfluoro-2-butyl)propane-1,2-diol (PFHp2OH)
SMILES: OCC(O)CC(F)(F)C(F)(F)C(F)(F)C(F)(F)F

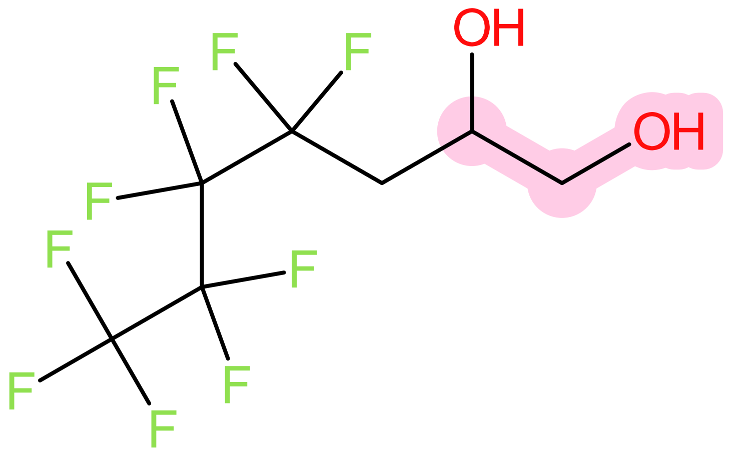

S7:Dodecafluoroheptanol (7H 6:1 FTOH)
SMILES: OCC(F)(F)C(F)(F)C(F)(F)C(F)(F)C(F)(F)C(F)F

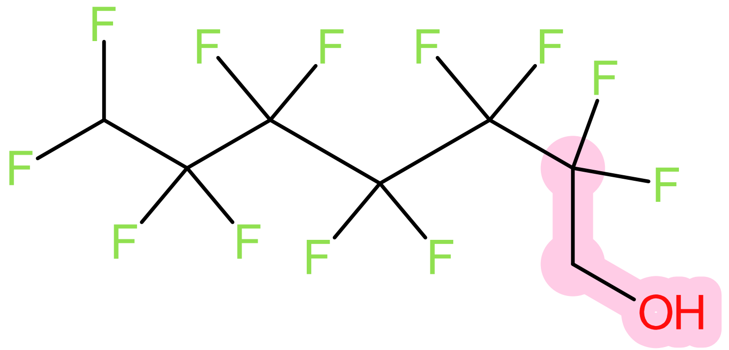

S12: Heptafluorobutanol (HpFBOH)
SMILES: OCC(F)(F)C(F)(F)C(F)(F)F

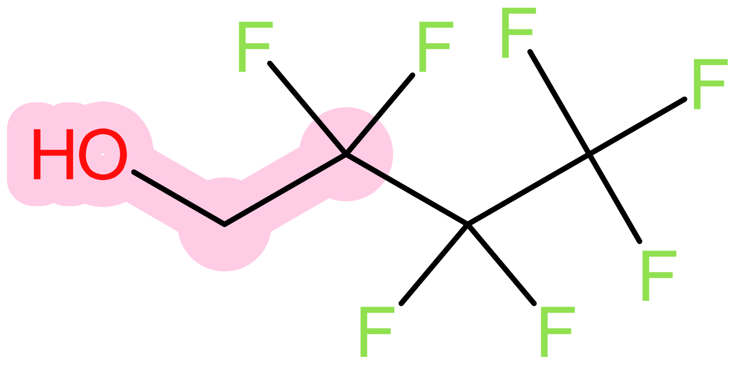

S14: Hexafluoroamylene glycol (CFHx2OH)
SMILES: OCC(F)(F)C(F)(F)C(F)(F)CO

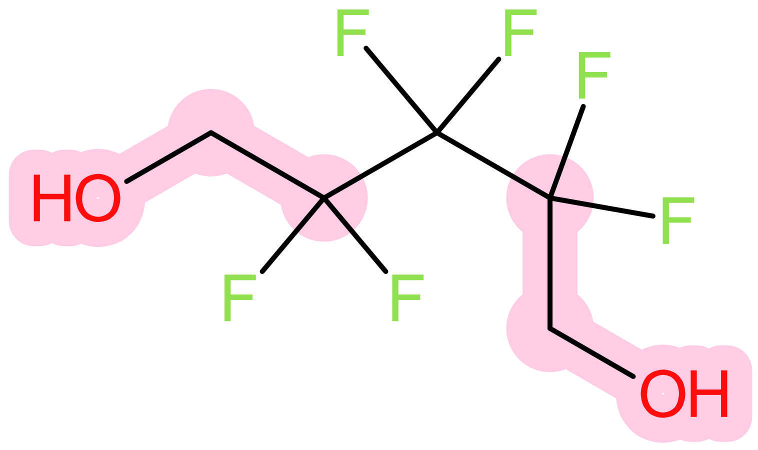

S21: 4:2 Fluorotelomer alcohol (4:2 FTOH)
SMILES: OCCC(F)(F)C(F)(F)C(F)(F)C(F)(F)F

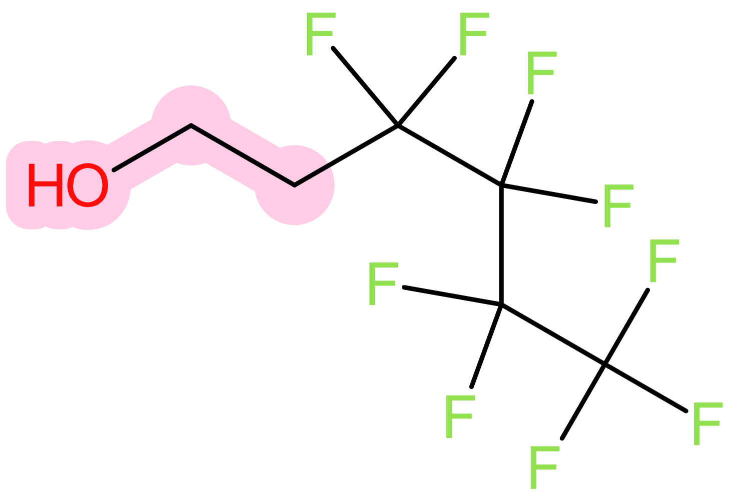

S23: 1H,1H,5H-Perfluoropentanol (PFPOH)
SMILES: OCC(F)(F)C(F)(F)C(F)(F)C(F)F

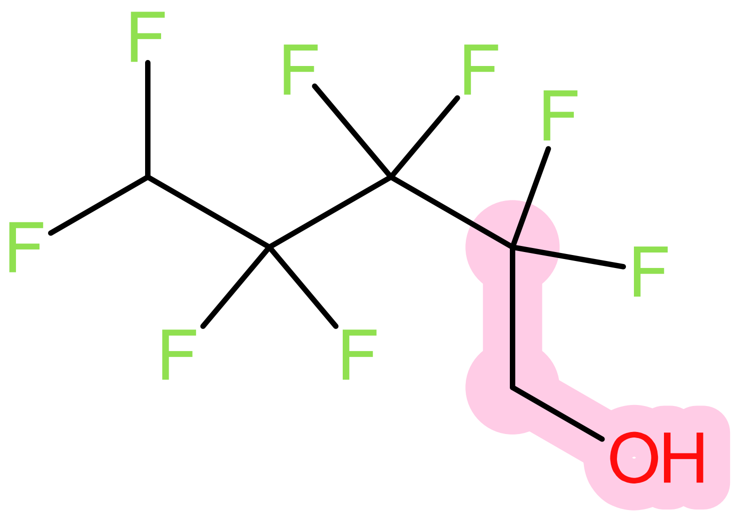

S24: 8:2 Fluorotelomer alcohol (8:2 FTOH)
SMILES: OCCC(F)(F)C(F)(F)C(F)(F)C(F)(F)C(F)(F)C(F)(F)C(F)(F)C(F)(F)F

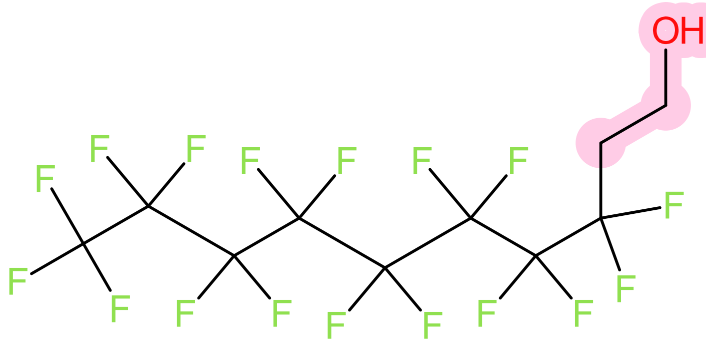

S26: 6:2 Fluorotelomer alcohol (6:2 FTOH)
SMILES: OCCC(F)(F)C(F)(F)C(F)(F)C(F)(F)C(F)(F)C(F)(F)F

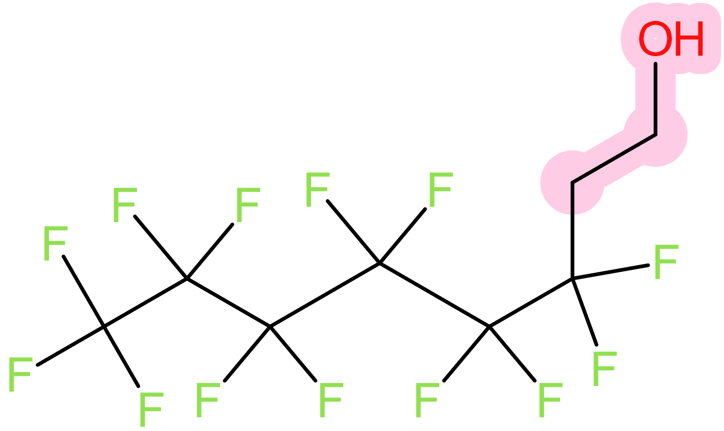

S40: 1-(Perfluorofluorooctyl)propane-2,3-diol (PFUd2OH)
SMILES: OCC(O)CC(F)(F)C(F)(F)C(F)(F)C(F)(F)C(F)(F)C(F)(F)C(F)(F)C(F)(F)F

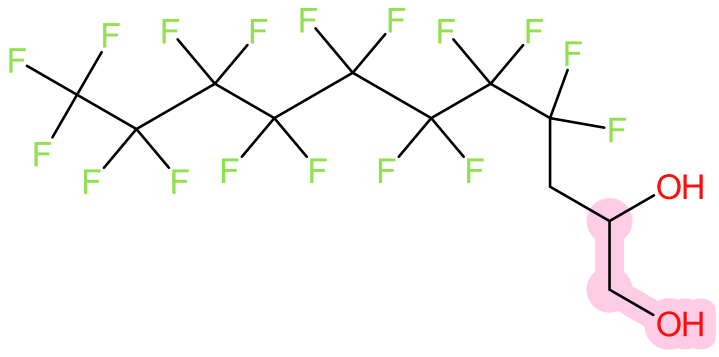

S43: N-Methyl-N-(2-hydroxyethyl)perfluorooctanesulfonamide (MeFOSE)
SMILES: CN(CCO)S(=O)(=O)C(F)(F)C(F)(F)C(F)(F)C(F)(F)C(F)(F)C(F)(F)C(F)(F)C(F)(F)F

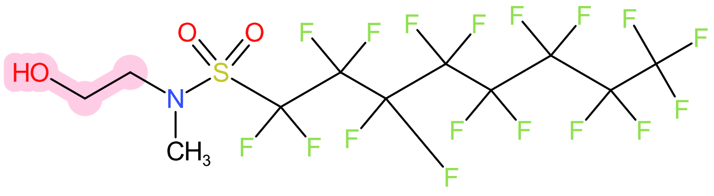

S47: 6:1 Fluorotelomer alcohol (6:1 FTOH)
SMILES: OCC(F)(F)C(F)(F)C(F)(F)C(F)(F)C(F)(F)C(F)(F)F

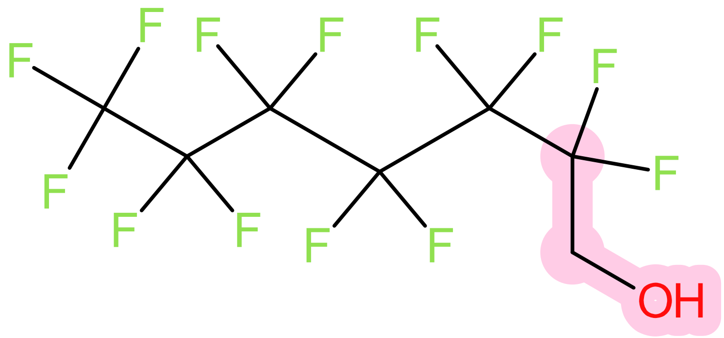

S56: Fluorinated triethylene glycol monomethyl ether (C7F3ETOH)
SMILES: OCC(F)(F)OC(F)(F)C(F)(F)OC(F)(F)C(F)(F)OC(F)(F)F

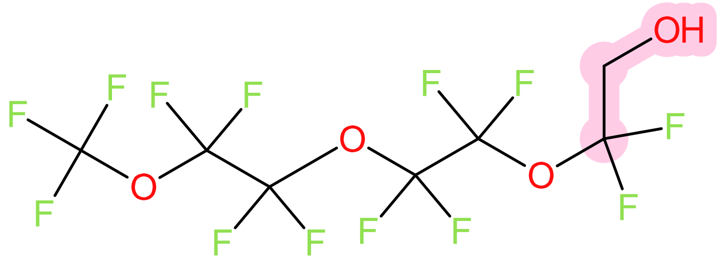

SGR10169

S3: Perfluoro-3,6-dioxaoctane-1,8-dioic acid (PFHx2Et2OA)
SMILES: OC(=O)C(F)(F)OC(F)(F)C(F)(F)OC(F)(F)C(O)=O

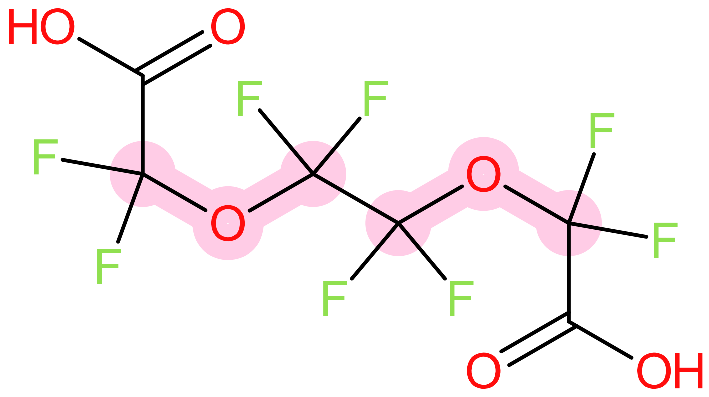

S9: Perfluoro(4-methoxybutanoic) acid (PFMBA)
SMILES: OC(=O)C(F)(F)C(F)(F)C(F)(F)OC(F)(F)F

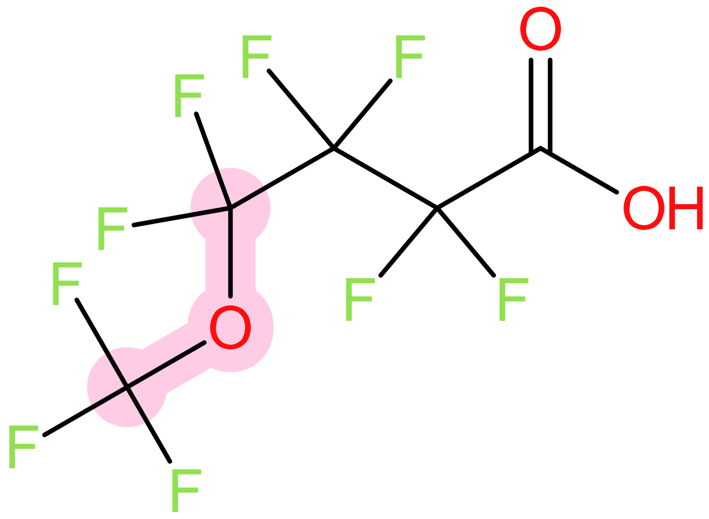

S11: Perfluoro-3,6-dioxaheptanoic acid (NFDHA)
SMILES: OC(=O)C(F)(F)OC(F)(F)C(F)(F)OC(F)(F)F

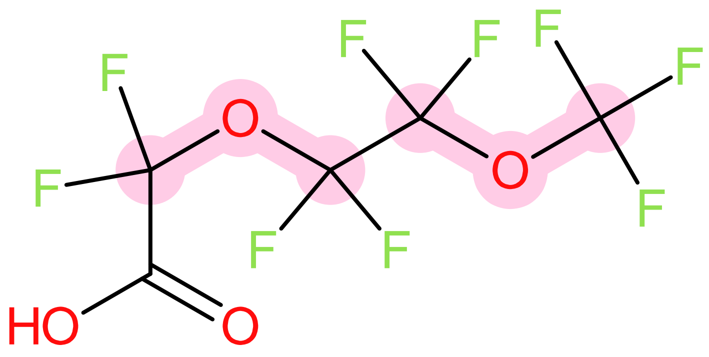

S22: Perfluoro-3,6,9-trioxatridecanoic acid (PFPE-6)
SMILES: OC(=O)C(F)(F)OC(F)(F)C(F)(F)OC(F)(F)C(F)(F)OC(F)(F)C(F)(F)C(F)(F)C(F)(F)F

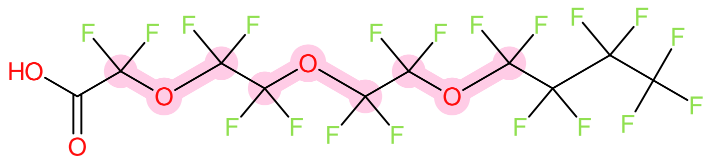

S36: Perfluoro-4-isopropoxybutanoic acid (PFPE-1)
SMILES: OC(=O)C(F)(F)C(F)(F)C(F)(F)OC(F)(C(F)(F)F)C(F)(F)F

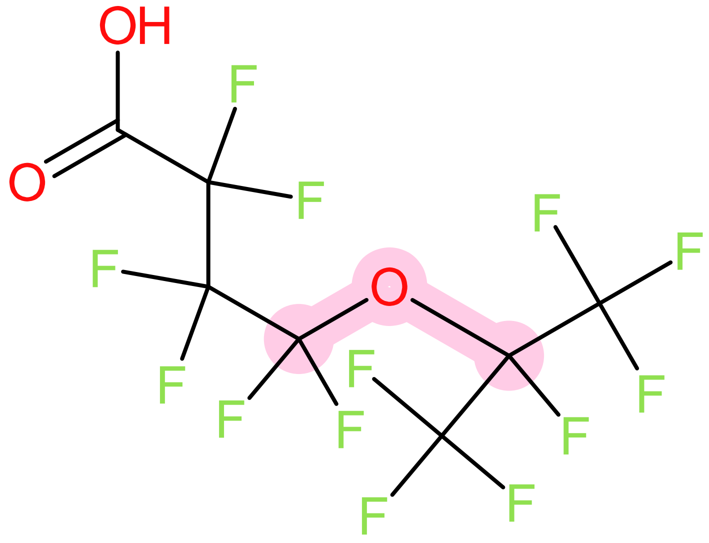

S39: Methyl perfluoro(3-(1-ethenyloxypropan-2-yloxy)propanoate) (MePF2ETOA)
SMILES: COC(=O)C(F)(F)C(F)(F)OC(F)(C(F)(F)F)C(F)(F)OC(F)=C(F)F

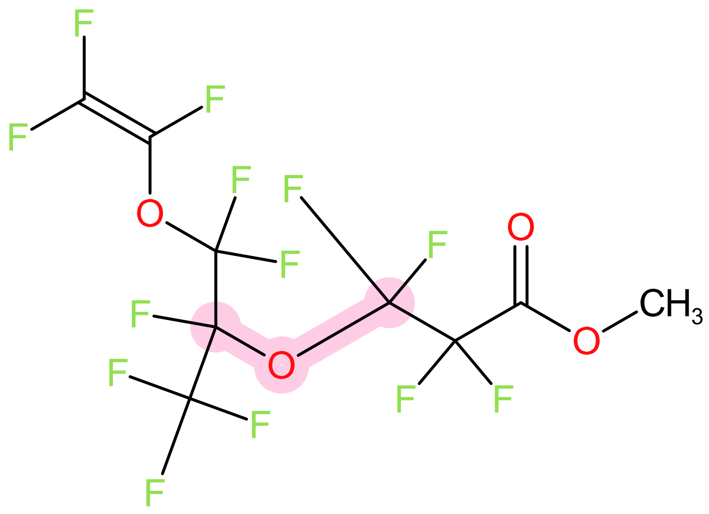

S49: Perfluoro-3-methoxypropanoic acid (PFMPA)
SMILES: OC(=O)C(F)(F)C(F)(F)OC(F)(F)F

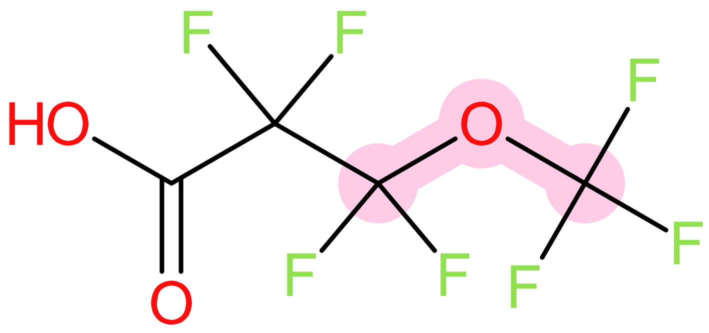

S56: Fluorinated triethylene glycol monomethyl ether (C7F3ETOH)
SMILES: OCC(F)(F)OC(F)(F)C(F)(F)OC(F)(F)C(F)(F)OC(F)(F)F

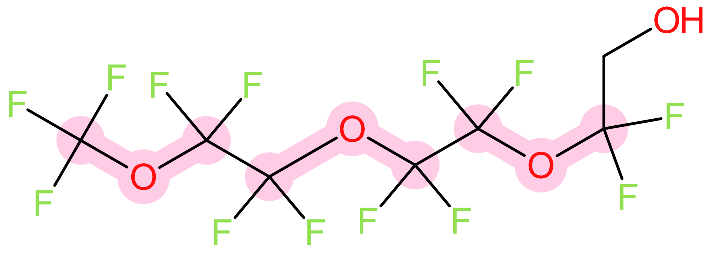

SGR10795

S7: Dodecafluoroheptanol (7H 6:1 FTOH)
SMILES: OCC(F)(F)C(F)(F)C(F)(F)C(F)(F)C(F)(F)C(F)F

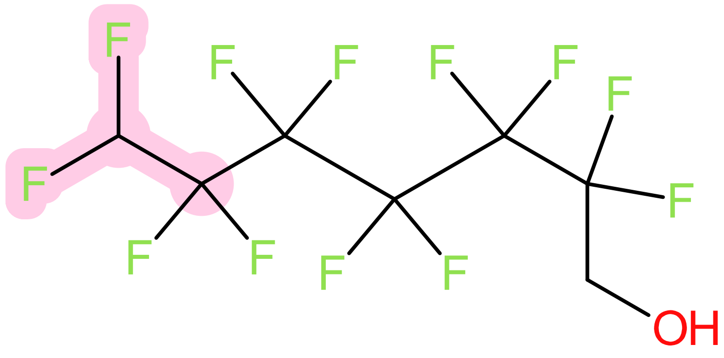

S16: Perfluoropentanamide (PFPAM)
SMILES: NC(=O)C(F)(F)C(F)(F)C(F)(F)C(F)F

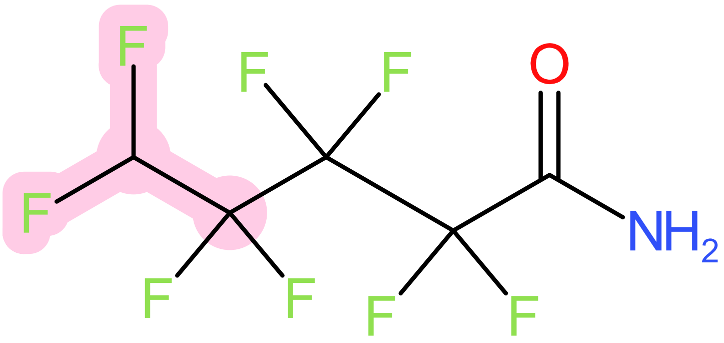

S17: 2,2,3,3,4,4-Hexafluorobutanoic acid (4H-PFBA)
SMILES: OC(=O)C(F)(F)C(F)(F)C(F)F

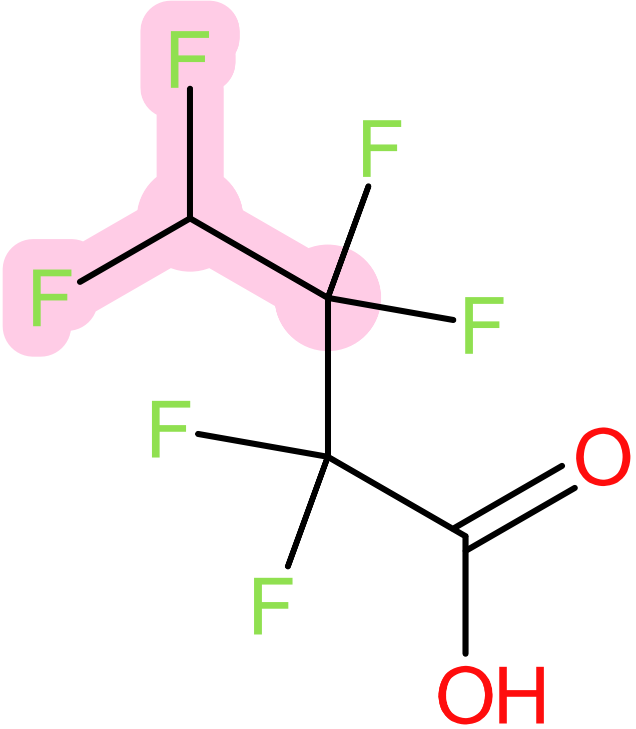

S23: 1H,1H,5H-Perfluoropentanol (PFPOH)
SMILES: OCC(F)(F)C(F)(F)C(F)(F)C(F)F

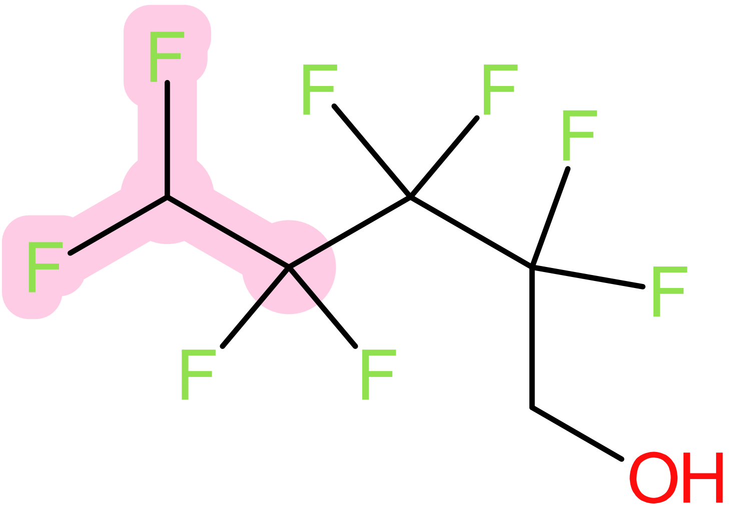

SGR10290

S2: 3-(Perfluoro-2-butyl)propane-1,2-diol (PFHp2OH)
SMILES: OCC(O)CC(F)(F)C(F)(F)C(F)(F)C(F)(F)F

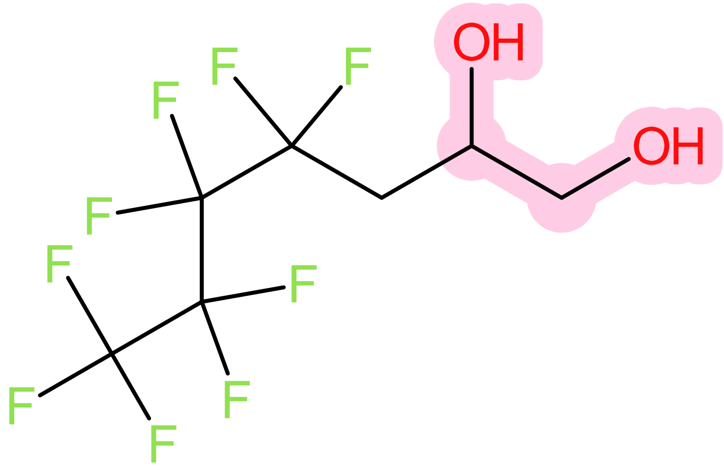

S40: 1-(Perfluorofluorooctyl)propane-2,3-diol (PFUd2OH)
SMILES: OCC(O)CC(F)(F)C(F)(F)C(F)(F)C(F)(F)C(F)(F)C(F)(F)C(F)(F)C(F)(F)F

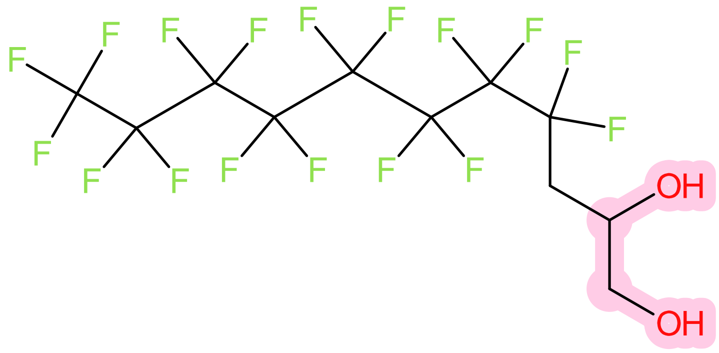

S56: Fluorinated triethylene glycol monomethyl ether (C7F3ETOH)

SMILES: OCC(F)(F)OC(F)(F)C(F)(F)OC(F)(F)C(F)(F)OC(F)(F)F

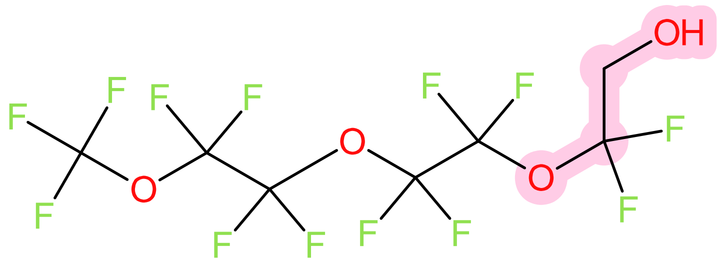

SGR10418

S1: 1-Pentafluoroethylethanol (PFBOH)
SMILES: CC(O)C(F)(F)C(F)(F)F

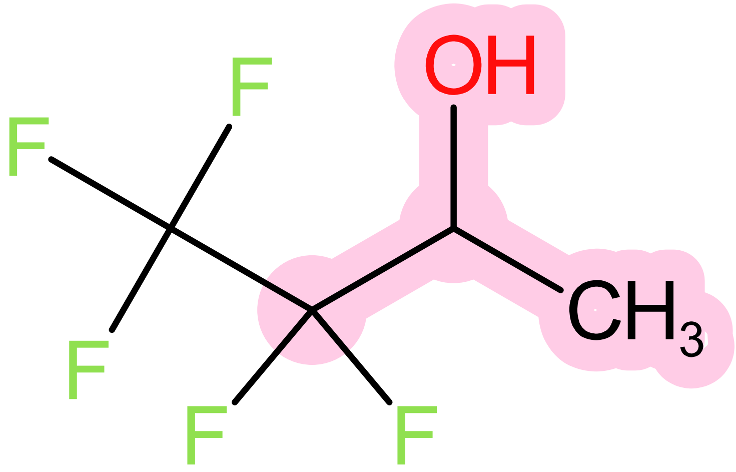

S2: 3-(Perfluoro-2-butyl)propane-1,2-diol (PFHp2OH)
SMILES: OCC(O)CC(F)(F)C(F)(F)C(F)(F)C(F)(F)F

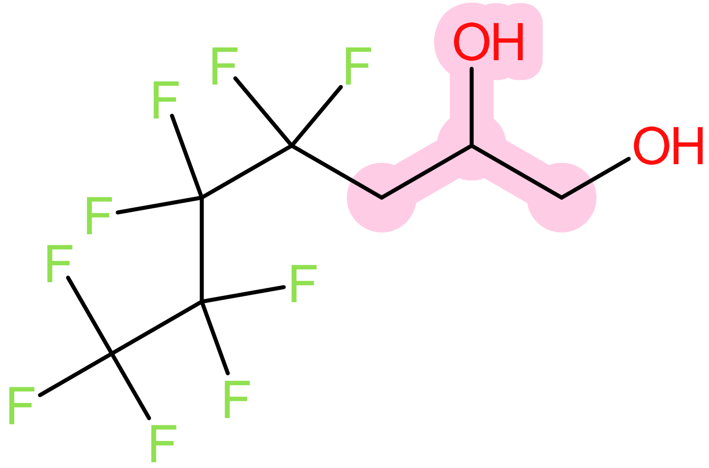

S40: 1-(Perfluorofluorooctyl)propane-2,3-diol (PFUd2OH)
SMILES: OCC(O)CC(F)(F)C(F)(F)C(F)(F)C(F)(F)C(F)(F)C(F)(F)C(F)(F)C(F)(F)F

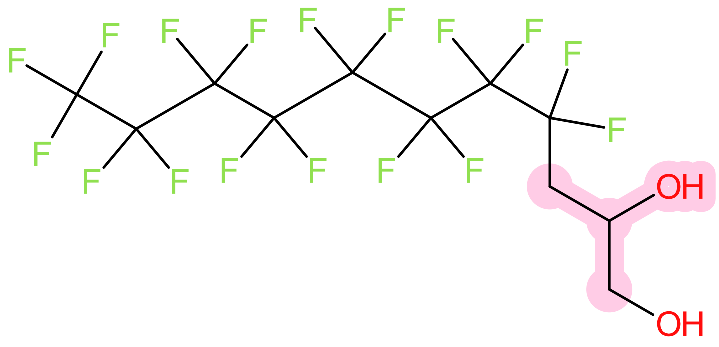

SGR10493

S1: 1-Pentafluoroethylethanol (PFBOH)
SMILES: CC(O)C(F)(F)C(F)(F)F

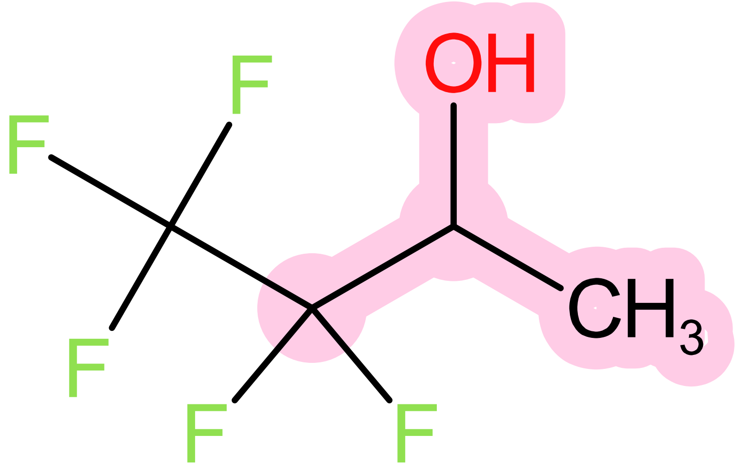

S2: 3-(Perfluoro-2-butyl)propane-1,2-diol (PFHp2OH)
SMILES: OCC(O)CC(F)(F)C(F)(F)C(F)(F)C(F)(F)F

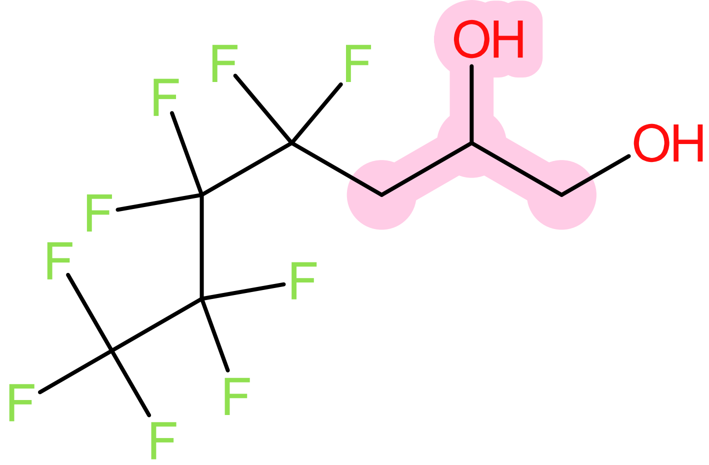

S40: 1-(Perfluorofluorooctyl)propane-2,3-diol (PFUd2OH)

SMILES: OCC(O)CC(F)(F)C(F)(F)C(F)(F)C(F)(F)C(F)(F)C(F)(F)C(F)(F)C(F)(F)F

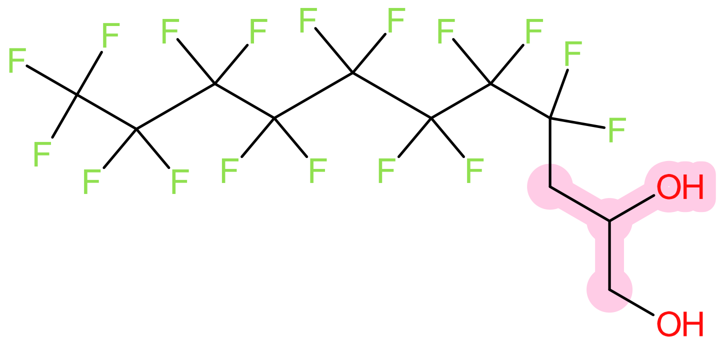

SGR10684

S1: 1-Pentafluoroethylethanol (PFBOH)
SMILES: CC(O)C(F)(F)C(F)(F)F

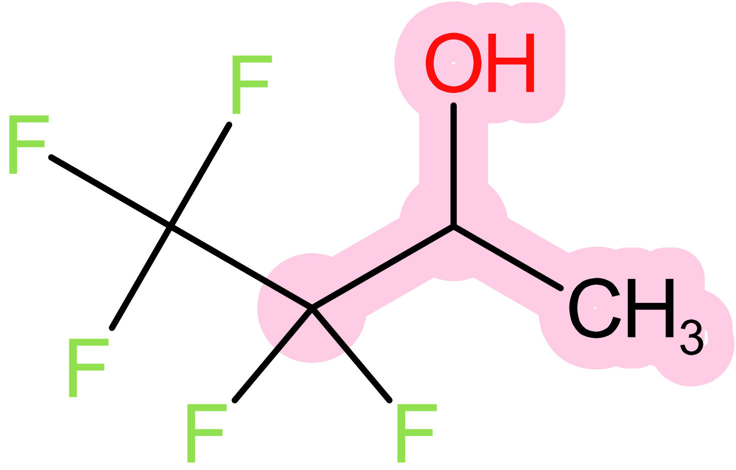

S2: 3-(Perfluoro-2-butyl)propane-1,2-diol (PFHp2OH)
SMILES: OCC(O)CC(F)(F)C(F)(F)C(F)(F)C(F)(F)F

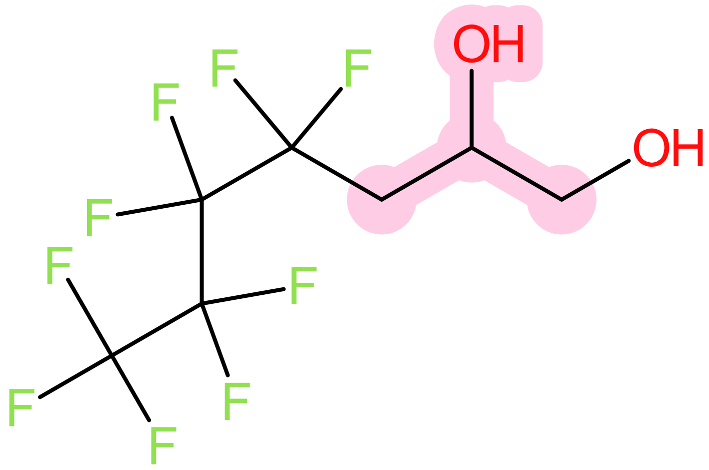

S40: 1-(Perfluorofluorooctyl)propane-2,3-diol (PFUd2OH)
SMILES: OCC(O)CC(F)(F)C(F)(F)C(F)(F)C(F)(F)C(F)(F)C(F)(F)C(F)(F)C(F)(F)F

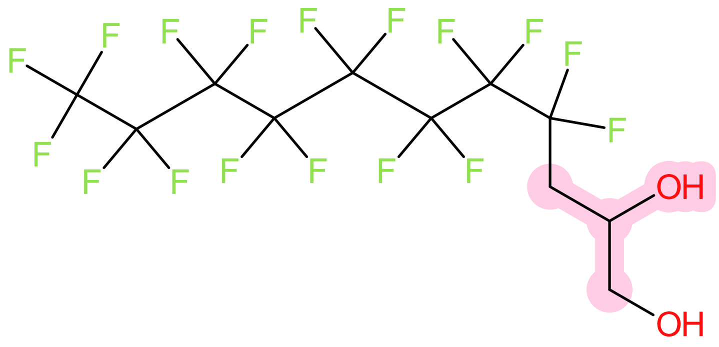

SGR10736

S1: 1-Pentafluoroethylethanol (PFBOH)
SMILES: CC(O)C(F)(F)C(F)(F)F

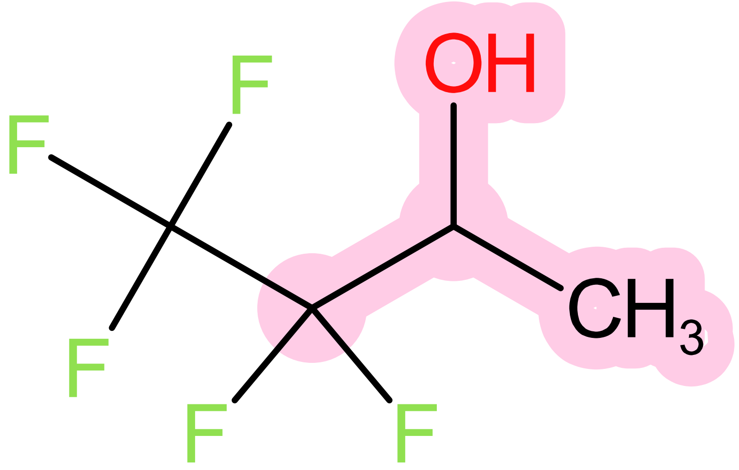

S2: 3-(Perfluoro-2-butyl)propane-1,2-diol (PFHp2OH)
SMILES: OCC(O)CC(F)(F)C(F)(F)C(F)(F)C(F)(F)F

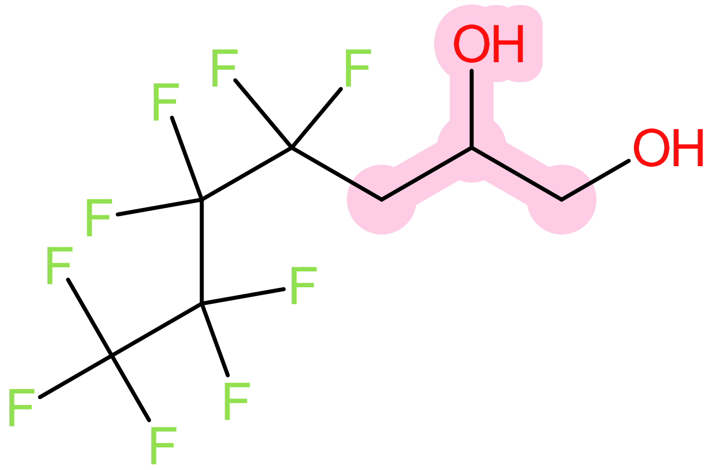

S40: 1-(Perfluorofluorooctyl)propane-2,3-diol (PFUd2OH)
SMILES: OCC(O)CC(F)(F)C(F)(F)C(F)(F)C(F)(F)C(F)(F)C(F)(F)C(F)(F)C(F)(F)F

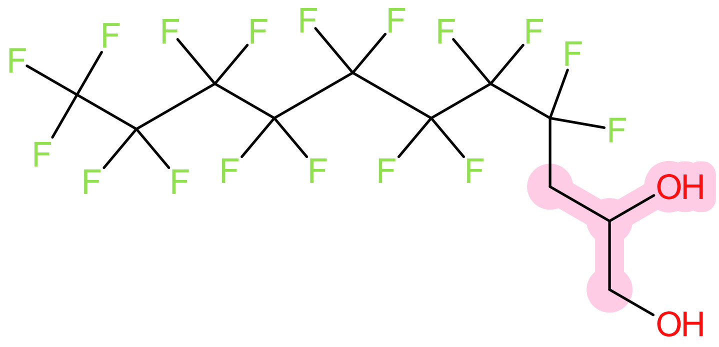

SGR10354

S2: 3-(Perfluoro-2-butyl)propane-1,2-diol (PFHp2OH)

SMILES: OCC(O)CC(F)(F)C(F)(F)C(F)(F)C(F)(F)F

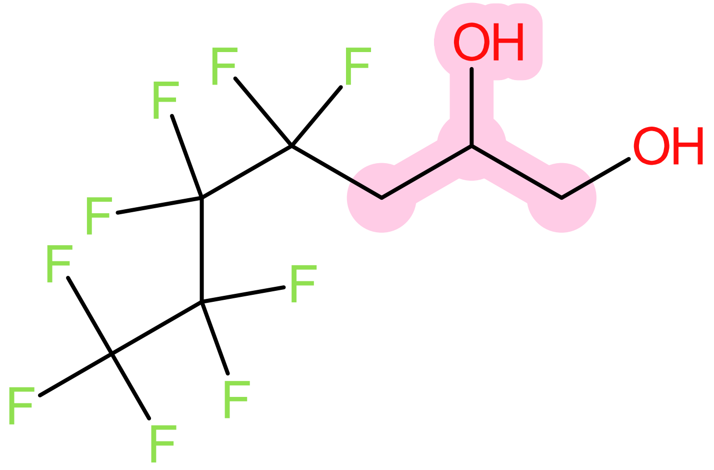

S40: 1-(Perfluorofluorooctyl)propane-2,3-diol (PFUd2OH)

SMILES: OCC(O)CC(F)(F)C(F)(F)C(F)(F)C(F)(F)C(F)(F)C(F)(F)C(F)(F)C(F)(F)F

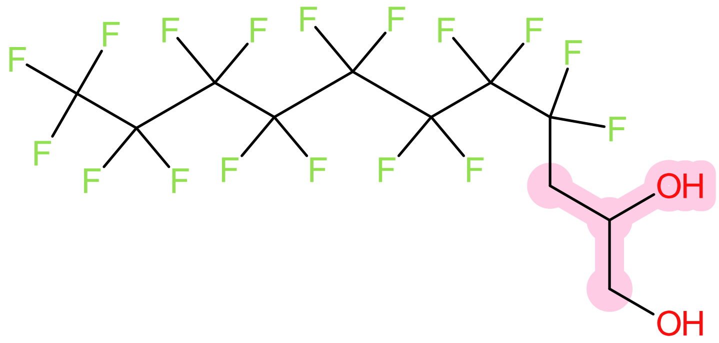

SGR10668

S39: Methyl perfluoro(3-(1-ethenyloxypropan-2-yloxy)propanoate) (MePF2ETOA)
SMILES: COC(=O)C(F)(F)C(F)(F)OC(F)(C(F)(F)F)C(F)(F)OC(F)=C(F)F

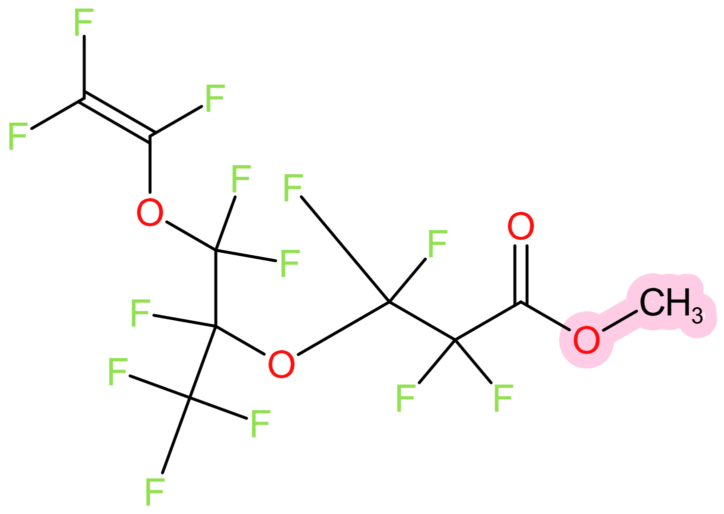

S43: N-Methyl-N-(2-hydroxyethyl)perfluorooctanesulfonamide (MeFOSE)
SMILES: CN(CCO)S(=O)(=O)C(F)(F)C(F)(F)C(F)(F)C(F)(F)C(F)(F)C(F)(F)C(F)(F)C(F)(F)F

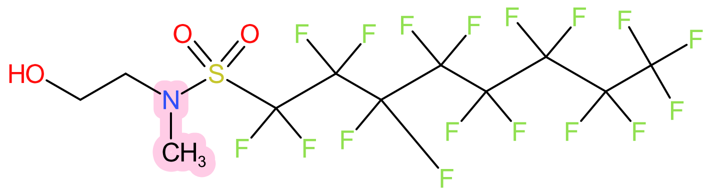

SGR10703

S31: Perfluorohexanesulfonamide (PFHxSA)
SMILES: NS(=O)(=O)C(F)(F)C(F)(F)C(F)(F)C(F)(F)C(F)(F)C(F)(F)F

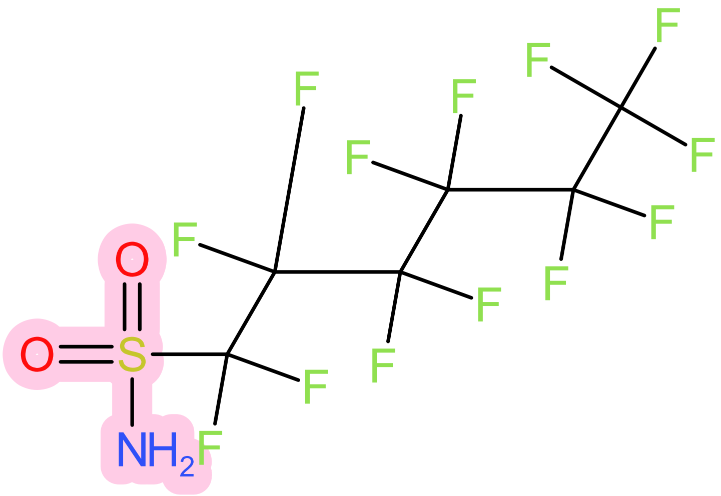

S43: N-Methyl-N-(2-hydroxyethyl)perfluorooctanesulfonamide (MeFOSE)
SMILES: CN(CCO)S(=O)(=O)C(F)(F)C(F)(F)C(F)(F)C(F)(F)C(F)(F)C(F)(F)C(F)(F)C(F)(F)F

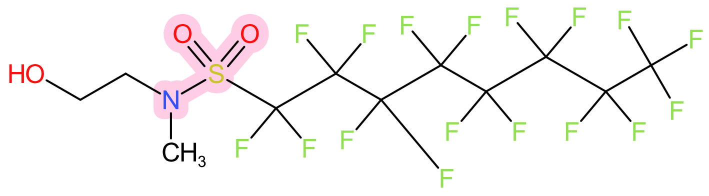

FUNCTIONAL GROUPS

SGR10072 (14 chem)
SGR10761 (14 chem)
SGR10295 (12 chem)
SGR10203 (8 chem)
SGR10153 (7 chem)
SGR10109 (3 chem)
SGR10343 (3 chem)
SGR10099 (2 chem)
SGR10289 (2 chem)
SGR10587 (2 chem)

SGR10072

S1: 1-Pentafluoroethylethanol (PFBOH)
SMILES: CC(O)C(F)(F)C(F)(F)F

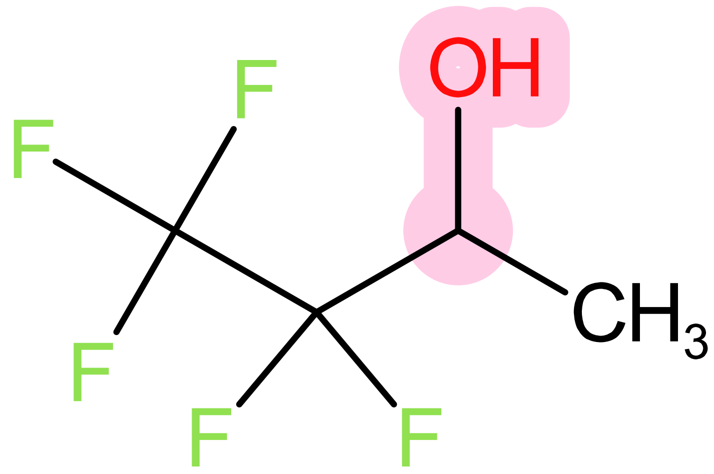

S2: 3-(Perfluoro-2-butyl)propane-1,2-diol (PFHp2OH)
SMILES: OCC(O)CC(F)(F)C(F)(F)C(F)(F)C(F)(F)F

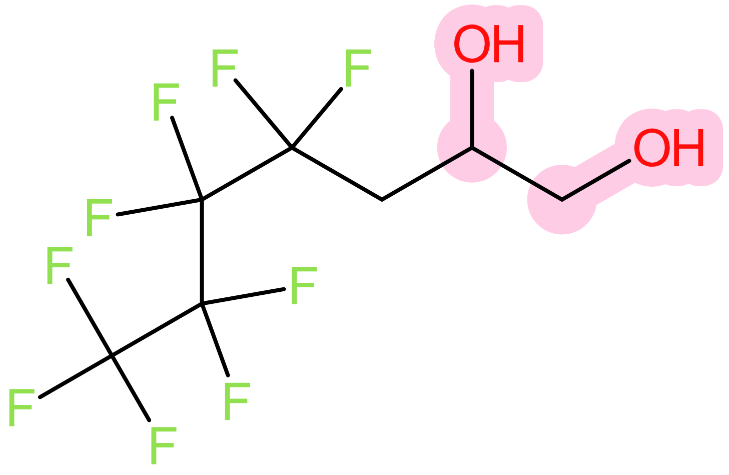

S7: Dodecafluoroheptanol (7H 6:1 FTOH)
SMILES: OCC(F)(F)C(F)(F)C(F)(F)C(F)(F)C(F)(F)C(F)F

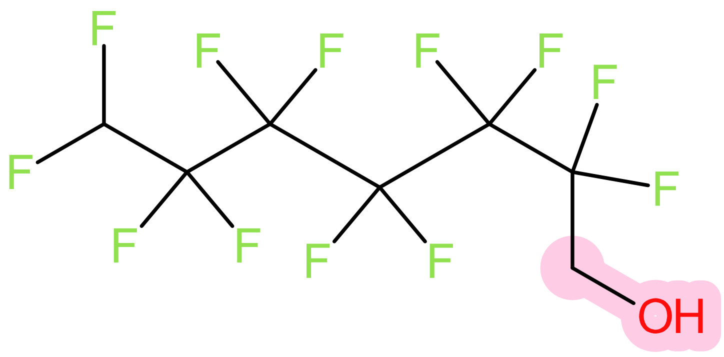

S12: Heptafluorobutanol (HpFBOH)
SMILES: OCC(F)(F)C(F)(F)C(F)(F)F

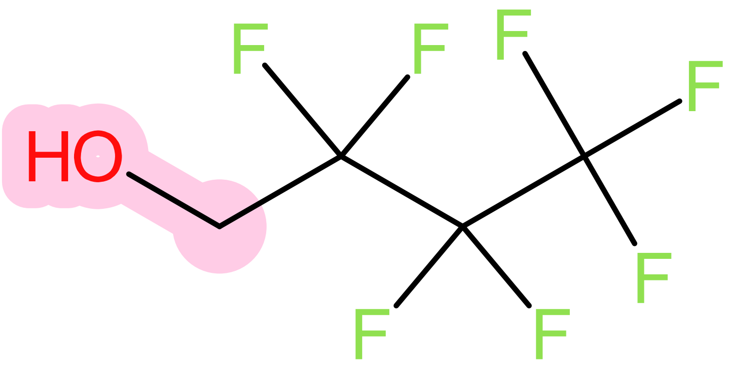

S14: Hexafluoroamylene glycol (CFHx2OH)
SMILES: OCC(F)(F)C(F)(F)C(F)(F)CO

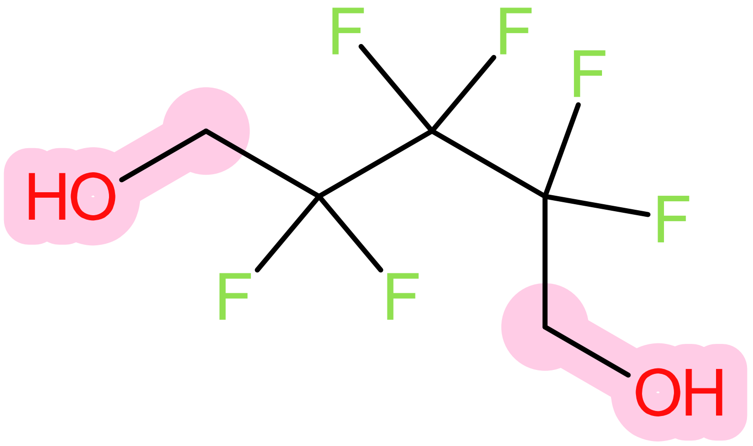

S20: 2-Aminohexafluoropropan-2-ol (AmFPrOH)
SMILES: NC(O)(C(F)(F)F)C(F)(F)F

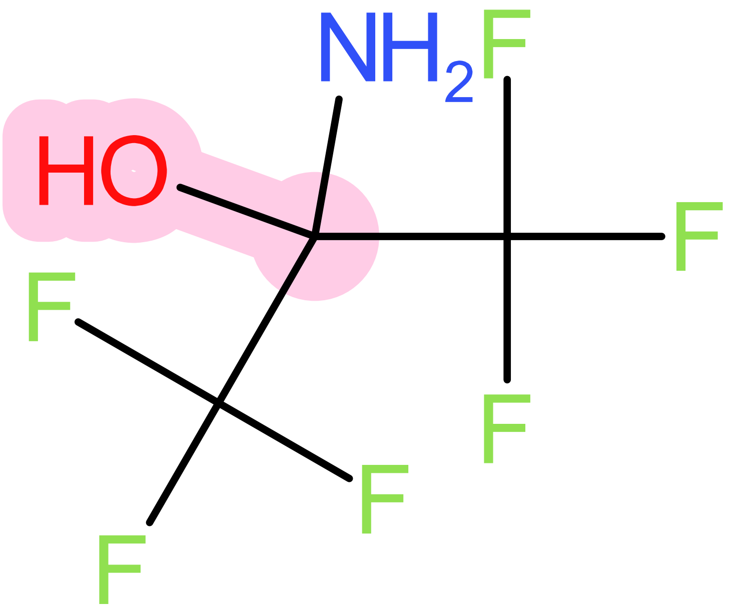

S21: 4:2 Fluorotelomer alcohol (4:2 FTOH)
SMILES: OCCC(F)(F)C(F)(F)C(F)(F)C(F)(F)F

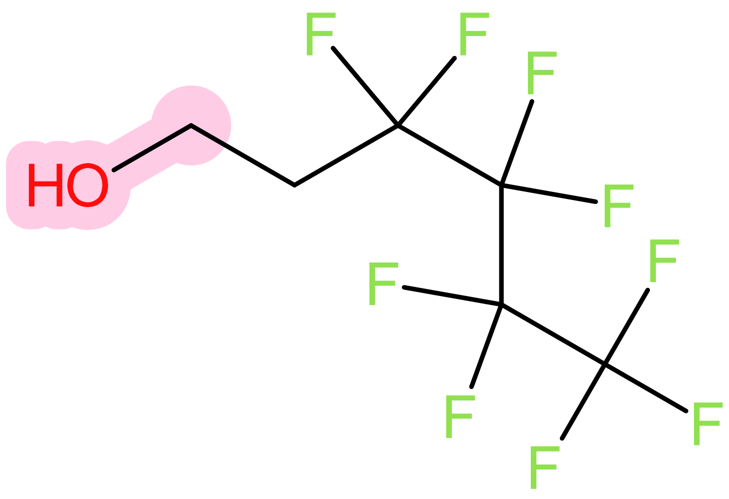

S23: 1H,1H,5H-Perfluoropentanol (PFPOH)
SMILES: OCC(F)(F)C(F)(F)C(F)(F)C(F)F

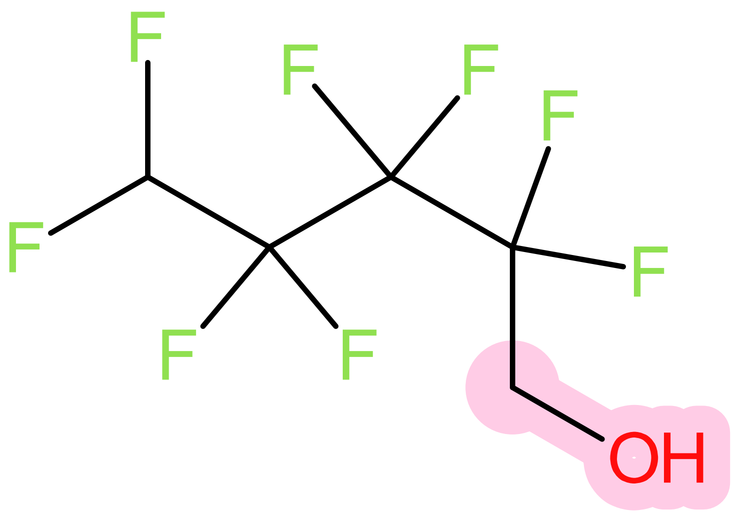

S24: 8:2 Fluorotelomer alcohol (8:2 FTOH)
SMILES: OCCC(F)(F)C(F)(F)C(F)(F)C(F)(F)C(F)(F)C(F)(F)C(F)(F)C(F)(F)F

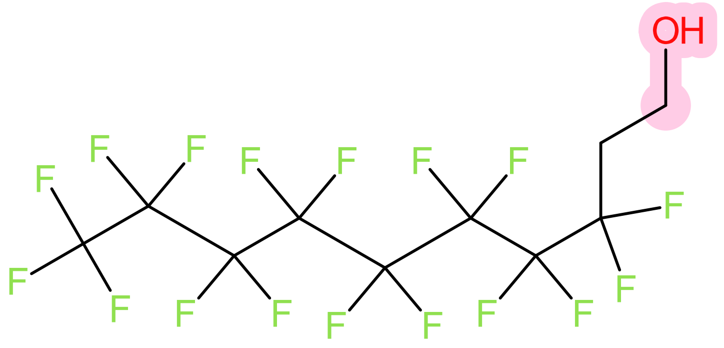

S26: 6:2 Fluorotelomer alcohol (6:2 FTOH)
SMILES: OCCC(F)(F)C(F)(F)C(F)(F)C(F)(F)C(F)(F)C(F)(F)F

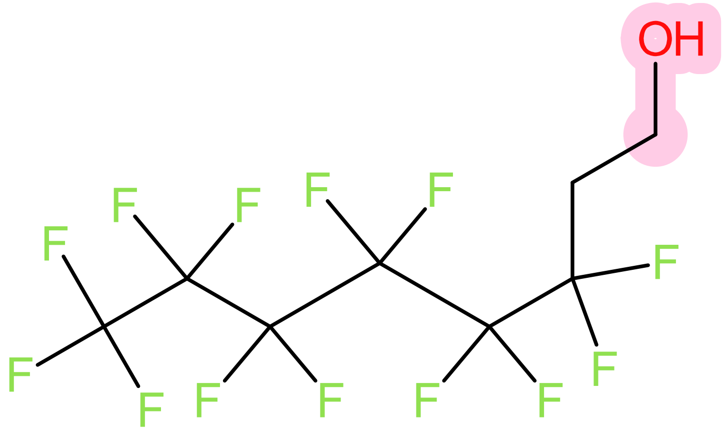

S40: 1-(Perfluorofluorooctyl)propane-2,3-diol (PFUd2OH)
SMILES: OCC(O)CC(F)(F)C(F)(F)C(F)(F)C(F)(F)C(F)(F)C(F)(F)C(F)(F)C(F)(F)F

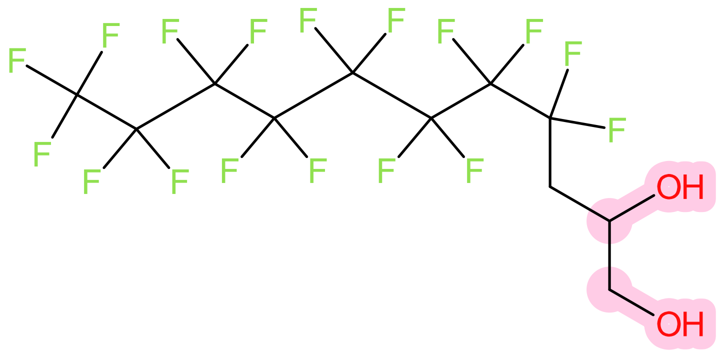

S43: N-Methyl-N-(2-hydroxyethyl)perfluorooctanesulfonamide (MeFOSE)
SMILES: CN(CCO)S(=O)(=O)C(F)(F)C(F)(F)C(F)(F)C(F)(F)C(F)(F)C(F)(F)C(F)(F)C(F)(F)F

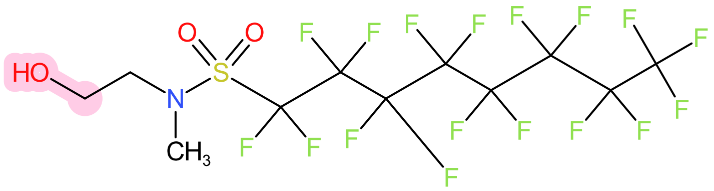

S47: 6:1 Fluorotelomer alcohol (6:1 FTOH)
SMILES: OCC(F)(F)C(F)(F)C(F)(F)C(F)(F)C(F)(F)C(F)(F)F

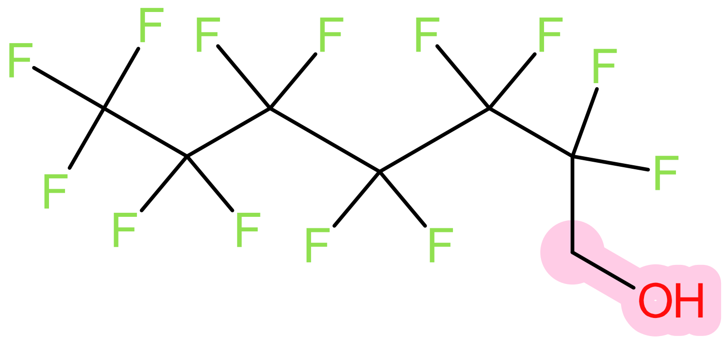

S56: Fluorinated triethylene glycol monomethyl ether (C7F3ETOH)
SMILES: OCC(F)(F)OC(F)(F)C(F)(F)OC(F)(F)C(F)(F)OC(F)(F)F

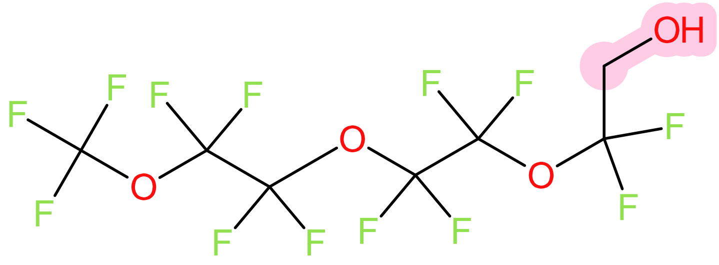

SGR10761

S1: 1-Pentafluoroethylethanol (PFBOH)
SMILES: CC(O)C(F)(F)C(F)(F)F

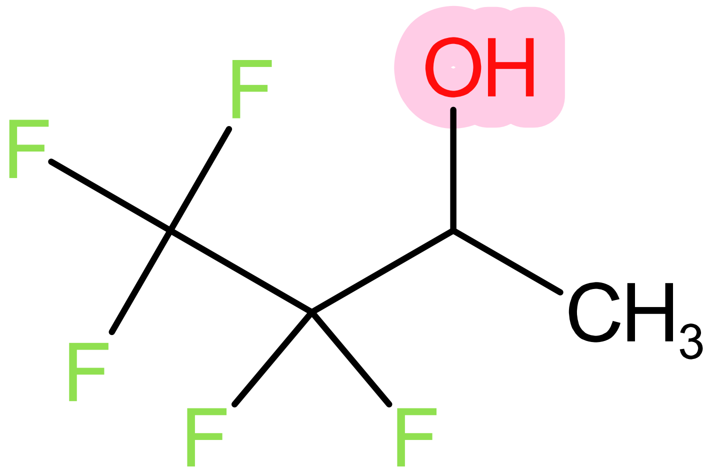

S2: 3-(Perfluoro-2-butyl)propane-1,2-diol (PFHp2OH)
SMILES: OCC(O)CC(F)(F)C(F)(F)C(F)(F)C(F)(F)F

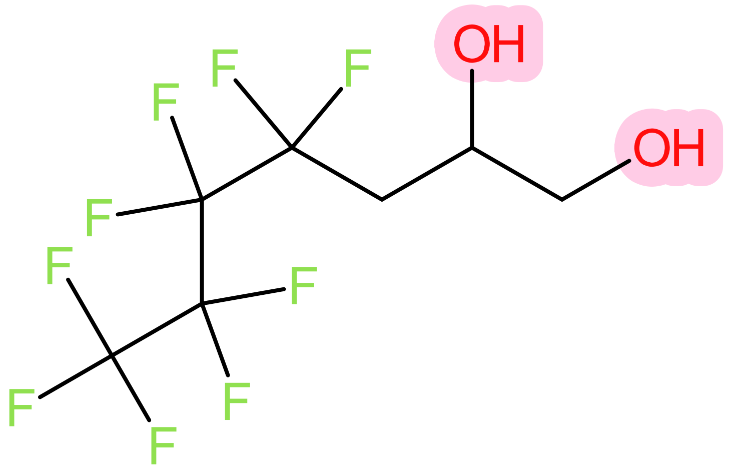

S7: Dodecafluoroheptanol (7H 6:1 FTOH)
SMILES: OCC(F)(F)C(F)(F)C(F)(F)C(F)(F)C(F)(F)C(F)F

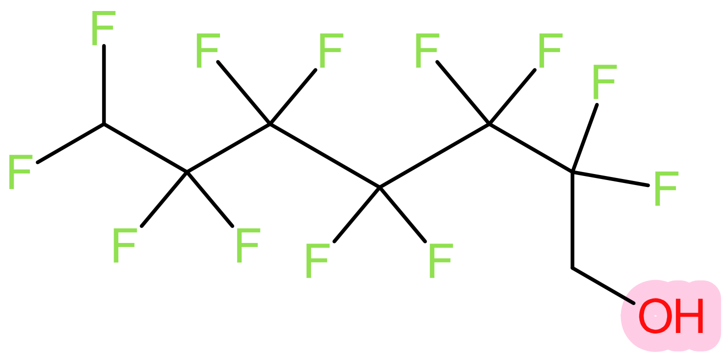

S12: Heptafluorobutanol (HpFBOH)
SMILES: OCC(F)(F)C(F)(F)C(F)(F)F

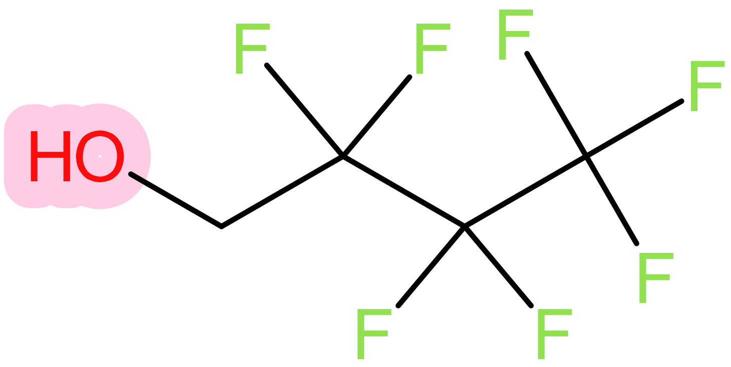

S14: Hexafluoroamylene glycol (CFHx2OH)
SMILES: OCC(F)(F)C(F)(F)C(F)(F)CO

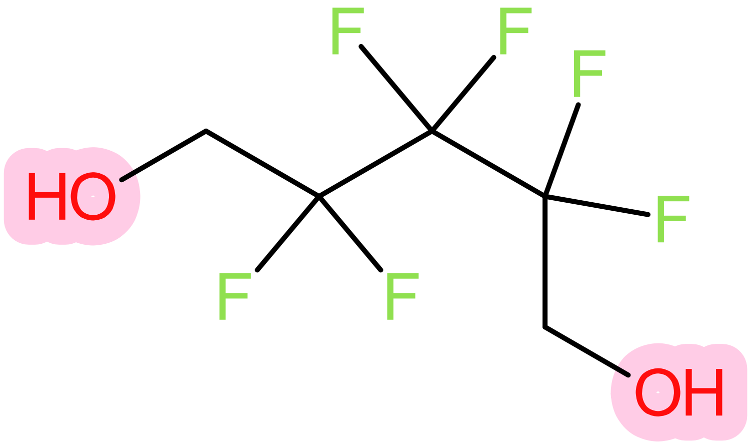

S20: 2-Aminohexafluoropropan-2-ol (AmFPrOH)
SMILES: NC(O)(C(F)(F)F)C(F)(F)F

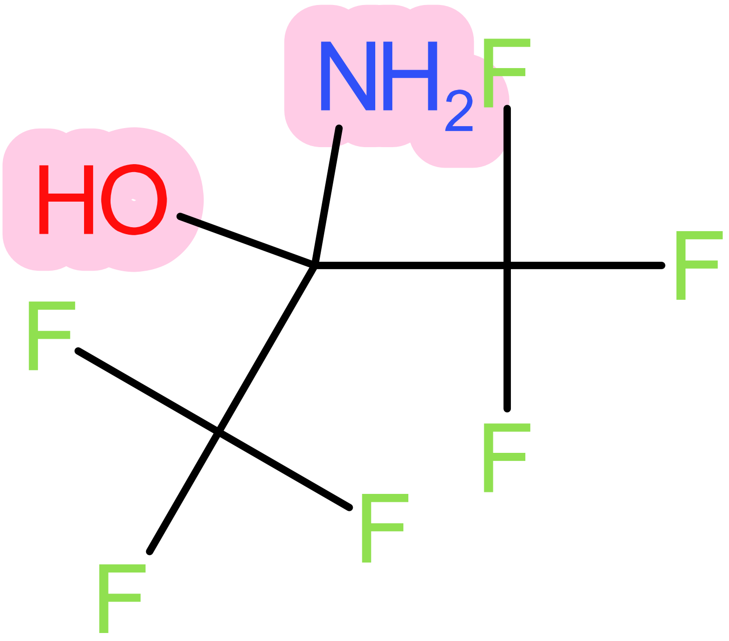

S21: 4:2 Fluorotelomer alcohol (4:2 FTOH)
SMILES: OCCC(F)(F)C(F)(F)C(F)(F)C(F)(F)F

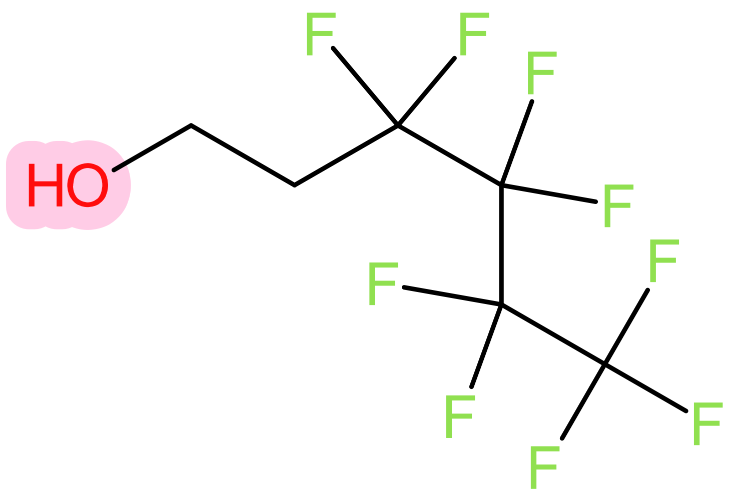

S23: 1H,1H,5H-Perfluoropentanol (PFPOH)
SMILES: OCC(F)(F)C(F)(F)C(F)(F)C(F)F

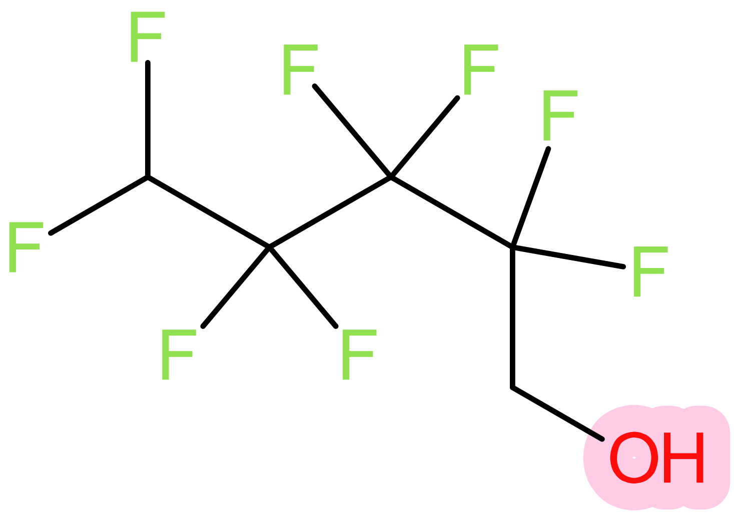

S24: 8:2 Fluorotelomer alcohol (8:2 FTOH)
SMILES: OCCC(F)(F)C(F)(F)C(F)(F)C(F)(F)C(F)(F)C(F)(F)C(F)(F)C(F)(F)F

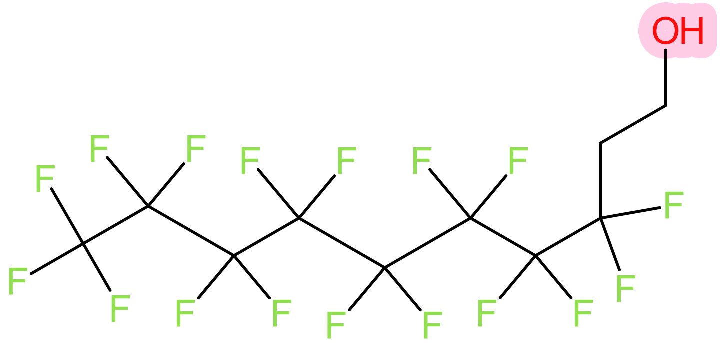

S26: 6:2 Fluorotelomer alcohol (6:2 FTOH)
SMILES: OCCC(F)(F)C(F)(F)C(F)(F)C(F)(F)C(F)(F)C(F)(F)F

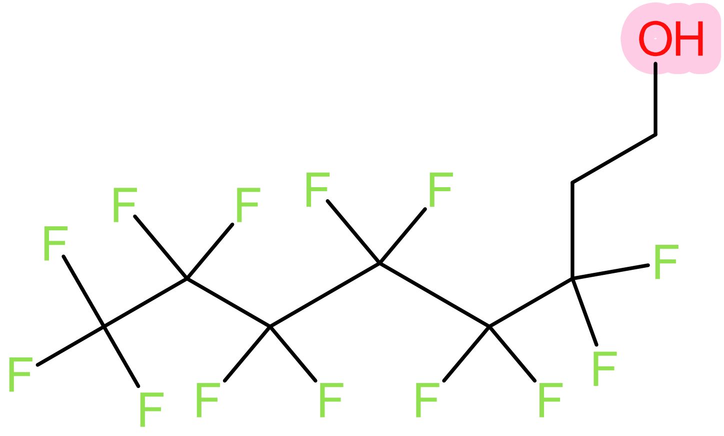

S40: 1-(Perfluorofluorooctyl)propane-2,3-diol (PFUd2OH)
SMILES: OCC(O)CC(F)(F)C(F)(F)C(F)(F)C(F)(F)C(F)(F)C(F)(F)C(F)(F)C(F)(F)F

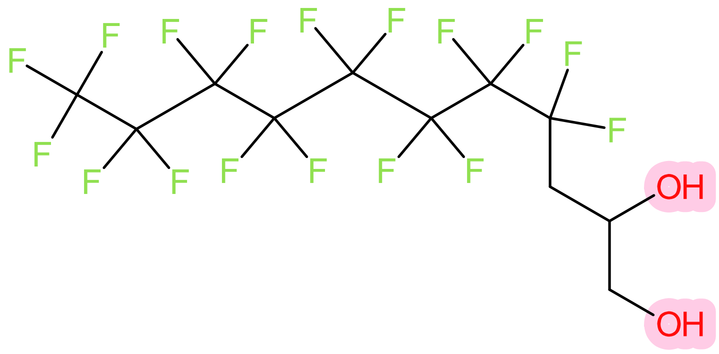

S43: N-Methyl-N-(2-hydroxyethyl)perfluorooctanesulfonamide (MeFOSE)
SMILES: CN(CCO)S(=O)(=O)C(F)(F)C(F)(F)C(F)(F)C(F)(F)C(F)(F)C(F)(F)C(F)(F)C(F)(F)F

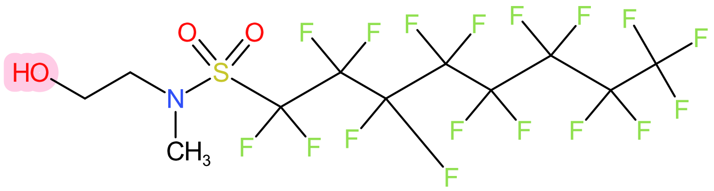

S47: 6:1 Fluorotelomer alcohol (6:1 FTOH)
SMILES: OCC(F)(F)C(F)(F)C(F)(F)C(F)(F)C(F)(F)C(F)(F)F

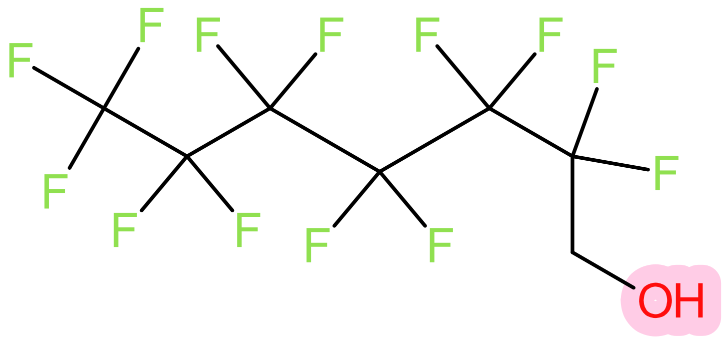

S56: Fluorinated triethylene glycol monomethyl ether (C7F3ETOH)
SMILES: OCC(F)(F)OC(F)(F)C(F)(F)OC(F)(F)C(F)(F)OC(F)(F)F

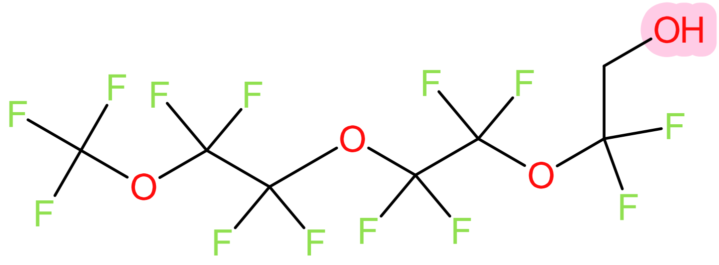

SGR10295

S2: 3-(Perfluoro-2-butyl)propane-1,2-diol (PFHp2OH)
SMILES: OCC(O)CC(F)(F)C(F)(F)C(F)(F)C(F)(F)F

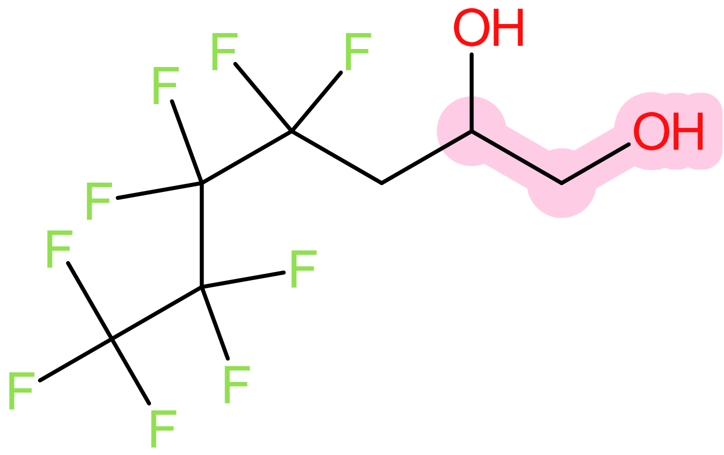

S7: Dodecafluoroheptanol (7H 6:1 FTOH)
SMILES: OCC(F)(F)C(F)(F)C(F)(F)C(F)(F)C(F)(F)C(F)F

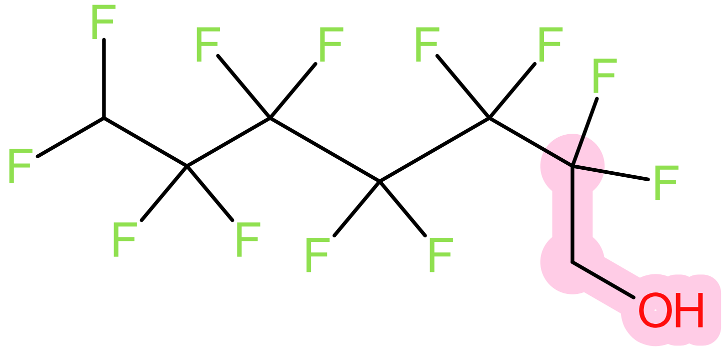

S12: Heptafluorobutanol (HpFBOH)
SMILES: OCC(F)(F)C(F)(F)C(F)(F)F

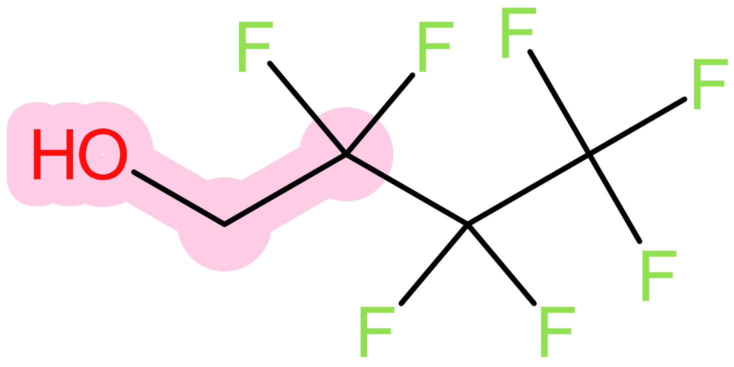

S14: Hexafluoroamylene glycol (CFHx2OH)
SMILES: OCC(F)(F)C(F)(F)C(F)(F)CO

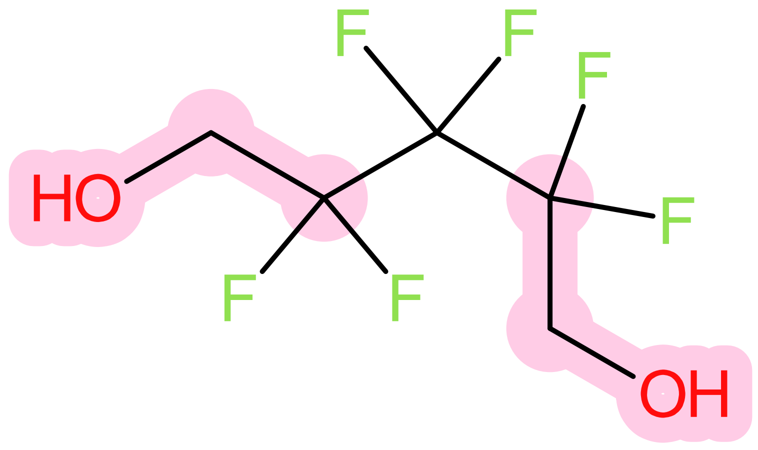

S21: 4:2 Fluorotelomer alcohol (4:2 FTOH)
SMILES: OCCC(F)(F)C(F)(F)C(F)(F)C(F)(F)F

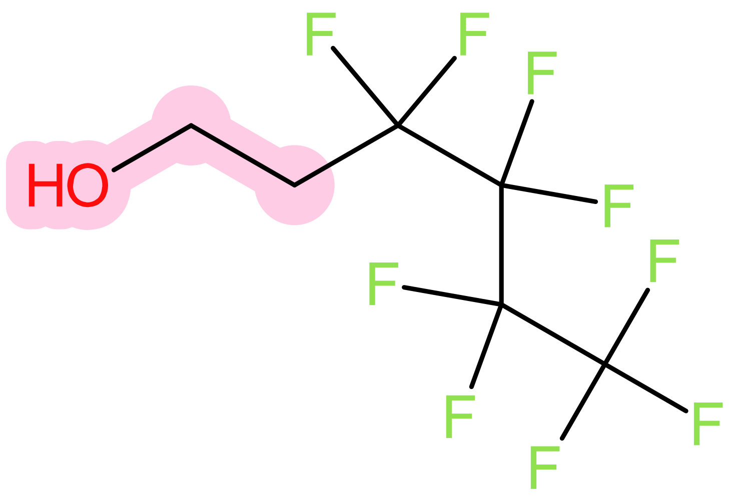

S23: 1H,1H,5H-Perfluoropentanol (PFPOH)
SMILES: OCC(F)(F)C(F)(F)C(F)(F)C(F)F

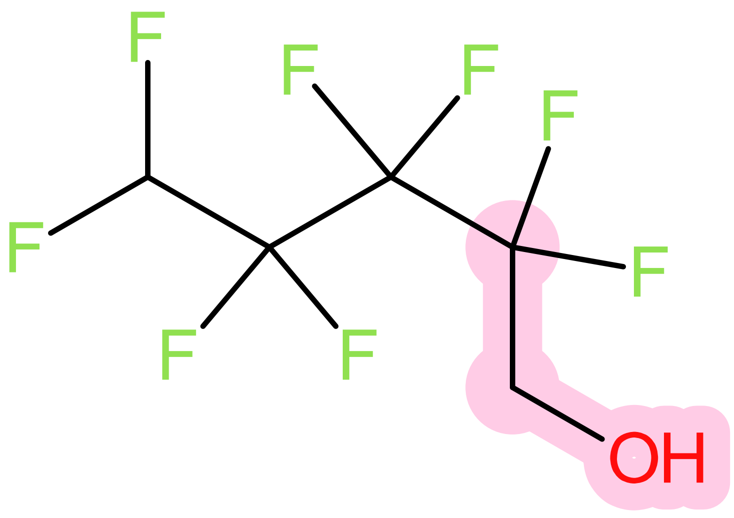

S24: 8:2 Fluorotelomer alcohol (8:2 FTOH)
SMILES: OCCC(F)(F)C(F)(F)C(F)(F)C(F)(F)C(F)(F)C(F)(F)C(F)(F)C(F)(F)F

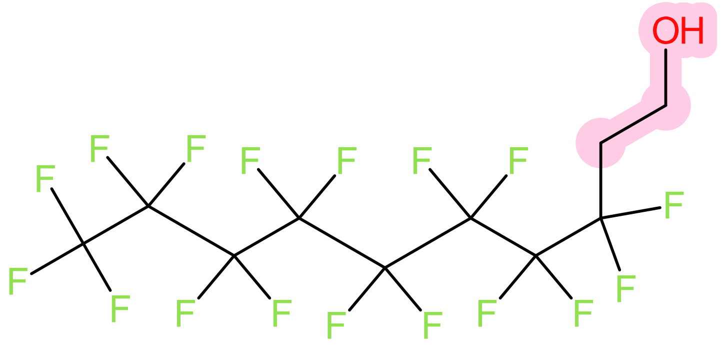

S26: 6:2 Fluorotelomer alcohol (6:2 FTOH)
SMILES: OCCC(F)(F)C(F)(F)C(F)(F)C(F)(F)C(F)(F)C(F)(F)F

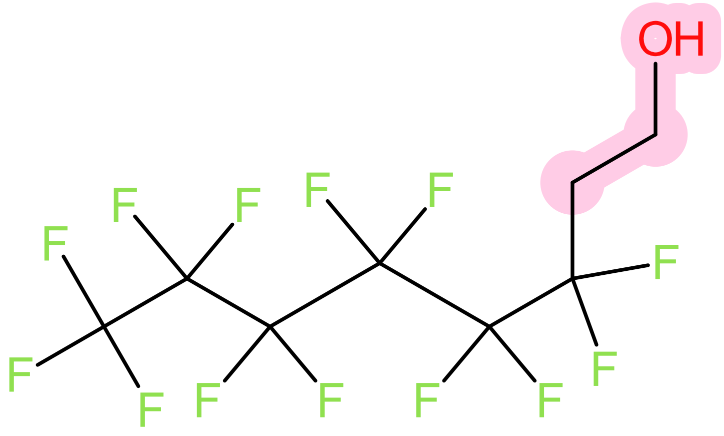

S40: 1-(Perfluorofluorooctyl)propane-2,3-diol (PFUd2OH)
SMILES: OCC(O)CC(F)(F)C(F)(F)C(F)(F)C(F)(F)C(F)(F)C(F)(F)C(F)(F)C(F)(F)F

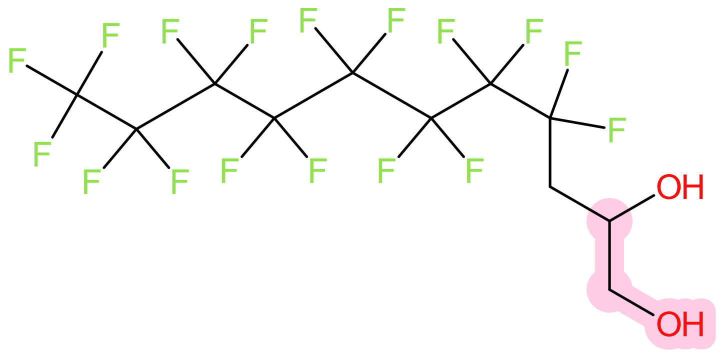

S43: N-Methyl-N-(2-hydroxyethyl)perfluorooctanesulfonamide (MeFOSE)
SMILES: CN(CCO)S(=O)(=O)C(F)(F)C(F)(F)C(F)(F)C(F)(F)C(F)(F)C(F)(F)C(F)(F)C(F)(F)F

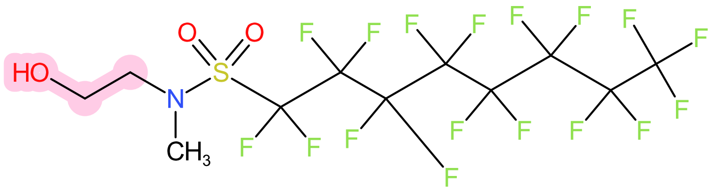

S47: 6:1 Fluorotelomer alcohol (6:1 FTOH)
SMILES: OCC(F)(F)C(F)(F)C(F)(F)C(F)(F)C(F)(F)C(F)(F)F

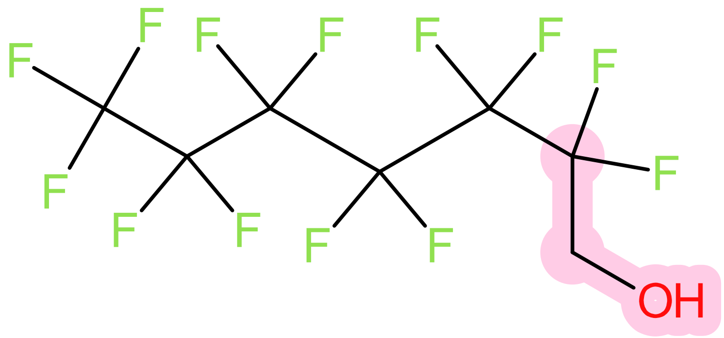

S56: Fluorinated triethylene glycol monomethyl ether (C7F3ETOH)
SMILES: OCC(F)(F)OC(F)(F)C(F)(F)OC(F)(F)C(F)(F)OC(F)(F)F

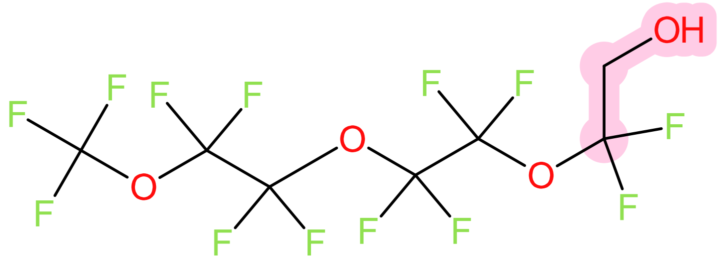

SGR10203

S3: Perfluoro-3,6-dioxaoctane-1,8-dioic acid (PFHx2Et2OA)
SMILES: OC(=O)C(F)(F)OC(F)(F)C(F)(F)OC(F)(F)C(O)=O

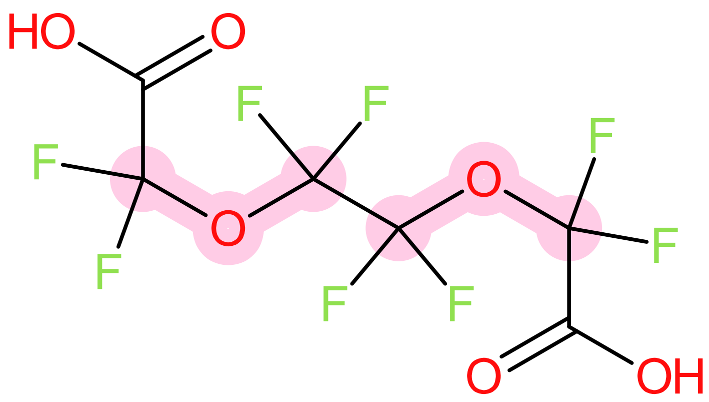

S9: Perfluoro(4-methoxybutanoic) acid (PFMBA)
SMILES: OC(=O)C(F)(F)C(F)(F)C(F)(F)OC(F)(F)F

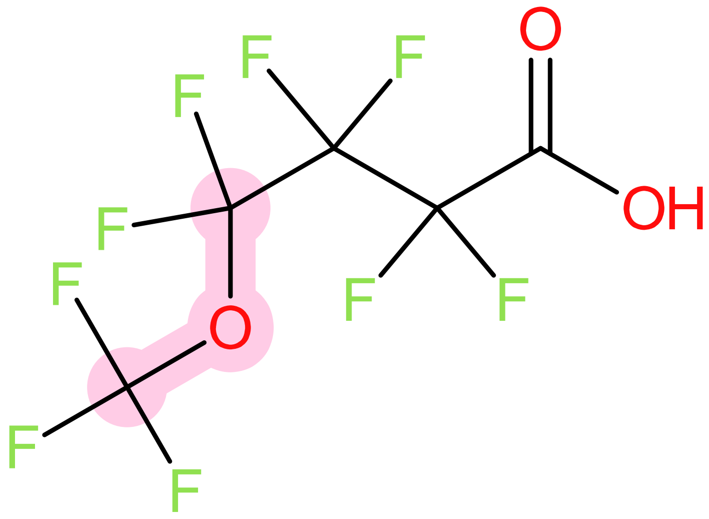

S11: Perfluoro-3,6-dioxaheptanoic acid (NFDHA)
SMILES: OC(=O)C(F)(F)OC(F)(F)C(F)(F)OC(F)(F)F

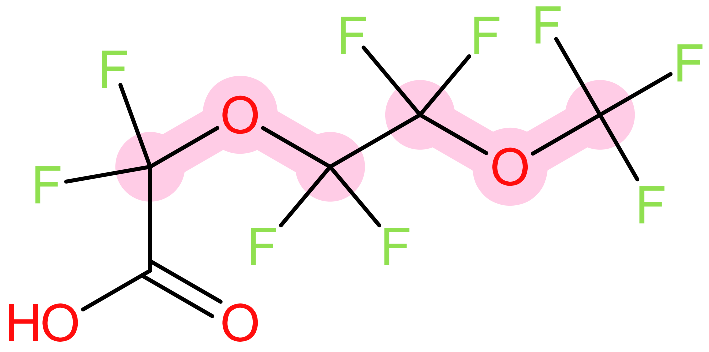

S22: Perfluoro-3,6,9-trioxatridecanoic acid (PFPE-6)
SMILES: OC(=O)C(F)(F)OC(F)(F)C(F)(F)OC(F)(F)C(F)(F)OC(F)(F)C(F)(F)C(F)(F)C(F)(F)F

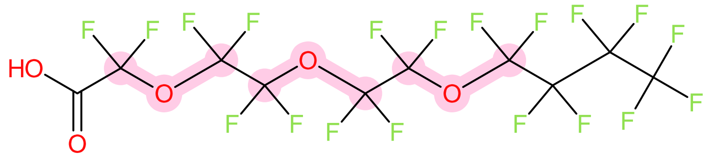

S36: Perfluoro-4-isopropoxybutanoic acid (PFPE-1)
SMILES: OC(=O)C(F)(F)C(F)(F)C(F)(F)OC(F)(C(F)(F)F)C(F)(F)F

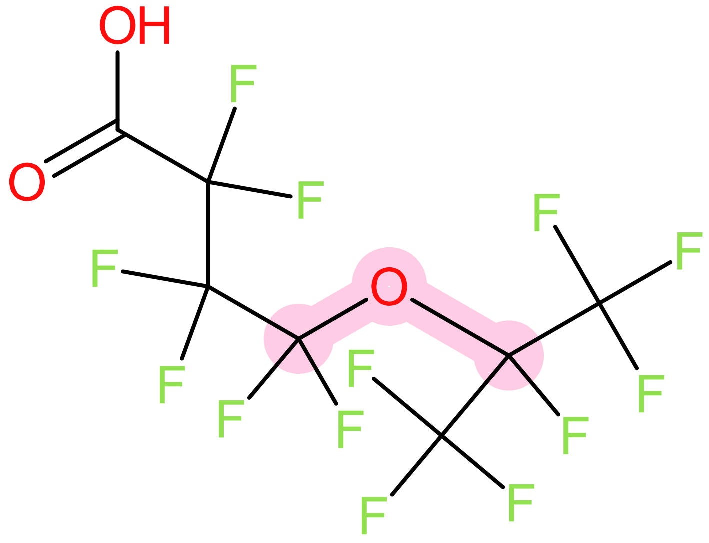

S39: Methyl perfluoro(3-(1-ethenyloxypropan-2-yloxy)propanoate) (MePF2ETOA)
SMILES: COC(=O)C(F)(F)C(F)(F)OC(F)(C(F)(F)F)C(F)(F)OC(F)=C(F)F

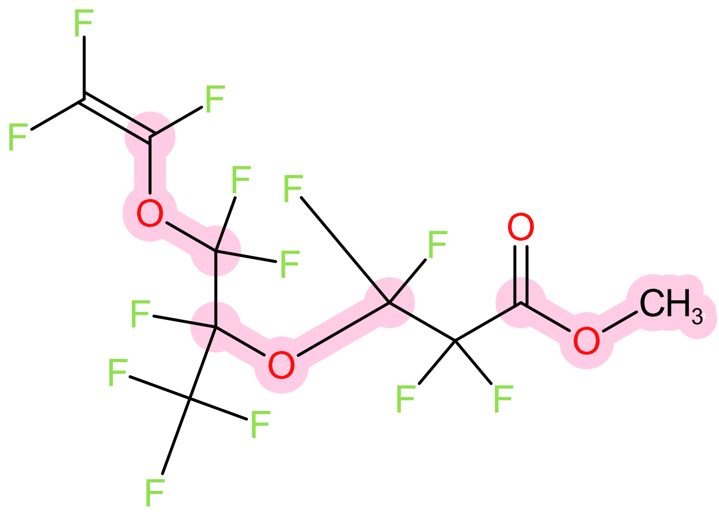

S49: Perfluoro-3-methoxypropanoic acid (PFMPA)
SMILES: OC(=O)C(F)(F)C(F)(F)OC(F)(F)F

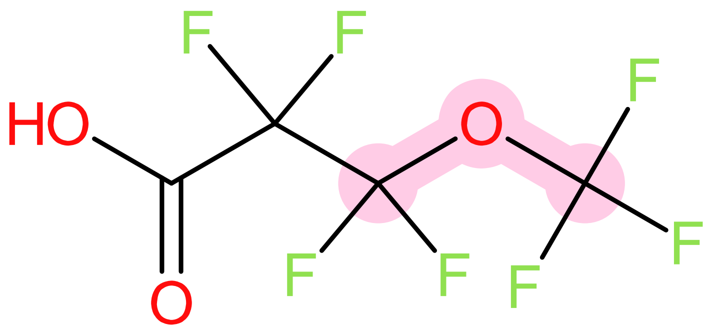

S56: Fluorinated triethylene glycol monomethyl ether (C7F3ETOH)
ID_56 OCC(F)(F)OC(F)(F)C(F)(F)OC(F)(F)C(F)(F)OC(F)(F)F

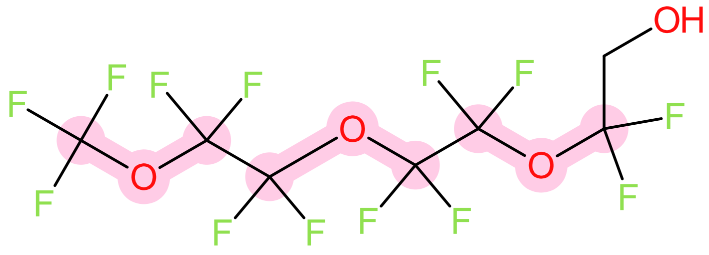

SGR10153

S10: Perfluorobutanesulfonic acid (PFBS)
SMILES: OS(=O)(=O)C(F)(F)C(F)(F)C(F)(F)C(F)(F)F

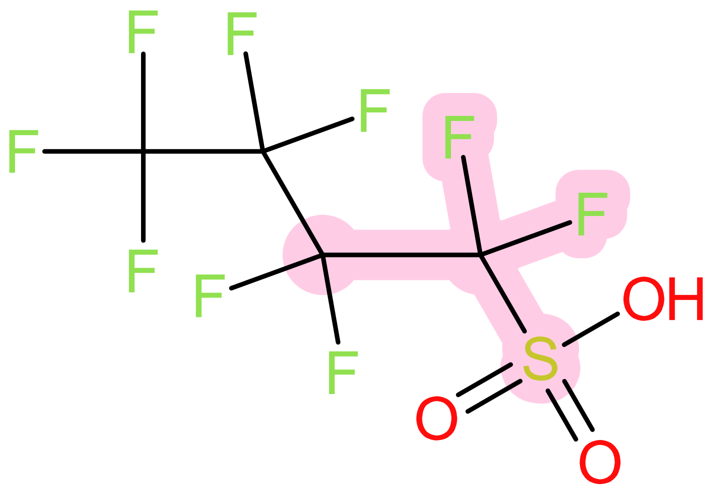

S29: Potassium perfluorobutanesulfonate (PFBS-K)
SMILES: OS(=O)(=O)C(F)(F)C(F)(F)C(F)(F)C(F)(F)F

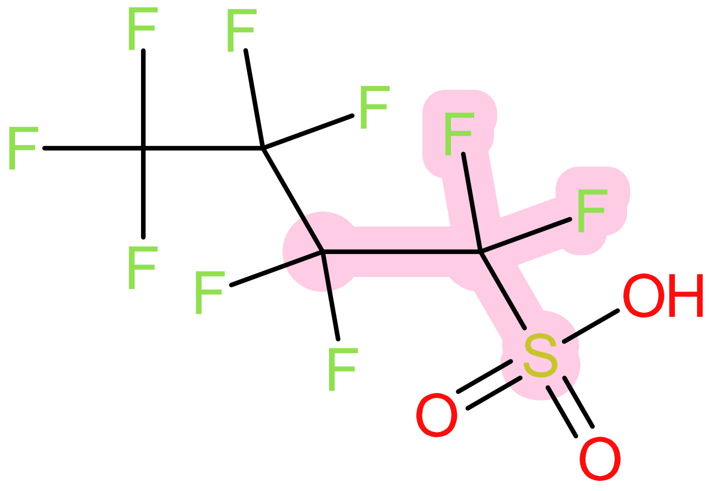

S31: Perfluorohexanesulfonamide (PFHxSA)
SMILES: NS(=O)(=O)C(F)(F)C(F)(F)C(F)(F)C(F)(F)C(F)(F)C(F)(F)F

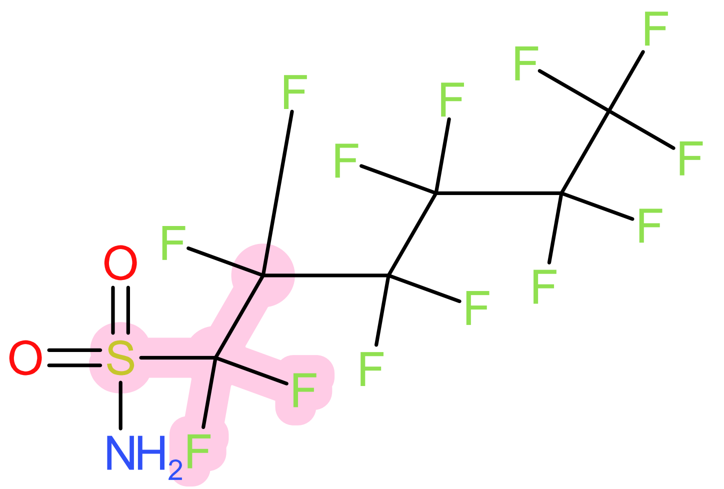

S37: Perfluorooctanesulfonic acid (PFOS)
SMILES: OS(=O)(=O)C(F)(F)C(F)(F)C(F)(F)C(F)(F)C(F)(F)C(F)(F)C(F)(F)C(F)(F)F

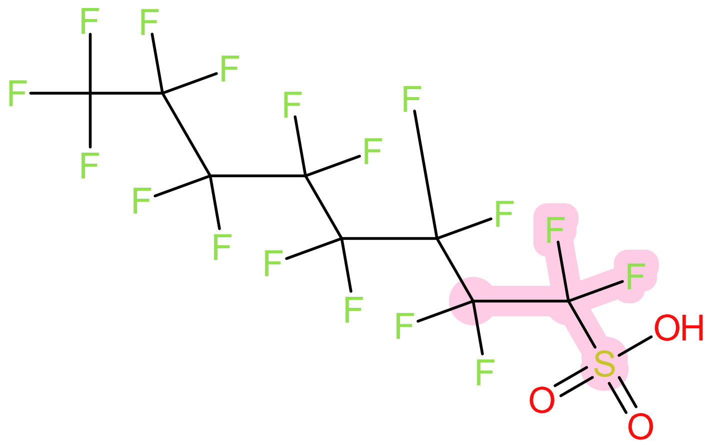

S38: 2,2,2-Trifluoroethyl perfluorobutanesulfonate (ET-PFBS)

SMILES: FC(F)(F)COS(=O)(=O)C(F)(F)C(F)(F)C(F)(F)C(F)(F)F

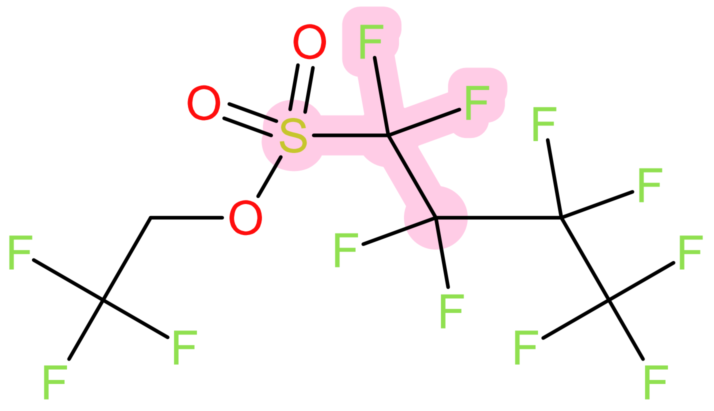

S43: N-Methyl-N-(2-hydroxyethyl)perfluorooctanesulfonamide (MeFOSE)

SMILES: CN(CCO)S(=O)(=O)C(F)(F)C(F)(F)C(F)(F)C(F)(F)C(F)(F)C(F)(F)C(F)(F)C(F)(F)F

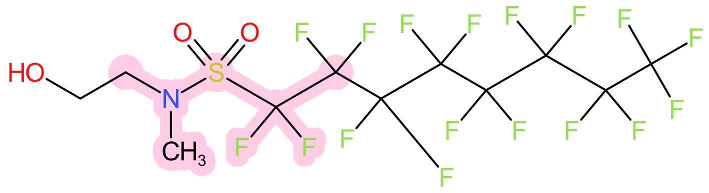

S51: Perfluorohexanesulfonic acid (PFHxS)

SMILES: OS(=O)(=O)C(F)(F)C(F)(F)C(F)(F)C(F)(F)C(F)(F)C(F)(F)F

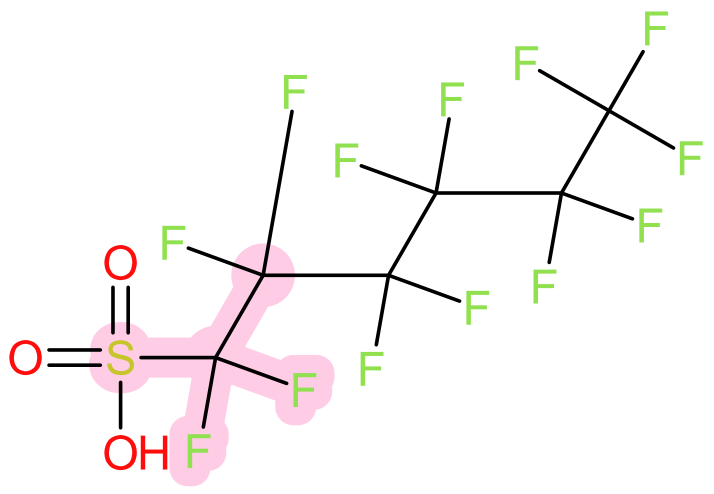

SGR10109

S1: 1-Pentafluoroethylethanol (PFBOH)
SMILES: CC(O)C(F)(F)C(F)(F)F

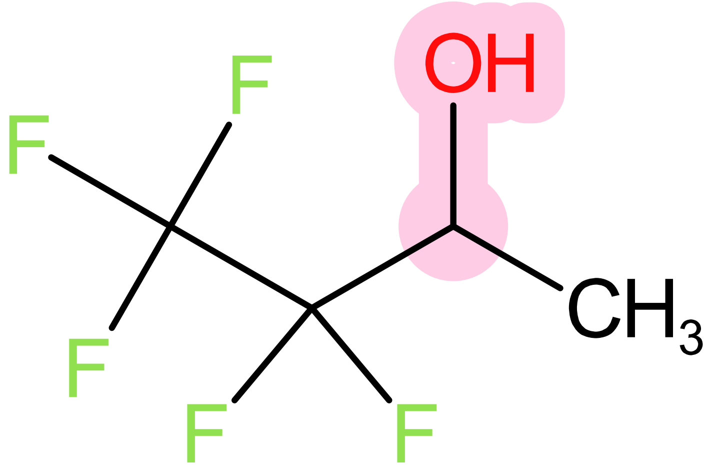

S2: 3-(Perfluoro-2-butyl)propane-1,2-diol (PFHp2OH)
SMILES: OCC(O)CC(F)(F)C(F)(F)C(F)(F)C(F)(F)F

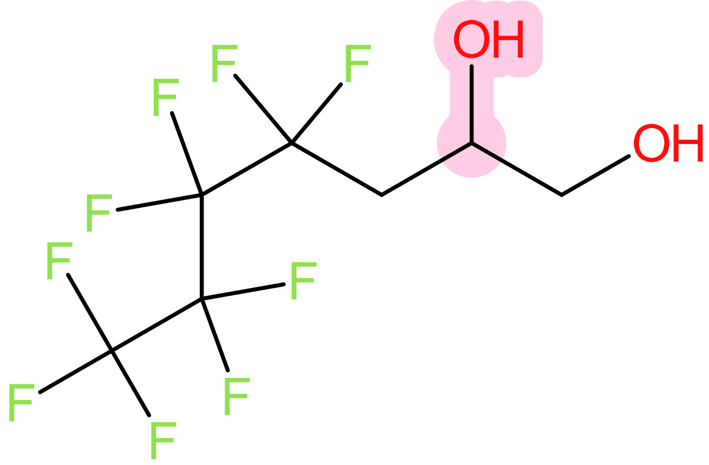

S40: 1-(Perfluorofluorooctyl)propane-2,3-diol (PFUd2OH)

SMILES: OCC(O)CC(F)(F)C(F)(F)C(F)(F)C(F)(F)C(F)(F)C(F)(F)C(F)(F)C(F)(F)F

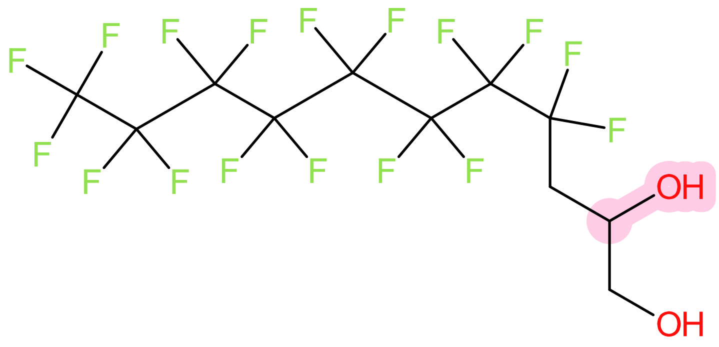

SGR10343

S20: 2-Aminohexafluoropropan-2-ol (AmFPrOH)
SMILES: NC(O)(C(F)(F)F)C(F)(F)F

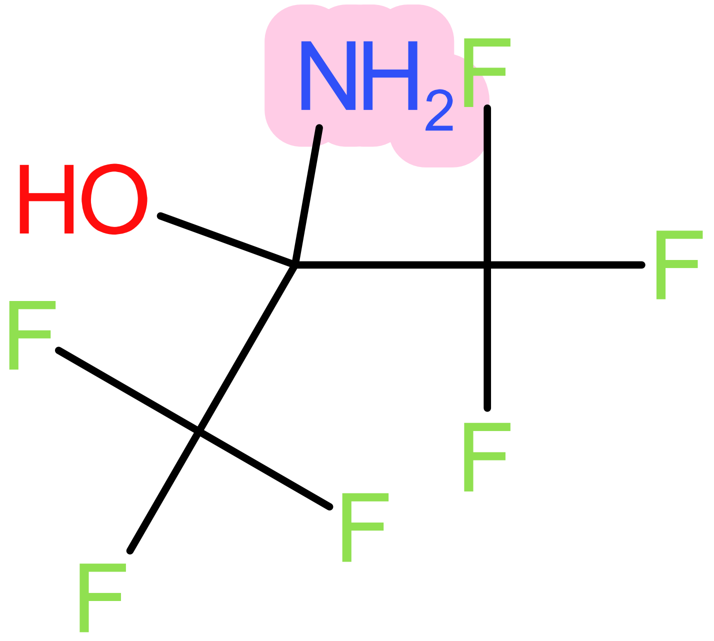

S31:Perfluorohexanesulfonamide (PFHxSA)
SMILES: NS(=O)(=O)C(F)(F)C(F)(F)C(F)(F)C(F)(F)C(F)(F)C(F)(F)F

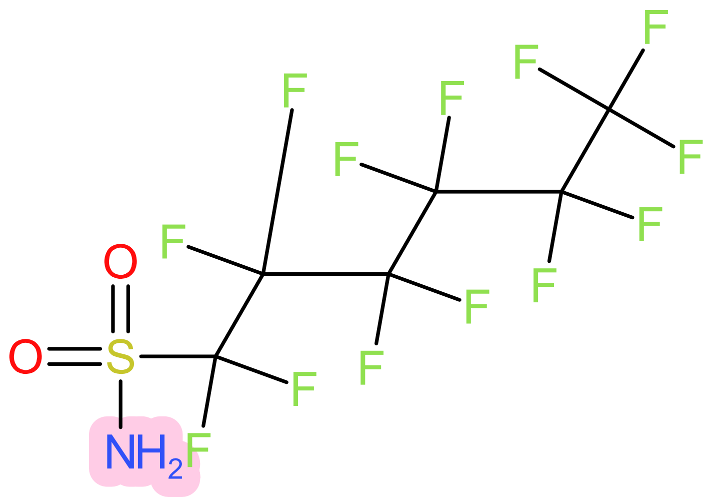

S48:Perfluorooctanamidine (PFOAMD)

SMILES: NC(=N)C(F)(F)C(F)(F)C(F)(F)C(F)(F)C(F)(F)C(F)(F)C(F)(F)F

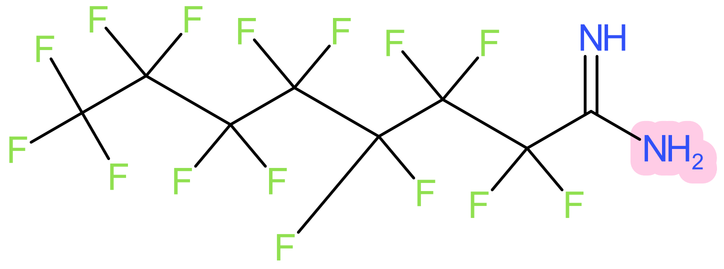

SGR10099

S31: Perfluorohexanesulfonamide (PFHxSA)

SMILES: NS(=O)(=O)C(F)(F)C(F)(F)C(F)(F)C(F)(F)C(F)(F)C(F)(F)F

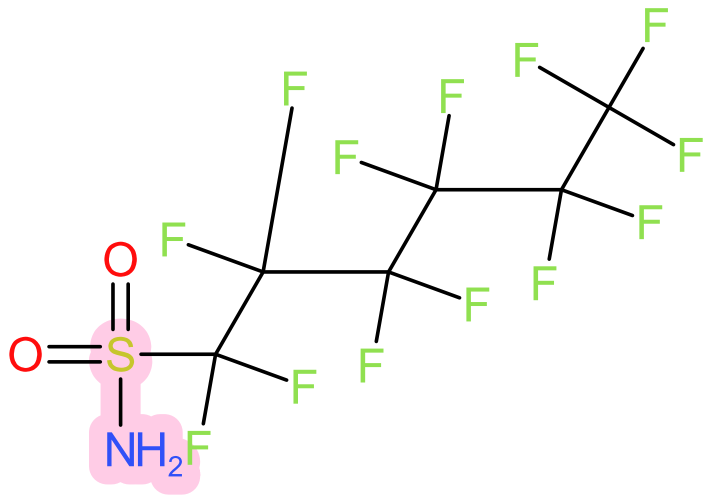

S43: N-Methyl-N-(2-hydroxyethyl)perfluorooctanesulfonamide (MeFOSE)

SMILES: CN(CCO)S(=O)(=O)C(F)(F)C(F)(F)C(F)(F)C(F)(F)C(F)(F)C(F)(F)C(F)(F)C(F)(F)F

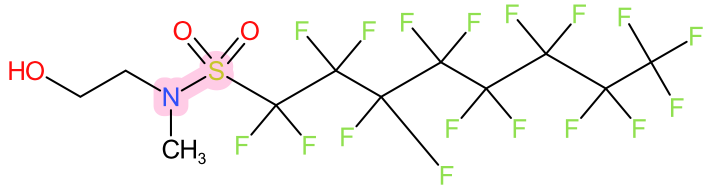

SGR10289

S2: 3-(Perfluoro-2-butyl)propane-1,2-diol (PFHp2OH)
SMILES: OCC(O)CC(F)(F)C(F)(F)C(F)(F)C(F)(F)F

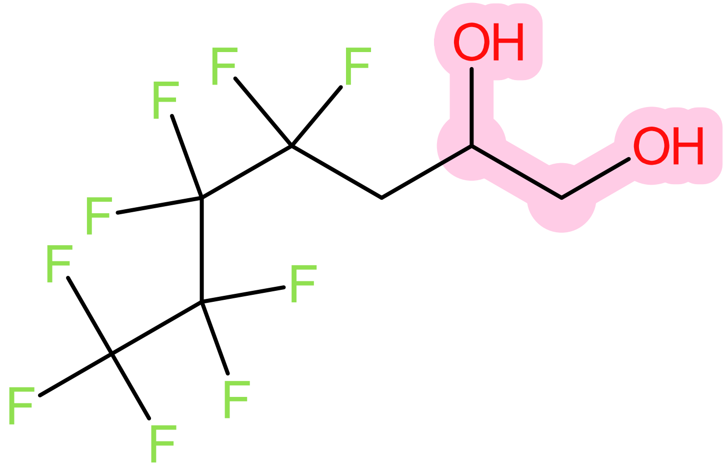

S40:1-(Perfluorofluorooctyl)propane-2,3-diol (PFUd2OH)
SMILES: OCC(O)CC(F)(F)C(F)(F)C(F)(F)C(F)(F)C(F)(F)C(F)(F)C(F)(F)C(F)(F)F

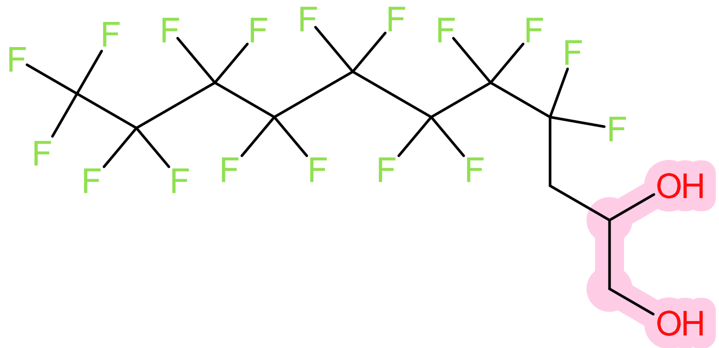

SGR10587

S31: Perfluorohexanesulfonamide (PFHxSA)
SMILES: NS(=O)(=O)C(F)(F)C(F)(F)C(F)(F)C(F)(F)C(F)(F)C(F)(F)F

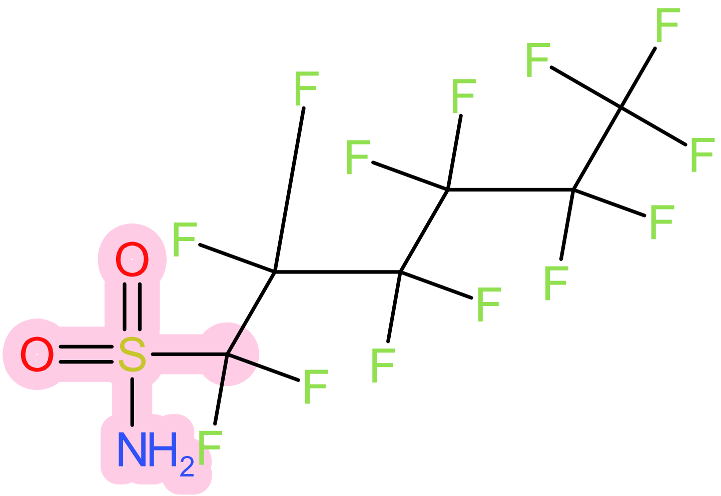

S43: N-Methyl-N-(2-hydroxyethyl)perfluorooctanesulfonamide (MeFOSE)
SMILES: CN(CCO)S(=O)(=O)C(F)(F)C(F)(F)C(F)(F)C(F)(F)C(F)(F)C(F)(F)C(F)(F)C(F)(F)F

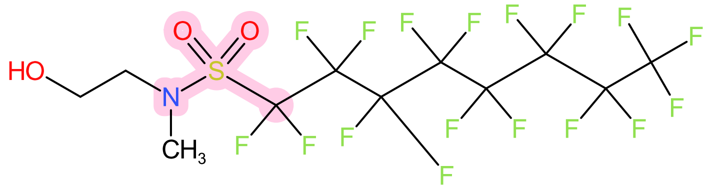

TOPOLOGY

SGR10704 (2 chem)
SGR10749 (2 chem)

SGR10704

S22: Perfluoro-3,6,9-trioxatridecanoic acid (PFPE-6)
SMILES: OC(=O)C(F)(F)OC(F)(F)C(F)(F)OC(F)(F)C(F)(F)OC(F)(F)C(F)(F)C(F)(F)C(F)(F)F

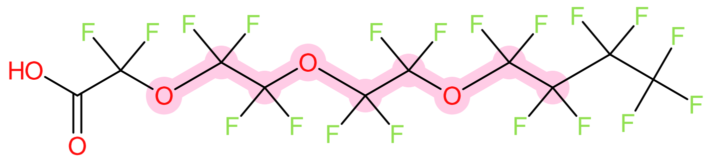

S56: Fluorinated triethylene glycol monomethyl ether (C7F3ETOH)
SMILES: OCC(F)(F)OC(F)(F)C(F)(F)OC(F)(F)C(F)(F)OC(F)(F)F

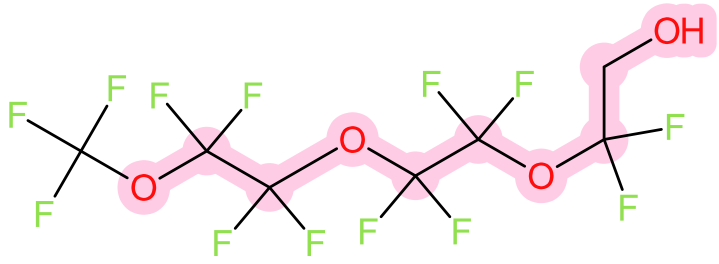

SGR10749

S2: 3-(Perfluoro-2-butyl)propane-1,2-diol (PFHp2OH)
SMILES: OCC(O)CC(F)(F)C(F)(F)C(F)(F)C(F)(F)F

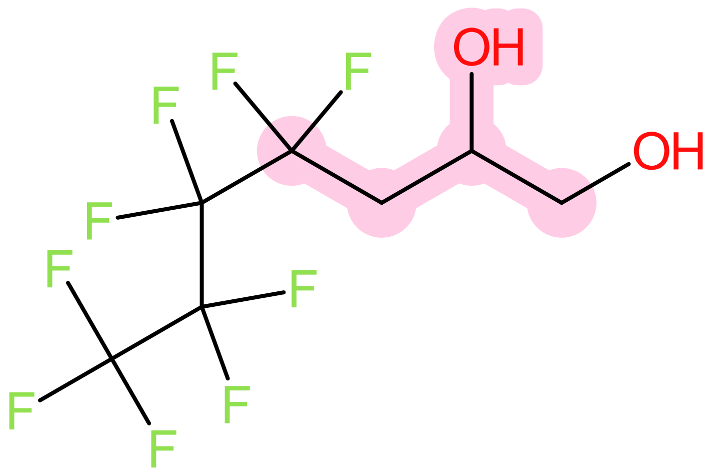

S40: 1-(Perfluorofluorooctyl)propane-2,3-diol (PFUd2OH)
SMILES: OCC(O)CC(F)(F)C(F)(F)C(F)(F)C(F)(F)C(F)(F)C(F)(F)C(F)(F)C(F)(F)F

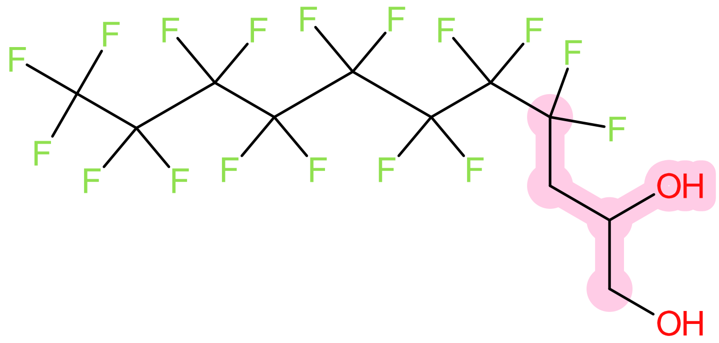

Supplement: Supplementary file 48 — Supplementary Material 48 [file 40246_2024_665_MOESM48_ESM.pdf]
